# Bridging and Conformational Control of Porphyrin Units through Non‐Traditional Rigid Scaffolds

**DOI:** 10.1002/chem.201904199

**Published:** 2020-01-21

**Authors:** Nitika Grover, Gemma M. Locke, Keith J. Flanagan, Michael H. R. Beh, Alison Thompson, Mathias O. Senge

**Affiliations:** ^1^ School of Chemistry SFI Tetrapyrrole Laboratory Trinity College Dublin Trinity Biomedical Sciences Institute The University of Dublin 152-160 Pearse Street Dublin 2 Ireland; ^2^ Department of Chemistry Dalhousie University P.O. Box 15000 Halifax, Nova Scotia B3H 4R2 Canada; ^3^ Physics Department E20 Technische Universität München James-Franck-Str. 1 85748 Garching Germany; ^4^ Institute for Advanced Study (TUM-IAS) Technische Universität München Lichtenberg-Str. 2a 85748 Garching Germany

**Keywords:** bicyclo[1.1.1]pentane, cubane, molecular tweezers, porphyrin arrays, supramolecular chemistry

## Abstract

Connecting two porphyrin units in a rigid linear fashion, without any undesired electron delocalization or communication between the chromophores, remains a synthetic challenge. Herein, a broad library of functionally diverse multi‐porphyrin arrays that incorporate the non‐traditional rigid linker groups cubane and bicyclo[1.1.1]pentane (BCP) is described. A robust, reliable, and versatile synthetic procedure was employed to access porphyrin‐cubane/BCP‐porphyrin arrays, representing the largest non‐polymeric structures available for cubane/BCP derivatives. These reactions demonstrate considerable substrate scope, from utilization of small phenyl moieties to large porphyrin rings, with varying lengths and different angles. To control conformational flexibility, amide bonds were introduced between the bridgehead carbon of BCP/cubane and the porphyrin rings. Through varying the orientation of the substituents around the amide bond of cubane/BCP, different intermolecular interactions were identified through single crystal X‐ray analysis. These studies revealed non‐covalent interactions that are the first‐of‐their‐kind including a unique iodine‐oxygen interaction between cubane units. These supramolecular architectures indicate the possibility to mimic a protein structure due to the sp^3^ rigid scaffolds (BCP or cubane) that exhibit the essential conformational space for protein function while simultaneously providing amide bonds for molecular recognition.

## Graphical abstract

**Synthesis and characterization** of novel porphyrin‐cubane/BCP arrays have been reported. The single crystal analysis revealed supramolecular 3D networks with combined and repetitive inter‐ and intramolecular H‐bonding interactions.

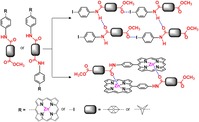

## Introduction

Defined molecular architectures are a prerequisite for the logical construction of multifunctional chemical systems. In carbon‐based covalent systems the individual effector units are typically linked by either conjugating sp‐ or sp^2^‐hybridized units or by flexible sp^3^‐hybridized bridges. The use of short, robust, and spatially defined aliphatic linker units opens new avenues with their potential application as molecular isolators, resistors and rigid scaffolds, alongside the benefit of their inherent materials properties.

Synthetic chemists are continually seeking to prepare new rigid multi‐porphyrin architectures due to their potential applications as organic conducting materials, near‐infrared (near‐IR) dyes, nonlinear optical materials, and molecular wires.^1^ A number of synthetic strategies have been employed to access these multi‐porphyrin arrays using approaches such as: (a) connecting the porphyrin units via phenylene, ethynyl, ethenyl or alkane linkers^2^ or (b) connecting two or more *meso*–*meso*‐linked porphyrin units via oxidative fusing reactions.^3^ However, most of the porphyrin arrays reported have problems such as poor solubility, synthetic inaccessibility, and conformational heterogeneity. In *meso*–*meso*‐linked porphyrin arrays, the porphyrin units are orthogonal to one another which can cause a significant energy/charge sink. Furthermore, porphyrin arrays joined directly by π‐conjugated linkers exhibit significantly altered UV/Vis spectra, indicating very strong electronic coupling, that is, loss of the characteristic of individual units due to delocalization of π‐electrons. Hence, it is necessary to design a molecule which can predictably exhibit a desired energy‐ and/or electron‐transfer process that is achievable without effecting electronic delocalization and/or an energy sink.^1^, ^2^, ^3^ A straightforward strategy for avoiding any undesirable overlap of the π‐systems may be to attach two porphyrin skeletons through non‐traditional rigid scaffolds such as bicyclo[1.1.1]pentane (BCP) or cubane. These saturated entities are transparent to UV/Vis light and exhibit specific three‐dimensional (3D) arrangements of the bridgehead carbons. This positions the chromophoric units in a rigid and linear fashion without any electron delocalization or conjugation such as to potentially reduce the drawbacks previously outlined.^4^

**Figure 1:**
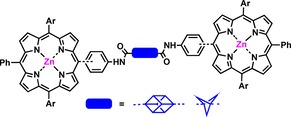
Schematic representation of synthesized porphyrin arrays.

Herein, we report the first synthesis of porphyrin dimers that utilize either BCP or cubane as a rigid linear scaffold (Figure 1). This library of BCP/cubane porphyrin arrays contains some of the largest non‐polymeric structures available for cubane and BCP.

1,4‐Disubstituted cubane is a well‐known bioisostere of *para*‐substituted phenylene rings due to the similar distance across the cube body diagonal of 2.72 Å vs. 2.79 Å for a benzene ring.^5^ BCP is the smallest member of the bicyclic alkane family, in terms of actual size rather than in terms of atoms present. Indeed, BCP exhibits the shortest non‐bonded distance between bridgehead carbon atoms of 1.85 Å, which is closer in bond length to ethyne (1.20 Å).^6^ The 3D, compact, electronically isolating, and saturated structures of cubane and BCP enable them to avoid undesirable π–π stacking which may lead to improved solubility of chromophoric arrays. Despite their desirable well‐defined dimensions and rigid‐rod geometries, the chemistry of these moieties is undeveloped, particularly in terms of functionalization or C−H‐activation at the bridgehead carbons.^7^

BCP and cubane are transparent to UV/Vis light and most often their application is restricted to bioisosteres^8^ and crystal engineering.^9^ The structural pre‐organization and high thermal stability of these compounds make them attractive candidates by which to link two chromophoric units, but their use has been neglected so far.7a, 10 The limited use of these non‐traditional scaffolds is due to perceived complex synthetic procedures and limited commercial supply chain of precursors. In addition, appending rigid sp^3^ linkers as connectors between two chromophoric units is synthetically demanding.

Recent synthetic developments by Baran, Aggarwal and ourselves include methods based on decarboxylative sp^3^ C−C coupling to functionalize the bridgehead carbons of cubane and BCP.^11^ Knochel and co‐workers have also reported an efficient method to synthesize 1,3‐bisaryl substituted BCPs.^12^ Additionally, amide bonds have been introduced at the bridgehead carbons of cubane and BCP,^13^ however most of these reported moieties were used as bioisosteres^8^ or in crystal engineering.^9^ Yet, these compounds also have the potential to be utilized as molecular building blocks. Moreover, cubane/BCP could be implemented as rigid scaffolds linking two chromophores while providing a synthetic handle for molecular recognition of small molecules or ions.

**Figure 2:**
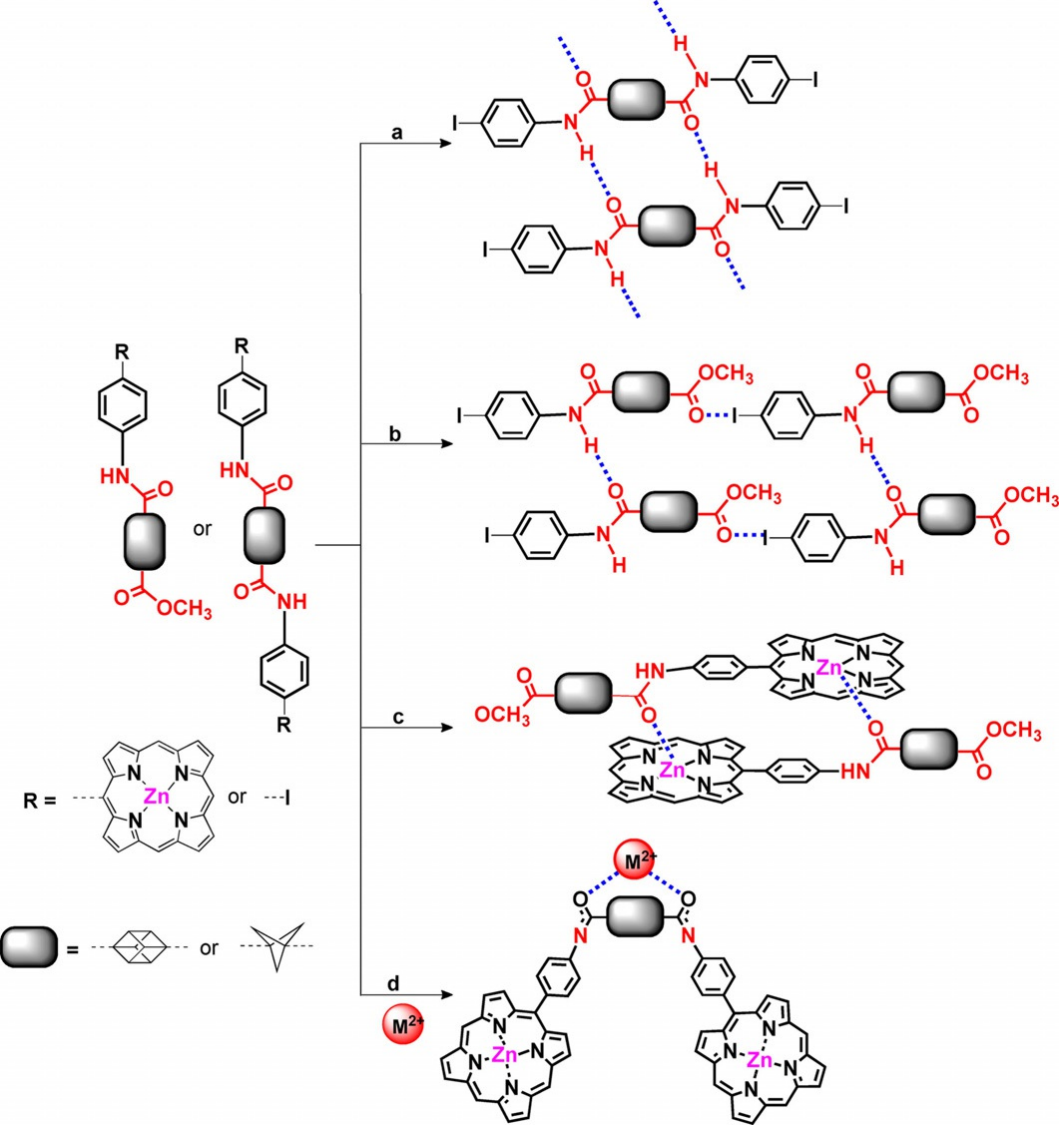
Schematic representation of potential interactions between amide‐connected BCP/cubane derivatives: (a) H‐bonding interactions; (b) iodine⋅⋅⋅oxygen interactions; (c) intermolecular axial interactions; (d) potential interaction site with external metal ions.

Building upon the progress made in synthetically accessing these non‐traditional rigid scaffolds, we envisioned appending BCP/cubane between two porphyrin units in a conformationally controlled manner. The utilization of semi‐rigid amide bonds for the attachment of a porphyrin skeleton to a rigid scaffold introduces a controlled conformational flexibility into porphyrin dyad(s). This allows significant modulation of the photophysical properties in the porphyrin dyad(s) through the coordination of transition metal(II) ions (Figure 2). By varying the distance and angles between the two chromophores it is hoped that the extent of the impact that cation coordination has on the photophysical properties of a multi‐chromophoric tweezer‐like system can be investigated.

As cubane and BCP are rigid and relatively inert scaffold, the amide bonds are the only variable in the system and a true measurement of their role in the conformational changes can thus be undertaken. We herein present porphyrin units bridged through non‐traditional BCP/cubane connectors as a test case for multichromophoric and/or electroactive systems in general.

## Results and Discussion

### Synthesis and characterization

The amide bond is crucially important as one of the main chemical linkages found in biologically and pharmaceutically active compounds.^14^ Amide bonds exhibit a planar *trans* configuration of the N−H and C=O moieties and undergo very little rotation or twisting around the bond due to amido–imido tautomerization. The semi‐rigid nature of amide bonds enables conformational control over the molecular architecture of the compound they are part of courtesy of hydrogen bonds and the coordination of metal ions (Figure 2). To this end, the synthetic design of the current project focused on the functionalization of the bridgehead BCP/cubane carbons through amide bonds. Firstly, we started with the synthesis of small rigid building blocks. Carboxylic acid derivatives of cubane (**1** and **2**) were reacted^15^ with substituted aryl amines **3**–**12** to access the amide derivatives **13**–**29**. The use of HATU/HOAt as an activating agent in presence of DIPEA in DMF at 25 °C provided the most suitable synthetic reaction condition by which to access amide substituted cubanes (**13**–**29**).

**Scheme 1:**
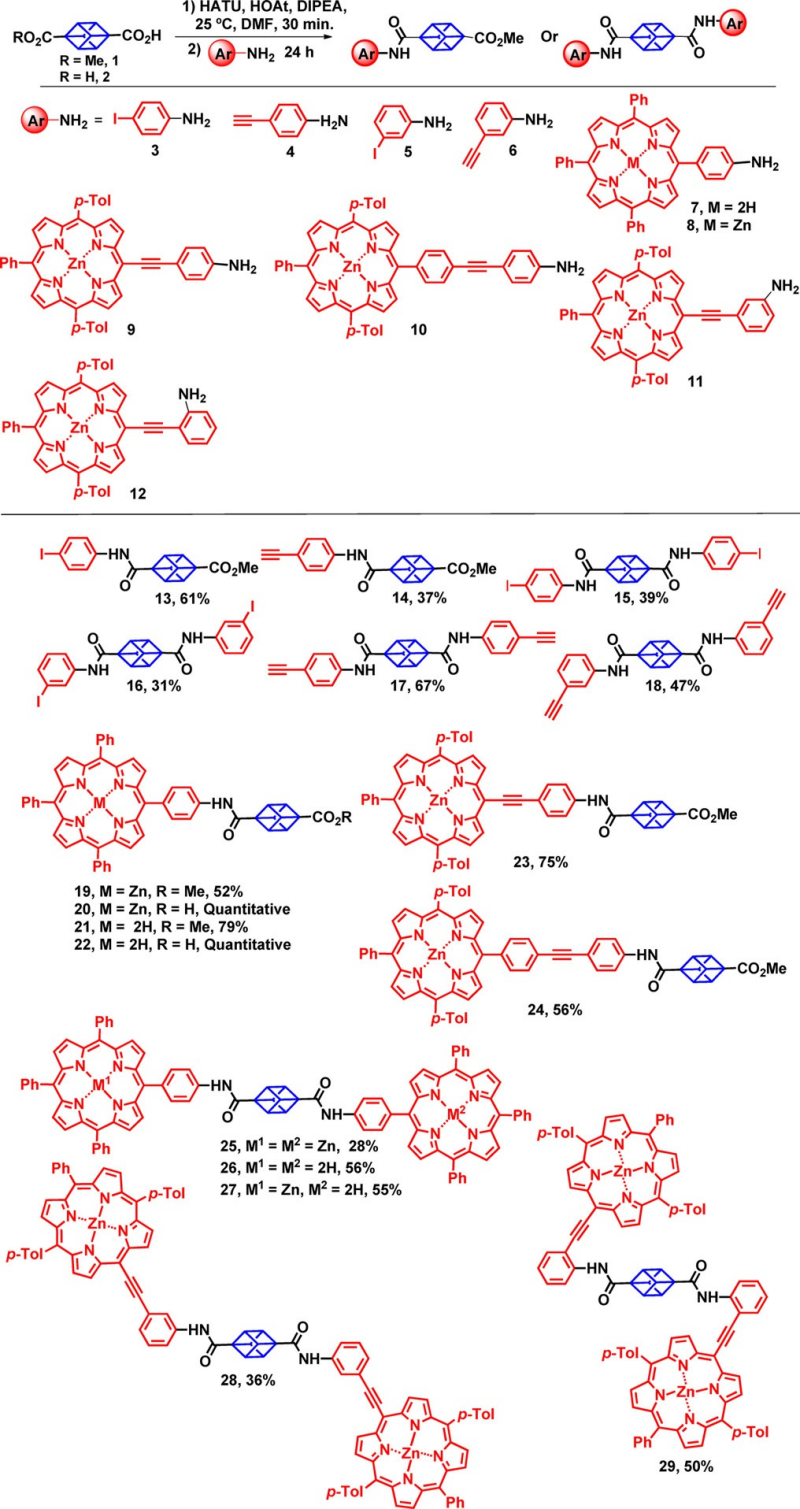
Amide coupling of cubane moieties **1** and **2** with amines **7**–**12**.

The reaction of 4‐methoxycarbonylcubane‐1‐carboxylic acid (**1**) with 4‐iodoaniline (**3**) proceeded smoothly in yield of 61 %. Similarly, we could couple 4‐ethynylaniline (**4**) and 4‐methoxycarbonylcubane‐1‐carboxylic acid (**1**) to access cubane **14** in a 37 % yield. Further, cubane‐1,4‐dicarboxylic acid was reacted with anilines **3** and **4** in above mentioned reaction conditions to afford the cubanes **15** and **17** in yields of 39 % and 67 %. The preliminary substrate scope was investigated by incorporating various combinations of structural motifs such as *meta*‐iodo/ethynyl‐substituted anilines to access the amide substituted cubanes **16** and **18**. (Scheme 1).

Generally, *meta*‐/*para*‐ethynyl‐substituted anilines reacted more efficiently with cubane‐1,4‐dicarboxylic acid (**2**) compared to those with iodo substituents. Attempts to purify the crude cubane compounds (**13**–**18**) via column chromatography, using silica gel, were mostly unfruitful due to degradation of the product on the silica gel. However, recrystallization from CH_2_Cl_2_ and excess hexane proved very effective in removing any remaining aniline and other impurities.

To demonstrate the potential value of this method, we next examined the scope of *meso*‐amine‐substituted porphyrins in amide coupling reactions with cubane **1** and **2**. 5‐(4′‐aminophenyl)‐10,15,20‐triphenylporphyrin (**7**) and its zinc(II) complex (**8**) were synthesized by mono‐nitration of TPP followed by reduction using procedures reported in the literature.^16^ Table 1 outlines the different reaction conditions employed to optimize the amide coupling reaction between **1** and **7**. The use of HATU/HOAt in presence of DIPEA furnished the amide‐coupled cubane‐porphyrin array **21** in 79 % isolated yield. However, the use of other activating agents such as DIC and ethylchloroformate in the presence of TEA or DMAP also resulted in the formation of product **21**, albeit in lower yields of 43 % and 31 %, respectively (Table 1). Reaction of porphyrin **8** with cubane **1** shows the neat conversion of porphyrin **8** into the cubane porphyrin array **19** in 52 % yield.

**Table 1:** Test C−H activation reactions for the amide coupling of 4‐(methoxycarbonyl)cubane‐1‐carboxylic acid (**1**) with 5‐(4′‐aminophenyl)‐10,15,20‐triphenylporphyrin (**7**).

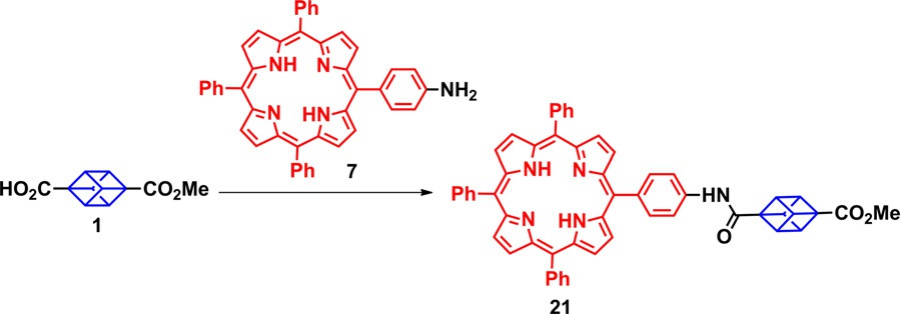					
Cubane [equiv]	Activating agent [equiv]	Base	Sol.	*t* [h]	Yield
2.0	DIC (1.3)	DMAP	THF	48	65
2.0	ECF^[a]^ (1.3)	NEt_3_	CHCl_3_	120	75
6.0	DIC (1.3)	DMAP	THF	48	65
3.0	HATU/HOAt (1.3/1.3)	DIPEA	DMF	24	25

[a] ECF=Ethylchloroformate.

Notable compounds **19** and **21** were subjected to further functionalization. Base hydrolysis of zinc and free base substituted porphyrin **19** and **21** yielded the carboxylic acid derivatives of cubane–porphyrin array **20** and **22** respectively, in quantitative yields. Similarly, the reaction of cubane‐1,4‐ dicarboxylic acid (**2**) with **7** and **8** resulted in access to the very first porphyrin–cubane–porphyrin arrays **25** and **26**, respectively. Next we attempted the synthesis of Zn^II^–porphyrin–cubane‐free base porphyrin array **27** via amide coupling reaction of amine substituted porphyrin **7** and carboxylic acid substituted cubane porphyrin **20**. Use of optimized reaction conditions resulted in unsymmetric dimer **27** in a 55 % yield.

To overcome the low solubility of phenyl substituted porphyrins we changed to more soluble 4′‐methylphenyl substituted amine porphyrins for further reactions. [5‐(4′‐Aminophenylacetylene)‐10,20‐bis(4′‐methylphenyl)‐15‐phenylporphyrinato]zinc(II) (**9**), [5‐(4′‐(4′′‐ethynylaniline)phenyl)‐10,20‐bis(4′‐methylphenyl)‐15‐phenylporphyrinato]zinc(II) (**10**), [5‐(3′‐aminophenylacetylene)‐10,20‐bis(4′‐methylphenyl)‐15‐phenylporphyrinato]zinc(II) (**11**) and [5‐(2′‐aminophenylacetylene)‐10,20‐bis(4′‐methylphenyl)‐15‐phenylporphyrinato]zinc(II) (**12**) were synthesized via modified Sonogashira reaction conditions by reacting the corresponding ethynyl anilines with [5‐iodo‐10,20‐bis(4′‐methylphenyl)‐15‐phenylporphyrinato]zinc(II) in the presence of Pd(PPh_3_)_2_Cl_2_ (0.15 equiv) and CuI (0.3 equiv) in THF/NEt_3_. Amide coupling of **9** and cubane **1** proceed efficiently and provide convenient access to porphyrin **23** in isolated yield of 75 %. Similarly, porphyrin **24** was achieved in 56 % yield by reacting porphyrin **10** with cubane **1**. Additionally, we tried the synthesis of cubane linked porphyrin dimers by using *meta*‐ and *ortho*‐substituted aniline **11** and **12**. Reaction of **11** and cubane **2** resulted into the formation of dimer **28** in 36 % yield whereas *ortho*‐dimer **29** was synthesized in 50 % yield via reaction of *ortho*‐amine substituted porphyrin **12** and cubane **2**. This robust reaction demonstrated considerable scope, from the utilization of small benzene rings to large porphyrin systems with varying lengths and at different angles with respect to the cubane plane.

**Scheme 2:**
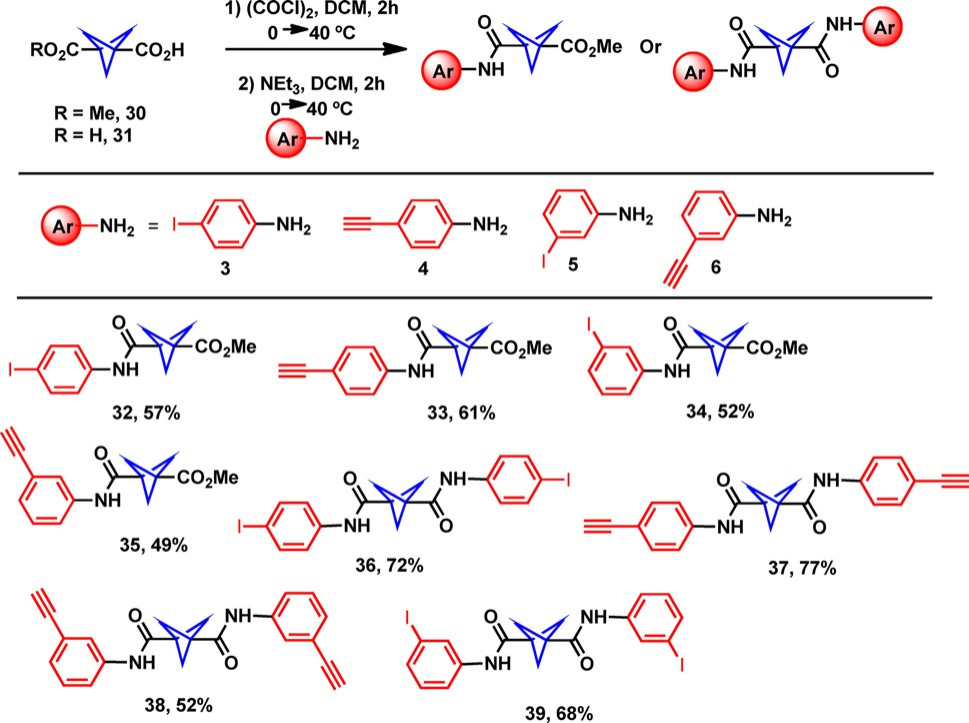
Amide coupling at bridgehead carbon of BCP moiety and substrate scope.

The successful functionalization of cubane motivated us to next attempt functionalization of the BCP using the same amide coupling method. However, initial attempts to synthesize the required BCP building blocks using HATU/HOAt, EDC, or DIC were unsuccessful. This may be due to unstable BCP intermediates capable of undergoing rearrangement to result in ring‐opened moieties. The BCP carboxylic acid was instead reacted with (COCl)_2_/TEA, followed by the desired amine. Initially, this reaction was conducted at room temperature similar to the cubane analogues above, however, this was met with limited success as the product was detected only in a small amounts by ^1^H NMR spectroscopy and mass spectrometry. This limited success was mitigated by the use of an elevated reaction temperature of 40 °C for both steps, which resulted in a significant increase in the product yields (49–77 %) (Scheme 2). The crude reaction mixtures were purified via recrystallization using a small amount of CH_2_Cl_2_ and excess hexane to access the desired products as white powders. The reaction of BCP **30** with aniline **3** and **4** resulted into the formation of **32** and **33** in 57 % and 61 % yield. Next, the *meta*‐phenyl substituted BCPs **34** and **35** were synthesized via amide coupling reaction of BCP **30** and aniline **5** and **6**, respectively. Amide coupling of BCP **31** and anilines **3**–**6** resulted into the formation of amide substituted BCPs **36**–**39**. We had found that the amide coupling reactions proceed optimally with *meta*‐/*para*‐substituted aniline in presence of (COCl)_2_/TEA. Unfortunately, amide coupling with *ortho*‐substituted anilines resulted in degradation. The ineffective *ortho*‐substituted aniline coupling may be caused by the proximity of the amine and iodo/ethynyl groups, enabling H‐bonding interactions between the moieties, ultimately reducing the basicity and reactivity of the amine. On the other hand, the cubane porphyrin dimer **29** was accessed in a 50 % yield due to the replacement of the small H‐bonding moieties with a porphyrin, preventing the reduced amine basicity.

**Scheme 3:**
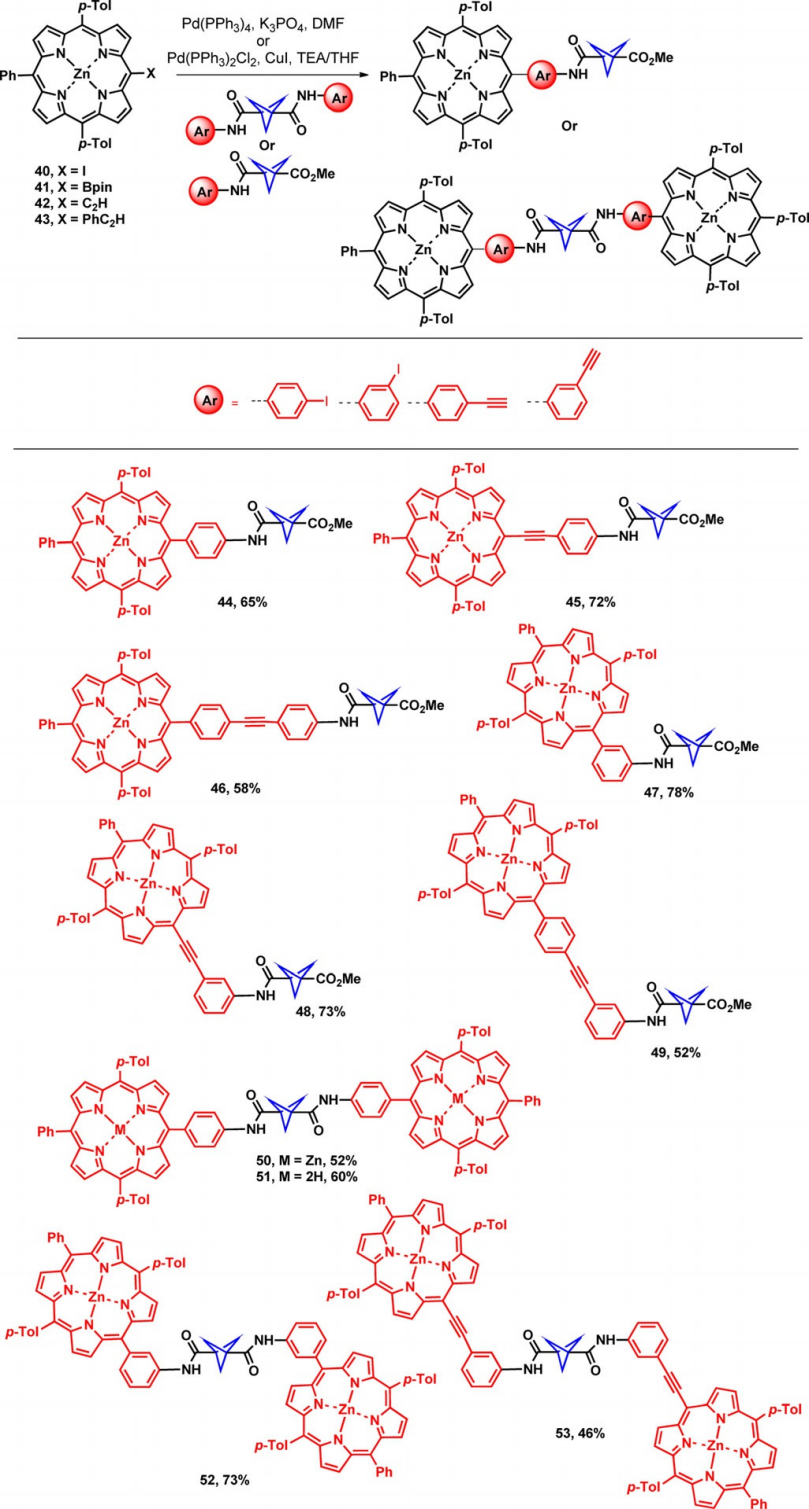
Suzuki [Pd(PPh_3_)_4_, K_3_PO_4_, DMF ] or Sonogashira coupling (Pd(PPh_3_)_2_Cl_2_, CuI, TEA/THF) reaction conditions and substrate scope.

The synthesized BCP‐based building blocks **32**–**39** were further subjected to Pd‐catalyzed cross‐coupling reactions to yield porphyrin‐BCP conjugates (**44**–**53**), as shown in Scheme 3. Pd‐Catalyzed cross‐coupling reactions are versatile and straightforward approaches for porphyrins to form carbon−carbon bonds with a wide range of functionalities.1b, 17 In contrast to cubane,^18^ the BCP ring is more tolerant towards Pd‐catalyzed cross‐coupling reactions.^19^ The first BCP‐porphyrin array **44** was afforded using Suzuki–Miyaura cross‐coupling reaction of [5‐(4′,4′,5′,5′‐tetramethyl‐1′,3′,2′‐dioxaborolan‐2′‐yl)‐10,20‐bis(4′‐methylphenyl)‐15‐phenylporphyrinato]zinc(II) (**41**)^20^ and BCP (**33**). The same synthetic strategy was employed to access the *meta*‐linked BCP porphyrin array **47** in 78 % yield. Neat conversion of **41** into **44** and **47** encouraged us to try further attempts to synthesize the porphyrin‐BCP‐porphyrin arrays. The BCP porphyrins **50** and **52** were synthesized through Suzuki–Miyaura cross‐coupling reactions with [5‐(4′,4′,5′,5′‐tetramethyl‐1′,3′,2′‐dioxaborolan‐2′‐yl)‐10,20‐bis(4′‐methylphenyl)‐15‐phenylporphyrinato]zinc(II) (**41**)^20^ and BCPs **36** and **39**, respectively. The isolated yields of *meta*‐derivatives **47** and **52** were higher as compared to those of corresponding *para* derivatives (**44** and **50**).

The porphyrin arrays **45** and **46** were synthesized using Sonogashira cross‐coupling reactions of [5‐ethynyl‐10,20‐bis(4′‐methylphenyl)‐15‐phenylporphyrinato]zinc(II) (**42**) and [5‐(4′‐ethynylphenyl)‐10,20‐bis(4′‐methylphenyl)‐15‐phenylporphyrinato]zinc(II) (**43**) and BCP **32**. The Sonogashira reactions did not proceed well for the synthesis of *meta*‐derivatives **48** and **53**, while a copper‐free modified Sonogashira reaction of [5‐iodo‐10,20‐bis(4′‐methylphenyl)‐15‐phenylporphyrinato]zinc(II) (**40**)^21^ with BCPs **35** and **38** proceeded well to access the porphyrin‐BCP arrays **48** and **53**, in 73 % and 46 % yield, respectively. Detailed synthetic procedures are described in the Supporting Information. All newly synthesized compounds were characterized by ^1^H NMR, ^13^C NMR, UV/Vis, and IR spectroscopic methods as well as via MALDI‐TOF‐MS spectrometry (see Figures S1–S135 in Supporting Information).

UV/Vis spectra of the chromophore arrays were recorded in CHCl_3_ or THF at room temperature. The free base dimers **26** and **51** illustrate a typical etio‐type porphyrin spectrum, having the Soret and four Q‐bands in decreasing intensity. The symmetrical zinc dimers **25**, **28**, **29**, **50**, **52**, and **53** showed an absorbance maximum at 422 nm. The full width at half maxima (FWHM) of these dimers is nearly equal to 5,10,15,20‐tetraphenylporphyrin (H_2_TPP) or its zinc(II) complex (ZnTPP), displaying no evidence of exciton coupling between two porphyrin units, which supports the suggested potential *trans* conformation of one porphyrin unit with respect to another unit.^22^ The absorption spectra of ethynyl‐linked porphyrin dimers such as **28**, **29**, and **53** exhibited a 15–18 nm bathochromic shift compared to the phenylene‐linked dimers **25** and **50** due to the π‐extended ethynyl or phenylethynyl moieties. The UV/Vis spectra of ethynyl‐linked dimers (**28**, **29**, and **53**) exhibit nearly the same FWHM and *λ*_max_ as compared to the precursor amine porphyrin (**11** and **12**). Similar *λ*_max_ values of monomers and dimers indicate the lack of through space or through bond electronic communication between porphyrin units, that is, a *trans* orientation of the synthesized dimers.

### Single crystal X‐ray analysis

The structure of compounds [5‐(2′‐aminophenylacetylene)‐10,20‐bis(4′‐methylphenyl)‐15‐phenylporphyrinato]zinc(II) (**12**), cubane **13**, BCP **33**, **35**, **38**, **45**, **46** and dimethyl bicyclo[1.1.1]pentane‐1,3‐dicarboxylate were determined using single crystal X‐ray diffraction analysis. Structural parameter tables and refinement details (Table S1 and S2) are provided in the Supporting Information.^23^, ^24^

**Figure 3:**
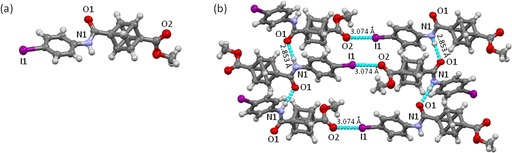
(a) Molecular structure of cubane **13**. (b) Molecular arrangement of compound **13** in the crystal shows the non‐covalent interactions between N1⋅⋅⋅O1=C and C=O2⋅⋅⋅I1.

The crystal structure of cubane scaffold **13** illustrates two types of intermolecular non‐covalent interactions (Figure 3). The structure exhibits a head‐to‐tail N1⋅⋅⋅O1=C interaction at a distance of 2.853 Å with an angle of 175.4°. Furthermore, the iodo atom at the *para*‐position of the phenylene moiety exhibits a head‐to‐tail halogen bond interaction with O2=C of the ester group with a distance of 3.074 Å and an angle of 170.1°. The observed halogen and hydrogen bond interactions are nearly orthogonal to each other. Interestingly, the combined and repetitive intermolecular halogen and hydrogen bond interactions result in a supramolecular 3D network between the cubane molecules directed by the substituents at 1,4‐bridgehead positions. There are only a few reports of oxygen‐iodine interactions,^25^ but the specific 3D‐orientation of cubane potentially favors this packing pattern enabling it to access this unusual interaction.

**Figure 4:**
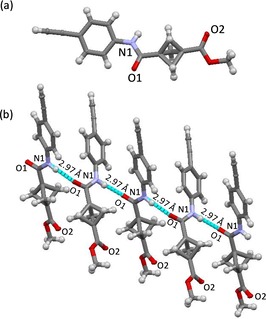
(a) Molecular structure of compound **33**. (b) Molecular arrangement of compound **33** in the crystal shows the non‐covalent interaction between N1⋅⋅⋅O1.

The crystal structures of BCP **33**, **35**, and **38** exhibit non‐covalent interactions between amide N‐donors and C=O acceptors within the crystal lattices. The nature of these interactions is dependent upon the substitution pattern at the phenylene ring. The crystal structure of *para*‐substituted BCP compound **33** reveals repetitive head‐to‐head N1⋅⋅⋅O1=C hydrogen‐bond interactions at distances of 2.970 Å (Figure 4). In contrast to **33**, the crystal structure of *meta*‐substituted BCP **35** exhibits head‐to‐tail N1⋅⋅⋅O2=C interactions at distances of 3.061 Å, leading to the formation of a non‐covalently attached inversion‐centered dimer (Figures S138 and S139 in Supporting Information).

Similarly, the crystal structure of bis‐*meta*‐substituted BCP **38** shows head‐to‐tail interactions at distances of 2.910 Å and forms a supramolecular 3D network/array (Figure S141 in Supporting Information).

In nature, the 3D structures of proteins and other biomolecules are controlled using H‐bonding interactions between *trans* N−H and C=O moieties of amino acids and these 3D architectures are responsible for their specific biological functions. The substituents surrounding the amide bonds direct the non‐covalent interactions in all of the above‐mentioned crystal structures, and this indicates the possibility of potentially mimicking protein architecture with sp^3^ rigid scaffolds (BCP or cubane). This would provide the conformational space essential for protein function, while simultaneously providing amide bonds for “substrate” coordination.

**Figure 5:**
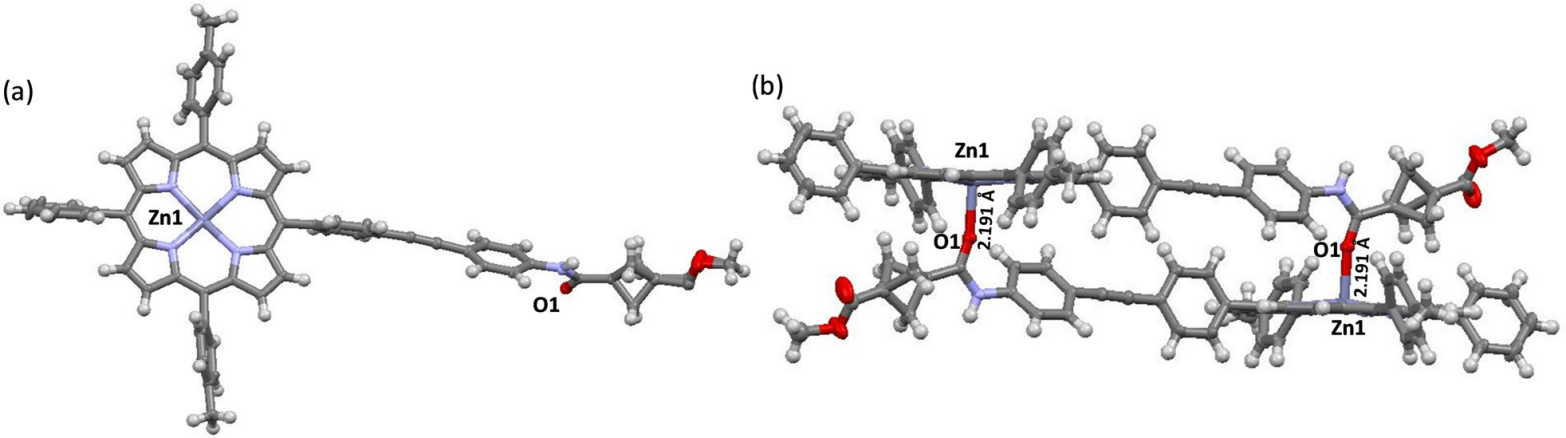
(a) Molecular structure of compound **46**. (b) Intermolecular head‐to‐tail interaction between the Zn metal of the porphyrin unit (acceptor) and the C=O donor moiety of the amide bond.

The crystal structure of the BCP‐porphyrin **46** illustrates the planar conformation of the macrocyclic core while the crystal packing of this molecule further shows intermolecular head‐to‐tail non‐covalent D⋅⋅⋅A interactions between the acceptor Zn^II^ metal of the porphyrin and donor oxygen atom of the carbonyl group in the amide moiety at a distance of 2.191 Å (Figure 5). This particular interaction supports the proposed mechanism of binding between a transition metal(II) and the C=O moiety of the amide bond (vide infra). Similarly, the crystal structure of [5‐(2′‐aminophenylacetylene)‐10,20‐bis(4′‐methylphenyl)‐15‐phenylporphyrinato]zinc(II) (**12**) also exhibits head‐to‐tail D⋅⋅⋅A interactions, in this case between the donor N‐atom of the amine group from one porphyrin to the Zn^II^ center of another (Figure S145 and S146 in the Supporting Information). This interaction is further supported by ^1^H NMR spectra where the *ortho*‐amine protons resonate at −0.58 ppm due to the shielding effect of the porphyrin ring current.

**Figure 6:**
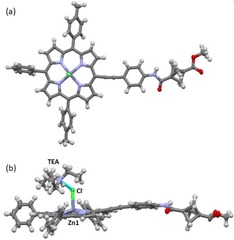
(a) Molecular structure of compound **45**. (b) Charge separated ion pair complex of porphyrin **45** and [HNEt_3_]^+^.

Along with the above mentioned non‐covalent interactions, we also observed a unique example of a porphyrin‐based ion pair complex, that is, a pair of opposite charges held together by Columbic interactions in the same solvent‐shell.^26^ Although charge‐separated ion pair complexes are quite common in transition‐metal organometallic chemistry, ion pair complexes of phlorins and porphodimethene‐based systems are also known, this type of interaction has not previously been observed for systems with intact porphyrin cores.^27^ The crystal structure of porphyrin **45** is unique as it exhibits an ion pair interaction between an axial chloride ligand and a [Et_3_NH]^+^ counter ion in the same unit cell without disturbing the aromatic 18π‐electron pathway (Figure 6). Axial coordination results in displacement of the Zn^II^ ion from the 24‐atom mean plane by 0.51 Å. The chloride and triethylammonium ions exhibit an ion pair interaction at a distance of 3.043 Å. This is further supported by the ^1^H and ^13^C NMR spectra of compound **45** where the ratio of the porphyrin derivative and [Et_3_NH]^+^ was found to be 1:1. To best of our knowledge, it is the first example of a porphyrin‐based charge‐separated ion pair complex. Figure 6 illustrates an example of the [Et_3_NH]^+^ [porphyrin(ZnCl)]^−^ ion pair in which a negatively charged porphyrin electrostatically interacts with positively charged triethylamine. In the crystal structure of **45**, Zn^II^ binds to the Cl^−^ and the oxidation state of the Zn metal ion remains unchanged, the chloride ion shows noncovalent interaction with [Et_3_NH]^+^. Most of the reported examples of chloride coordinated Zn^II^ porphyrinoids either fall into the class of 16 π electron macrocycles or N‐substituted porphyrins.^27^ In the case of N‐substituted porphyrins, the negative charge of the chloride is counter‐balanced by the positive charge on the tertiary core‐N‐atom whereas in present case charge is counter‐balanced by [Et_3_NH]^+^ and the porphyrin core remains intact making this an unique example.

Structure elucidation of BCP‐porphyrin **45** revealed the nearly coplanar nature of the BCP‐appended arm with respect to the porphyrin plane. In contrast, BCP‐porphyrin **46**, which has a larger distance between the BCP and porphyrin moieties, showed orthogonal rotation of the phenyl rings with respect to the porphyrin plane. Hence, **45** and its analogues show more promise towards the synthesis of cubane/BCP‐linked porphyrin systems for electron/energy studies owing to their extended conjugation.

## Conclusion

We have designed, synthesized, and characterized bridgehead substituted bicyclo[1.1.1]pentane and cubane derivatives via amide coupling reactions. This work demonstrates a broad substrate scope with over 35 new derivatives of cubane/BCP that were synthesized in moderate to good yields. The single crystal X‐ray structures of small rigid linker motifs (**13**, **33**, **35**, and **38**) revealed supramolecular 3D networks with combined and repetitive inter‐ and intramolecular H‐bonding interactions. Significantly, the crystal structure of cubane **13** showed an unusual C=O2⋅⋅⋅I1 interaction along with the usual N1⋅⋅⋅O1=C interaction to result in a 3D cage‐like structure. The formation of 3D supramolecular network dependent on the structurally pre‐organized BCP/cubane scaffold in association with the semi‐rigid amide moieties.

The crystal structure of porphyrin **45** illustrates a unique example of porphyrin based ion‐pair complex. Additionally, porphyrin **46** exhibits non‐covalent D⋅⋅⋅A interactions between the acceptor Zn^II^ metal of the porphyrin and the donor oxygen atom of the carbonyl group in the amide moiety in solid‐state. A follow‐up study on selective detection of small molecular motifs via this type of arrays is underway and will be reported separately.

## Experimental Section

General information, instrumentation, synthesis of precursors, crystallographic studies, and complete synthetic details of all synthesized compounds are given in Supporting Information.

### General procedures

**General procedure 1 for amide condensation reactions**: 4‐(Methoxycarbonyl)cubane‐1‐carboxylic acid (**1**) (1.0 equiv) or cubane‐1,4‐dicarboxylic acid (**2**) (1.0 equiv) was placed in oven‐dried microwave vial and heated under vacuum. The reaction flask was purged with argon, anhydrous DMF (0.25 mL) was added and the reaction mixture was heated slightly to dissolve the cubane. HATU (1.3/2.6 equiv), HOAt (1.3/2.6 equiv) and DIPEA (4.0/8.0 equiv) were then added and the reaction mixture was left to stir at rt. for 30 minutes under argon. The amine (2.0 equiv) was added under an argon flow to the flask alongside additional anhydrous DMF (0.25 mL). The reaction mixture was stirred at RT for a further 24 h and then diluted with H_2_O.

**General procedure 2 for amide condensation reactions**: In an oven‐dried microwave vial, a drop of DMF was added to the solution of 3‐(methoxycarbonyl)bicyclo[1.1.1]pentane‐1‐carboxylic acid (1.0 equiv) (**30**) or bicyclo[1.1.1]pentane‐1,3‐dicarboxylic acid (1.0 equiv) (**31**) in CH_2_Cl_2_. Oxalyl chloride (1.2 equiv/2.2 equiv) was added dropwise to above solution at 0 °C. The reaction mixture was warmed to 40 °C and stirred for 2 h under inert atmosphere. The reaction mixture was cooled to 0 °C. NEt_3_ (3.0/6.0 equiv) followed by substituted aniline (1.1/2.0 equiv) were added slowly to the reaction mixture. The reaction vial was warmed to 40 °C and stirred for 2 h under argon. The solvent was evaporated in vacuo, crude reaction mixture was recrystallized from CH_2_Cl_2_/hexane. The desired product was separated as white crystalline material.

**General procedure 3 to synthesize** ***meso*** **‐ethynylamine substituted porphyrins 9**–**12**: Iodo porphyrin (1.0 equiv) was placed in an oven‐dried Schlenk flask and heated under vacuum. The reaction flask was purged with argon and a mixture of THF/NEt_3_ (3:1) was added. Argon was bubbled through the solution for 15 min then ethynylaniline (5.0 equiv), PdCl_2_(PPh_3_)_2_ (0.15 equiv) and CuI (0.3 equiv) were added. The reaction mixture was heated to 70 °C and allowed to stir for 4 h. The reaction mixture was diluted with CH_2_Cl_2_ (10 mL) followed by removal of solvents in vacuo. Crude reaction mixture was purified by silica gel column chromatography.

**General procedure 4 for Sonogashira cross‐coupling using** ***meso*** **‐ethynyl porphyrin**: An oven‐dried Schlenk tube charged with ethynyl porphyrin (1.0 equiv) and iodo‐substituted cubane or BCP was heated under vacuum. THF (5 mL) followed by NEt_3_ (2.5 mL) were added to reaction vessel. Argon was bubbled through the solution for 10–15 min. and PdCl_2_(PPh_3_)_2_ (0.2 equiv) and CuI (0.3 equiv) were added. The resulting reaction mixture was heated at 40 °C and progress of the reaction was monitored by TLC. Reaction mixture was filtered through a Celite pad. Solvent was evaporated in vacuo, crude reaction mixture was purified by silica gel column chromatography.

**General procedure 5 for Suzuki cross‐coupling**: To an oven‐dried Schlenk tube charged with porphyrin (2.1 equiv), BCP linker (1.0 equiv) and K_3_PO_4_ (10.0 equiv) anhydrous DMF (5 mL) was added under inert atmosphere. The above solution was purged with argon for further 15 min followed by addition of 0.2 equiv of Pd(PPh_3_)_4_. The reaction mixture was heated to 100 °C and allowed to stir for 18 h. The solvent was removed in vacuo, crude reaction mixture was dissolved in CH_2_Cl_2_ washed with NaHCO_3_ followed by brine. Organic layer was extracted with CH_2_Cl_2_. Extracted organic phases were combined and solvent was evaporated. The resulting crude reaction mixture was purified by silica gel column chromatography.

### Synthesis of cubane linkers 13–18

**Methyl‐4‐((4′‐iodophenyl)carbamoyl)cubane‐1‐carboxylate (13)**: Synthesized via General Procedure 1 from 4‐(methoxycarbonyl)cubane‐1‐carboxylic acid (50 mg, 240 μmol), 4‐iodoaniline (160 mg, 730 μmol), HATU (119 mg, 310 μmol), HOAt (42.5 mg, 310 μmol) and DIPEA (17 μL) in anhydrous DMF (0.5 mL). The product was extracted with a mixture of CH_2_Cl_2_/MeOH (×3), dried over MgSO_4_ and the solvent removed under reduced pressure, the crystals were washed with CH_2_Cl_2_ to remove any remaining aniline and dried under reduced pressure. The product was obtained as white crystals. Yield: 120 mg, 61 %; m.p.: 240–245 °C; *R*_f_=0.35 (SiO_2_, EtOAc/hexane, 2:3, v/v); ^1^H NMR (600 MHz, [D_6_]DMSO): *δ*=9.77 (s, 1 H), 7.63 (d, *J=*8.6 Hz, 2 H), 7.50 (d, *J=*8.6 Hz, 2 H), 4.29–4.22 (m, 3 H), 4.18–4.15 (m, 3 H), 3.64 ppm (s, 3 H); ^13^C NMR (151 MHz, [D_6_]DMSO): *δ*=171.3, 169.5, 138.8, 137.2, 121.8, 86.8, 57.8, 54.9, 51.3, 46.5, 46.0 ppm; IR (neat): $\tilde{\nu }$ =1720 (m), 1644 (m), 1581 (m), 1512 (m), 1390 (m), 1322 (m), 1219 (w), 1169 (w), 1088 (m), 824 (s), 792 (s), 710 (m), 598 cm^−1^ (m); HRMS (APCI): *m*/*z*: calcd for C_17_H_13_INO_3_ [*M*−H]^−^ 405.994565, found 405.993716.

**Methyl‐4‐((4′‐ethynylphenyl)carbamoyl)cubane‐1‐carboxylate (14)**: Synthesized via General Procedure 1 from 4‐(methoxycarbonyl)cubane‐1‐carboxylic acid (15 mg, 70 μmol), HATU (43.5 mg, 114 μmol), HOAt (15.5 mg, 114 μmol), DIPEA (61 μL), 4‐ethynylaniline (25 mg, 210 μmol) in anhydrous DMF (0.5 mL). The product was extracted with a mixture of CH_2_Cl_2_/MeOH (×3), dried over MgSO_4_ and the solvent removed under reduced pressure, the crystals were washed with CH_2_Cl_2_ to remove any remaining aniline and dried under reduced pressure to afford white crystals. Yield: 8 mg, 37 %; m.p.: 243–247 °C; *R*_f_=0.67 (SiO_2_, EtOAc/hexane, 1:1, v/v); ^1^H NMR (600 MHz, [D_6_]DMSO): *δ*=9.86 (s, 1 H), 7.69 (d, *J=*8.5 Hz, 2 H), 7.41 (d, *J=*8.5 Hz, 2 H), 4.26 (t, *J=*4.6 Hz, 3 H), 4.17 (t, *J=*4.7 Hz, 3 H), 4.07 (s, 1 H), 3.64 ppm (s, 3 H); ^13^C NMR (151 MHz, [D_6_]DMSO): *δ*=171.2, 169.6, 139.6, 132.2, 119.3, 116.1, 83.6, 79.8, 57.8, 54.8, 51.3, 46.6, 46.0 ppm; IR (neat): $\tilde{\nu }$ =3252 (m), 2993 (w), 2950 (w), 1721 (s), 1645 (s), 1585 (s), 1509 (s), 1401 (m), 1336 (s), 1288 (m), 1221 (m), 1090 (s), 931 (w), 833 (s), 721 (s), 671 cm^−1^ (m); HRMS (APCI): *m*/*z*: calcd for C_19_H_16_NO_3_ [*M*+H]^+^ 306.112470; found 306.113336.

***N*** ^**1**^,***N*** ^**4**^ **‐Bis(4′‐iodophenyl)cubane‐1,4‐dicarboxamide (15)**: Synthesized via General Procedure 1 from cubane‐1,4‐dicarboxylic acid (46 mg, 240 μmol), 4‐iodoaniline (263 mg, 1.2 mmol), HATU (119 mg, 310 μmol), HOAt (42.5 mg, 310 μmol) and DIPEA (17 μL) in anhydrous DMF (0.5 mL). The reaction mixture was diluted with H_2_O and CH_2_Cl_2_ causing white crystals to crash out of the solution. The product was collected by vacuum filtration, washed with CH_2_Cl_2_ to remove unreacted aniline, and dried under reduced pressure to obtain white crystals. Yield: 55 mg, 39 %; m.p.: 257–259 °C; *R*_f_=0.37 (SiO_2_, hexane/EtOAc, 1:3, v/v); ^1^H NMR (400 MHz, [D_6_]DMSO): *δ*=9.77 (s, 2 H), 7.64 (d, *J=*8.6 Hz, 4 H), 7.52 (d, *J=*8.6 Hz, 4 H), 4.24 ppm (s, 6 H); ^13^C NMR (151 MHz, [D_6_]DMSO) : *δ*=169.9, 138.9, 137.2, 121.8, 86.7, 57.6, 46.3 ppm; IR (neat): $\tilde{\nu }$ =3342 (w), 2985 (w), 1738 (w), 1645 (s), 1588 (m), 1505 (s), 1390 (s), 1324 (m), 1289 (w), 1243 (m), 1217 (w), 1061 (w), 1007 (m), 955 (w), 809 (s), 795 (m), 669 (m), 610 cm^−1^ (w); HRMS (APCI): *m*/*z*: calcd for C_22_H_15_I_2_N_2_O_2_ [*M*−H]^−^ 592.922847; found 592.922494.

***N*** ^**1**^,***N*** ^**4**^ **‐Bis(3′‐iodophenyl)cubane‐1,4‐dicarboxamide (16)**: Synthesized via General Procedure 2 from cubane‐1,4‐dicarboxylic acid (46 mg, 240 μmol), Oxalyl chloride (45 μL, 530 μmol), NEt_3_ (0.13 mL, 960 μmol) and 3‐iodoaniline (64 μL, 530 μmol) in DMF (0.25 mL) and CH_2_Cl_2_ (1.5 mL). Solvents were evaporated in vacuo and resulting solid was washed with CH_2_Cl_2_ to give the product as white crystals. Yield: 44 mg, 31 %; m.p.: 243–248 °C; *R*_f_=0.37 (SiO_2_, EtOAc/hexane 3:1, v/v); ^1^H NMR (600 MHz, [D_6_]DMSO): *δ*=9.75 (s, 2 H), 8.13 (s, 2 H), 7.70 (d, *J=*8.2 Hz, 2 H), 7.41 (d, *J=*8.2 Hz, 2 H), 7.12 (t, *J=*8.2 Hz, 2 H), 4.25 ppm (s, 6 H); ^13^C NMR (151 MHz, [D_6_]DMSO): *δ*=169.9, 140.5, 131.7, 130.7, 127.7, 118.7, 94.4, 57.5, 46.3 ppm; IR (neat): $\tilde{\nu }$ =3200 (w), 2996 (w), 1738 (w), 1642 (s), 1580 (s), 1537 (m), 1474 (s), 1406 (m), 1333 (m), 1287 (w), 1242 (w), 1197 (w), 1092 (w), 996 (w), 947 (w), 901 (w), 865 (w), 845 (w), 772 (s), 721 (w), 681 (m), 657 (w), 615 (w), 572 cm^−1^ (w); HRMS (APCI): *m*/*z*: calcd for C_22_H_17_I_2_N_2_O_2_ [*M*+H]^+^ 594.937400; found 594.937107.

***N*** ^**1**^,***N*** ^**4**^ **‐Bis(4′‐ethynylphenyl)cubane‐1,4‐dicarboxamide (17)**: Synthesized via General Procedure 1 from cubane‐1,4‐dicarboxylic acid (30 mg, 157 μmol), HATU (156 mg, 410 μmol), HOAt (56 mg, 410 μmol), DIPEA (219 μL) and 4‐ethynylaniline (55 mg, 470 μmol) in DMF (0.5 mL). The reaction mixture was diluted with CH_2_Cl_2_ causing the product to crash out of the solution. The product was collected by vacuum filtration, washed with CH_2_Cl_2_ to remove any leftover aniline, and dried under reduced pressure to obtain white crystals. Yield: 39 mg, 67 %; m.p.: 247–252 °C; *R*_f_=0.74 (SiO_2_, EtOAc/hexane, 3:1, v/v); ^1^H NMR (600 MHz, [D_6_]DMSO): *δ*=9.84 (s, 2 H), 7.71 (d, *J=*8.5 Hz, 4 H), 7.42 (d, *J=*8.5 Hz, 4 H), 4.26 (s, 6 H), 4.08 ppm (s, 2 H); ^13^C NMR (151 MHz, [D_6_]DMSO): *δ*=169.9, 139.6, 132.2, 119.3, 116.1, 83.6, 79.8, 57.5, 46.3 ppm; IR (neat): $\tilde{\nu }$ =3283 (m), 3218 (w), 3086 (w), 3001 (w), 1650 (s), 1590 (s), 1510 (s), 1404 (s), 1338 (s), 1290 (m), 1252 (s), 1090 (m), 944 (m), 877 (m), 840 (s), 770 (m), 667 (m), 619 cm^−1^ (s); HRMS (APCI): *m*/*z*: calcd for C_26_H_19_N_2_O_2_ [*M*+H]^+^: 391.144104, found 391.143171.

***N*** ^**1**^,***N*** ^**4**^ **‐Bis(3′‐ethynylphenyl)cubane‐1,4‐dicarboxamide (18)**: Synthesized via General Procedure 1 from cubane‐1,4‐dicarboxylic acid (15 mg, 78 μmol), HATU (77 mg, 200 μmol), HOAt (27 mg, 200 μmol), DIPEA (108 μL) and 3‐ethynylaniline (27 μL, 234 μmol) in anhydrous DMF (1 mL). The reaction mixture was diluted with H_2_O and CH_2_Cl_2_ causing white crystals to crash out of the solution. The product was collected by vacuum filtration, washed with CH_2_Cl_2_ to remove any leftover aniline, and dried under reduced pressure to obtain white crystals. Yield: 36 mg, 50 %; m.p.: 297–302 °C (charred); *R*_f_=0.69 (SiO_2_, EtOAc/hexane, 3:1, v/v); ^1^H NMR (600 MHz, [D_6_]DMSO): *δ*=9.76 (s, 2 H), 7.86 (s, 2 H), 7.69 (d, *J=*7.9 Hz, 2 H), 7.33 (t, *J=*7.9 Hz, 2 H), 7.16 (d, *J=*7.9 Hz, 2 H), 4.26 (s, 6 H), 4.17 ppm (s, 2 H); ^13^C NMR (151 MHz, [D_6_]DMSO): *δ*=169.9, 139.3, 129.1, 126.4, 122.4, 121.8, 120.2, 83.4, 80.4, 57.5, 46.3, 45.9 ppm; IR (neat): $\tilde{\nu }$ =3312 (w), 3218 (w), 3048 (w), 2999 (w), 1649 (m), 1603 (m), 1524(m), 1480 (s), 1406 (s), 1331 (s), 1298 (m), 1225 (m), 1086 (w), 950 (m), 859 (m), 782 (s), 683 (s), 599 cm^−1^ (s); HRMS (APCI): *m*/*z*: calcd for C_26_H_19_N_2_O_2_ [*M*+H]^+^ 391.144104; found 391.143981.

### Synthesis of cubane porphyrin dimers 25–29

***N*** ^**1**^,***N*** ^**4**^ **‐Bis[4′‐{(10′′,15′′,20′′‐triphenylporphyrinato)zinc(II)‐5′′‐yl}‐phenyl]cubane‐1,4‐dicarboxamides (25)**: Synthesized via General Procedure 1 from cubane‐1,4‐dicarboxylic acid (15 mg, 80 μmol), [5‐(4′‐aminophenyl)‐10,15,20‐triphenylporphyrinato]zinc(II) (**8**) (110 mg, 160 μmol), HATU (79 mg, 210 μmol), HOAt (28.5 mg, 210 μmol) and DIPEA (111 μL) in anhydrous DMF (0.5 mL). H_2_O was added and the product was extracted with CH_2_Cl_2_/MeOH (×3), washed with H_2_O (×4), dried over MgSO_4_ and the solvent removed under reduced pressure. The crude material was purified by column chromatography (SiO_2_, CH_2_Cl_2_/(CH_3_)_2_CO, 100:0 to 98.8:0.02). The product was obtained as purple crystals. Yield: 35 mg, 28 %; m.p.: >350 °C; *R*_f_=0.71 (SiO_2_, CH_2_Cl_2_/(CH_3_)_2_CO, 20:1, v/v); ^1^H NMR (600 MHz, CDCl_3_/[D_8_]THF): *δ*=8.90 (dd, *J=*14.4, 4.5 Hz, 8 H), 8.86 (s, 8 H), 8.21–8.19 (m, 16 H), 7.99 (s, 2 H), 7.98 (s, 4 H), 7.75–7.70 (m, 18 H), 4.58 ppm (s, 6 H); ^13^C NMR (151 MHz, CDCl_3_/[D_8_]THF): *δ*=169.9, 150.2, 150.1, 143.4, 139.6, 137.2, 135.2, 135.2, 134.6, 134.6, 131.7, 127.3, 126.5, 120.8, 120.1, 117.8, 60.5, 58.9, 47.2 ppm; IR (neat): $\tilde{\nu }$ =1736 (w), 1660 (w), 1594 (w), 1486 (m), 1439 (w), 1398 (w), 1338 (w), 1235 (w), 1203 (w), 1174 (w), 1067 (w), 993 (s), 796 (s), 749 (m), 718 (m), 701 (s), 569 cm^−1^ (w); UV/Vis (CHCl_3_): *λ*_max_ (log*ϵ*) = 422 (6.19), 549 (4.80), 589 nm (4.15); HRMS (MALDI‐TOF): *m*/*z*: calcd for C_98_H_62_N_10_O_2_Zn_2_ [*M*]^+^ 1538.3640; found 1538.3662.

***N*** ^**1**^,***N*** ^**4**^ **‐Bis[4′‐{(10′′,15′′,20′′‐triphenylporphyrin)‐5′′‐yl}‐phenyl]cubane‐1,4‐dicarboxamides (26)**: Synthesized via General Procedure 1 from cubane **2** (50 mg, 69 μmol), 5‐(4′‐aminophenyl)‐10,15,20‐triphenylporphyrin (**7**) (129 mg, 210 μmol), HATU (34.1 mg, 90 μmol), HOAt (12.2 mg, 90 μmol) and DIPEA (48 μL) in anhydrous DMF (0.5 mL). H_2_O was added and the product was extracted with CH_2_Cl_2_ (×3), washed with H_2_O (×4), dried over MgSO_4_ and the solvent removed under reduced pressure. The crude material was purified by column chromatography (SiO_2_, CH_2_Cl_2_/(CH_3_)_2_CO, 100:0 to 98.8:0.02). The product was obtained as purple crystals. Yield: 55 mg, 56 %; m.p.: >350 °C; *R*_f_=0.52 (SiO_2_, CH_2_Cl_2_/EtOAc, 40:1, v/v); ^1^H NMR (600 MHz, CDCl_3_): *δ*=8.87 (d, *J=*3.4 Hz, 8 H), 8.85 (s, 8 H), 8.23–8.22 (m, 16 H), 8.02 (d, *J=*7.8 Hz, 2 H), 7.78–7.75 (m, 20 H), 7.58 (s, 2 H), 4.63 (s, 6 H), −2.76 ppm (s, 4 H); ^13^C NMR (151 MHz, CDCl_3_): *δ*=189.7, 142.3, 135.4, 134.7, 127.9, 126.8, 120.4, 118.1, 47.3 ppm; IR (neat): $\tilde{\nu }$ =1674 (w), 1584 (w), 1494 (w), 1397 (w), 1317 (w), 1190 (w), 965 (m), 798 (s), 753 (m), 722 (m), 701 (s); UV/Vis (CH_2_Cl_2_): *λ*_max_ (log*ϵ*) = 422 (6.01), 518 (4.65), 554 (4.40), 593 (4.29), 649 nm cm^−1^ (4.21); HRMS (MALDI‐TOF): *m*/*z*: calcd for C_98_H_67_N_10_O_2_ [*M*+H]^+^: 1415.5448, found 1415.5514.

***N*** ^**1**^ **‐[4′‐{(10′′,20′′,15′′‐Triphenylporphyrinato)zinc(II)‐5′‐yl}phenyl]‐*N*** ^**4**^ **‐[4′‐{(10′′,20′′,15′′‐triphenylporphyrin)‐5′‐yl}phenyl]cubane‐1,4‐dicarboxamide (27)**: Synthesized via General Procedure 1 from compound **22** (30 mg, 37 μmol), 5‐(4‐aminophenyl)‐10,15,20‐triphenylporphyrinato zinc(II) (8) (31 mg, 45 μmol), HATU (43.5 mg, 114 μmol), HOAt (15.5 mg, 114 μmol) and DIPEA (61 μL) in anhydrous DMF (0.5 mL). The reaction mixture washed with brine and the products were extracted with a mixture of CH_2_Cl_2_/THF (×3), dried over MgSO_4_ and the solvent removed under reduced pressure. The crude material was purified by column chromatography (SiO_2_, CH_2_Cl_2_/(CH_3_)_2_CO, 100:0 to 98.8:0.02). The product was recrystallized from CH_2_Cl_2_/CH_3_OH and obtained as purple crystals. Yield: 28 mg, 51 %; m.p.: >350 °C; *R*_f_=0.64 (SiO_2_, (CH_3_)_2_CO/CH_2_Cl_2_, 1:20, v/v); ^1^H NMR (600 MHz, [D_6_]DMSO): *δ*=10.21 (s, 1 H), 10.16 (s, 1 H), 8.92 (s, 2 H), 8.86–8.84 (m, 8 H), 8.80–8.78 (m, 6 H), 8.25–8.15 (m, 20 H), 7.83 (dd, *J=*25.5, 5.4 Hz, 18 H), 4.52 (s, 6 H), −2.88 ppm (s, 2 H); ^13^C NMR (151 MHz, [D_6_]DMSO): *δ*=170.1, 149.5, 149.3, 149.2, 142.8, 141.2, 134.6, 134.4, 134.2, 134.2, 131.5, 128.1, 127.4, 127.0, 126.6, 120.3, 120.0, 117.9, 117.7, 46.6 ppm; IR (neat): $\tilde{\nu }$ =1658 (w), 1596 (w), 1489 (m), 1400 (w), 1320 (w), 1179 (w), 1071 (w), 1002 (m), 994 (m), 796 (s), 718 (m), 700 (s), 563 cm^−1^ (w); UV/Vis (CHCl_3_): *λ*_max_ (log*ϵ*)=422 (5.79), 451 (5.66), 550 (4.43), 671 nm (4.81); HRMS (MALDI‐TOF): *m*/*z*: calcd for C_98_H_64_N_10_O_2_Zn [*M*]^+^ 1476.4505; found 1476.4495.

***N*** ^**1**^,***N*** ^**4**^ **‐Bis[3′‐{(10′′,20′′‐bis(4′‐methylphenyl)‐15′′‐phenylporphyrinato)zinc(II)‐5′′‐yl}phenylacetylene]cubane‐1,4‐dicarboxamide (28)**: Synthesized via General Procedure 1 from cubane‐1,4‐dicarboxylic acid (10.5 mg, 55 μmol) (2), porphyrin 11 (85 mg, 110 μmol), HATU (54.4 mg, 143 μmol), HOAt (19.5 mg, 143 μmol) and DIPEA (76 μL) in anhydrous DMF (0.5 mL). The reaction mixture was then washed with brine and the products were extracted with a mixture of CH_2_Cl_2_/MeOH (×3), dried over MgSO_4_ and the solvent removed under reduced pressure. The crude material was purified by column chromatography (SiO_2_, CH_2_Cl_2_:(CH_3_)_2_CO,100:0 to 98.8:0.02). The product was recrystallized from CH_2_Cl_2_/CH_3_OH and obtained as purple crystals. Yield=32 mg, 36 %; m.p.: >350 °C; *R*_f_=0.33 (SiO_2_, CH_2_Cl_2_/(CH_3_)_2_CO, 20:1, v/v); ^1^H NMR (600 MHz, CDCl_3_): *δ*=9.77 (d, *J=*4.4 Hz, 4 H), 8.97 (d, *J=*4.4 Hz, 4 H), 8.80 (dd, *J=*21.3, 4.5 Hz, 8 H), 8.19 (s, 1 H), 8.16 (d, *J=*4.4 Hz, 4 H), 8.08 (d, *J=*7.2 Hz, 8 H), 7.80–7.77 (m, 3 H), 7.74–7.69 (m, 8 H), 7.59 (s, 1 H), 7.55 (d, *J=*7.2 Hz, 8 H), 7.52–7.49 (m, 3 H), 4.50 (s, 6 H), 2.71 ppm (s, 12 H); ^13^C NMR (151 MHz, CDCl_3_): *δ*=169.7, 152.3, 150.7, 150.0, 149.9, 143.2, 140.1, 138.1, 137.1, 134.6, 134.5, 132.8, 131.9, 131.7, 130.5, 129.5, 127.4, 127.3, 126.5, 125.4, 122.7, 122.5, 121.9, 119.7, 98.8, 95.2, 93.9, 67.7, 58.8, 47.1, 21.7 ppm; UV/Vis (THF): *λ*_max_ (log*ϵ*)=443 (6.13), 557 (4.65), 624 nm (4.94); IR (neat): $\tilde{\nu }$ =1650 (w), 1598 (w), 1519 (w), 1483 (m), 1401 (w), 1339 (m), 1305 (w), 1207 (m), 1180 (w), 1064 (w), 996 (s), 845 (w), 792 (s), 714 (m), 701 (m), 682 (m), 569 cm^−1^ (m); HRMS (MALDI‐TOF): *m*/*z*: calcd for C_106_H_70_N_10_O_2_Zn_2_ [*M*]^+^ 1642.4266; found 1642.4296.

***N*** ^**1**^,***N*** ^**4**^ **‐Bis[2′‐{(10′′,20′′‐bis(4′‐methylphenyl)‐15′′‐phenylporphyrinato)zinc(II)‐5′′‐yl}phenylacetylene]cubane‐1,4‐dicarboxamide (29)**: Synthesized via General Procedure 1 from cubane‐1,4‐dicarboxylic acid (8.4 mg, 44 μmol) (2), porphyrin 12 (65 mg, 87 μmol), HATU (43.5 mg, 114 μmol), HOAt (15.5 mg, 114 μmol) and DIPEA (61 μL) in anhydrous DMF (0.5 mL). The reaction mixture was then washed with brine and the products were extracted with a mixture of CH_2_Cl_2_/MeOH (×3), dried over MgSO_4_ and the solvent removed under reduced pressure. The crude material was purified by column chromatography (SiO_2_, CH_2_Cl_2_:(CH_3_)_2_CO, 100:0 to 98.8:0.02). The product was recrystallized from CH_2_Cl_2_/CH_3_OH and obtained as purple crystals. Yield: 36 mg, 50 %; m.p.: >350 °C; *R*_f_=0.75 (SiO_2_, CH_2_Cl_2_/(CH_3_)_2_CO, 40:1, v/v); ^1^H NMR (600 MHz, [D_8_]THF): *δ*=9.61 (d, *J=*4.4 Hz, 4 H), 8.91 (d, *J=*4.4 Hz, 4 H), 8.75 (dd, *J=*11.1, 4.4 Hz, 10 H), 8.43 (d, *J=*8.1 Hz, 2 H), 8.15 (d, *J=*6.4 Hz, 4 H), 8.03 (d, *J=*7.6 Hz, 8 H), 7.95 (d, *J=*6.8 Hz, 2 H), 7.74 (dt, *J=*13.9, 7.0 Hz, 6 H), 7.51 (d, *J=*7.5 Hz, 8 H), 7.37 (t, *J=*6.8 Hz, 2 H), 7.22 (t, *J=*6.8 Hz, 2 H), 3.81 (s, 6 H), 2.60 ppm (s, 12 H); ^13^C NMR (151 MHz, [D_8_]THF): *δ*=170.0, 153.0, 151.8, 150.9, 146.7, 144.3, 141.1, 138.1, 135.4, 135.3, 133.7, 132.6, 132.4, 132.3, 130.8, 130.3, 128.2, 127.4, 124.0, 123.2, 120.7, 114.8, 101.1, 98.11, 91.6, 86.1, 59.7, 47.8, 21.6 ppm; IR (neat): $\tilde{\nu }$ =3380 (w), 2920 (w), 2187 (w), 1652 (m), 1574 (m), 1510 (m), 1313 (m), 1206 (m), 1063 (m), 995 (s), 944 (m), 794 (s), 755 (s), 715 (m), 596 cm^−1^ (m); UV/Vis (THF): *λ*_max_ (log*ϵ*)=439 (5.92), 574 (4.53), 622 nm (4.72); HRMS (MALDI‐TOF): *m*/*z*: calcd for C_106_H_70_N_10_O_2_Zn_2_ [*M*]^+^ 1642.4266; found 1642.4304.

### Synthesis of BCP linkers 32–39

**Methyl‐3‐((4′‐iodophenyl)carbamoyl)bicyclo[1.1.1]pentane‐1‐carboxylate (32)**: Synthesized according to General Procedure 2 using compounds 3‐(methoxycarbonyl)bicyclo[1.1.1]pentane‐1‐carboxylic acid (**30**) (52.0 mg, 0.306 mmol), oxalyl chloride (32.0 μL, 0.378 mmol), NEt_3_ (130 μL, 0.934 mmol), 4‐iodoaniline (77.8 mg, 0.355 mmol). The solvent was removed in vacuo, crude product was recrystallized from CH_2_Cl_2_/hexane to afford titled compound as off‐white powder. Yield: 65 mg, 57 %; m.p.: 206 °C; *R*_f_=0.75 (SiO_2_, hexane/EtOAc, 7:3, v/v); ^1^H NMR (400 MHz; CDCl_3_): *δ*=7.60 (d, *J=*8.8 Hz, 2 H), 7.34 (br s, 1 H), 7.30 (d, *J=*8.8 Hz, 2 H), 3.70 (s, 3 H), 2.36 ppm (s, 6 H); ^13^C NMR (100 MHz; CDCl_3_): *δ*=169.5, 167.4, 137.8, 136.8, 121.6, 87.9, 52.4, 51.9, 40.0, 36.5 ppm; IR(neat): $\tilde{\nu }$ =3279 (w), 1723 (m), 1653 (s), 1593 (m), 1526 (s), 1392 (m), 1310 (s), 1214 (s), 1044 (m), 812 (s), 785 (s), 691 cm^−1^ (s); HRMS(APCI): *m*/*z*: calcd for C_14_H_15_INO_3_^+^ [*M*+H]^+^, 372.0091; found 372.0091.

**Methyl‐3‐((4′‐ethynylphenyl)carbamoyl)bicyclo[1.1.1]pentane‐1‐carboxylate (33)**: Synthesized according to General Procedure 2 using compounds 3‐(methoxycarbonyl)bicyclo[1.1.1]pentane‐1‐carboxylic acid (**30**) (80.0 mg, 0.47 mmol), oxalyl chloride (47.0 μL, 0.56 mmol), NEt_3_ (200 μL, 1.4 mmol), 4‐ethynylaniline (60 mg, 0.47 mmol). The solvent was removed in vacuo, crude product was recrystallized from CH_2_Cl_2_/hexane to afford titled compound as an off‐white powder. Yield: 77 mg, 61 %; m.p.: 216 °C; *R*_f_=0.25 (SiO_2_, hexane/EtOAc, 7:3, v/v); ^1^H NMR (400 MHz; CDCl_3_): *δ*=7.49 (d, *J=*8.0 Hz, 2 H), 7.43 (d, *J=*8.0 Hz, 2 H), 7.23 (brs, 1 H), 3.69 (s, 3 H), 3.03 (s, 1 H), 2.36 ppm (s, 6 H); ^13^C NMR (100 MHz; CDCl_3_): *δ*=169.5, 167.1, 137.6, 133.0, 119.3, 118.1, 83.2, 52.4, 52.0, 40.1, 36.7 ppm; IR(neat): $\tilde{\nu }$ =3281 (w), 3242 (m), 1718 (m), 1651 (m), 1587 (m), 1506 (m), 1312 (s), 1217 (s), 1043 (w), 943 (s), 826 (s), 721 (m), 677 cm^−1^ (m); HRMS(APCI): *m*/*z*: calcd for (C_16_H_14_NO_3_ [*M*−H]^−^) 268.0974; found 268.0984; calcd for (C_16_H_15_ClNO_3_ [*M*+Cl]^−^) 304.0746; found 304.0754.

**Methyl‐3‐((3′‐iodophenyl)carbamoyl)bicyclo[1.1.1]pentane‐1‐carboxylate (34)**: Synthesized according to General Procedure 2 using **30** (80.0 mg, 0.47 mmol), oxalyl chloride (47.0 μL, 0.56 mmol), NEt_3_ (200 μL, 1.4 mmol), 3‐iodoaniline (57.0 μL, 0.47 mmol). The solvent was removed in vacuo, crude product was recrystallized from CH_2_Cl_2_/hexane to afford titled compound as white powder. Yield: 90 mg, 52 %; m.p.: 198 °C; *R*_f_=0.35 (SiO_2_, hexane/EtOAc, 7:3, v/v); ^1^H NMR (400 MHz; CDCl_3_): *δ*=7.93 (t, *J=*1.7 Hz, 1 H), 7.56 (d, *J=*12 Hz, 1 H), 7.48 (d, *J=*7.9 Hz, 1 H), 7.14 (brs, 1 H), 7.07(t, *J=*8 Hz, 1 H), 3.74 (s, 3 H), 2.40 ppm (s, 6 H); ^13^C NMR (100 MHz; CDCl_3_): *δ*=169.4, 167.2, 138.4, 133.7, 130.5, 128.3, 118.8, 94.1, 52.4, 51.9, 39.9 ppm; IR(neat): $\tilde{\nu }$ =3276 (w), 1653 (s), 1525 (m), 1588 (s), 1525 (m), 1415 (s), 1302 (s), 1212 (s), 1043 (m), 883 (m), 775 (s), 653 cm^−1^ (s); HRMS(APCI): *m*/*z*: calcd for C_14_H_13_INO_3_ [*M*−H]^−^ 369.9940; found 369.9954.

**Methyl‐3‐((3′‐ethynylphenyl)carbamoyl)bicyclo[1.1.1]pentane‐1‐carboxylate (35)**: Synthesized according to General Procedure 2 using **30** (80.0 mg, 0.47 mmol), oxalyl chloride (47.0 μL, 0.56 mmol), NEt_3_ (200 μL, 1.4 mmol), 3‐ethynylaniline (58 μL, 0.47 mmol). The solvent was removed in vacuo, crude product was recrystallized from CH_2_Cl_2_/hexane to afford titled compound as white powder. Titled compound was obtained by recrystallization from CH_2_Cl_2_/hexane. Yield: 62 mg, 49 %; m.p.: 185 °C; *R*_f_=0.30 (SiO_2_, hexane/EtOAc 7:3, v/v); ^1^H NMR (400 MHz; [D_6_]DMSO): *δ*=9.73 (s, 1 H), 7.81 (t, *J=*1.7 Hz, 1 H), 7.65 (d, *J=*9.4 Hz, 1 H), 7.33 (t, *J=*7.9 Hz, 1 H), 7.17 (dt, *J=*7.8, 1.2 Hz, 1 H), 4.17 (s, 1 H), 3.64 (s, 3 H), 2.30 ppm (s, 6 H); ^13^C NMR (101 MHz; [D_6_]DMSO): *δ*=169.8, 167.9, 139.2, 129.5, 127.2, 123.1, 122.3, 120.8, 83.7, 81.0, 52.4, 52.0, 40.6, 40.4, 40.1, 39.9, 39.8, 39.6, 39.4, 36.8 ppm; IR(neat): $\tilde{\nu }$ =3374 (w), 3193 (w), 1713 (m), 1672 (m), 1603 (m), 1406 (m), 1307 (s), 1213 (s), 1054 (w), 862 (w), 791 (s), 686 (s), 630 cm^−1^ (s); HRMS(APCI): *m*/*z*: calcd for C_16_H_14_NO_3_ [*M*−H]^−^ 268.0974; found. 268.0985.

***N*** ^**1**^,***N*** ^**3**^ **‐Bis(4′‐iodophenyl)bicyclo[1.1.1]pentane‐1,3‐dicarboxamide (36)**: Synthesized according to General Procedure 2 using **31** (70 mg, 0.45 mmol), oxalyl chloride (85.0 μL, 0.99 mmol), NEt_3_ (0.37 mL, 2.7 mmol) and 4‐iodoaniline (207 mg, 0.98 mmol). The reaction solvent was removed in vacuo and the resulting residue was washed with CH_2_Cl_2_, and the insoluble material was collected to yield the desired product as a white solid. Yield: 180 mg, 72 %; m.p.: 301 °C; ^1^H NMR (400 MHz; [D_6_]DMSO): *δ*=9.72 (s, 2 H), 7.63 (d, *J=*8 Hz, 4 H), 7.49 (d, *J=*12 Hz, 4 H), 2.31 ppm (s, 6 H); ^13^C NMR (100 MHz; [D_6_]DMSO): *δ*=168.3, 138.9, 137.6, 122.4, 87.5, 52.1, 39.2 ppm; IR(neat): $\tilde{\nu }$ =3321 (w), 1666 (s), 1584 (w), 1502 (s), 1387 (m), 1307 (m), 1004 (m), 814 (s), 661 cm^−1^ (m); HRMS(APCI): *m*/*z*: calcd for C_19_H_17_I_2_N_2_O_2_ [*M*+H]^+^ 558.9374; found 558.9370.

***N*** ^**1**^,***N*** ^**3**^ **‐Bis(4′‐ethynylphenyl)bicyclo[1.1.1]pentane‐1,3‐dicarboxamide (37)**: Synthesized according to General Procedure 2 using **31** (50 mg, 0.32 mmol), oxalyl chloride (60.0 μL, 0.64 mmol), NEt_3_ (0.28 mL, 2.7 mmol) and 4‐ethynylaniline (78 mg, 0.64 mmol). Titled compound was obtained by recrystallization from CH_2_Cl_2_. Yield: 87 mg, 77 %; m.p.: 326 °C; ^1^H NMR (400 MHz; [D_6_]DMSO): *δ*=9.84 (s, 2 H), 7.70 (d, *J=*8.5 Hz, 4 H), 7.43 (d, *J=*8.5 Hz, 4 H), 4.10 (s, 2 H) 2.35 ppm (s, 6 H); ^13^C NMR (100 MHz; [D_6_]DMSO): *δ*=168.4, 139.7, 132.7, 120.1, 116.8, 83.9, 80.4, 52.3, 39.2 ppm; IR(neat): $\tilde{\nu }$ =3292 (w), 3269 (w), 1649 (s), 1589 (m), 1522 (m), 1504 (m), 1402 (w), 1324 (m), 1246 (w), 825 (s), 705 (w), 616 cm^−1^ (m); HRMS(APCI): *m*/*z*: calcd for C_23_H_19_N_2_O_2_ [*M*+H]^+^ 355.1441; found 355.1437.

***N*** ^**1**^,***N*** ^**3**^ **‐Bis(3′‐ethynylphenyl)bicyclo[1.1.1]pentane‐1,3‐dicarboxamide (38)**: Synthesized according to General Procedure 2 using **31** (50 mg, 0.320 mmol), oxalyl chloride (60.0 μL, 0.640 mmol), NEt_3_ (0.28 mL, 2.70 mmol) and 4‐ethynylaniline (78 mg, 0.64 mmol). Titled compound was obtained by recrystallization from CH_2_Cl_2_. Yield: 59 mg, 52 %; m.p.: 240 °C; ^1^H NMR (400 MHz; [D_6_]DMSO): *δ*=9.85 (s, 2 H), 7.84 (d, *J=*1.6 Hz, 2 H), 7.70 (d, *J=*9.5 Hz, 2 H), 7.30 (t, *J=*8.0 Hz, 2 H), 7.14 (d, *J=*9.8 Hz, 2 H), 4.15 (s, 2 H), 2.34 ppm (s, 6 H); ^13^C NMR (101 MHz; [D_6_]DMSO): *δ*=168.5, 139.1, 131.8, 120.2, 116.8, 83.8, 80.1, 52.4; IR (neat): $\tilde{\nu }$ =3286 (w), 1651 (s), 1583 (m), 1540 (m), 1426 (s), 1307 (w), 1317 (w), 1217 (w), 792 (s), 686 (m) 643 cm^−1^ (s); HRMS(APCI): *m*/*z*: calcd for C_23_H_19_N_2_O_2_ [*M*+H]^+^ 355.1441; found 355.1441.

***N*** ^**1**^,***N*** ^**3**^ **‐Bis(3′‐iodophenyl)bicyclo[1.1.1]pentane‐1,3‐dicarboxamide (39)**: Synthesized according to General Procedure 2 using **31** (50 mg, 0.320 mmol), oxalyl chloride (60.0 μL, 0.640 mmol), NEt_3_ (0.28 mL, 2.70 mmol) and 3‐iodoaniline (140 mg, 0.64 mmol). Titled compound was obtained by recrystallization from CH_2_Cl_2_. Yield: 68 %; m.p.: 320 °C; ^1^H NMR (400 MHz; [D_6_]DMSO): *δ*=9.77 (s, 2 H), 8.11 (t, *J=*1.8 Hz, 2 H), 7.79 (d, *J=*8.0 Hz, 2 H), 7.42 (d, *J=*8.0 Hz, 2 H), 7.12 (t, *J=*8.0 Hz, 2 H), 2.33 ppm (s, 6 H); ^13^C NMR (101 MHz; [D_6_]DMSO): *δ*=167.9, 140.0, 132.0, 130.6, 128.0, 119.1, 94.4, 51.8, 45.7 ppm; IR(neat): $\tilde{\nu }$ =3272 (w), 1652 (s), 1586 (m), 1522 (m), 1414 (m), 1310 (w), 1243 (w), 882 (w), 776 (s), 681 cm^−1^ (s); HRMS(APCI): *m*/*z*: calcd for C_19_H_15_I_2_N_2_O_2_ [*M*−H]^−^ 556.9223; found 556.9074.

### Synthesis of BCP porphyrin dimers 50–52

***N*** ^**1**^,***N*** ^**3**^ **‐Bis[4′‐{(10′′,15′′,20′′‐triphenylporphyrinato)zinc(II)‐5′′‐yl}‐phenyl]bicyclo[1.1.1]pentane‐1,3‐dicarboxamides (50)**: Synthesized according to General Procedure 5 using BCP **36** (30 mg, 0.028 mmol), [5‐(4′,4′,5′,5′‐tetramethyl‐1′,3′,2′‐dioxaborolan‐2′‐yl)‐10,20‐bis(4′‐methylphenyl)‐15‐phenylporphyrinato]zinc(II) (**41**) (40 mg, 0.055 mmol), K_3_PO_4_ (140 mg, 0.34 mmol) and Pd(PPh_3_)_4_ (13 mg, 0.0056 mmol). Crude reaction mixture was purified using silica gel column chromatography to yield desired compound. Yield: 22 mg, 52 %; m.p.: 207 °C; *R*_f_=0.8 (SiO_2_, CH_2_Cl_2_/EtOAc, 8:2, v/v); ^1^H NMR (400 MHz; CDCl_3_): *δ*=8.89 (d, *J=*4.6 Hz, 4 H), 8.85 (d, *J=*4.6 Hz, 4 H), 8.84 (s, 8 H), 8.19 (d, *J=*7.6 Hz, 16 H), 8.04 (d, *J=*8.1 Hz, 4 H), 7.73–769 (m, 20 H), 2.71 ppm (s, 6 H); ^13^C NMR (101 MHz; CDCl_3_): *δ*=169.6, 167.1, 150.4, 150.2, 149.8, 139.7, 137.1, 134.5, 134.4, 134.3, 132.2, 132.1, 131.9, 131.5, 129.8, 127.3, 126.5, 89.9, 89.7, 52.5, 52.0, 40.1, 21.5 ppm; IR (neat): $\tilde{\nu }$ =2921 (w), 1736 (w), 1666 (w), 1529 (w), 1483 (w), 1339 (w), 1298 (w), 1208 (m), 998 (s), 796 (s), 719 (m); UV/Vis (CHCl_3_): *λ*_max_ (log*ϵ*)=424 (6.80), 551 (5.41), 590 nm cm^−1^ (4.82); HRMS(MALDI‐TOF): *m*/*z*: calcd for C_95_H_62_N_10_O_2_Zn_2_ [*M*]^+^ 1502.3640; 1502.3638 found.

***N*** ^**1**^,***N*** ^**3**^ **‐Bis[4′‐{(10′′,20′′‐bis(4‐methylphenyl)‐15′′‐phenylporphyrin)‐5′′‐yl}‐phenyl]bicyclo[1.1.1]pentane‐1,3‐dicarboxamides (51)**: Synthesized according to General Procedure 5 using BCP **36** (23 mg, 0.042 mmol), **41** (58 mg, 0.083 mmol), K_3_PO_4_ (200 mg, 0.50 mmol), Pd(PPh_3_)_4_ (20 mg, 8.4 μmol). Reaction mixture was heated at 100 °C for 12 h. The crude reaction mixture was purified using silica gel column chromatography, desired compound was eluted via using CH_2_Cl_2_/(CH_3_)_2_CO, 8:2. Yield: 34 mg, 60 %; m.p.: 331 °C; *R*_f_=0.2 (SiO_2_, CH_2_Cl_2_/EtOAc, 8:2, v/v); ^1^H NMR (600 MHz, CDCl_3_): *δ*=8.92–8.85 (m, 16 H), 8.25 (t, *J=*7.6 Hz, 8 H), 8.11 (d, *J=*7.7 Hz, 8 H), 8.03 (d, *J=*7.9 Hz, 4 H), 7.81–7.76 (m, 6 H), 7.66 (s, 2 H), 7.59 (d, *J=*7.7 Hz, 8 H), 2.77 (s, 6 H), 2.74 (s, 12 H), −2.74 ppm (s, 4 H); ^13^C NMR (151 MHz, CDCl_3_): *δ*=167.4, 144.2, 142.2, 139.3, 139.2, 138.8, 137.4, 136.9, 135.2, 134.5, 134.5, 127.4, 126.6, 120.3, 120.1, 119.1, 118.1, 52.4, 42.0, 21.6 ppm; IR (neat): $\tilde{\nu }$ =2981 (w), 2832 (w), 1710 (m), 1595 (m), 1367 (w), 1219 (m), 1140 (m), 1093 (m), 1047 (m), 942 (m), 807 (s), 757 (s), 691 (m), 652 cm^−1^ (m); UV/Vis (CHCl_3_): *λ*_max_ (log*ϵ*)=423 (5.92), 519 (4.52), 555 (4.25), 594 nm (4.04), 651 nm (4.06); HRMS(MALDI‐TOF): *m*/*z*: calcd for C_99_H_75_N_10_O_2_ [*M*+H]^+^ 1434.5996; 1435.6091 found.

***N*** ^**1**^,***N*** ^**3**^ **‐Bis[3′‐{(10′′,15′′,20′′‐triphenylporphyrinato)zinc(II)‐5′′‐yl}‐phenyl]bicyclo[1.1.1]pentane‐1,3‐dicarboxamides (52)**: Synthesized according to General Procedure 5 using BCP **39** (19 mg, 0.033 mmol), **41** (50 mg, 0.066 mmol), K_3_PO_4_ (70 mg, 0.33 mmol) and Pd(PPh_3_)_4_ (0.0066 mmol, 7.6 mg). Reaction mixture was heated at 100 °C for 4 h. The crude reaction mixture was subjected to silica gel column chromatography and titled compound was eluted via CH_2_Cl_2_/EtOAc, 9:1. Yield: 37 mg, 73 %; m.p.: 220 °C; *R*_f_=0.35 (SiO_2_, CH_2_Cl_2_/EtOAc, 8:2, v/v); ^1^H NMR (400 MHz, CDCl_3_/CD_3_OD): *δ*=8.85–8.81 (m, 16 H), 8.32(brs, 2 H), 8.17 (d, *J=*4.3 Hz, 4 H), 8.03 (d, *J=*7.1 Hz, 10 H), 7.95 (d, *J=*7.6 Hz, 2 H), 7.71–7.62 (m, 8 H), 7.47 (d, *J=*7.2 Hz, 8 H), 2.65 (s, 12 H), 2.44 ppm (s, 6 H); ^13^C NMR (101 MHz, CDCl_3_/CD_3_OD): *δ*=150.1, 149.9, 149.7, 144.1, 140.3, 136.7, 134.4, 131.5, 131.4, 131.2, 127.0, 126.6, 126.2, 120.0, 52.1, 39.0, 21.3 ppm; IR (neat): $\tilde{\nu }$ =2920 (w), 1650 (w), 1603 (w), 1518 (w), 1477 (w), 1388(w), 1204 (w), 1065 (w), 992 (s), 794 (s), 721 (m), 693 cm^−1^ (m); UV/Vis (CHCl_3_): *λ*_max_ (log*ϵ*)=422 (6.80), 551 (5.55), 589 nm (5.08); HRMS(MALDI‐TOF): *m*/*z*: calcd for C_99_H_70_N_10_O_2_Zn_2_ [*M*]^+^ 1562.4770; 1562.4253 found.

## Conflict of interest

The authors declare no conflict of interest.

## Supporting information

As a service to our authors and readers, this journal provides supporting information supplied by the authors. Such materials are peer reviewed and may be re‐organized for online delivery, but are not copy‐edited or typeset. Technical support issues arising from supporting information (other than missing files) should be addressed to the authors.

Supplementary
