# Supplementary material for: Bridging and Conformational Control of Porphyrin Units through Non‐Traditional Rigid Scaffolds
Source: Chemistry. 2020 Jan 21;26(11):2405–16. doi: 10.1002/chem.201904199 (PMC7064986; doi:10.1002/chem.201904199)
Supplement: Supplementary file 1 — Supplementary [file CHEM-26-2405-s001.pdf]

CHEMISTRY
A European Journal

Supporting Information

Bridging and Conformational Control of Porphyrin Units through Non-Traditional Rigid Scaffolds

Nitika Grover+,[a] Gemma M. Locke+,[a] Keith J. Flanagan,[a] Michael H. R. Beh,[a, b] Alison Thompson,[b] and Mathias O. Senge*[a, c, d]

chem_201904199_sm_miscellaneous_information.pdf

Table of Content

1.	Experimental Section	2
2.	NMR and Mass Spectrometer Spectra	8
3.	Single Crystal X-ray Structures	76
5.	References	84

1. Experimental Section

General Information

All reactions were carried out under an argon atmosphere unless otherwise mentioned. Reactions involving moisture and/or air sensitive reagents were carried out in oven-dried glassware and with standard Schlenk line techniques. All commercial reagents and anhydrous solvents were used as received from vendors (Fischer Scientific, Sigma Aldrich and SpiroChem AG). Tetrahydrofuran (THF), diethyl ether (Et_2O), toluene, and dichloromethane (CH_2Cl_2) were obtained by passing the degassed solvents through an activated alumina column. Yields refer to chromatographically and spectroscopically (^{1}H-NMR) homogeneous material, unless otherwise noted. Reactions were monitored by thin layer chromatography (TLC) carried out on silica gel plates using UV light as a visualizing agent or *p*-anisaldehyde and heat as a staining/developing agent. Silica gel 60* Merck, 230–400 mesh or aluminum oxide (neutral, activated with 6.5% water, Brockmann Grade III) were used for flash column chromatography. Room temperature refers to 20–25 °C.

Instrumentation

Melting points are uncorrected and were measured with a Stuart SMP-50 melting point apparatus. NMR spectra were recorded using Bruker DPX400 (400 MHz for ^{1}H-NMR, 101 MHz for ^{13}C NMR), Bruker AV 600 (600 MHz for ^{1}H-NMR, 151 MHz for ^{13}C-NMR) and Bruker AV 400 (400 MHz for ^{1}H-NMR, 101 MHz for ^{13}C NMR) instruments. Chemical shifts are given in ppm and referenced to the residual peak of the deuterated NMR solvent. The assignment of the signals was confirmed by 2D spectra (COSY, HMBC, HSQC). ESI mass spectra were acquired in positive or negative modes as required, using a Micromass time-of-flight mass spectrometer (TOF), or a Bruker mircoOTOF-Q II spectrometer interfaced to a Dionex UltiMate 3000 LC. APCI experiments were carried out on a Bruker microOTOF-Q III spectrometer interfaced to a Dionex Ultimate 3000 C or direct insertion probe in positive or negative modes. IR spectra were recorded on a PerkinElmer Spectrum 100 FT-IR spectrometer. UV−Vis spectra were recorded in solutions using a Specord 250 spectrophotometer from Analytik Jena (1 cm path length quartz cell). *In vacuo*, either refers a Büchi Rotavapor R-200; a Büchi Rotavapor R-210; a Büchi Rotavapor R-100 with a Büchi V-491 heating bath and Büchi V-850 vacuum controller.

Caution: Most cubanes are kinetically inert, but nevertheless they are high-energy materials and should be handled with care. All reactions in microwave vials/pressure tube were performed in properly equipped fume hoods behind a blast shield.

Synthesis of precursors

Synthesis of cubane precursors. Dimethylcubane-1,4-biscarboxylate and its precursors were synthesized as per the Bliese and Tsanaktsidis method.[1] 4-(Methoxycarbonyl)cubane-1-carboxylic acid (**1**) and cubane-1,4-dicarboxylic acid (**2**) were prepared according to the procedure reported in literature.[2]

Synthesis of dipyrromethane and porphyrin precursors. Tetraphenylporphyrin (TPP) was synthesized according to the Adler-Longo method.[3] 5-(4′-aminophenyl)-10,15,20-triphenylporphyrin (**7**) and its zinc(II) complex (**8**) were synthesized by regioselective mono-nitration at the *p*-position of meso-phenyl followed by a reduction.[4] meso-free dipyrromethane (DPM) was synthesized according to the procedure reported by Lindsey and co-workers.[5] 5,15-bis(4′-methylphenyl)-10-phenylporphyrin was afforded *via* a standard porphyrin condensation[6] followed by Senge reaction conditions.[7] 5-Iodo-10,20-bis(4′-methylphenyl)-15-phenylporphyrin and its Zn(II)) complex (**40**) were obtained *via* performing known procedures in literature.[8] [5-Iodo-10,20-bis(4′-methylphenyl)-15-phenylporphyrinato]zinc(II) further subjected to Suzuki[9] and Sonogashira[10] coupling reaction conditions to get [5-(4′,4′,5′,5′-tetramethyl-1′,3′,2′-dioxaborolan-2′-yl)-10,20-bis(4′-methylphenyl)-15-phenylporphyrinato]zinc(II) (**41**) and [5-ethynyl-10,20-bis(4′-methylphenyl)-15-phenylporphyrinato]zinc(II) (**42**), respectively.

Synthesis of BCP precursors. Dimethylbicyclo[1.1.1]pentane-1,4-dicarboxylate and 3-(methoxycarbonyl)bicyclo[1.1.1]pentane-1-carboxylic acid (**30**) were synthesized from bicyclo[1.1.1]pentane-1,3-dicarboxylic acid (**31**) according to the procedure reported by Pellicciari and co-workers.[11]

All known compounds had analytical data consistent with those previously reported in the literature.

Synthesis of Aminoporphyrins (9–12)

[5-(4'-Aminophenylacetylene)-10,20-bis(4'-methylphenyl)-15-phenylporphyrinato]zinc(II) (9). Synthesized *via* General Procedure 3 from [5-iodo-10,20-bis(4′-methylphenyl)-15-phenylporphyrinato]zinc(II) (100 mg, 130 µmol), 4-ethynylaniline (76 mg, 660 µmol), $PdCl_2(PPh_3)_2$ (14 mg, 20 µmol) and CuI (7.5 mg, 40 µmol) in THF/NEt_3 (2 mL/0.66 mL). The reaction was heated to 70 °C and allowed to stir for 4 h. The crude material was purified by column chromatography (aluminum oxide, activated, Brockmann Grade III), CH_2Cl_2:hexane, 2:1). Recrystallized ($CHCl_3$/hexane) to yield green crystals. Yield = 85 mg, 88%; m.p. = >350 °C; R_f = 0.64 (SiO_2, EtOAc:hexane 1:1, v/v); ^{1}H NMR (600 MHz, THF-d_8): δ = 9.75 (d, *J* = 4.5 Hz, 2H), 8.88 (d, *J* = 4.5 Hz, 2H), 8.73 (dd, *J* = 23.2, 4.5 Hz, 4H), 8.14 (d, *J* = 4.5 Hz 2H), 8.06 (d, *J* = 7.6 Hz, 4H), 7.75–7.71 (m, 5H), 7.57 (d, *J* = 7.6 Hz, 4H), 6.77 (d, 2H), 5.01 (s, 2H), 2.69 (s, 6H) ppm; ^{13}C NMR (151 MHz, THF-d_8): δ = 153.0, 151.4, 151.0, 150.8,

150.4, 144.6, 141.5, 137.9, 135.4, 135.3, 133.7, 132.8, 132.2, 132.2, 131.3, 128.2, 128.1, 127.3, 122.5, 122.4, 115.1, 112.6, 102.4, 98.6, 91.4, 21.7 ppm; IR (neat)/cm^{-1}: ṽ = 2919 (w), 2184 (w), 1603 (m), 1509 (m), 1339 (m), 1206 (m), 1055 (w), 996 (s), 946 (w), 793 (s), 714 (m), 569 (w); UV-Vis ($CHCl_3$): λ_{max} (log ε) = 452 (5.31), 581 (4.09), 634 nm (4.52); HRMS (MALDI-TOF) *m/z* calcd. for $C_{48}H_{33}N_5Zn$ $[M]^+$ 743.2027; found 743.2029.

[5-(4'-(4″-Ethynylaniline)phenyl)-10,20-bis(4'-methylphenyl)-15-phenylporphyrinato]zinc(II) (10). Synthesized *via* General Procedure **3** from [5-(4′-ethynylphenyl)-10,20-bis(4′-methylphenyl)-15-phenylporphyrinato]zinc(II) (117 mg, 150 µmol), 4-ethynylaniline (88 mg, 750 µmol), $PdCl_2(PPh_3)_2$ (16 mg, 22 µmol) and CuI (9 mg, 45 µmol) in THF/NEt_3 (3 mL/1 mL). The reaction was heated to 70 °C and allowed to stir for 24 h. The crude material was purified by column chromatography (aluminum oxide, activated, Brockmann Grade III, CH_2Cl_2:hexane, 2:1). Recrystallized ($CHCl_3$/hexane) to yield green crystals. Yield = 72 mg, 58%; m.p. = >350 °C; R_f = 0.56 (SiO_2, CH_2Cl_2:hexane, 3:1, v/v); ^{1}H NMR (600 MHz, THF-d$_8$): δ = 8.86 (s, 4H), 8.83 (dd, *J* = 21.6, 4.5 Hz, 4H), 8.19 (dd, *J* = 7.1, 1.3 Hz, 2H), 8.16 (d, *J* = 7.9 Hz, 2H), 8.07 (d, *J* = 7.7 Hz, 4H), 7.83 (d, *J* = 7.9 Hz, 2H), 7.75–7.72 (m, 3H), 7.56 (d, *J* = 7.7 Hz, 4H), 7.34 (d, *J* = 8.2 Hz, 2H), 6.62 (d, *J* = 8.2 Hz, 2H), 4.92 (s, 2H), 2.69 (s, 6H) ppm; ^{13}C NMR (151 MHz, THF-d$_8$): δ = 151.3, 151.3, 151.1, 150.9, 150.3, 144.8, 143.9, 141.8, 137.8, 135.5, 135.4, 133.7, 132.6, 132.4, 132.2, 132.1, 130.1, 128.2, 128.0, 127.3, 124.7, 121.7, 121.6, 120.9, 114.8, 111.5, 92.9, 87.5, 21.7 ppm; IR (neat)/cm^{-1}: ṽ = 2921 (w), 2852 (w), 1599 (w), 1515 (m), 1337 (m), 1286 (w), 1179 (w), 1067 (w), 994 (s), 793 (s), 719 (m), 561 (w); UV-Vis (THF): λ_{max} (log ε) = 427 (5.81), 559 (4.38), 600 nm (4.20); HRMS (MALDI-TOF) *m/z* calcd. for $C_{54}H_{37}N_5Zn$ $[M]^+$ 819.2340; found 819.2344.

[5-(3'-Aminophenylacetylene)-10,20-bis(4'-methylphenyl)-15-phenylporphyrinato]zinc(II) (11). Synthesized *via* General Procedure 3 from [5-iodo-10,20-bis(4′-methylphenyl)-15-phenylporphyrinato]zinc(II) (100 mg, 130 µmol), 3-ethynylaniline (74 µL, 660 µmol), $PdCl_2(PPh_3)_2$ (14 mg, 20 µmol) and CuI (7.5 mg, 40 µmol) in THF/NEt_3 (2 mL/0.66 mL). The reaction was heated to 70 ^{o}C and allowed to stir for 4 h. The crude material was purified by column chromatography (SiO_2, CH_2Cl_2:hexane, 2:1). Recrystallized ($CHCl_3$/hexane) to yield green crystals. Yield = 90 mg, 92%; m.p. = 238–240 °C; R_f = 0.59 (SiO_2, EtOAc:hexane, 1:1, v/v); ^{1}H NMR (600 MHz, THF-d$_8$): δ = 9.76 (d, *J* = 4.5 Hz, 2H), 8.91 (d, *J* = 4.5 Hz, 2H), 8.75 (dd, *J* = 19.4, 4.5 Hz, 4H), 8.15 (d, *J* = 7.6 Hz, 2H), 8.07 (d, *J* = 7.6 Hz, 4H), 7.74–7.70 (m, 3H), 7.58 (d, *J* = 7.6 Hz, 4H), 7.27 (d, *J* = 7.7 Hz, 2H), 7.22 (t, *J* = 7.7 Hz, 1H), 6.71 (d, *J* = 7.7 Hz, 1H), 4.76 (brs, 2H), 2.70 (s, 6H) ppm; ^{13}C NMR (151 MHz, THF-d$_8$): δ = 153.2, 151.6, 150.9, 149.9, 144.5, 141.4, 138.0, 135.4, 135.3, 133.1, 133.0, 132.9, 132.4, 132.3, 131.3, 130.1, 129.3, 129.2, 128.3, 127.3, 125.9, 123.2, 122.7, 120.9, 117.8, 115.6, 100.8, 97.7, 92.7, 21.7 ppm; IR (neat)/cm^{-1}: ṽ = 3016 (w), 2970 (w), 1738 (s), 1439 (w), 1365 (m), 1228 (m), 1217 (m), 995 (w), 894 (w), 793 (w), 714 (w), 700 w); UV-Vis ($CHCl_3$): λ_{max} (log ε) = 444 (5.64), 566 (4.31), 611 nm(4.09); HRMS (MALDI-TOF) *m/z* calcd. for $C_{48}H_{33}N_5Zn$ $[M]^+$ 743.2027; found 743.2024.

[5-(2'-Aminophenylacetylene)-10,20-bis(4'-methylphenyl)-15-phenylporphyrinato]zinc(II) (12). Synthesized *via* General Procedure 3 from [5-iodo-10,20-bis(4′-methylphenyl)-15-phenylporphyrinato]zinc(II) (100 mg, 130 µmol), 2-ethynylaniline (148 µL, 660 µmol), $PdCl_2(PPh_3)_2$ (14 mg, 20 µmol) and CuI (7.5 mg, 40 µmol) in THF/NEt_3 (2 mL/0.66 mL). The reaction was heated to 70 ^{o}C and allowed to stir for 4 h. The crude material was purified by column chromatography (SiO_2, CH_2Cl_2:hexane, 2:1). Recrystallized ($CHCl_3$/hexane) to yield green crystals. Yield = 75 mg, 77%; m.p. = 234–237 °C; R_f = 0.79 (SiO_2, EtOAc:hexane 1:1, v/v); ^{1}H NMR (600 MHz, $CDCl_3$): δ = 8.87 (d, *J* = 4.3 Hz, 2H), 8.81 (d, *J* = 4.3 Hz, 2H), 8.75 (d, *J* = 4.3 Hz, 2H), 8.37 (brs, 2H), 8.32 (d, *J* = 4.3 Hz, 2H), 8.08 (d, *J* = 6.8 Hz, 4H), 7.82–7.79 (m, 3H), 7.62 (d, *J* = 6.8 Hz, 4H), 6.87 (d, *J* = 7.1 Hz, 1H), 6.53 (t, *J* = 7.1 Hz, 1H), 6.22 (t, *J* = 7.1 Hz, 1H), 3.20 (brs, 1H), 2.84 (s, 6H), -0.58 (brs, 2H) ppm; ^{13}C NMR (151 MHz, $CDCl_3$): δ = 152.2, 150.8, 150.0, 149.6, 143.5, 140.2, 136.9, 134.8, 134.6, 132.7, 131.8, 129.9, 129.8, 127.5, 127.4, 127.2, 126.7, 122.5, 121.8, 121.1, 115.0, 110.9, 99.2, 98.2, 88.1, 21.8 ppm; IR (neat)/cm^{-1}: ṽ = 1596 (w), 1488 (m), 1439 (w), 1339 (m), 1205 (m), 1179 (m), 1064 (w), 996 (s), 946 (w), 825 (w), 792 (s), 741 (s), 708 (s), 662 (w), 609 (w), 568 (w); UV-Vis ($CHCl_3$): λ_{max} (log ε) = 441 (5.48), 568 (4.28), 614 nm (4.35); HRMS (MALDI-TOF) *m/z* calcd. for $C_{48}H_{33}N_5Zn$ $[M]^+$ 743.2027; found 743.2015.

Synthesis of Cubane Porphyrin Monomers (19–24)

[5-{4′-(4″-Carbamoyl-1″-methoxycarbonylcubane)phenyl}-10,15,20-triphenylporphyrinato]zinc(II) (19). Synthesized *via* General Procedure 1 from 4-(methoxycarbonyl)cubane-1-carboxylic acid (38 mg, 80 µmol), [5-(4′-aminophenyl)-10,15,20-triphenylporphyrinato]zinc(II) (38 mg, 55 µmol), HATU (27 mg, 72 µmol), HOAt (10 mg, 72 µmol) and DIPEA (38 µL) in anhydrous DMF (0.5 mL). The products were extracted with a mixture of CH_2Cl_2/MeOH (× 3), dried over $MgSO_4$ and the solvent removed under reduced pressure. The crude material was purified by column chromatography (SiO_2, CH_2Cl_2). The crude material was purified by column chromatography (SiO_2, CH_2Cl_2:EtOAc, 100:0 to 98.8:0.02). The product was recrystallized from CH_2Cl_2/MeOH and obtained as purple crystals. Yield = 25 mg, 52%; m.p. = >350 °C; R_f = 0.65 (SiO_2, EtOAc:CH_2Cl_2, 1:9, v/v); ^{1}H NMR (600 MHz, DMSO-d$_6$): δ = 10.10 (s, 1H), 8.82 (d, *J* = 4.5 Hz, 2H), 8.77 (s, 6H), 8.18 (d, *J* = 6.0 Hz, 6H), 8.12–8.08 (m, 4H), 7.80 (d, *J* = 6.0 Hz, 9H), 4.42 (t, *J* = 4.7 Hz, 3H), 4.28 (t, *J* = 4.7 Hz, 3H), 3.69 (s, 3H) ppm; ^{13}C NMR (151 MHz, DMSO-d$_6$): δ = 171.3, 169.7, 149.5, 149.2, 149.2, 142.7, 138.5, 137.6, 134.4, 134.2, 131.6, 131.5, 127.4, 126.6, 120.3, 120.2, 120.2, 117.6, 58.0, 55.0, 51.3, 46.7, 46.1 ppm; IR (neat)/cm^{-1}: ṽ = 2986 (w), 1722

(w), 1655 (m), 1594 (w), 1507 (m), 1440 (w), 1203 (m), 1067 (w), 992 (s), 797 (s), 751 (m), 703 (m), 604 (w); UV-Vis ($CHCl_3$): λ_{max} (log *ε*) = 422 (5.69), 549 (4.25), 590 nm (3.37); HRMS (MALDI-TOF) *m/z* calcd. for $C_{55}H_{37}N_5O_3Zn$ $[M]^+$: 879.2188, found 879.2172.

[5-{4′-(4″-Carbamoylcubane-1″-carboxylate)phenyl}-10,15,20-triphenylporphyrinato]zinc(II) (20). [5-(Methyl-4′-(phenyl carbamoyl)cubane-1′-carboxylate)-10,15,20-triphenylporphyrinato]zinc(II) (**19**) (30 mg, 34 µmol) was dissolved in mixture of THF/MeOH (2 mL:1 mL). LiOH (1 mg, 41 µmol) in H_2O (4 mL) was added to above solution and reaction mixture was stirred at rt for 18h. Reaction mixture was washed with 1M HCl and extracted with a mixture of $CHCl_3$/THF, organic layer was dried over $MgSO_4$ and solvents were removed *in vacuo*. The product was obtained as purple crystals. Yield = 29 mg, quantitative; m.p. = >350 °C; R_f = 0.35 (SiO_2, $CHCl_3$/MeOH, 6:1); ^1H NMR (600 MHz, 5:$CDCl_3$/2:$(CD_3)_2CO$/ 0.01:Pyridine-d_5): δ = 8.79 (s, 1H), 8.63 (d, *J* = 4.5 Hz, 2H), 8.59–8.58 (m, 6H), 7.92 (d, *J* = 6.4 Hz, 6H), 7.86 (d, *J* = 8.1 Hz, 2H), 7.77 (d, *J* = 8.1 Hz, 2H), 7.49–7.42 (m, 9H), 7.01 (s, 1H), 4.26–4.17 (m, 3H), 4.10–4.06 (m, 3H) ppm; ^{13}C NMR (151 MHz, 5:$CDCl_3$/2:$(CD_3)_2CO$/0.01:Pyridine-d_5): δ = 173.4, 170.1, 149.8, 149.6, 149.6, 143.1, 138.6, 137.6, 134.5, 134.2, 134.1, 131.2, 126.8, 125.9, 120.1, 120.1, 119.8, 117.3, 58.4, 56.0, 46.9, 46.4 ppm; IR (neat)/cm^{-1}: ṽ = 3325 (w), 2959(w), 2923 (w), 22852 (w), 1648 (w), 1595 (w), 1511 (w), 1440 (w), 1400 (w), 1338 (w), 1260 (w), 1069 (s), 1001 (s), 795 (s), 700 (s), 558 (m); UV-Vis ($CHCl_3$): λ_{max} (log *ε*) = 421 (6.54), 549 nm (5.15); HRMS (MALDI-TOF) *m/z* calcd. for $C_{54}H_{35}N_5O_3Zn$ $[M]^+$ 865.2031; found 865.2042.

[5-{4′-(4″-Carbamoyl-1″-methoxycarbonylcubane)phenyl}-10,15,20-triphenylporphyrin (21). Synthesized *via* General Procedure 1 from 4-(methoxycarbonyl)cubane-1-carboxylic acid (197.4 mg, 956 µmol), 5-(4′-aminophenyl)-10,15,20-triphenylporphyrin (200 mg, 318 µmol), HATU (158mg, 413 µmol), HOAt (56 mg, 143 µmol) and DIPEA (222 µL) in anhydrous DMF (1 mL). The product was extracted with CH_2Cl_2 (×3), washed with H_2O (×4), dried over $MgSO_4$ and the solvent removed under reduced pressure. The crude material was purified by column chromatography (SiO_2, CH_2Cl_2:hexane, 1:1). Product was obtained as purple crystals. Yield =197 mg, 75%; m.p. = 229–232 °C; R_f = 0.88 (SiO_2, CH_2Cl_2:MeOH, 20:1, v/v); ^1H NMR (400 MHz, $CDCl_3$):δ = 8.85 (d, *J* = 3.9 Hz, 8H), 8.20 (t, *J* = 8.1 Hz, 8H), 7.97 (d, *J* = 8.1 Hz, 2H), 7.79–7.73 (m, 9H), 7.49 (s, 1H), 4.49–4.44 (m, 3H), 4.43–4.38 (m, 3H), 3.78 (s, 3H), -2.78 (s, 2H) ppm; ^{13}C NMR (101 MHz, $CDCl_3$): δ = 172.1, 169.9, 142.3, 138.4, 137.4, 135.3, 134.6, 127.8, 126.8, 120.3, 118.1, 58.8 , 56.1, 51.9, 47.8, 47.5, 46.9, 46.8 ppm; IR (neat)/cm^{-1}: ṽ = 1711 (m), 1662 (m), 1591 (m), 1520 (m), 1440 (m), 1323 (m), 1219 (m), 980 (m), 965 (m), 796 (s), 721 (m), 698 (s); UV-Vis (CH_2Cl_2): λ_{max} (log *ε*) = 419 (6.75), 516 (5.33), nm 551 (5.01), 591 (4.83), 647 (4.75); HRMS (MALDI-TOF) *m/z* calcd. for $C_{55}H_{39}N_5O_3$ $[M]^+$ 817.3052; found 817.3053.

[5-{4′-(4″-Carbamoylcubane-1″-carboxylate)phenyl}-10,15,20-triphenylporphyrin (22). 5-(Methyl-4′-(phenylcarbamoyl)cubane-1′-carboxylate)-10,15,20-triphenylporphyrin (**21**) (63 mg, 77 µmol) was dissolved in a mixture of THF/MeOH (6 mL:2 mL). LiOH (3.7 mg, 154 µmol) in 3 mL H_2O was added dropwise to the solution. The reaction mixture was stirred at rt. for 18 h and then diluted with H_2O. Solution was acidified to pH 5 with 1M HCl and extracted with a mixture of $CHCl_3$ and THF, organic phase was dried over $MgSO_4$ and solvents were removed *in vacuo*. The product was obtained as purple crystals. Yield = 55 mg, 98%; m.p. = >350 °C; R_f = 0 (SiO_2, CH_2Cl_2, v/v); ^1H NMR (400 MHz, DMSO-d_6): δ = 10.10 (s, 1H), 8.89 (d, 2H), 8.83 (s, 6H), 8.23–8.21 (m, 7H), 8.15 (s, 4H), 7.84–7.72 (m, 9H), 4.40–4.38 (m, 3H), 4.24–4.21 (m, 3H), -2.94 (s, 2H) ppm; ^{13}C NMR (151 MHz, DMSO-d_6): δ = 170.1, 141.2, 139.0, 135.9, 134.6, 134.2, 128.1, 127.0, 120.0, 119.9, 119.9, 117.9, 67.0, 58.0, 46.7, 46.5, 46.1, 46.0 ppm; IR (neat)/cm^{-1}: ṽ = 3344 (w), 1691 (m), 1587 (m), 1517 (m), 1400 (m), 1339 (w), 1160 (m), 1094 (m), 966 (s), 801 (s), 729 (s), 697 (s), 618 (m); UV-Vis (CH_3OH): λ_{max} (log *ε*) = 415 (5.52), 513 (4.16), 548 (3.88), 591 (3.78), 647 nm (3.56); HRMS (MALDI-TOF) *m/z* calcd. for $C_{54}H_{37}N_5O_3$ $[M]^+$ 803.2896; found 803.2894.

[5-{4′-(4″-Carbamoyl-1″-methoxycarbonylcubane)ethynylphenyl}-10,20-bis(4′-methylphenyl)-15-phenylporphyrinato]zinc(II) (23). Synthesized *via* General Procedure 4 from [5-ethynyl-10,20-bis(4′-methylphenyl)-15-phenylporphyrinato]zinc(II) (60 mg, 90 µmol), cubane **13** (25 mg, 60 µmol), $PdCl_2(PPh_3)_2$ (6.3 mg, 9 µmol) and CuI (3.4 mg, 18 µmol) in anhydrous THF/NEt_3 (1 mL:0.33 mL). The reaction was allowed to stir at 70 °C for 4 h and then diluted with CH_2Cl_2 (10 mL) before removal of solvents *in vacuo.* The crude material was purified by column chromatography (SiO_2, CH_2Cl_2:hexane, 2:1). Recrystallized ($CHCl_3$/hexane) to yield purple crystals. Yield = 42 mg, 75%; m.p. = >350 °C; R_f = 0.81 (SiO_2, CH_2Cl_2:EtOAc, 9:1, v/v); ^1H NMR (400 MHz, $CDCl_3$): δ = 9.80 (d, *J* = 4.5 Hz, 2H), 9.02 (d, *J* = 4.5 Hz, 2H), 8.86 (dd, *J* = 15.8, 4.5 Hz, 4H), 8.17 (d, *J* = 8.3 Hz, 2H), 8.09 (d, *J* = 7.8 Hz, 4H), 7.94 (d, *J* = 8.3 Hz, 2H), 7.76–7.69 (m, 3H), 7.57 (d, *J* = 7.8 Hz, 4H), 7.48 (d, 2H), 7.10 (brs, 1H), 4.21–4.20 (m, 3H), 4.14–4.13 (m, 3H), 3.71 (s, 3H), 2.72 (s, 6H) ppm; ^{13}C NMR (151 MHz, $CDCl_3$): δ = 171.9, 152.3, 150.8, 150.1, 142.8, 139.7, 137.3, 134.5, 134.4, 133.0, 132.5, 132.2, 132.1, 130.9, 127.7, 127.5, 126.7, 122.7, 122.2, 119.5, 100.3, 96.0, 92.7, 65.4, 58.4, 51.8, 47.3, 46.7, 21.7 ppm; IR (neat)/cm^{-1}: ṽ = 2970 (w), 2923 (w), 1736 (s), 1657 (m), 1582 (w), 1511 (s), 1434 (m), 1403 (m), 1339 (m), 1216 (s), 1205 (s), 1181 (m), 1088 (m), 1064 (w), 996 (s), 945 (w), 836 (m), 795 (s), 716 (m), 701 (m), 596 (w); UV-Vis ($CHCl_3$): λ_{max} (log *ε*) = 444 (5.64), 568 (4.29), 614 nm (4.47); HRMS (MALDI-TOF) *m/z* calcd. for $C_{59}H_{41}N_5O_3Zn$ $[M]^+$ 931.2501; found 931.2469.

[5-{4′-(4″-Ethynylphenyl)carbamoyl)-1″-methoxycarbonylcubane)phenyl}-10,20-bis(4′-methylphenyl)-15-phenylporphyrinato]zinc(II) (24). Synthesized *via* General Procedure 4 from [5-(4′-ethynylphenyl)-10,20-bis(4′-methylphenyl)-15-phenylporphyrinato]zinc(II) (25 mg, 60 µmol), cubane 13 (66 mg, 90 µmol) $PdCl_2(PPh_3)_2$ (6.3 mg, 9 µmol) and CuI (3.4 mg, 18 µmol) in anhydrous THF/NEt_3 (1 mL:0.33 mL). The reaction was allowed to stir at 65 ^oC for 4 h and then diluted with CH_2Cl_2 (10 mL) before removal of solvents *in vacuo.* The crude material was purified by column chromatography (SiO_2, CH_2Cl_2:$(CH_3)_2CO$ = 100:0 to 98.8:0.02). Recrystallized ($CHCl_3$/hexane) to yield purple crystals. Yield = 34 mg, 56%; m.p. = >350 °C; R_f = 0.64 (SiO_2, CH_2Cl_2: EtOAc, 9:1, v/v); ^1H NMR (600 MHz, $CDCl_3$): δ = 9.00–8.93 (m, 8H), 8.22 (t, *J* = 7.6 Hz, 4H), 8.10 (d, *J* = 7.4 Hz, 4H), 7.92 (d, *J* = 7.6 Hz, 2H), 7.78–7.74 (m, 3H), 7.62 (d, *J* = 8.1 Hz, 2H), 7.56 (d, *J* = 7.4 Hz, 6H), 7.17 (s, 1H), 4.28 (s, 6H), 3.74 (s, 3H), 2.72 (s, 6H) ppm; ^{13}C NMR (151 MHz, $CDCl_3$): δ = 171.9, 169.4, 150.6, 150.5, 150.3, 149.9, 143.2, 143.0, 139.9, 137.7, 137.3, 134.6, 134.6, 134.5, 132.7, 132.4, 132.2, 132.1, 131.7, 129.9, 127.6, 127.5, 126.7, 122.6, 121.5, 121.3, 120.2, 119.5, 90.2, 89.5, 58.6, 56.0, 51.9, 47.4, 46.8, 21.7 ppm; IR (neat)/cm^{-1}: ṽ = 2970 (w), 1738 (s), 1486 (w), 1435 (w), 1365 (m), 1217 (s), 1204 (s), 1086 (w), 994 (m), 794 (m), 1217 (w), 1061 (w), 1007 (m), 955 (w), 809 (s), 795 (m), 717 (m), 701 (w), 568 (w); UV-Vis ($CHCl_3$): λ_{max} (log *ε*) 423 (5.72), 550 (4.34), 591 nm (3.80); HRMS (MALDI-TOF) *m/z* calcd. for $C_{65}H_{45}N_5O_3Zn$ $[M]^+$ 1007.2814; found 1007.2787.

Synthesis of Porphyrin Precursors for 'Pd-catalyzed Coupling Reactions'

[5-(4′-Bromophenyl)-10,20-bis(4′-methylphenyl)-15-phenylporphyrinato]zinc(II). [5-Iodo-10,20-bis(4′-methylphenyl)-15-phenylporphyrinato]zinc(II) (150 mg, 200 µmol), 4-bromophenyl pinacol borane (566 mg, 2 mmol) and K_3PO_4 (850 mg, 4 mmol) were placed in an oven-dried Schlenk flask and heated under vacuum. The reaction flask was purged with argon and THF (13 mL) was added. Argon was bubbled through the solution for 15 min then $Pd(PPh_3)_4$ (46 mg, 40 µmol) was added. The reaction was heated to 70 ^oC and allowed to stir for 4 h. The reaction mixture was diluted with CH_2Cl_2 (10 mL) before removal of solvents *in vacuo.* The crude material was purified by column chromatography (SiO_2, CH_2Cl_2:hexane, 1:1). Recrystallized ($CHCl_3$/hexane) to yield pink/purple crystals. Yield = 147 mg, 95%; m.p. = >350 °C; R_f = 0.49 (SiO_2, CH_2Cl_2:hexane, 1:1, v/v); ^1H NMR (400 MHz, $CDCl_3$) δ = 8.98 (t, *J* = 4.7 Hz, 4H), 8.92 (dd, *J* = 11.1, 4.7 Hz, 4H), 8.21 (d, *J* = 8.3 Hz, 2H), 8.10 (d, *J* = 7.6 Hz, 6H), 7.89 (d, *J* = 8.3 Hz, 2H), 7.79–7.72 (m, 3H), 7.56 (d, *J* = 7.6 Hz, 4H), 2.72 (s, 6H) ppm; ^{13}C NMR (151 MHz, $CDCl_3$) δ = 150.6, 150.5, 150.4, 149.9, 142.9, 142.0, 139.9, 137.3, 135.9, 134.5, 132.4, 132.3, 132.1, 131.6, 129.9, 127.7, 127.6, 126.7, 122.3, 121.6, 119.4, 21.7 ppm; IR (neat)/cm^{-1}: ṽ = 1481 (w), 1337 (m), 1205 (w), 1179 (w), 1067 (m), 997 (s), 847 (w), 794 (s), 746 (m), 720 (m), 699 (m), 661 (w); UV-Vis ($CHCl_3$): λ_{max} (log *ε*) = 401 (4.64), 422 (5.80), 550 nm (4.38); HRMS (MALDI-TOF) *m/z* calcd. for $C_{46}H_{31}N_4BrZn$ $[M]^+$ 782.1024; found 782.1027.

[5-Iodo-10,20-bis(4′-methylphenyl)-15-phenylporphyrinato]zinc(II) (40). Synthesized according to conventional synthetic procedure reported in literature.[8] Yield = 750 mg, 83%; m.p. = >350 °C; R_f = 0.73 (SiO_2, CH_2Cl_2:hexane, 2:1, v/v); ^1H NMR (400 MHz, $CDCl_3$): δ = 9.77 (d, *J* = 4.7 Hz, 2H), 8.98 (d, *J* = 4.7 Hz, 2H), 8.91 (dd, *J* = 11.4, 4.7 Hz, 4H), 8.19–8.17 (m, 2H), 8.07 (d, *J* = 7.9 Hz, 4H), 7.78–7.71 (m, 3H), 7.57 (d, *J* = 7.9 Hz, 4H), 2.73 (s, 6H) ppm; ^{13}C NMR (101 MHz, $CDCl_3$): δ = 152.2, 151.5, 150.8, 150.7, 142.7, 139.6, 137.8, 137.5, 134.5, 134.4, 133.8, 132.5, 132.5, 127.7, 127.5, 126.7, 122.2, 122.1, 21.7 ppm; IR (neat)/cm^{-1}: ṽ =1490 (w), 1320 (w), 1211 (w), 1074 (w), 996 (s), 795 (s), 782 (s), 755 (m), 722 (m), 707 (m), 567 (w); UV-Vis ($CHCl_3$): λ_{max} (log *ε*) = 426 (5.79), 555 (4.41), 594 nm (3.96); HRMS (MALDI-TOF) *m/z* calcd. for $C_{40}H_{27}N_4ZnI$ $[M]^+$ 754.0572; found 754.0590.

[5-(4′,4′,5′,5′-Tetramethyl-1′,3′,2′-dioxaborolan-2′-yl)-10,20-bis(4′-methylphenyl)-15-phenylporphyrinato]zinc(II) (41). [5-Iodo-10,20-bis(4′-methylphenyl)-15-phenylporphyrinato]zinc(II) (100 mg, 0.13 mmol) was placed in an oven-dried Schlenk tube and heated under vacuum. The flask was purged with argon followed by addition of 10 mL anhydrous 1,2-dichloroethane. Argon was bubbled through the solution for 5–10 min. $PdCl_2(PPh_3)_2$ (16 mg, 22 µmol) and 4,4,5,5-tetramethyl-1,3,2-dioxaborolane (0.19 mL, 1.3 mmol) were added and the reaction mixture was allowed to stir for 2 h at 90 ^oC. The solvent was then removed *in vacuo.* The crude reaction mixture was purified by silica gel column chromatography using CH_2Cl_2:hexane (2:1) to afford the title compound as a bright pink solid. Yield = 90 mg, 91%; m.p. = >350 °C; R_f = 0.43 (SiO_2, CH_2Cl_2:hexane, 2:1, v/v); ^1H NMR (600 MHz, $CDCl_3$): δ = 9.90 (d, *J* = 4.6 Hz, 2H), 9.10 (d, *J* = 4.6 Hz, 2H), 8.95 (dd, *J* = 14.8, 4.6 Hz, 4H), 8.21 (d, *J* = 4.6 Hz, 2H), 8.10 (d, *J* = 7.6 Hz, 4H), 7.77–7.72 (m, 3H), 7.56 (d, *J* = 7.6 Hz, 4H), 2.72 (s, 6H), 1.86 (s, 12H) ppm; ^{13}C NMR (151 MHz, $CDCl_3$): δ = 154.5, 150.7, 150.3, 149.4, 143.0, 140.1, 137.2, 134.6, 134.5, 133.2, 132.8, 132.1, 131.7, 127.6, 127.4, 126.6, 122.6, 121.2, 85.4, 25.5, 21.7 ppm; IR (neat)/cm^{-1}: ṽ = 1527 (w), 1446 (w), 1367 (w), 1303 (m), 1203 (w), 1143 (m), 1061 (m), 996 (s), 856 (w), 795 (s), 719 (m), 699 (m), 661 (w); UV-Vis ($CHCl_3$): λ_{max} (log *ε*) = 420 (5.72), 549 nm (4.34); HRMS (MALDI-TOF) *m/z* calcd. for $C_{46}H_{39}N_4O_2ZnB$ $[M]^+$ 754.2458; found 754.2459.

[5-Ethynyl-10,20-bis(4′-methylphenyl)-15-phenylporphyrinato]zinc(II) (42). Step 1. Synthesized according to known synthetic procedure[10] using [5-Iodo-10,20-bis(4′-methylphenyl)-15-phenylporphyrinato]zinc(II) (150 mg, 0.2 mmol), $PdCl_2(PPh_3)_2$ (21 mg, 30 µmol), ethynyltrimethylsilane (0.28 mL, 2.0 mmol) and CuI (11.5 mg, 60 µmol) in anhydrous THF and NEt_3 (2 mL:0.67 mL). The reaction was heated to 70 ^oC and allowed to stir for 3 h.

The crude material was purified by column chromatography (SiO_2, CH_2Cl_2:hexane, 2:1) then recrystallized ($CHCl_3$/hexane) to yield purple crystals. Yield = 97 mg, 67%; m.p. = >350 °C; R_f = 0.83 (SiO_2, CH_2Cl_2:hexane, 2:1, v/v); ^1H NMR (600 MHz, $CDCl_3$): δ = 9.75 (d, *J* = 4.5 Hz, 2H), 9.01 (d, *J* = 4.5 Hz, 2H), 8.87 (dd, *J* = 21.9, 4.5 Hz, 4H), 8.17 (d, *J* = 4.5 Hz, 2H), 8.08 (d, *J* = 7.6 Hz, 4H), 7.77–7.72 (m, 3H), 7.57 (d, *J* = 7.6 Hz, 4H), 2.72 (s, 6H), 0.61 (s, 9H) ppm; ^{13}C NMR (151 MHz, $CDCl_3$): δ = 152.8, 150.9, 150.3, 150.0, 142.8, 139.6, 137.4, 134.5, 134.4, 133.2, 132.3, 132.0, 131.1, 127.7, 127.5, 126.7, 122.9, 122.2, 107.8, 101.4, 99.5, 21.7, 0.6 ppm; IR (neat)/cm^{-1}: ṽ = 2149 (w), 1489 (w), 1434 (w), 1338 (w), 1248 (w), 1209 (w), 1064 (w), 999 (m), 839 (s), 795 (s), 699 (s), 559 (w); UV-Vis ($CHCl_3$): λ_{max} (log *ε*) = 432 (5.70), 563 (4.32), 604 nm (4.18); HRMS (MALDI-TOF) *m/z* calcd. for $C_{45}H_{36}N_4SiZn$ $[M]^+$ 724.2001; found 724.2002.

Step 2. [5-Phenyl-10,20-bis(4′-methylphenyl)-15-trimethylsilyl ethynylporphyrinato]zinc(II) (176 mg, 0.24 mmol) was placed in an oven-dried Schlenk flask and heated under vacuum. The flask was purged with argon and anhydrous THF (7 mL) was added by syringe. 1M solution of TBAF (0.36 mL, 0.36 mmol) was added dropwise to the solution at room temperature and then stirred under N_2. Progress of reaction was monitored by TLC, reaction mixture was diluted with CH_2Cl_2 (10 mL) solvents was evaporated *in vacuo.* The crude mixture was purified by column chromatography (SiO_2, CH_2Cl_2:hexane = 2:1) then recrystallized ($CHCl_3$/hexane) to yield purple/green crystals. Yield = 125 mg, 80%; m.p. = >350 °C; R_f = 0.8 (SiO_2, CH_2Cl_2:hexane, 2:1, v/v); ^1H NMR (600 MHz, $CDCl_3$): δ = 9.76 (d, *J* = 4.5 Hz, 2H), 9.03 (d, *J* = 4.5 Hz, 2H), 8.89 (dd, *J* = 18.8, 4.5 Hz, 4H), 8.18 (d, 4.5 Hz, 2H), 8.08 (d, *J* = 7.7 Hz, 4H), 7.77–7.72 (m, 3H), 7.57 (d, *J* = 7.7 Hz, 4H), 4.15 (s, 1H), 2.72 (s, 6H) ppm; ^{13}C NMR (151 MHz, $CDCl_3$): δ = 152.9, 151.0, 150.3, 150.0, 142.7, 139.6, 137.5, 134.5, 134.4, 133.3, 132.4, 132.1, 131.1, 127.8, 127.5, 126.7, 123.1, 122.2, 98.3, 86.3, 83.5, 21.7 ppm; IR (neat)/cm^{-1}: ṽ = 3265 (w), 1493 (w), 1441 (w), 1339 (w), 1209 (w), 1071 (m), 997 (s), 791 (s), 756 (m), 714 (s), 639 (m); UV-Vis ($CHCl_3$): λ_{max} (log *ε*) = 428 (5.69), 560 (4.37), 600 (4.03); HRMS (MALDI-TOF) *m/z* calcd. for $C_{42}H_{28}N_4Zn$ $[M]^+$ 653.1684; found 653.1669.

5-(4′-Ethynylphenyl)-10,20-bis(4′-methylphenyl)-15-phenylporphyrinato]zinc(II) (43). Step 1. An oven-dried Schlenk tube was charged with [5-Iodo-10,20-bis(4′-methylphenyl)-15-phenylporphyrinato]zinc(II) (200 mg, 260 µmol), [(trimethylsilyl)ethynyl]phenyl boronic acid pinacol ester (794 mg, 2.7 mmol), and K_3PO_4 (1.13 g, 5.3 mmol). The tube was purged with argon and anhydrous THF (17 mL) was added by syringe. $Pd(PPh_3)_4$ (60 mg, 52 µmol) was added to the above reaction mixture and heated at 70 ^oC and allowed to stir for 18 h. The solvent was then removed *in vacuo.* The crude material was purified by column chromatography (SiO_2, CH_2Cl_2:hexane, 2:1) then recrystallized ($CHCl_3$/hexane) to yield purple crystals. Yield =145 mg, 70%; m.p. = >350 °C; R_f = 0.49 (SiO_2, CH_2Cl_2:hexane, 2:1, v/v); ^1H NMR (400 MHz, $CDCl_3$): δ = 8.98 (dd, *J* = 4.8, 0.9 Hz, 4H), 8.92 (dd, *J* = 13.4, 4.8 Hz, 4H), 8.22 (d, 4.8 Hz, 2H), 8.17 (d, *J* = 8.0 Hz, 2H), 8.09 (d, *J* = 7.8 Hz, 4H), 7.87 (d, *J* = 8.0 Hz, 2H), 7.78–7.71 (m, 3H), 7.55 (d, *J* = 7.8 Hz, 4H), 2.71 (s, 6H), 0.38 (s, 9H) ppm; ^{13}C NMR (101 MHz, $CDCl_3$): δ = 134.6, 134.5, 132.4, 132.2, 132.1, 131.2, 130.4, 127.5, 126.7, 125.9, 105.3, 95.7, 84.1, 25.0, 0.1 ppm; IR (neat)/cm^{-1}: ṽ = 2958 (w), 2158 (w), 2204 (w), 1607 (w), 1354 (m), 1249 (m), 1139 (m), 999 (m), 840 (s), 796 (s), 652 (s); UV-Vis ($CHCl_3$): λ_{max} (log *ε*) = 423 (5.30), 551 nm (3.96); HRMS (MALDI -TOF) *m/z* calcd. for $C_{51}H_{40}N_4SiZn$ $[M]^+$ 800.2314; found 800.2326.

Step 2. [5-Phenyl-10,20-bis(4′-methylphenyl)-15-(4′-trimethylsilylethynylphenyl)porphyrinato]zinc(II) 128 mg, 160 µmol) was placed in an oven-dried Schlenk flask and heated under vacuum. The flask was purged with argon and anhydrous THF was added by syringe. 1M solution of TBAF (240 µL, 240 µmol) was added dropwise to the solution at rt. and then stirred under argon for 5 min. The solvent was removed *in vacuo.* The crude material was purified by column chromatography (SiO_2, CH_2Cl_2). Recrystallized ($CHCl_3$/hexane) to yield purple crystals. Yield = 116 mg, 99%; m.p. = >350 °C; R_f = 0.74 (SiO_2, CH_2Cl_2:hexane, 2:1, v/v); ^1H NMR (400 MHz, $CDCl_3$): δ = 9.03–8.88 (m, 8H), 8.21–8.19 (m, 4H), 8.10 (d, *J* = 6.2 Hz, 4H), 7.89 (d, 2H), 7.76–7.75 (m, 3H), 7.56 (d, *J* = 6.2 Hz, 4H), 3.31 (s, 1H), 2.72 (s, 6H) ppm; ^{13}C NMR (101 MHz, $CDCl_3$): δ = 150.5, 149.9, 142.9, 139.9, 137.3, 134.5, 132.4, 132.1, 130.5, 127.5, 126.7, 121.5, 29.9, 21.7 ppm; IR (neat)/cm^{-1}: ṽ = 2919 (w), 1492 (w), 1338 (w), 1206 (w), 1179 (w), 1068 (w), 996 (s), 795 (s), 720 (m), 700 (m), 652 (w), 616 (w); UV-Vis ($CHCl_3$): λ_{max} (log *ε*) = 422 (5.74), 550 nm (4.37); HRMS (MALDI-TOF) *m/z* calcd. for $C_{48}H_{32}N_4Zn$ $[M]^+$ 728.1918; found 728.1904.

Synthesis of BCP Porphyrin Monomers (44–49)

[5-{4′-(3″-Carbamoyl-1″-methoxycarbonylbicyclo[1.1.1]pentane)phenyl}-10,15,20-triphenylporphyrinato]zinc(II) (44). Synthesized according to General Procedure 4 using [5-(4′,4′,5′,5′-tetramethyl-1′,3′,2′-dioxaborolan-2′-yl)-10,20-bis(4′-methylphenyl)-15-phenylporphyrinato]zinc(II) (**41**) (42 mg, 0.058 mmol), BCP **32** (22 mg, 0.058 mmol), K_2CO_3 (80 mg, 0.58 mmol), $Pd(PPh_3)_4$ (14 mg, 0.012 mmol). The crude reaction mixture was purified by silica gel column chromatography using hexane:EtOAc, 3:1 as the eluent. Yield = 32 mg, 65%; m.p. = 220 °C; R_f = 0.85 (SiO_2, CH_2Cl_2:EtOAc, 9.5:0.5, v/v); ^1H NMR (400 MHz; $CDCl_3$): δ = 8.94 (s, 8H), 8.22–8.17 (m, 8H), 7.89 (d, *J* = 8.1 Hz, 2H), 7.79–7.71 (m, 9H), 7.48 (s, 1H), 3.76 (s, 3H), 2.49 (s, 6H) ppm; ^{13}C NMR (101 MHz; $CDCl_3$): δ = 169.6, 167.3, 150.4, 150.2, 150.1, 142.9, 139.8, 139.7, 138.0, 137.1, 136.8, 136.7, 134.9, 134.4, 134.3, 132.1, 132.0, 131.9, 131.6, 127.3, 126.5, 121.2, 117.8, 52.57, 51.80, 29.7 ppm; IR (neat)/cm^{-1}: ṽ = 2920 (w), 1728 (w), 1655 (w), 1593 (w), 1518 (w), 1439 (w), 1312 (w), 1207 (m), 1066

(w), 991 (s), 794 (s), 750 (w), 699 (s); UV-Vis ($CHCl_3$): λ_{max} (log ε) = 422 (5.55), 540 (3.95), 588 nm (3.54); HRMS(MALDI-TOF): m/z = calc. for $C_{52}H_{37}N_5O_3Zn$ $[M]^+$ 843.2188; 843.2210 found.

[5-{4′-(3″-Carbamoyl)-1″-methoxycarbonylbicyclo[1.1.1]pentane)ethynylphenyl}-10,20-(4′-methylphenyl)-15-phenylporphyrinato]zinc(II) (45). Synthesized according to General Procedure 4 using 5-ethynyl-10,20-bis(4′-methylphenyl)-15-phenylporphyrinato]zinc(II) (**42**) (42 mg, 0.064 mmol), BCP **32** (24 mg, 0.064 mmol), CuI (6.5 mg, 0.034 mmol), $Pd(PPh_3)_2Cl_2$ (8 mg, 0.011 mmol). The crude reaction mixture was purified by silica gel column chromatography using hexane:EtOAc, 3:1 as the eluent. Yield = 41 mg, 72%; m.p. = 262 °C; R_f = 0.80 (SiO_2, CH_2Cl_2:EtOAc, 9.5:0.5, v/v); ^1H NMR (400 MHz; $CDCl_3$): δ = 9.79 (d, *J* = 4.6 Hz, 2H), 9.01 (d, *J* = 4.6 Hz, 2H), 8.85 (dd, *J* = 15.3, 4.7 Hz, 4H), 8.17 (d, *J* = 4.6 Hz, 2H), 8.08 (d, *J* = 7.8 Hz, 4H), 7.96 (d, *J* = 8.5 Hz, 2H), 7.76 –7.70 (m, 3H), 7.57 (d, *J* = 7.8 Hz, 4H), 7.53 (d, *J* = 8.5 Hz, 2H), 7.22 (s, 1H), 3.73 (s, 3H), 2.72 (s, 6H), 2.28 (s, 6H) ppm; ^{13}C NMR (101 MHz; $CDCl_3$): δ = 152.1, 150.6, 149.9, 142.7, 139.5, 137.1, 134.3, 134.2, 132.3, 132.0, 131.9, 130.6, 127.3, 126.5, 122.0, 119.6, 52.4, 51.9, 45.6, 29.4, 21.5 ppm; IR (neat)/cm^{-1}: ṽ = 2914 (w), 1727 (w), 1582 (w), 1512 (m), 1314 (w), 1208 (m), 990 (s), 788 (s), 715 (m), 702 (m); UV-Vis ($CHCl_3$): λ_{max} (log ε) = 444 (5.48), 567 (4.13), 614 nm (4.30); HRMS(MALDI-TOF): m/z = calc. for $C_{56}H_{41}N_5O_3Zn$ $[M]^+$ 895.2501; 895.2527 found.

[5-{4′-(3″-((4‴-Ethynylphenyl)carbamoyl)-1″-methoxycarbonylbicyclo[1.1.1]pentane))phenyl}-10,20-bis(4′-methylphenyl)-15-phenylporphyrinato]zinc(II) (46). Synthesized according to General Procedure 4 using 5-(4′-ethynylphenyl)-10,20-bis(4′-methylphenyl)-15-phenylporphyrinato]zinc(II) (30 mg, 0.040 mmol), BCP **32** (15 mg, 0.040 mmol), CuI (10 mg, 0.06 mmol), $Pd(PPh_3)_2Cl_2$ (8 mg, 0.011 mmol). The crude reaction mixture was purified by silica gel column chromatography using hexane:EtOAc, 3:1 as the eluent. Yield = 22 mg, 58%; m.p. = 256 °C; R_f = 0.72 (SiO_2, CH_2Cl_2:EtOAc, 19:1, v/v); ^1H NMR (400 MHz; $CDCl_3$): δ = 9.02–8.95 (m, 8H), 8.24 (d, *J* = 8.0 Hz, 4H), 8.13 (d, J = 7.8 Hz, 4H), 7.94 (d, *J* = 8.0 Hz, 2H), 7.80–7.75 (m, 3H), 7.65 (t, *J* = 8.0 Hz, 4H), 7.58 (d, *J* = 7.8 Hz, 4H), 3.76(s, 3H), 2.74(s, 6H), 2.42(s, 6H) ppm; ^{13}C NMR (101 MHz; $CDCl_3$): δ = 169.6, 167.0, 150.4, 150.3, 150.2, 149.8, 143.1, 142.8, 139.8, 137.1, 134.4, 134.4, 134.3, 132.6, 132.2, 132.1, 131.9, 131.5, 129.7, 127.3, 126.5, 122.4, 121.3, 121.2, 120.1, 119.5, 90.0, 89.4, 52.5, 51.9, 46.1, 29.7, 21.5 ppm; IR (neat)/cm^{-1}: ṽ = 3018 (w), 2863 (w), 1686 (m), 1574 (w), 1523 (w), 1487 (w), 1255 (m), 1199 (s), 1069 (w), 996 (s), 794 (s), 751 (w), 718 (m); UV-Vis ($CHCl_3$): λ_{max} (log ε) = 424 (5.29), 551 (3.93), 590 nm (3.37); HRMS(MALDI-TOF): m/z = calc. for $C_{62}H_{45}N_5O_3Zn$ $[M]^+$ 971.2814; 971.2819 found.

[5-{3′-(3″-Carbamoyl-1″-methoxycarbonylbicyclo[1.1.1]pentane)phenyl}-10,20-bis(4′-methylphenyl)-15-phenylporphyrinato]zinc(II) (47). Synthesized using according to General Procedure 5 using BCP **34** (27 mg, 0.073 mmol), [5-(4′,4′,5′,5′-tetramethyl-1′,3′,2′-dioxaborolan-2′-yl)-10,20-bis(4′-methylphenyl)-15-phenylporphyrinato]zinc(II) (**41**) (50 mg, 0.066 mmol), K_2CO_3 (91 mg, 0.66 mmol) and $Pd(PPh_3)_4$ (15 mg, 0.013 mmol). The crude reaction mixture was purified using column chromatography, desired compound was eluted with CH_2Cl_2. Yield = 44 mg, 78%; m.p = 246 °C; R_f = 0.6 (SiO_2, CH_2Cl_2:EtOAc, 9.5:0.5, v/v); ^1H NMR (400 MHz; $CDCl_3$): δ = 9.01–8.99 (m, 4H), 8.97–8.95 (m, 4H), 8.24 (dd, *J* = 9.6, 3.9 Hz, 2H), 8.17 (s, 1H), 8.12 (d, *J*= 8.0 Hz, 5H), 8.03 (d, *J* = 8.0 Hz, 1H), 7.82–7.77 (m, 3H), 7.73 (t, *J* = 8.0 Hz, 1H), 7.58 (d, *J* = 8.0 Hz, 4H), 7.41 (s, 1H), 3.67 (s, 3H), 2.74 (s, 6H), 2.35 (s, 6H) ppm; ^{13}C NMR (101 MHz; $CDCl_3$): δ = 169.6, 150.4, 150.4, 150.1, 149.9, 143.7, 142.8, 139.8, 137.1, 135.5, 134.4, 134.3, 132.2, 132.0, 131.9, 131.6, 127.5, 127.3, 126.5, 125.8, 121.3, 121.2, 119.0, 52.4, 51.8, 21.5 ppm; IR (neat)/cm^{-1}: ṽ = 3020 (w), 2919 (w), 1737 (w), 1664 (w), 1527 (m), 1483 (m), 1206 (m), 997 (s), 794 (s), 717 (m), 698 (m); UV-Vis ($CHCl_3$): λ_{max} (log ε) = 422 (6.99), 550 (5.59), 589 nm (4.89); HRMS(MALDI-TOF): m/z = calc. for $C_{54}H_{41}N_5O_3Zn$ $[M]^+$ 871.2501; 871.2492 found.

[5-{3′-(3″-Carbamoyl)-1″-methoxycarbonylbicyclo[1.1.1]pentane)ethynylphenyl}-10,20-bis(4′-methylphenyl))-15-phenylporphyrinato]zinc(II) (48). To an oven-dried Schlenk tube charged with BCP **35** (20 mg, 0.075 mmol), [5-iodo-10,20-(4′-methylphenyl)-15-phenylporphyrinato]zinc(II) (50 mg, 0.066 mmol) and K_2CO_3 (91 mg, 0.66 mmol) 5mL of DMF was added. The solution was degassed for 5–10 min. followed by addition of $Pd(PPh_3)_4$ (15 mg, 0.013 mmol). Reaction mixture was heated at 100 °C for 4 h. The crude reaction mixture was purified using column chromatography (SiO_2, CH_2Cl_2). Yield = 43 mg, 73%; m.p = 316 °C; R_f = 0.4 (SiO_2, CH_2Cl_2:EtOAc, 9.5:0.5, v/v); ^1H NMR (400 MHz; $CDCl_3$): δ = 9.81 (d, *J* = 4.6 Hz, 2H), 9.03 (d, *J* = 4.6 Hz, 2H), 8.91 (d, *J* = 4.6 Hz, 2H), 8.87 (d, *J* = 4.6 Hz, 2H), 8.20(d, *J* = 4.6 Hz, 2H), 8.11 (d, *J* = 8.0 Hz, 4H), 7.97 (d, *J* = 8.0 Hz, 2H), 7.79-7.75 (m, 3H), 7.50 (d, *J* = 8.0 Hz, 4H), 7.46 (d, *J* = 8.0 Hz, 2H), 7.14 (s, 1H), 3.75 (s, 3H), 2.75 (s, 6H), 2.24 (s, 6H) ppm; ^{13}C NMR (101 MHz; $CDCl_3$): δ = 169.2, 168.9, 152.2, 150.6, 150.0, 149.9, 137.2, 134.4, 134.3, 132.8, 132.3, 132.0, 131.9, 127.3, 126.5, 122.1, 95.7, 52.3, 51.9, 21.5 ppm; IR (neat)/cm^{-1}: ṽ = 2998 (w), 2920 (w), 1727 (w), 1677 (w), 1507 (m), 1306 (w), 1208 (m), 996 (s), 836 (w), 791 (s), 785 (s), 707 (s); UV-Vis ($CHCl_3$): λ_{max} (log ε) = 444 (6.77), 568 (5.40), 619 nm (5.51); HRMS(MALDI-TOF): m/z = calc. for $C_{56}H_{41}N_5O_3Zn$ $[M]^+$ 895.2501; 895.2485 found.

[5-{3′-(3″-((4‴-Ethynylphenyl)carbamoyl)-1″-methoxycarbonylbicyclo[1.1.1]pentane))phenyl}-10,20-bis(4′-methylphenyl)-15-phenylporphyrinato]zinc(II) (49). Synthesized using according to General Procedure 5 using BCP **34** (25 mg, 0.069 mmol), 5-(4′-ethynylphenyl)-10,20-bis(4′-methylphenyl)-15-phenylporphyrinato]zinc(II) (50 mg, 0.069 mmol), $Pd(PPh_3)_4$ (10 mg, 0.0086) and K_2CO_3 (0.69 mmol) in DMF. Reaction mixture was refluxed for 4 h. The crude reaction mixture was purified using column chromatography (SiO_2, CH_2Cl_2 to CH_2Cl_2:EtOAc,

9:1). Yield = 34 mg, 52%; m.p = 207 °C; R_f = 0.8 (SiO_2, CH_2Cl_2:EtOAc, 8:2, v/v); ^{1}H NMR (400 MHz; $CDCl_3$): δ = 9.00–8.98 (m, 4H), 8.95–8.93(m, 4H), 8.22 (d, *J* = 8.1 Hz, 4H), 8.11 (d, *J* = 7.7 Hz, 4H), 7.91(d, *J* = 7.8 Hz, 2H), 7.83 (s, 1H), 7.78–7.75 (m, 3H), 7.63 (d, *J* = 8.1 Hz, 1H), 7.56 (d, *J* = 7.6 Hz, 4H), 7.46-7.38 (m, 2H), 7.20 (s, 1H), 3.73 (s, 3H), 2.72 (s, 6H), 2.43 (s, 6H) ppm; ^{13}C NMR (101 MHz; $CDCl_3$): δ = 169.6, 167.1, 150.4, 150.2, 149.8, 139.7, 137.1, 134.5, 134.4, 134.3, 132.2, 132.1, 131.9, 131.5, 129.8, 127.3, 126.5, 89.9, 89.7, 52.5, 52.0, 40.1, 21.5 ppm; IR (neat)/cm^{-1}: ṽ = 2921 (w), 1736 (w), 1666 (w), 1529 (w), 1483 (w), 1339 (w), 1298 (w), 1208 (m), 998 (s), 796 (s), 719 (m); UV-Vis ($CHCl_3$): λ_{max} (log ε) = 424 (6.80), 551 (5.41), 590 nm (4.82); HRMS(MALDI-TOF): m/z = calcd. for $C_{62}H_{45}N_5O_3Zn$ $[M]^+$ 971.2814; 971.2786 found.

2. NMR and Mass Spectrometer Spectra

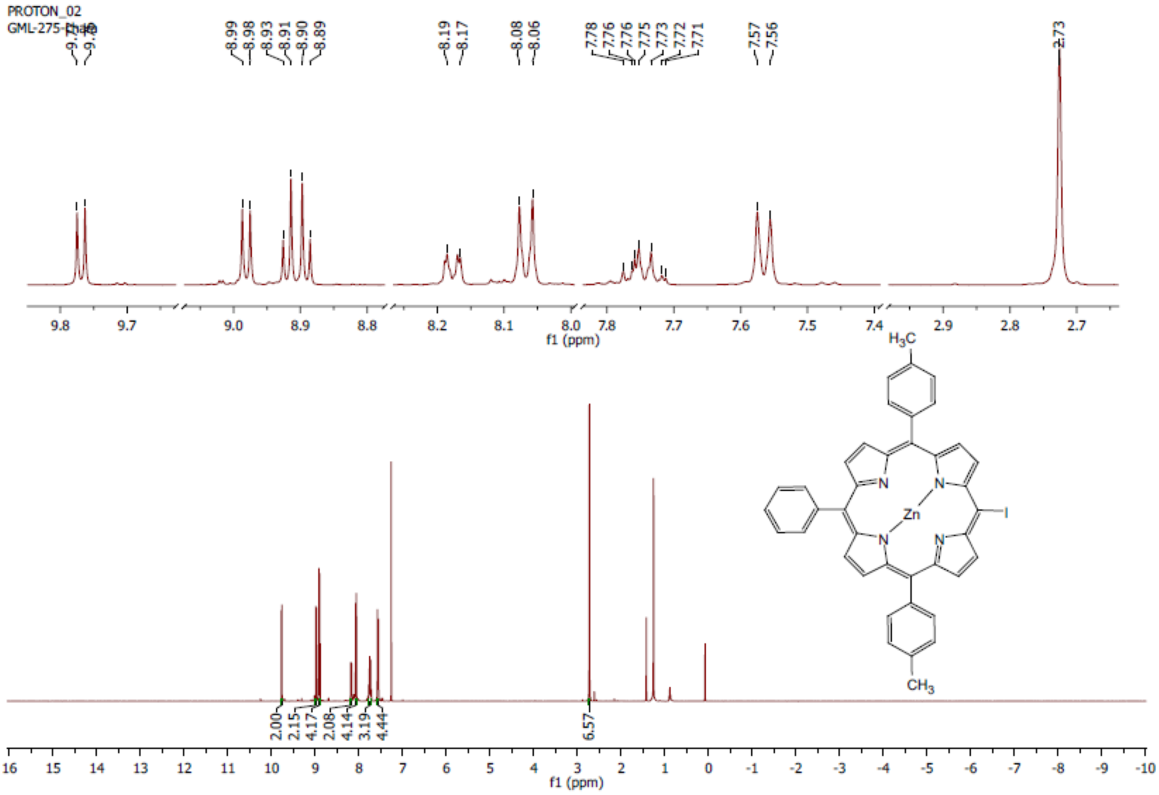

Fig. S1 ^{1}H NMR spectrum of [5-iodo-15-phenyl-10,20-bis(4′-methylphenyl)porphyrinato]zinc(II) in $CDCl_3$.

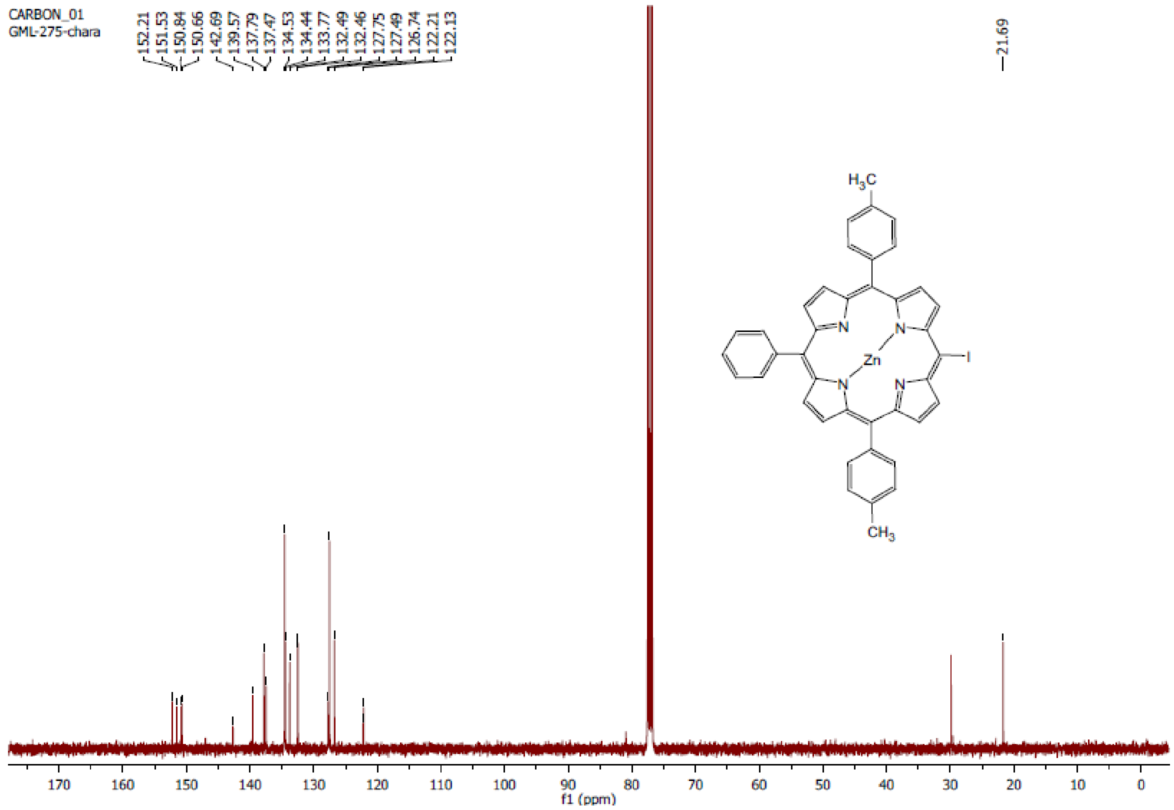

Fig. S2 ^{13}C NMR spectrum of [5-iodo-15-phenyl-10,20-bis(4′-methylphenyl)porphyrinato]zinc(II) in $CDCl_3$.

Elemental Composition Report **Page 1**

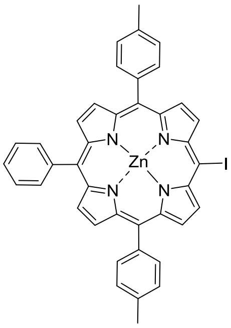

Single Mass Analysis

Tolerance = 20.0 PPM / DBE: min = -1.5, max = 400.0

Element prediction: Off

Number of isotope peaks used for i-FIT = 5

Monoisotopic Mass, Odd and Even Electron Ions

15 formula(e) evaluated with 1 results within limits (up to 10 closest results for each mass)

Elements Used:

C: 0-40 H: 0-27 N: 0-4 Zn: 0-1 I: 0-1

Gemma Locke (MSe), GML-275

Q-TOF20190520MF013 6 (0.111) AM (Cen,8, 80.00, Ht,10000.0,1570.68,0.70); Sm (SG, 2x3.00); Sb (15,10.00); Cm (6:75)

TOF MS LD+
3.48e+003

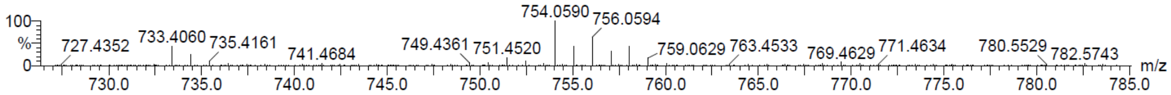

Minimum:				-1.5			
Maximum:		5.0	20.0	400.0			
Mass	Calc. Mass	mDa	PPM	DBE	i-FIT	i-FIT (Norm)	Formula
754.0590	754.0572	1.8	2.4	29.0	153.2	0.0	C40 H27 N4 Zn I

Fig. S3 MALDI-TOF-MS of [5-iodo-15-phenyl-10,20-bis(4′-methylphenyl)porphyrinato]zinc(II).

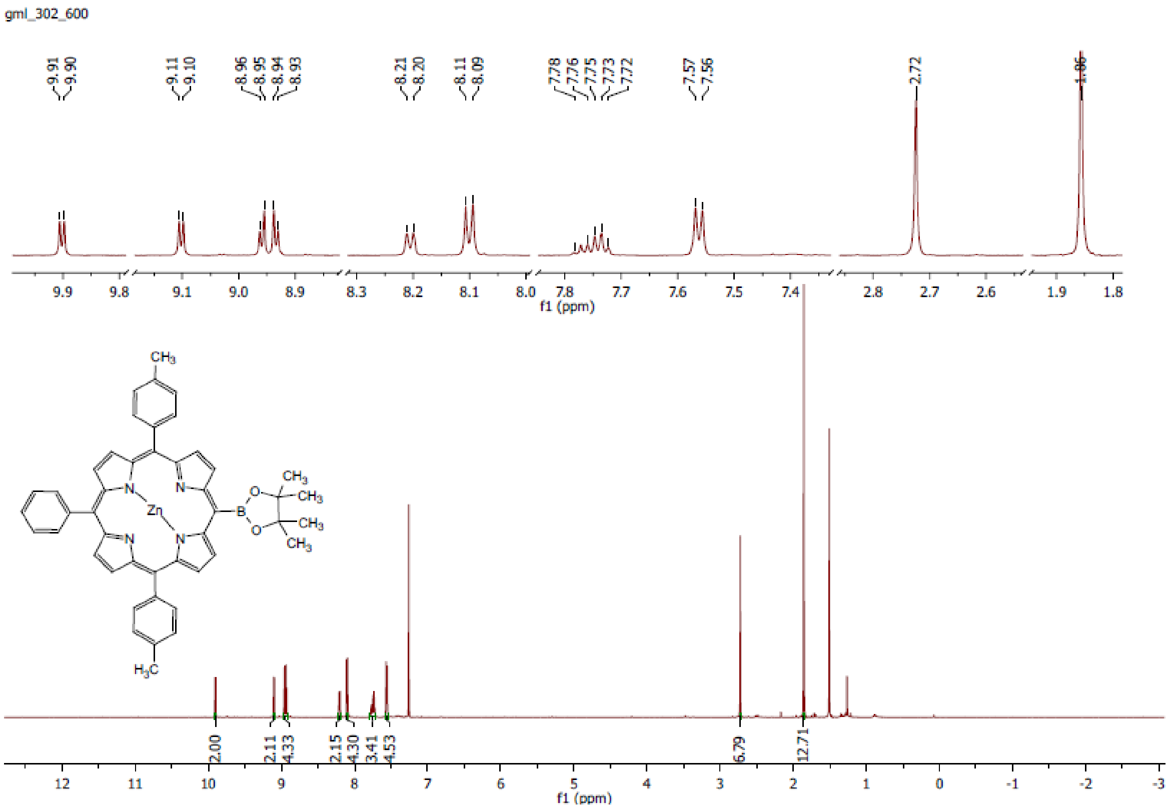

Fig. S4 ^{1}H NMR spectrum of [5-(4′,4′,5′,5′-tetramethyl-1′,3′,2′-dioxaborolan-2′-yl)-10,20-bis(4′-methylphenyl)-15-phenylporphyrinato]zinc(II) in $CDCl_3$.

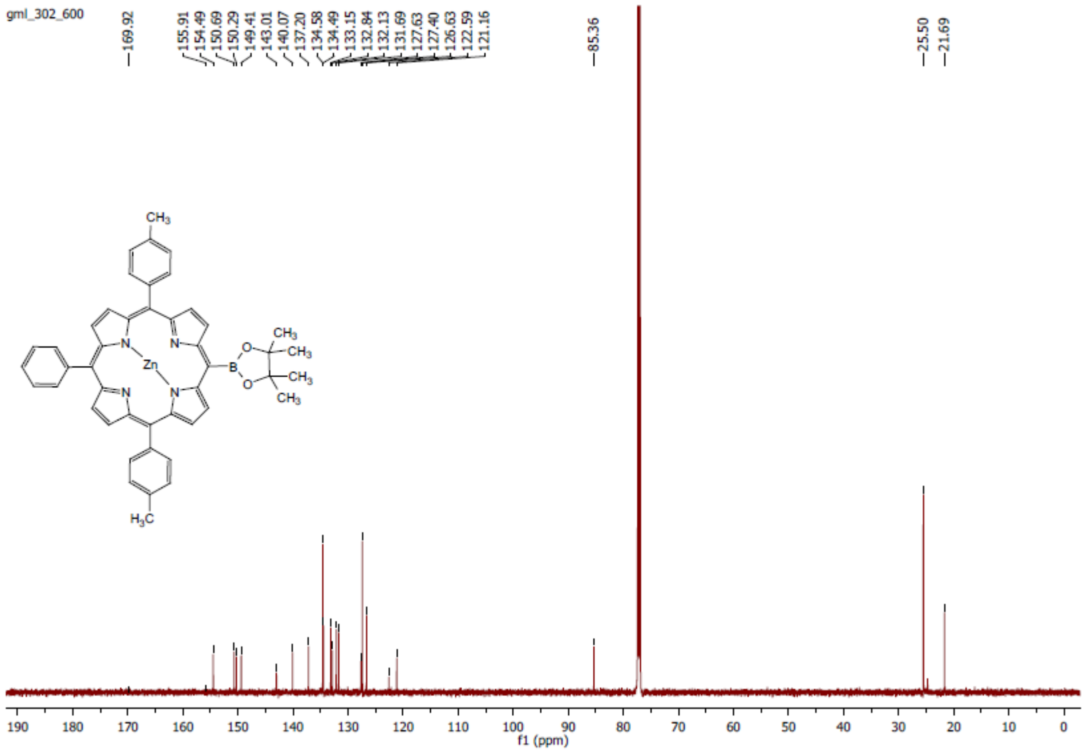

Fig. S5 ^{13}C NMR spectrum of [5-(4′,4′,5′,5′-tetramethyl-1′,3′,2′-dioxaborolan-2′-yl)-10,20-bis(4′-methylphenyl)-15-phenylporphyrinato]zinc(II) in $CDCl_3$.

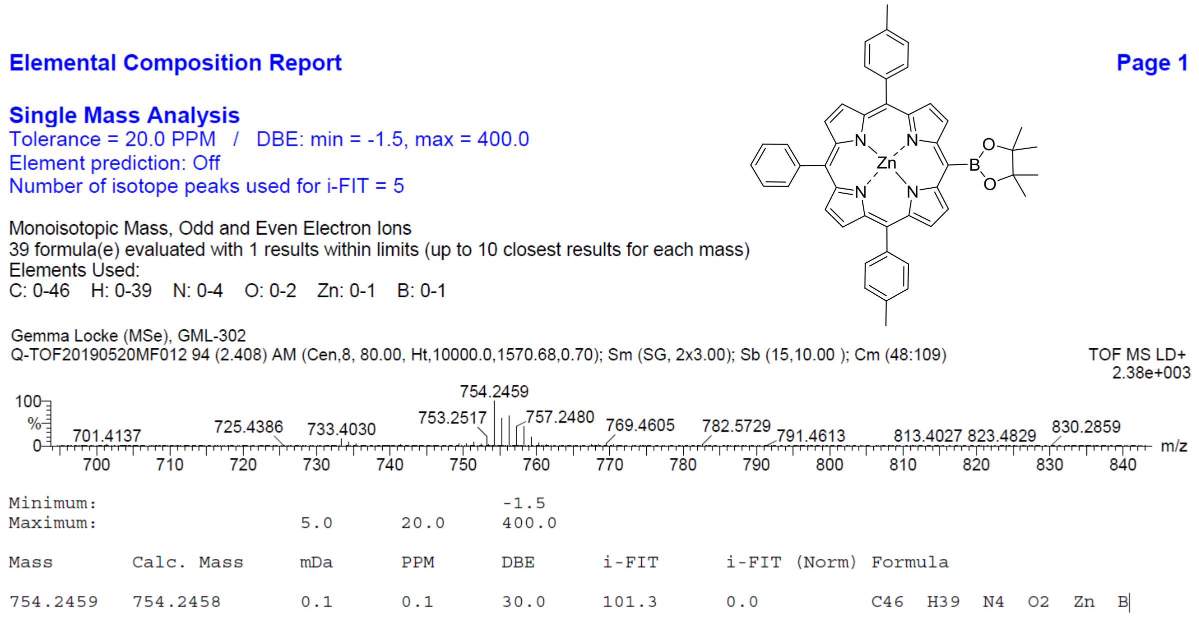

Elemental Composition Report Page 1

Single Mass Analysis
Tolerance = 20.0 PPM / DBE: min = -1.5, max = 400.0
Element prediction: Off
Number of isotope peaks used for i-FIT = 5

Monoisotopic Mass, Odd and Even Electron Ions
39 formula(e) evaluated with 1 results within limits (up to 10 closest results for each mass)
Elements Used:
C: 0-46 H: 0-39 N: 0-4 O: 0-2 Zn: 0-1 B: 0-1

Gemma Locke (MSe), GML-302
Q-TOF20190520MF012 94 (2.408) AM (Cen,8, 80.00, Ht,10000.0,1570.68,0.70); Sm (SG, 2x3.00); Sb (15,10.00); Cm (48:109) TOF MS LD+ 2.38e+003

Minimum:				-1.5			
Maximum:		5.0	20.0	400.0			
Mass	Calc. Mass	mDa	PPM	DBE	i-FIT	i-FIT (Norm)	Formula
754.2459	754.2458	0.1	0.1	30.0	101.3	0.0	C46 H39 N4 O2 Zn B

Fig. S6 MALDI-TOF-MS of [5-(4′,4′,5′,5′-tetramethyl-1′,3′,2′-dioxaborolan-2′-yl)-10,20-bis(4′-methylphenyl)-15-phenylporphyrinato]zinc(II).

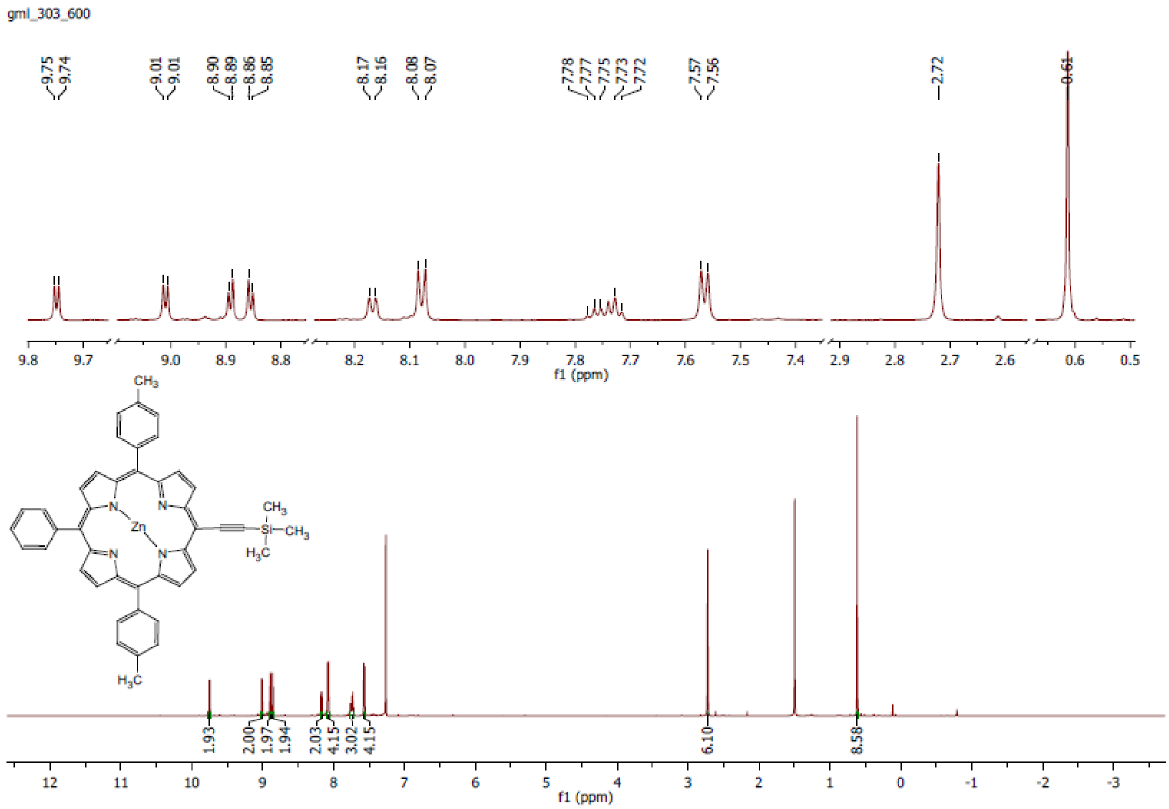

Fig. S7 ^{1}H NMR spectrum of [5-Phenyl-10,20-bis(4′-methylphenyl)-15-trimethylsilyl ethynylporphyrinato]zinc(II) in $CDCl_3$.

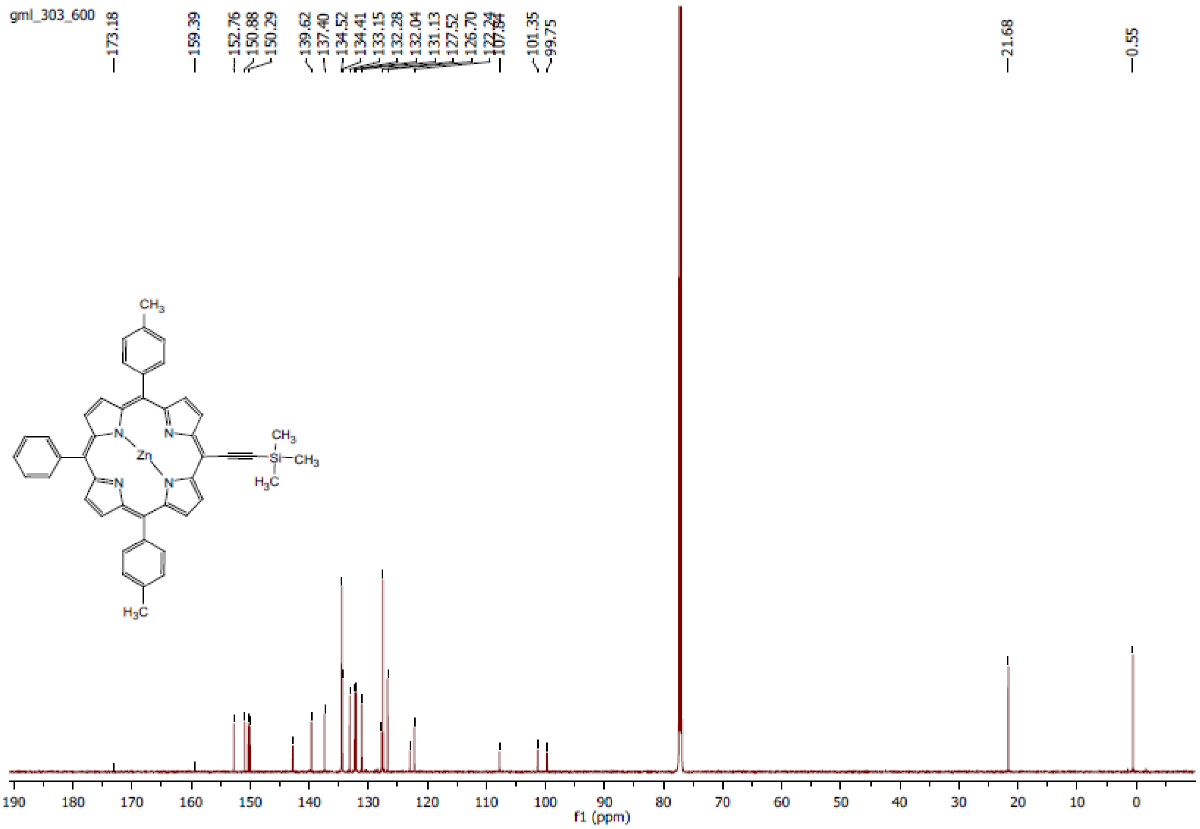

Fig. S8 ^{13}C NMR spectrum of [5-Phenyl-10,20-bis(4′-methylphenyl)-15-trimethylsilyl ethynylporphyrinato]zinc(II) in $CDCl_3$.

Elemental Composition Report **Page 1**

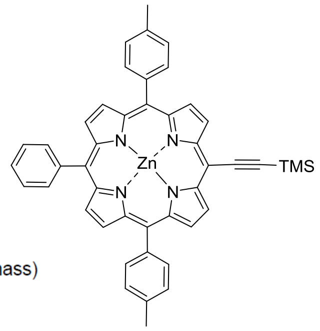

Single Mass Analysis
Tolerance = 20.0 PPM / DBE: min = -1.5, max = 400.0
Element prediction: Off
Number of isotope peaks used for i-FIT = 5

Monoisotopic Mass, Odd and Even Electron Ions
12 formula(e) evaluated with 1 results within limits (up to 10 closest results for each mass)
Elements Used:
C: 0-45 H: 0-36 N: 0-4 Si: 0-1 Zn: 0-1
Gemma Locke (MSe), GML-303
Q-TOF20190520MF010 82 (1.846) AM (Cen,8, 80.00, Ht,10000.0,1570.68,0.70); Sm (SG, 2x3.00); Sb (15,10.00); Cm (4:105) TOF MS LD+ 3.26e+003

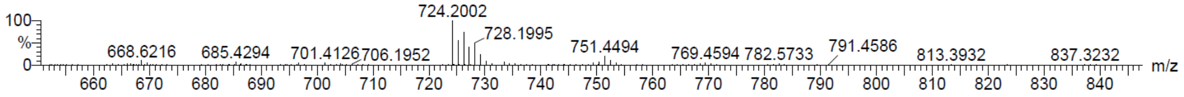

Minimum:				-1.5			
Maximum:		5.0	20.0	400.0			
Mass	Calc. Mass	mDa	PPM	DBE	i-FIT	i-FIT (Norm)	Formula
724.2002	724.2001	0.1	0.1	31.0	161.8	0.0	C45 H36 N4 Si Zn

Fig. S9 MALDI-TOF-MS of [5-Phenyl-10,20-bis(4′-methylphenyl)-15-trimethylsilyl ethynylporphyrinato]zinc(II).

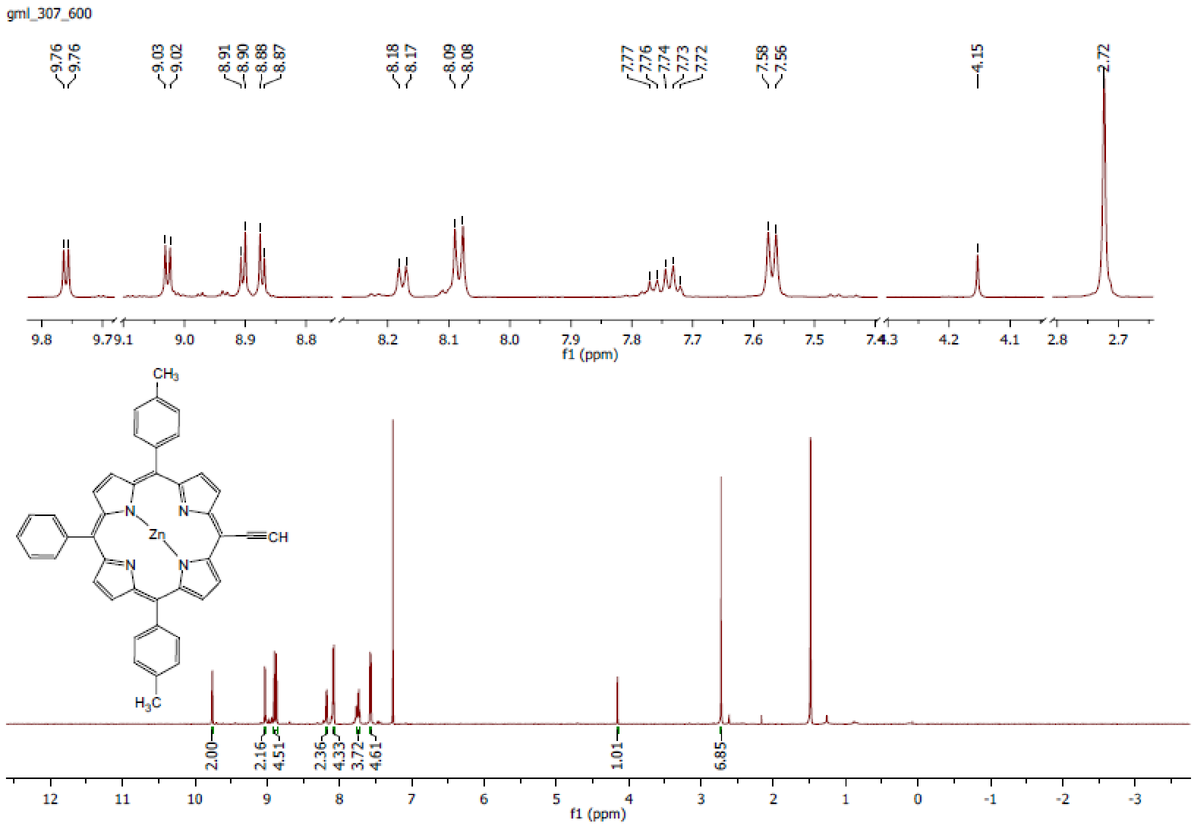

Fig. S10 ^{1}H NMR spectrum of [5-ethynyl-10,20-bis(4'-methylphenyl)-15-phenylporphyrinato]zinc(II) in $CDCl_3$.

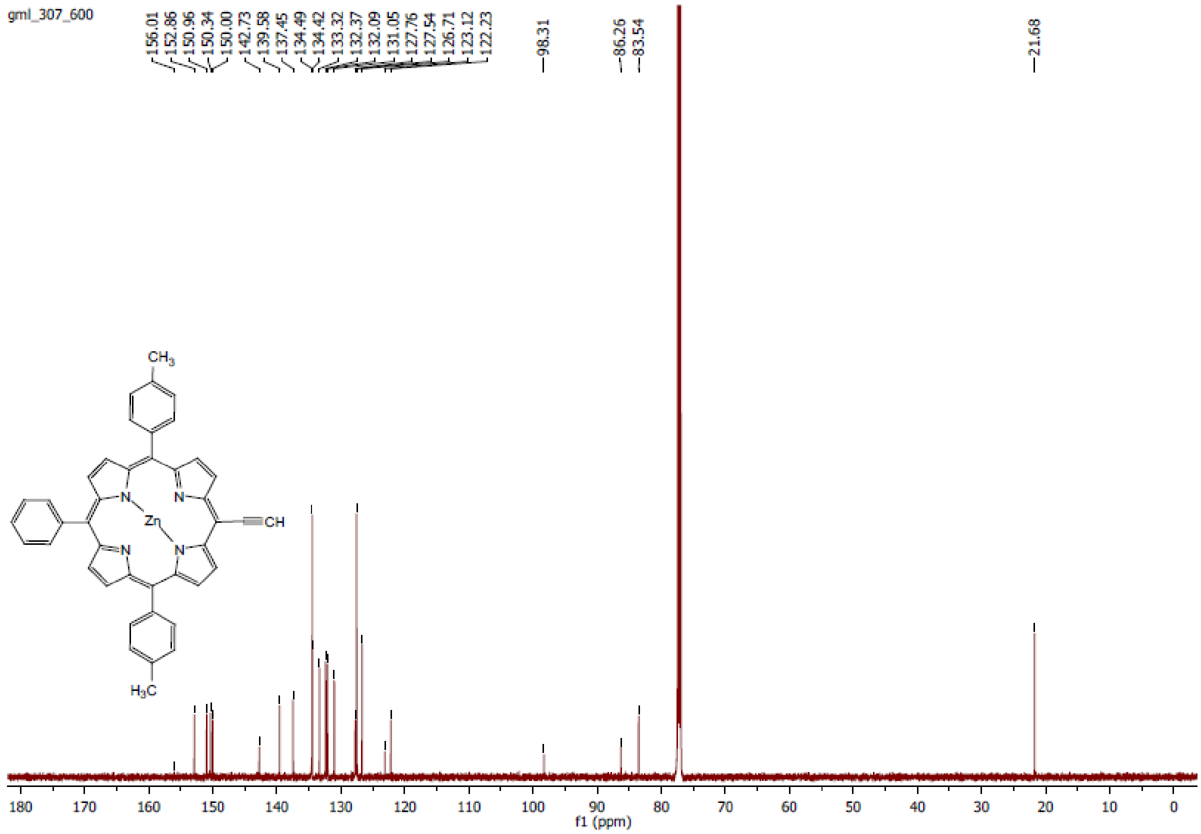

Fig. S11 ^{13}C NMR spectrum of [5-ethynyl-10,20-bis(4'-methylphenyl)-15-phenylporphyrinato]zinc(II) in $CDCl_3$.

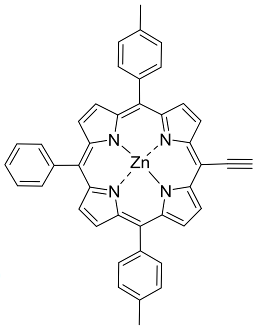

Elemental Composition Report | **Page 1**

Single Mass Analysis
Tolerance = 20.0 PPM / DBE: min = -1.5, max = 400.0
Element prediction: Off
Number of isotope peaks used for i-FIT = 5

Monoisotopic Mass, Odd and Even Electron Ions
5 formula(e) evaluated with 1 results within limits (up to 10 closest results for each mass)
Elements Used:
C: 0-42 H: 0-29 N: 0-4 Zn: 0-1

Gemma Locke (MSe), GML-307-chara
Q-TOF20190607MF007 82 (1.519) AM (Cen,8, 80.00, Ht,10000.0,1570.68,0.70); Sm (SG, 2x3.00); Sb (15,10.00); Cm (5:97-80:85) TOF MS LD+ 3.32e+003

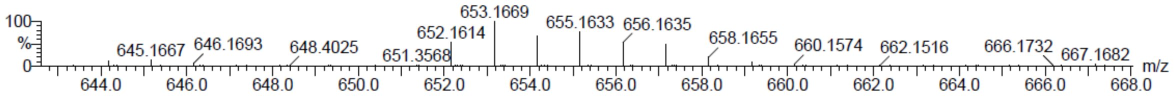

Minimum:				-1.5			
Maximum:		5.0	20.0	400.0			
Mass	Calc. Mass	mDa	PPM	DBE	i-FIT	i-FIT (Norm)	Formula
653.1669	653.1684	-1.5	-2.3	30.5	162.2	0.0	C42 H29 N4 Zn

Fig. S12 MALDI-TOF-MS of [5-ethynyl-10,20-bis(4'-methylphenyl)-15-phenylporphyrinato]zinc(II).

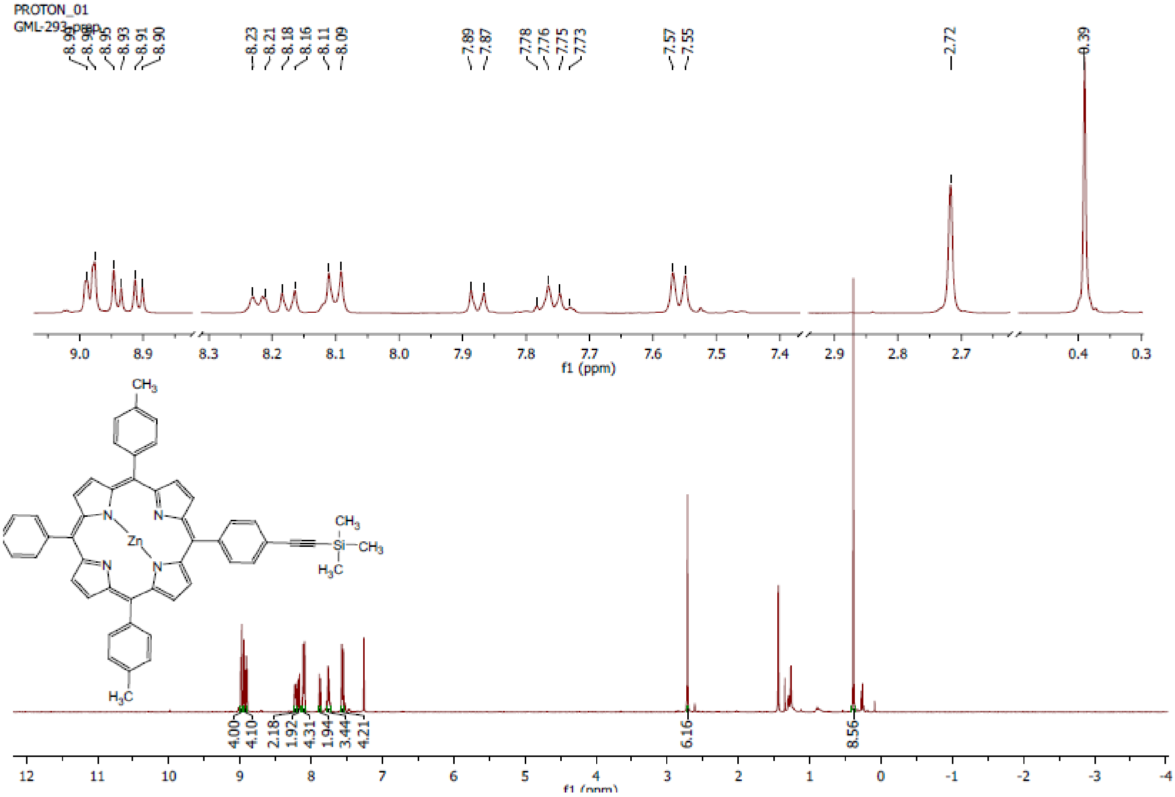

Fig. S13 ^{1}H NMR spectrum of [5-phenyl-10,20-bis(4'-methylphenyl)-15-(4'-trimethylsilyl ethynylphenyl)porphyrinato]zinc(II) in $CDCl_3$.

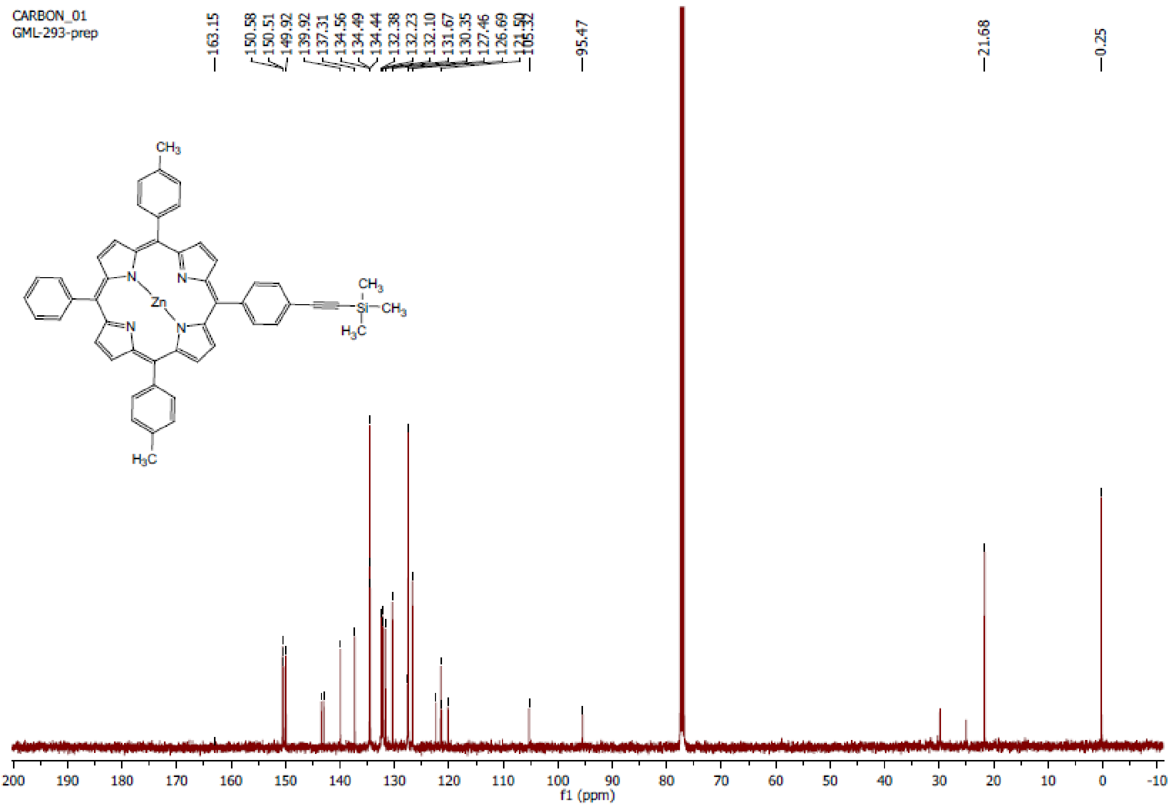

Fig. S14 ^{13}C NMR spectrum of [5-phenyl-10,20-bis(4′-methylphenyl)-15-(4′-trimethylsilyl ethynylphenyl)porphyrinato]zinc(II) in $CDCl_3$.

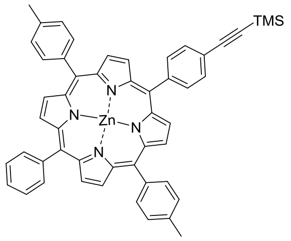

Elemental Composition Report **Page 1**

Single Mass Analysis
Tolerance = 100.0 PPM / DBE: min = -1.5, max = 400.0
Element prediction: Off
Number of isotope peaks used for i-FIT = 5

Monoisotopic Mass, Odd and Even Electron Ions
10 formula(e) evaluated with 1 results within limits (up to 10 best isotopic matches for each mass)
Elements Used:
C: 0-51 H: 0-40 N: 0-4 Si: 0-1 Zn: 0-1
Gemma Locke (MSe), GML-293
Q-TOF20180914MF007 35 (0.648) AM (Top,6, Ht,10000.0,1570.68,0.70); Sm (SG, 2x3.00); Sb (15,10.00); Cm (6:75-(52+68)) TOF MS LD+ 1.05e+003

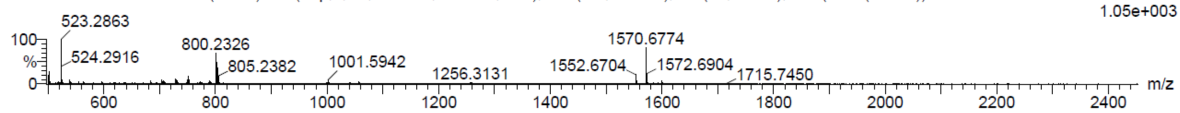

Minimum:				-1.5			
Maximum:		5.0	100.0	400.0			
Mass	Calc. Mass	mDa	PPM	DBE	i-FIT	i-FIT (Norm)	Formula
800.2326	800.2314	1.2	1.5	35.0	85.7	0.0	C51 H40 N4 Si Zn

Fig. S15 MALDI-TOF-MS of [5-phenyl-10,20-bis(4′-methylphenyl)-15-(4′-trimethylsilyl ethynylphenyl)porphyrinato]zinc(II)).

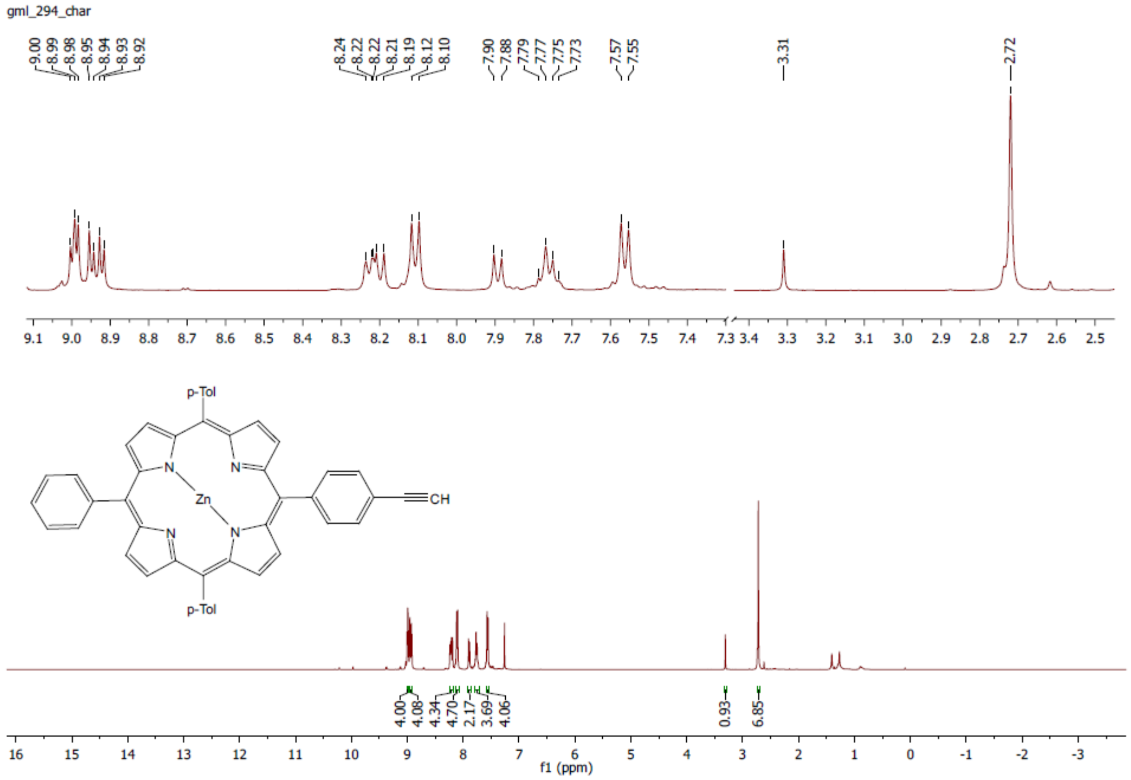

Fig. S16 ^{1}H NMR spectrum of 5-(4′-ethynylphenyl)-10,20-bis(4′-methylphenyl)-15-phenylporphyrinato]zinc(II) in $CDCl_3$.

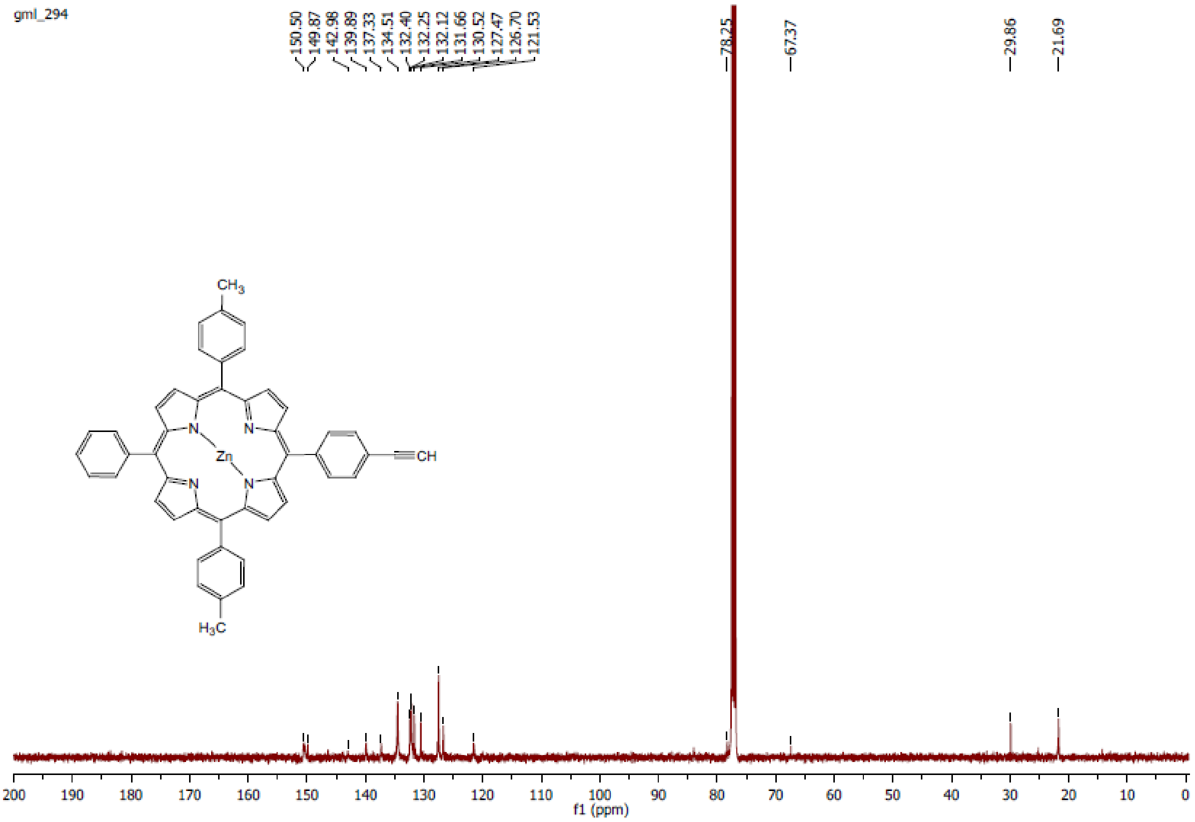

Fig. S17 ^{13}C NMR spectrum of 5-(4′-ethynylphenyl)-10,20-bis(4′-methylphenyl)-15-phenylporphyrinato]zinc(II) in $CDCl_3$.

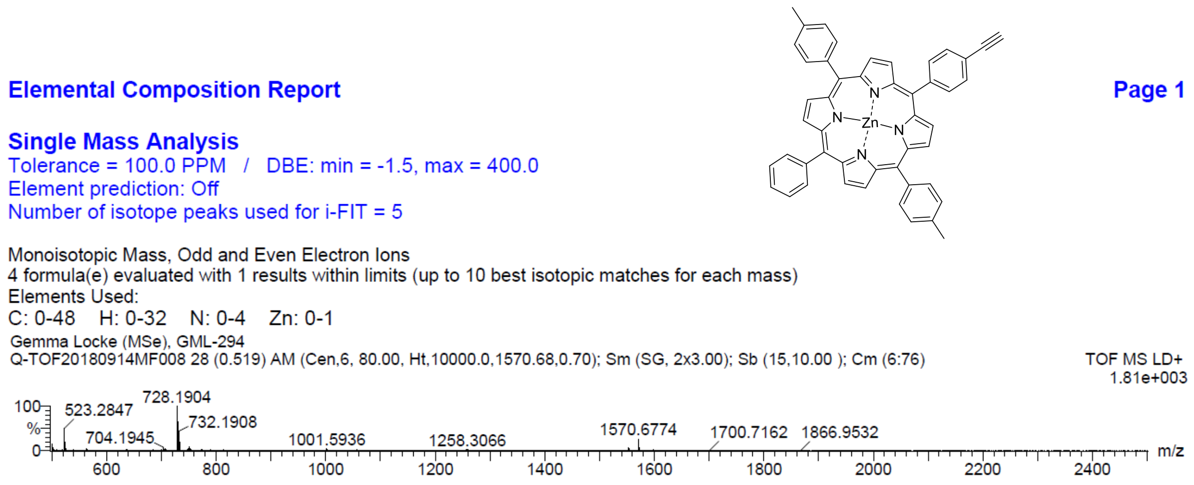

Minimum:				-1.5			
Maximum:		5.0	100.0	400.0			
Mass	Calc. Mass	mDa	PPM	DBE	i-FIT	i-FIT (Norm)	Formula
728.1904	728.1918	-1.4	-1.9	35.0	115.8	0.0	C48 H32 N4 Zn

Fig. S18 MALDI-TOF-MS of 5-(4′-Ethynylphenyl)-10,20-bis(4′-methylphenyl)-15-phenylporphyrinato]zinc(II).

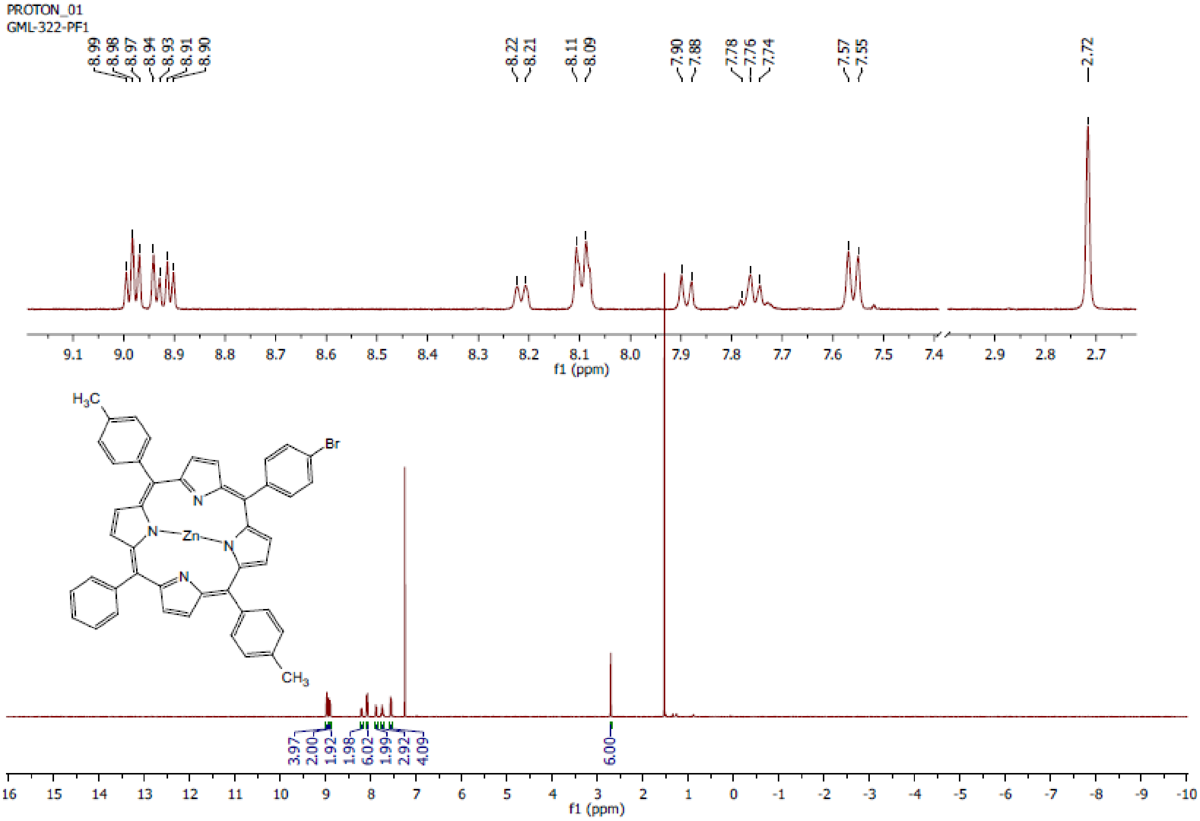

Fig. S19 ^{1}H NMR spectrum of 5-[(4′-bromophenyl)-15-phenyl-10,20-bis-(4′-methylphenyl)porphyrinato]zinc(II) in $CDCl_3$.

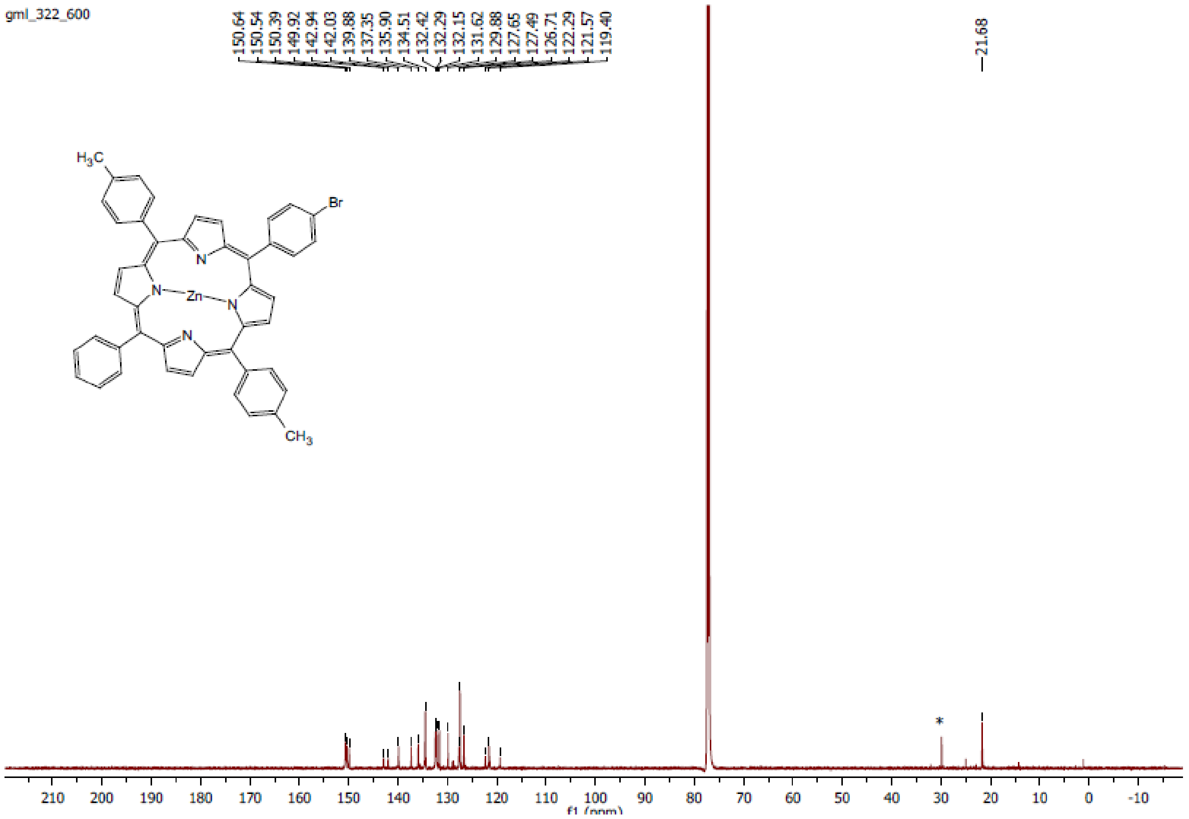

Fig. S20 ^{13}C NMR spectrum of 5-[(4'-bromophenyl)-15-phenyl-10,20-bis-(4'-methylphenyl)porphyrinato]zinc(II) in $CDCl_3$.

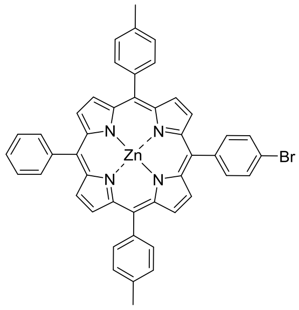

Elemental Composition Report — **Page 1**

Single Mass Analysis
Tolerance = 100.0 PPM / DBE: min = -1.5, max = 400.0
Element prediction: Off
Number of isotope peaks used for i-FIT = 5

Monoisotopic Mass, Odd and Even Electron Ions
15 formula(e) evaluated with 1 results within limits (up to 10 closest results for each mass)
Elements Used:
C: 0-46 H: 0-31 N: 0-4 Br: 0-1 Zn: 0-1

Gemma Locke (MSe), GML-322
Q-TOF2019015MF009 61 (1.130) AM (Cen,8, 80.00, Ht,10000.0,1570.68,0.70); Sm (SG, 2x3.00); Sb (15,10.00); Cm (11:79) TOF MS LD+ 4.15e+003

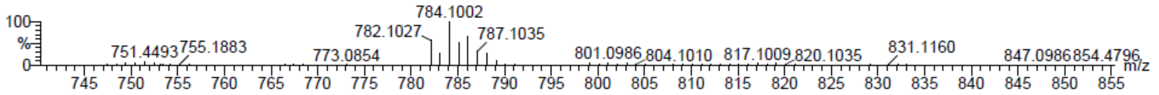

Minimum:				-1.5			
Maximum:		5.0	100.0	400.0			
Mass	Calc. Mass	mDa	PPM	DBE	i-FIT	i-FIT (Norm)	Formula
782.1027	782.1024	0.3	0.4	33.0	144.7	0.0	C46 H31 N4 Br Zn

Fig. S21 MALDI-TOF-MS of [5- (4'-bromophenyl)-15-phenyl-10,20-bis-(4'-methylphenyl)porphyrinato]zinc(II).

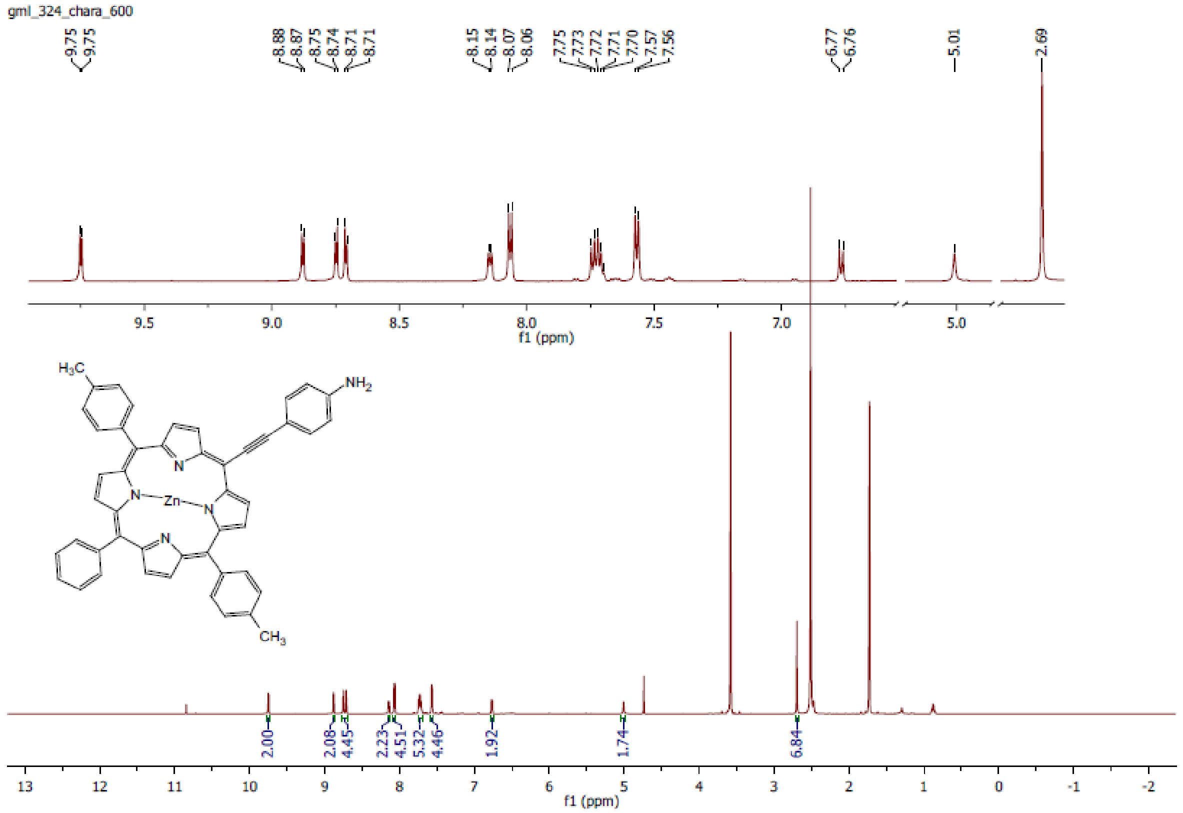

Fig. S22 ^1H NMR spectrum of [5-(4'-aminophenylacetylene)-10,20-bis(4'-methylphenyl)-15-phenylporphyrinato]zinc(II) (**9**) in THF-d_8.

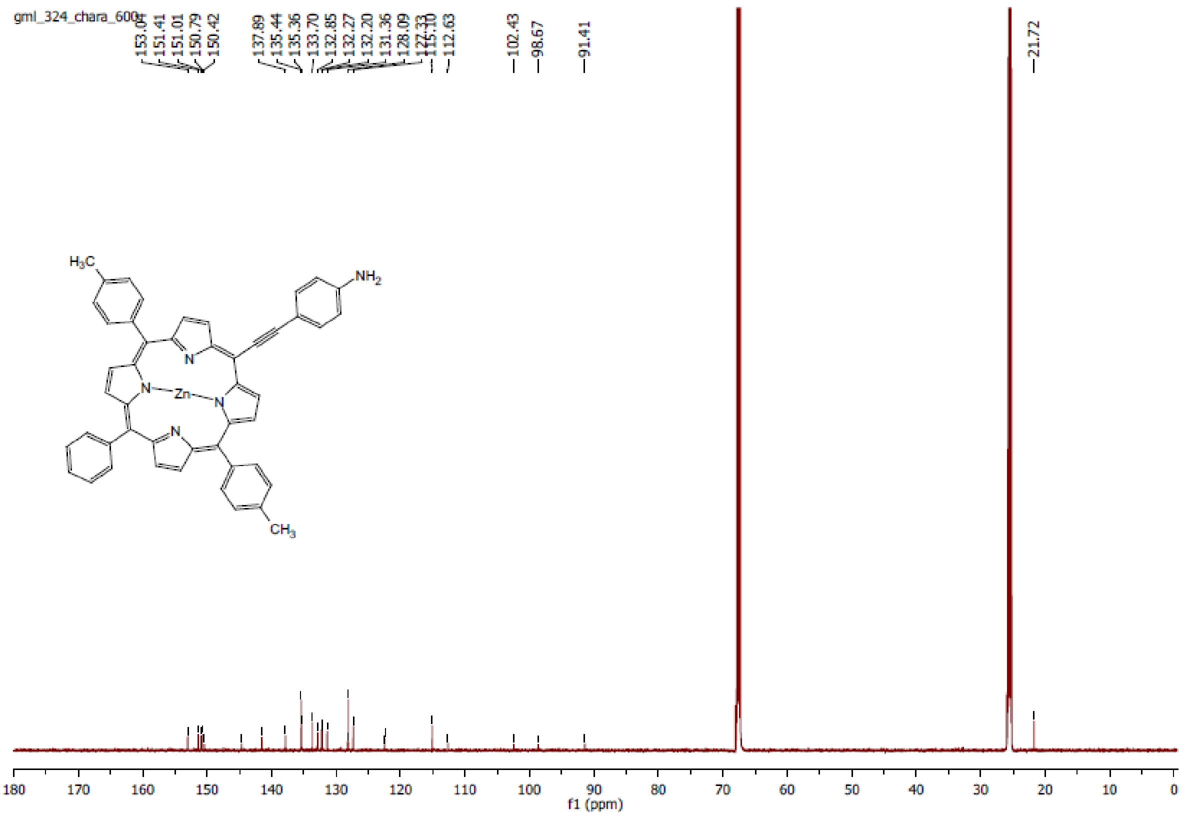

Fig. S23 ^{13}C NMR spectrum of [5-(4'-aminophenylacetylene)-10,20-bis(4'-methylphenyl)-15-phenylporphyrinato]zinc(II) (**9**) in THF-d_8.

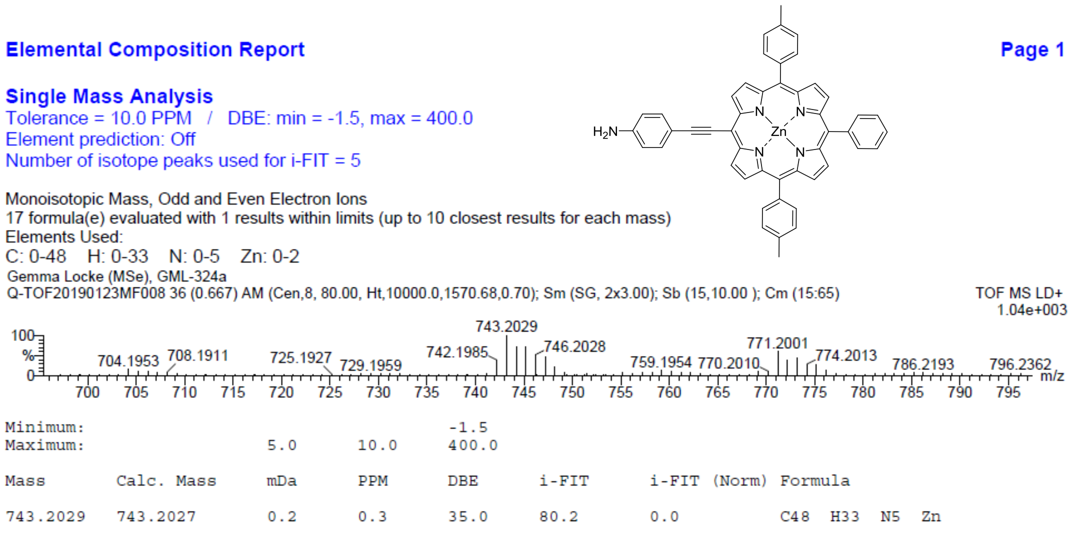

Fig. S24 MALDI-TOF-MS of [5-(4'-aminophenylacetylene)-10,20-bis(4'-methylphenyl)-15-phenylporphyrinato]zinc(II) (**9**).

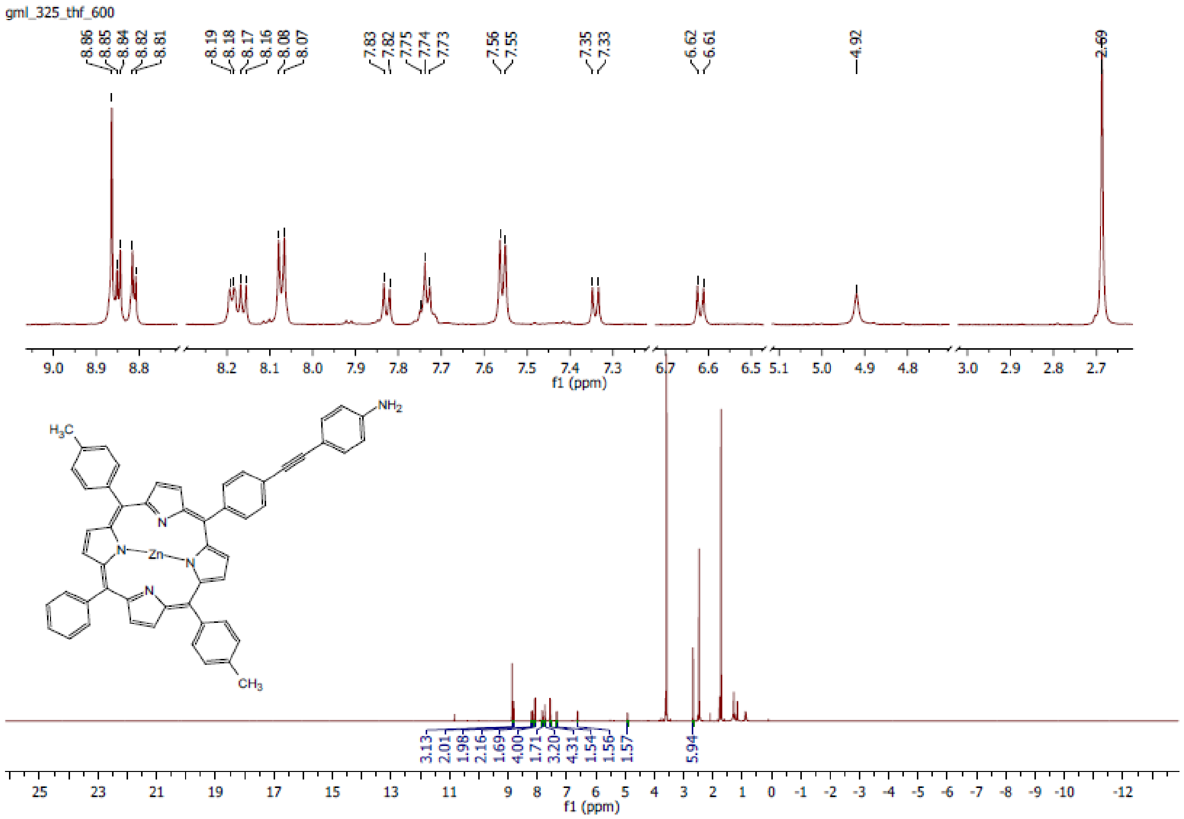

Fig. S25 ^{1}H NMR spectrum of [5-(4'-(4''-ethynylaniline)phenyl)-10,20-bis(4'-methylphenyl)-15-phenylporphyrinato]zinc(II) (**10**) in THF-d_8.

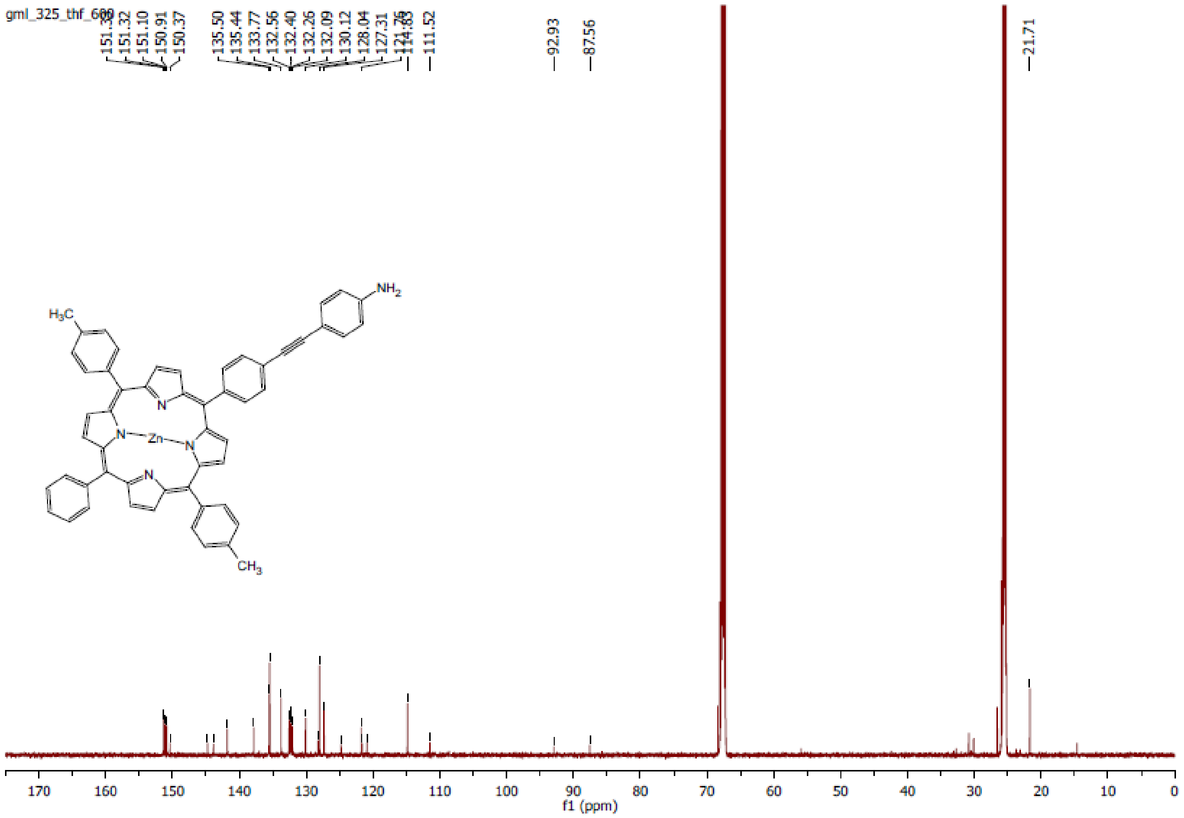

Fig. S26 ^{13}C NMR of [5-(4'-(4"-ethynylaniline)phenyl)-10,20-bis(4'-methylphenyl)-15-phenylporphyrinato]zinc(II) (**10**) in THF-d_8.

Elemental Composition Report **Page 1**

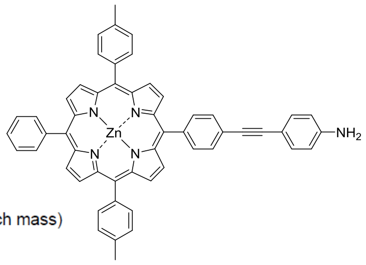

Single Mass Analysis
Tolerance = 100.0 PPM / DBE: min = -1.5, max = 400.0
Element prediction: Off
Number of isotope peaks used for i-FIT = 5

Monoisotopic Mass, Odd and Even Electron Ions
5 formula(e) evaluated with 1 results within limits (up to 10 closest results for each mass)
Elements Used:
C: 0-54 H: 0-37 N: 0-5 Zn: 0-1

Gemma Locke (MSe), GML-325
Q-TOF2019018MF002 48 (0.889) AM (Cen,8, 80.00, Ht,10000.0,1570.68,0.70); Sm (SG, 2x3.00); Sb (15,10.00); Cm (14:74) TOF MS LD+ 9.21e+002

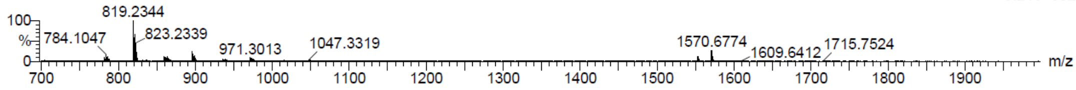

Minimum:				-1.5			
Maximum:		5.0	100.0	400.0			
Mass	Calc. Mass	mDa	PPM	DBE	i-FIT	i-FIT (Norm)	Formula
819.2344	819.2340	0.4	0.5	39.0	40.5	0.0	C54 H37 N5 Zn

Fig. S27 MALDI-TOF-MS of [5-(4'-(4"-ethynylaniline)phenyl)-10,20-bis(4'-methylphenyl)-15-phenylporphyrinato]zinc(II) (**10**).

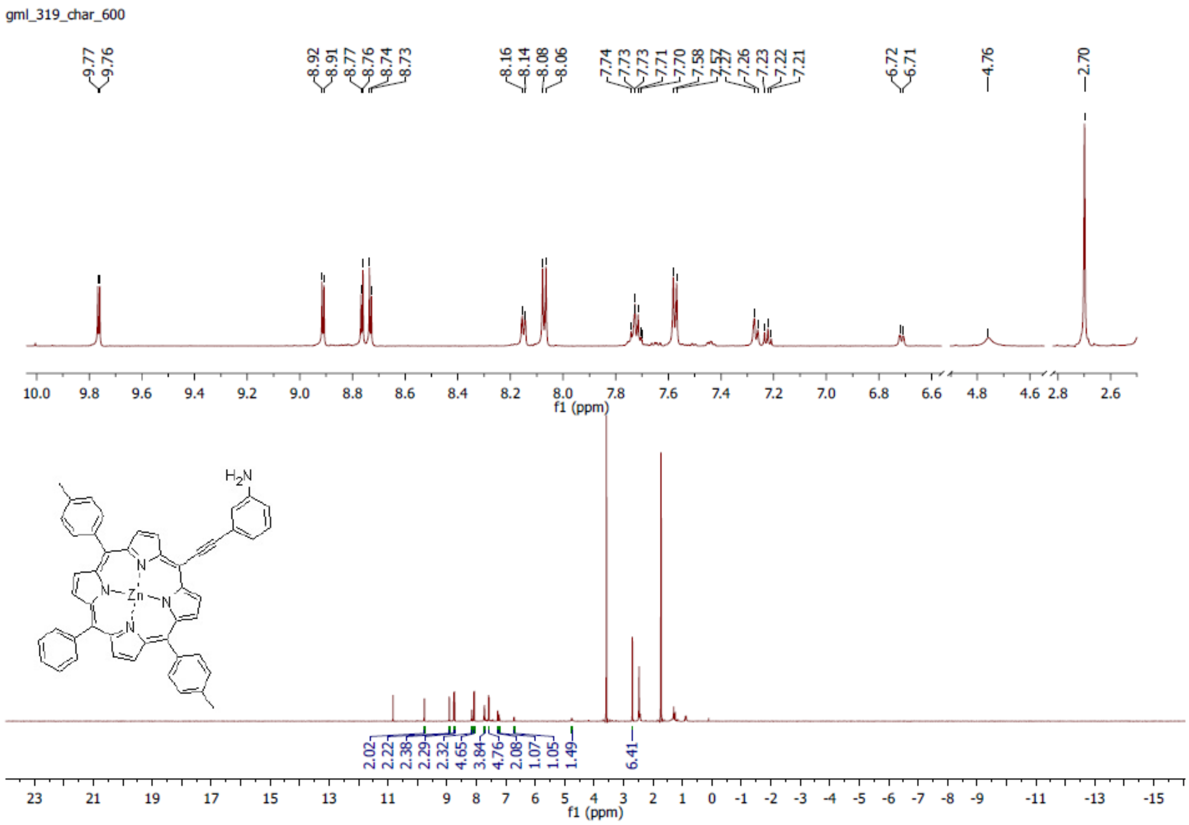

Fig. S28 ^1H NMR spectrum of [5-(3'-aminophenylacetylene)-10,20-bis(4'-methylphenyl)-15-phenylporphyrinato]zinc(II) (**11**) in THF-d_8.

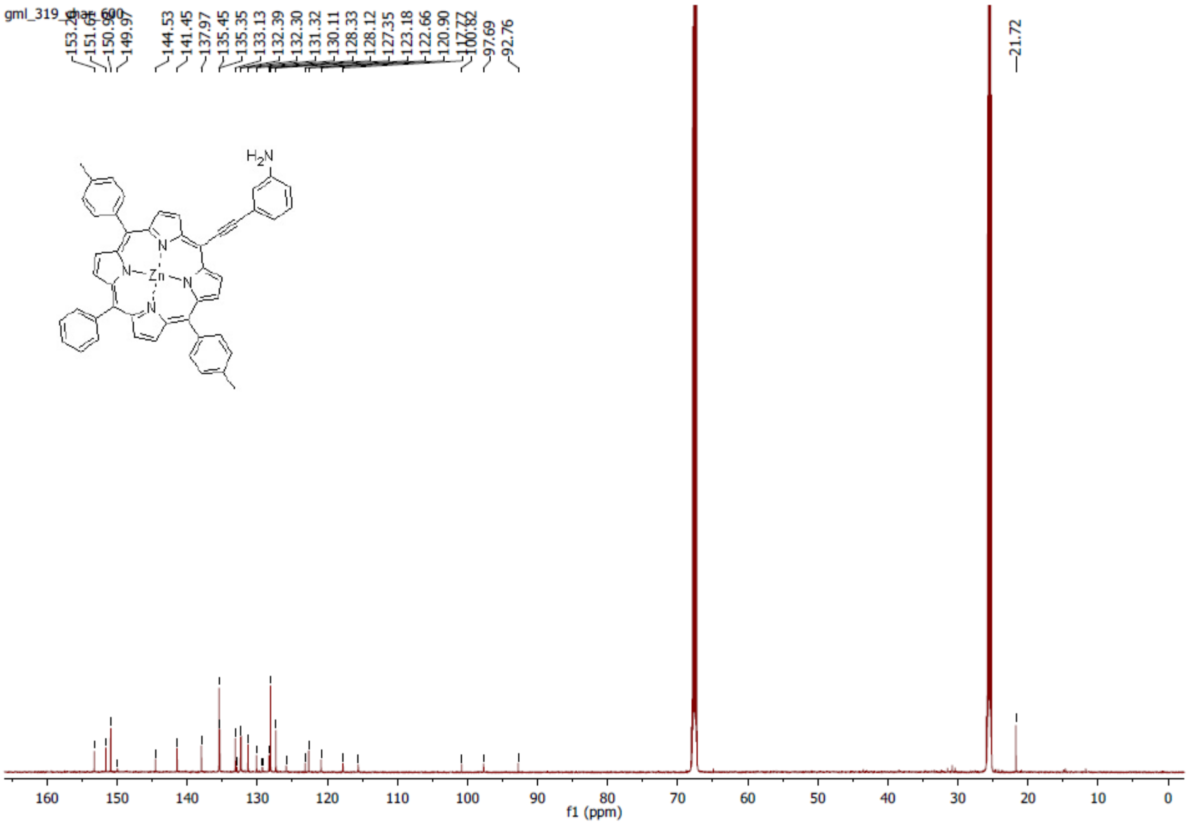

Fig. S29 ^{13}C NMR spectrum of [5-(3'-aminophenylacetylene)-10,20-bis(4'-methylphenyl)-15-phenylporphyrinato]zinc(II) (**11**) in THF-d_8.

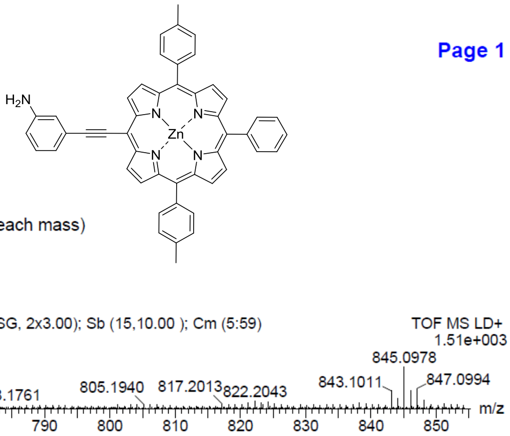

Elemental Composition Report Page 1

Single Mass Analysis
Tolerance = 100.0 PPM / DBE: min = -1.5, max = 400.0
Element prediction: Off
Number of isotope peaks used for i-FIT = 5

Monoisotopic Mass, Odd and Even Electron Ions
5 formula(e) evaluated with 1 results within limits (up to 10 closest results for each mass)
Elements Used:
C: 0-48 H: 0-33 N: 0-5 Zn: 0-1

Gemma Locke (MSe), GML-319
Q-TOF20190109MF007 30 (0.556) AM (Cen,8, 80.00, Ht,10000.0,1570.68,0.70); Sm (SG, 2x3.00); Sb (15,10.00); Cm (5:59) TOF MS LD+ 1.51e+003

Minimum:				-1.5			
Maximum:		5.0	100.0	400.0			
Mass	Calc. Mass	mDa	PPM	DBE	i-FIT	i-FIT (Norm)	Formula
743.2024	743.2027	-0.3	-0.4	35.0	119.8	0.0	C48 H33 N5 Zn

Fig. S30 MALDI-TOF-MS of [5-(3'-aminophenylacetylene)-10,20-bis(4'-methylphenyl)-15-phenylporphyrinato]zinc(II) (**11**).

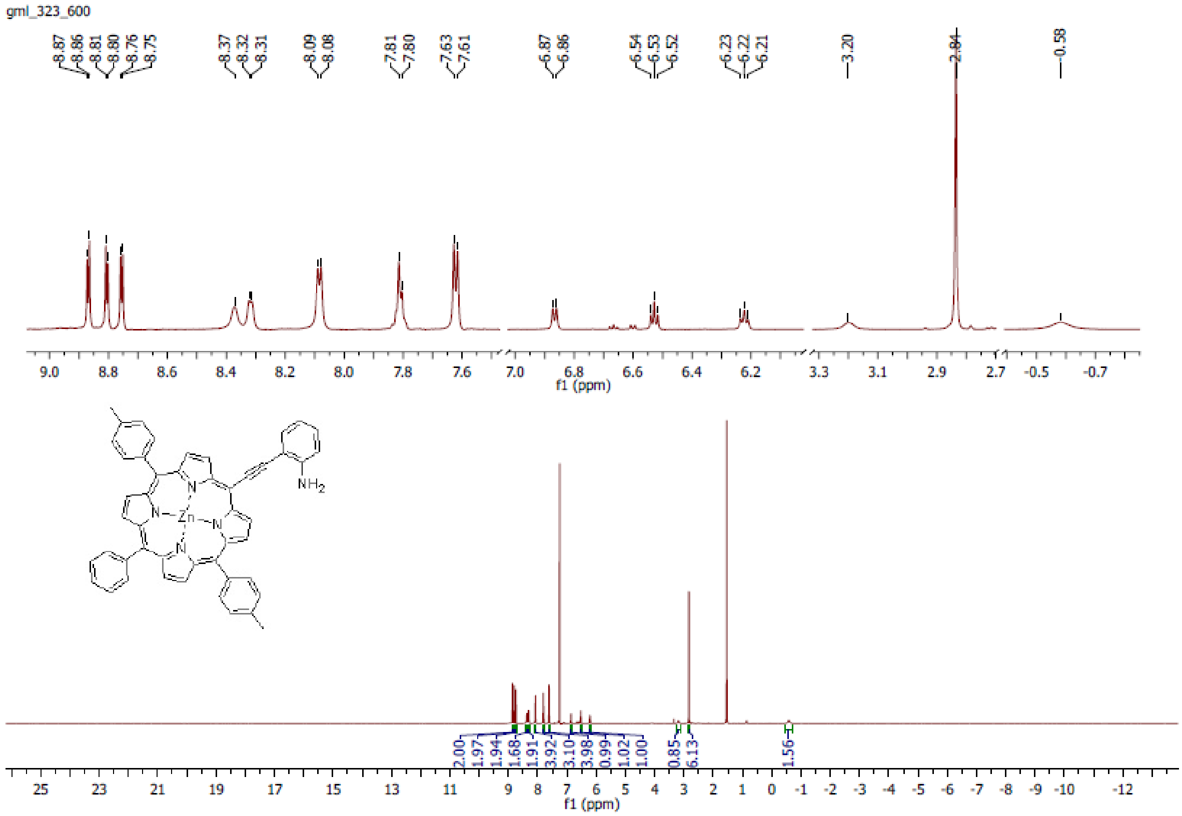

Fig. S31 ^{1}H NMR spectrum of [5-(2'-aminophenylacetylene)-10,20-bis(4'-methylphenyl)-15-phenylporphyrinato]zinc(II) (**12**) in $CDCl_3$.

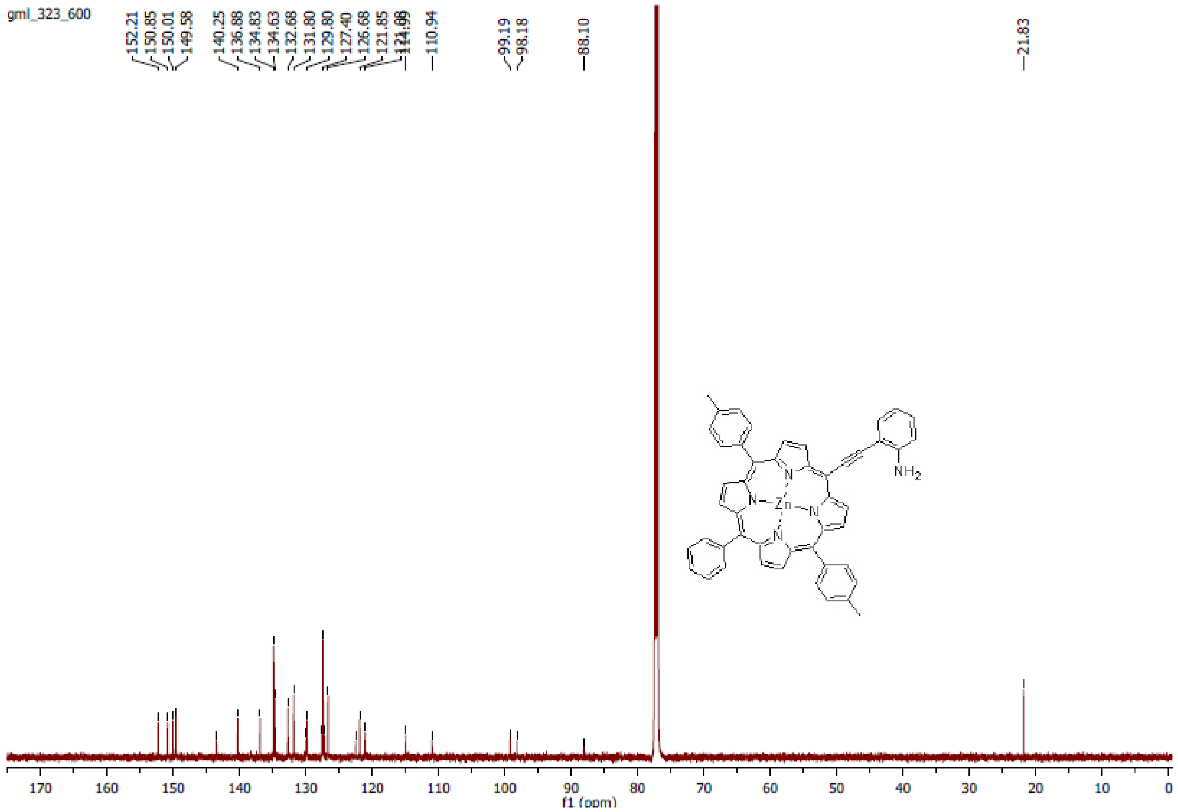

Fig. S32 ^{13}C NMR spectrum of [5-(2'-aminophenylacetylene)-10,20-bis(4'-methylphenyl)-15-phenylporphyrinato]zinc(II) (**12**) in $CDCl_3$.

Elemental Composition Report **Page 1**

Single Mass Analysis
Tolerance = 100.0 PPM / DBE: min = -1.5, max = 400.0
Element prediction: Off
Number of isotope peaks used for i-FIT = 5

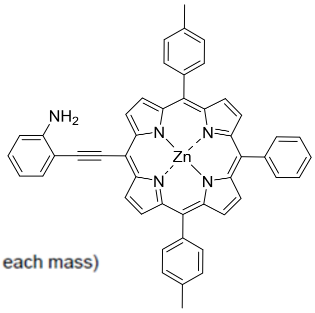

Monoisotopic Mass, Odd and Even Electron Ions
5 formula(e) evaluated with 1 results within limits (up to 10 closest results for each mass)
Elements Used:
C: 0-48 H: 0-33 N: 0-5 Zn: 0-1
Gemma Locke (MSe), GML-743
Q-TOF2019015MF010 31 (0.574) AM (Cen,8, 80.00, Ht,10000.0,1570.68,0.70); Sm (SG, 2x3.00); Sb (15,10.00); Cm (7:75-47:49) TOF MS LD+
2.66e+003

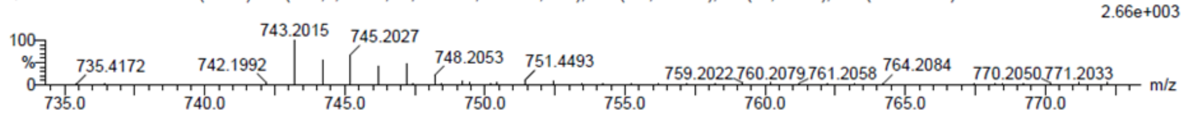

Minimum:				-1.5			
Maximum:		5.0	100.0	400.0			
Mass	Calc. Mass	mDa	PPM	DBE	i-FIT	i-FIT (Norm)	Formula
743.2015	743.2027	-1.2	-1.6	35.0	99.4	0.0	C48 H33 N5 Zn

Fig. S33 MALDI-TOF-MS of [5-(2'-aminophenylacetylene)-10,20-bis(4'-methylphenyl)-15-phenylporphyrinato]zinc(II) (**12**).

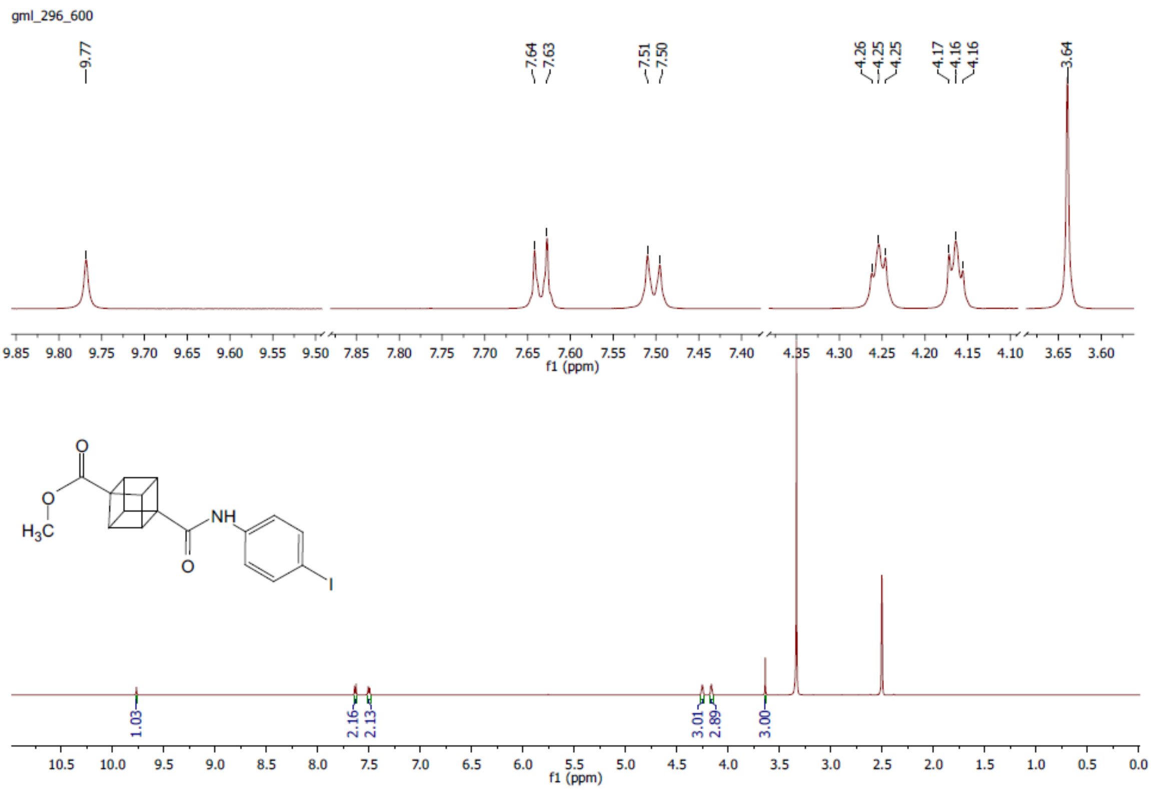

Fig. S34 ^{1}H NMR spectrum of cubane **13** in DMSO-d_6.

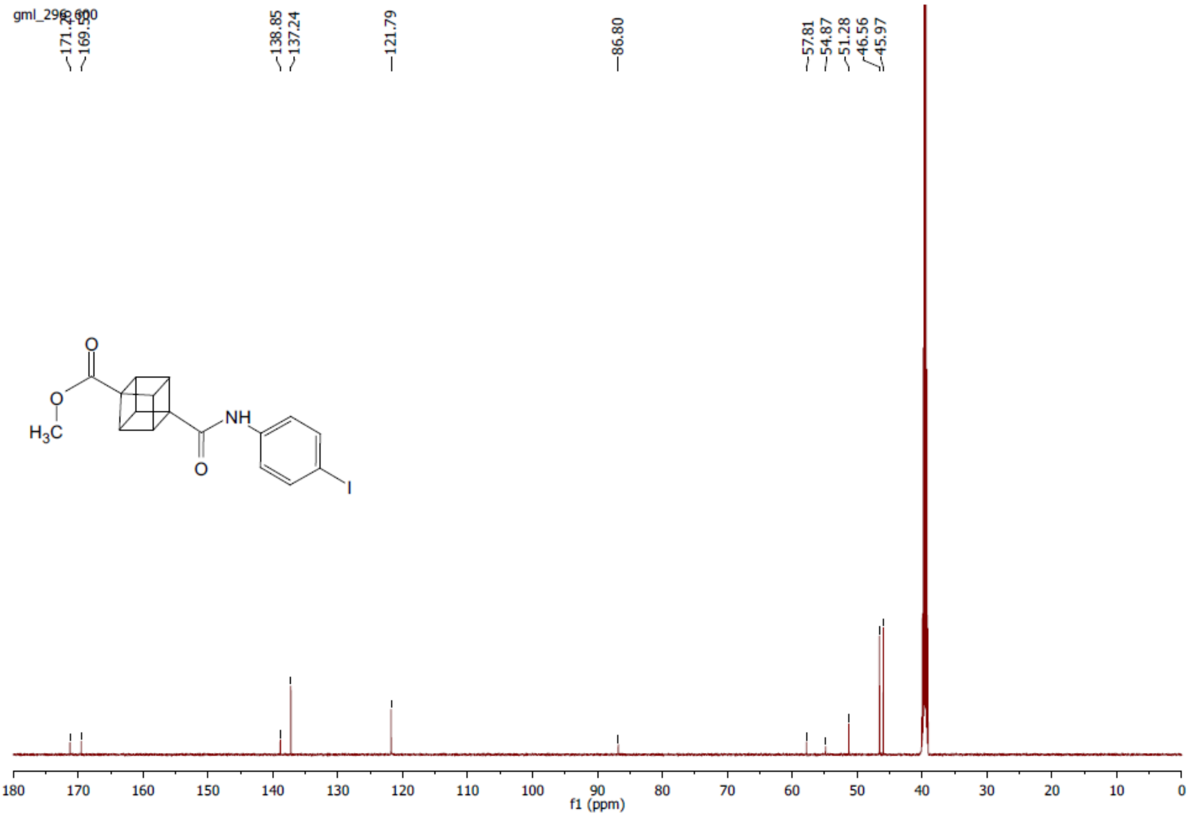

Fig. S35 ^{13}C NMR spectrum of cubane **13** in DMSO-d_6.

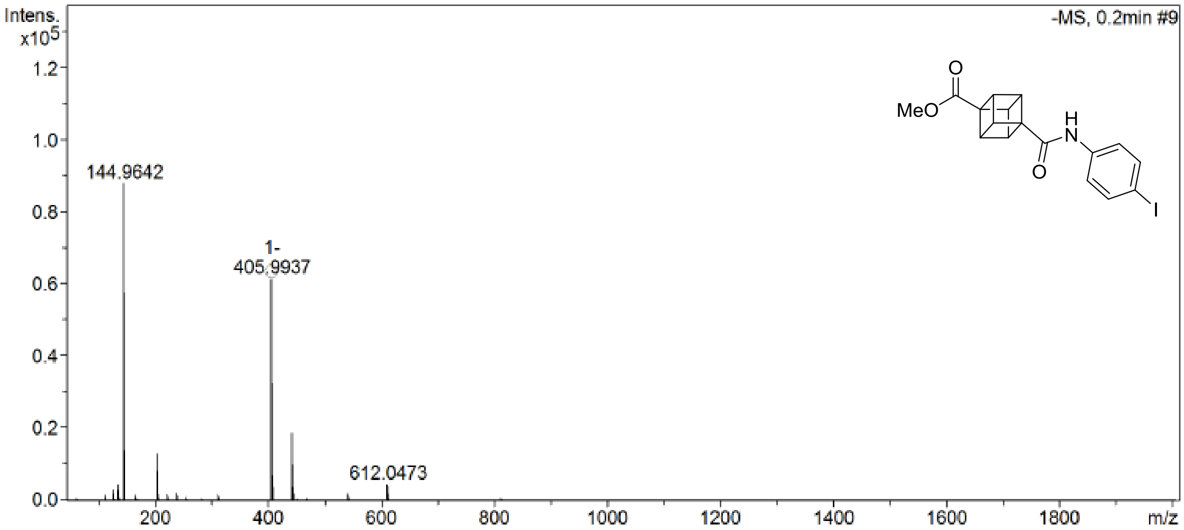

Meas. m/z	#	Ion Formula	m/z	err [mDa]	err [ppm]	rdb	N-Rule	e⁻ Conf	mSigma
405.993716	1	C17H13INO3	405.994565	0.8	2.1	11.5	ok	even	46.5

Fig. S36 HRMS (ESI) of cubane **13**.

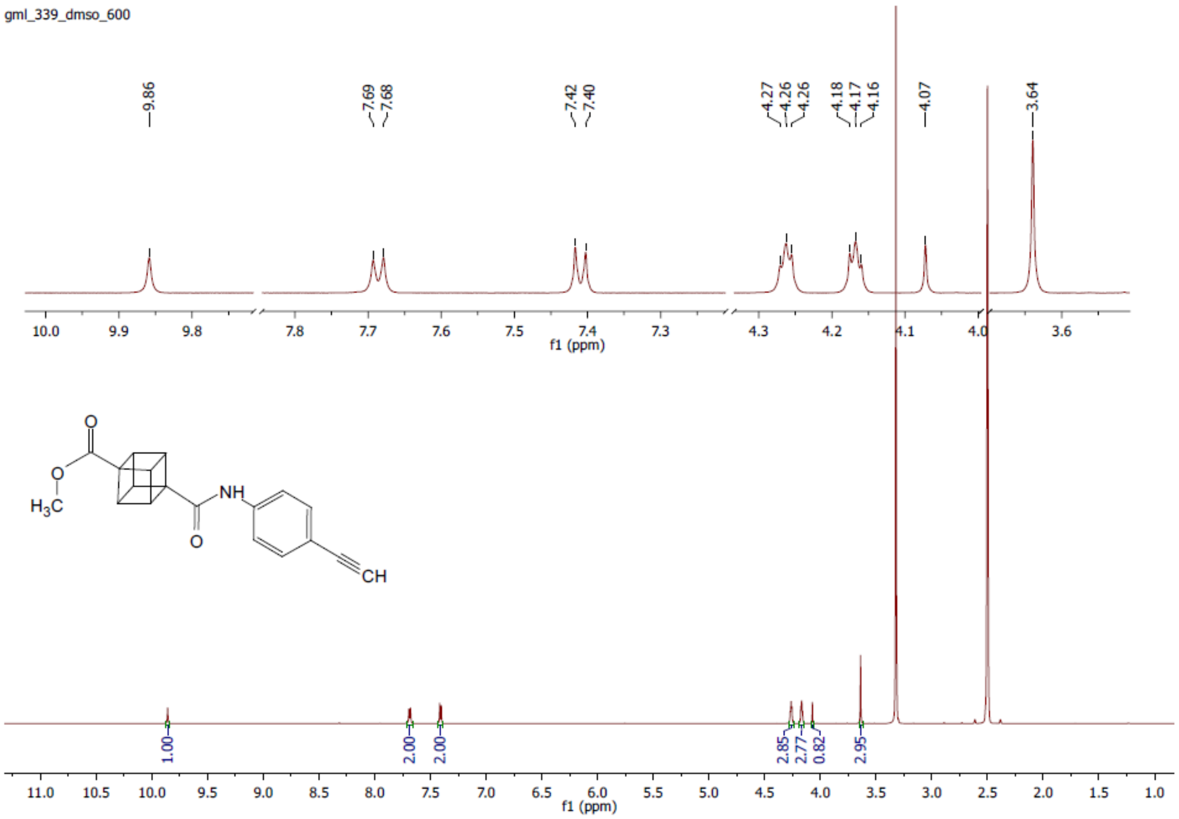

Fig. S37 ^{1}H NMR spectrum of cubane **14** in DMSO-d_6.

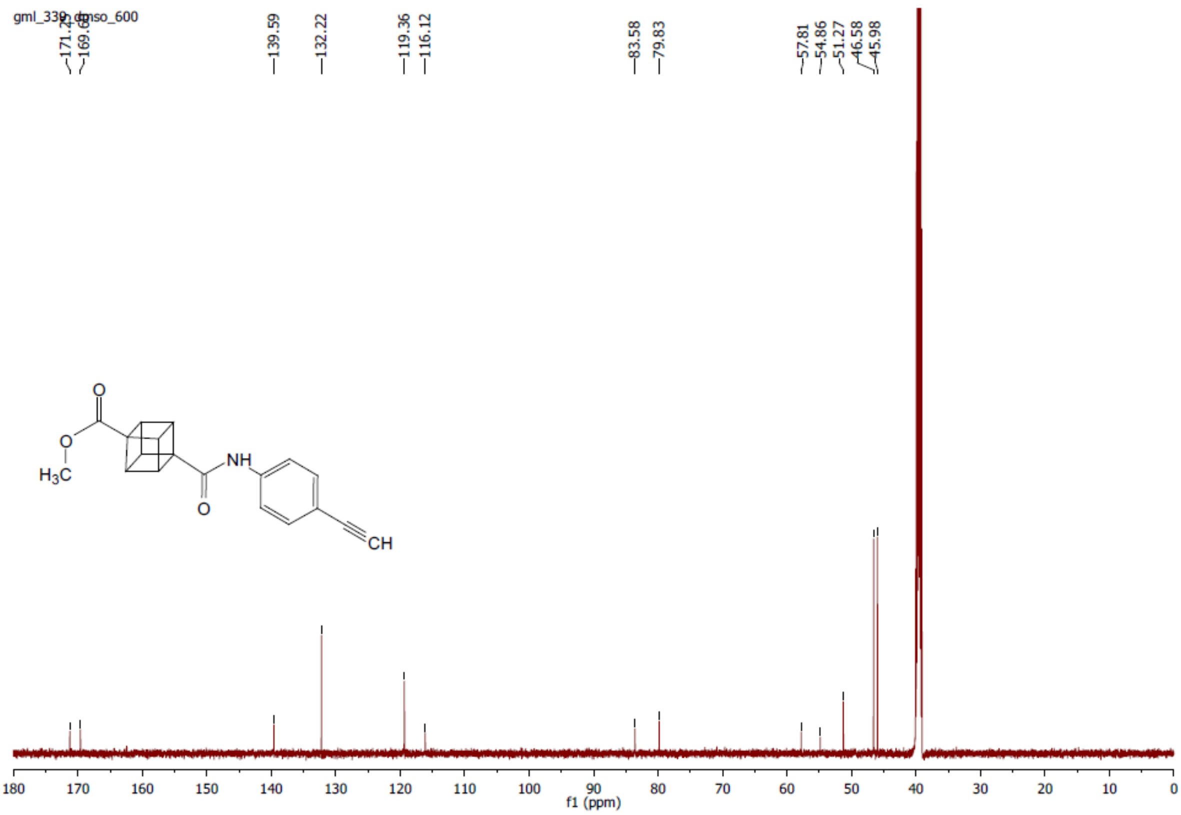

Fig. S38 ^{13}C NMR spectrum of cubane **14** in DMSO-d_6.

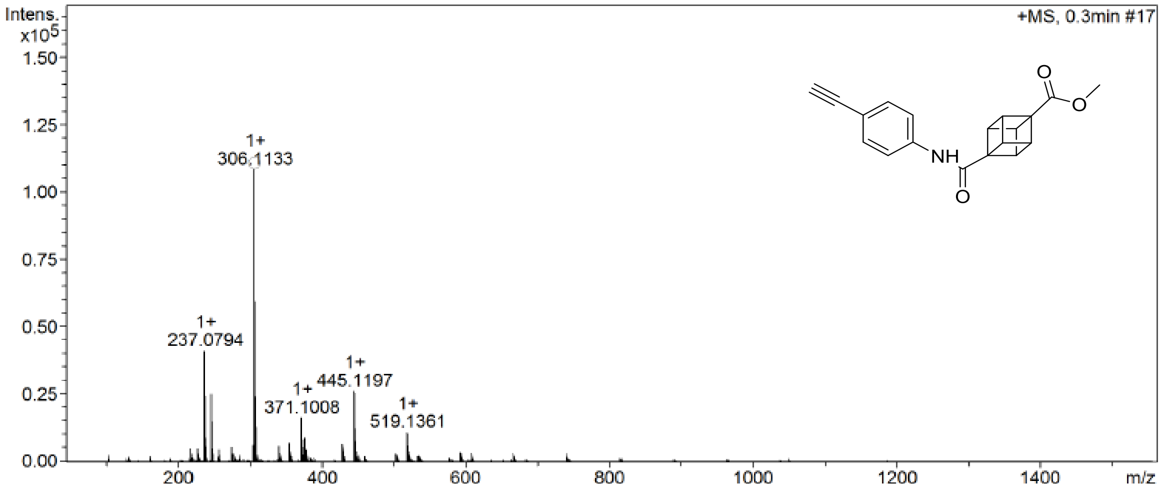

Meas. m/z	#	Ion Formula	m/z	err [mDa]	err [ppm]	rdb	N-Rule	e⁻ Conf	mSigma
306.113336	1	C19H16NO3	306.112470	-0.9	-2.8	12.5	ok	even	8.1

Fig. S39 HRMS (APCI) of cubane **14**.

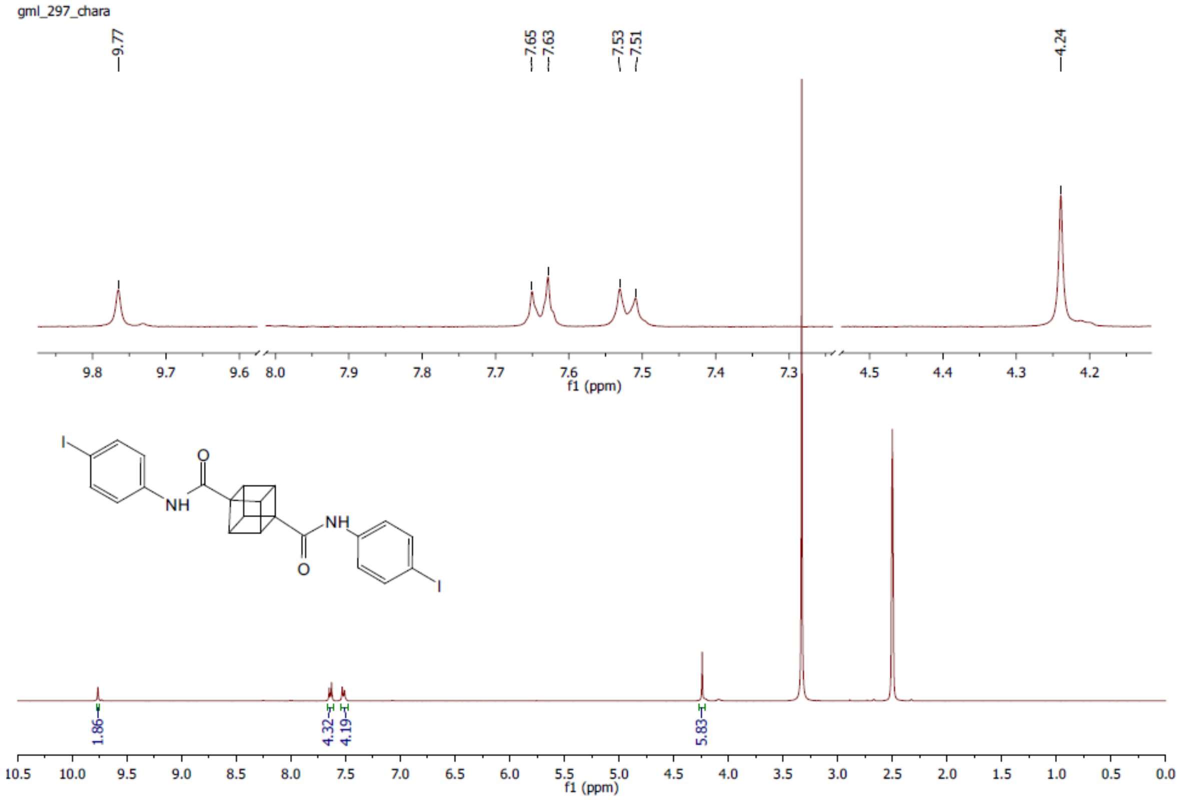

Fig. S40 ^{1}H NMR spectrum of cubane **15** in DMSO-d_6.

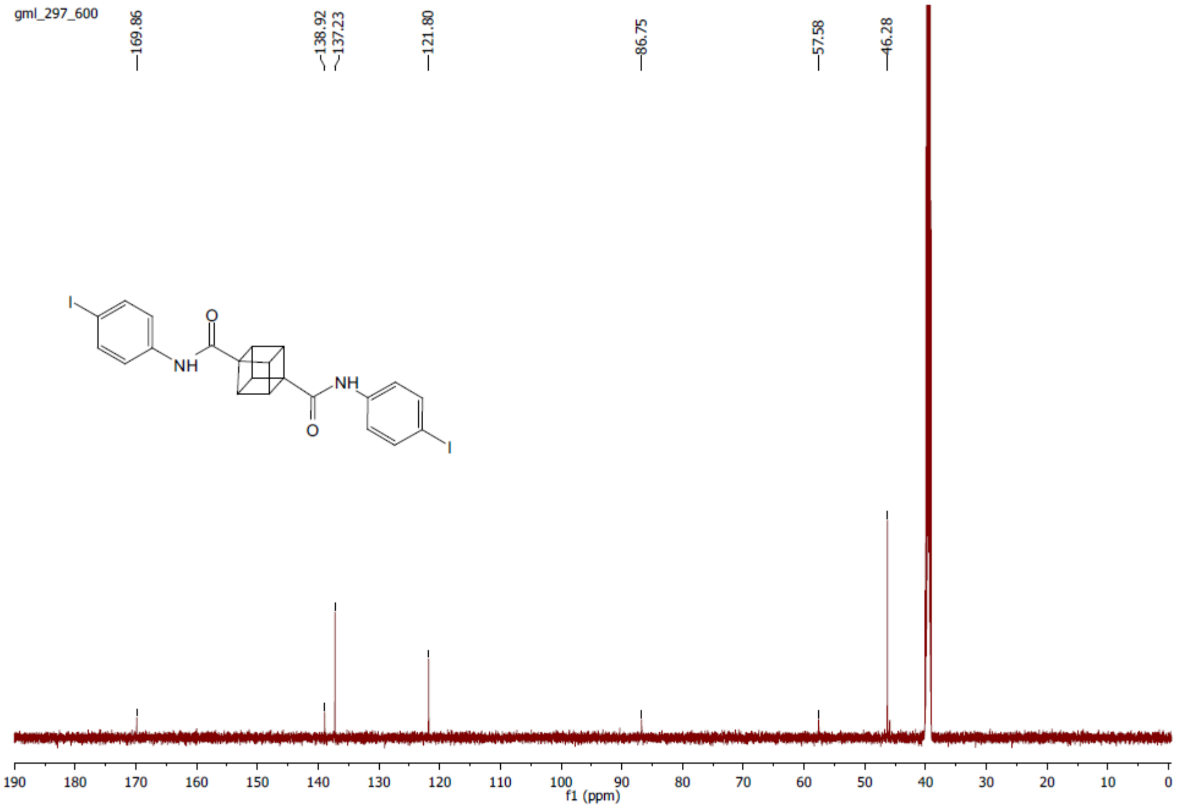

Fig. S41 ^{13}C NMR spectrum of cubane **15** in DMSO-d_6.

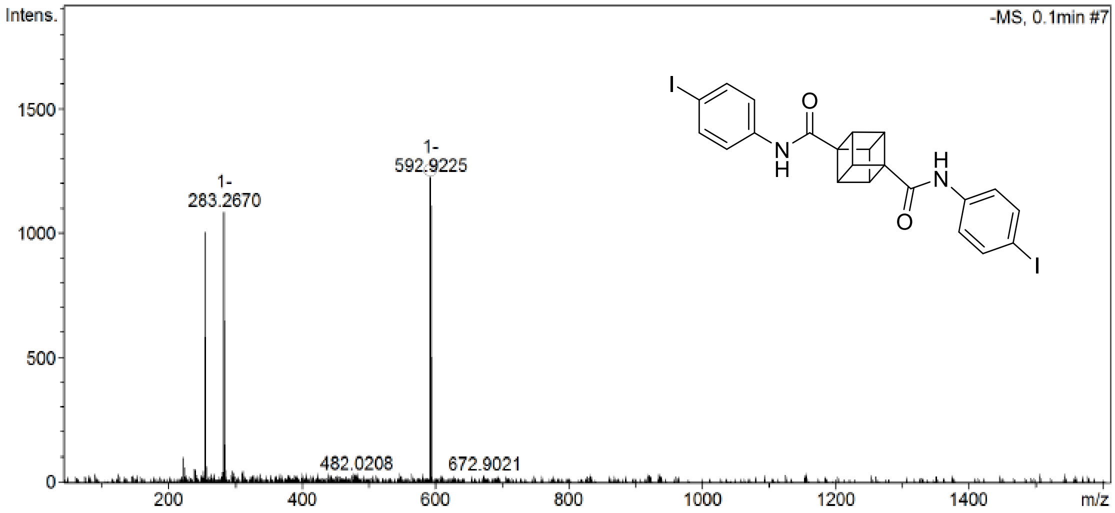

Meas. m/z	#	Ion Formula	m/z	err [mDa]	err [ppm]	rdb	N-Rule	e⁻ Conf	mSigma
592.922494	1	C22H15I2N2O2	592.922847	0.4	0.6	15.5	ok	even	26.1

Fig. S42 HRMS (ESI) of cubane **15**.

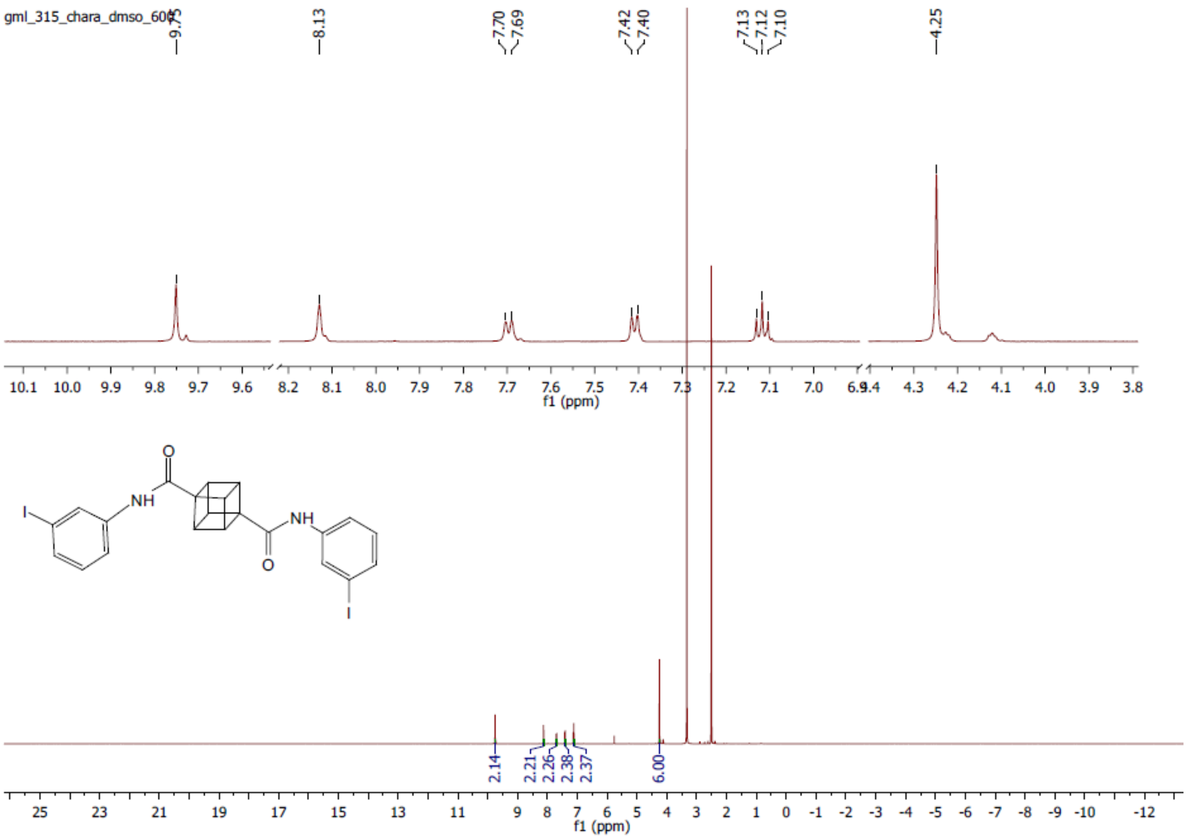

Fig. S43 ^{1}H NMR spectrum of cubane **16** in DMSO-d_6.

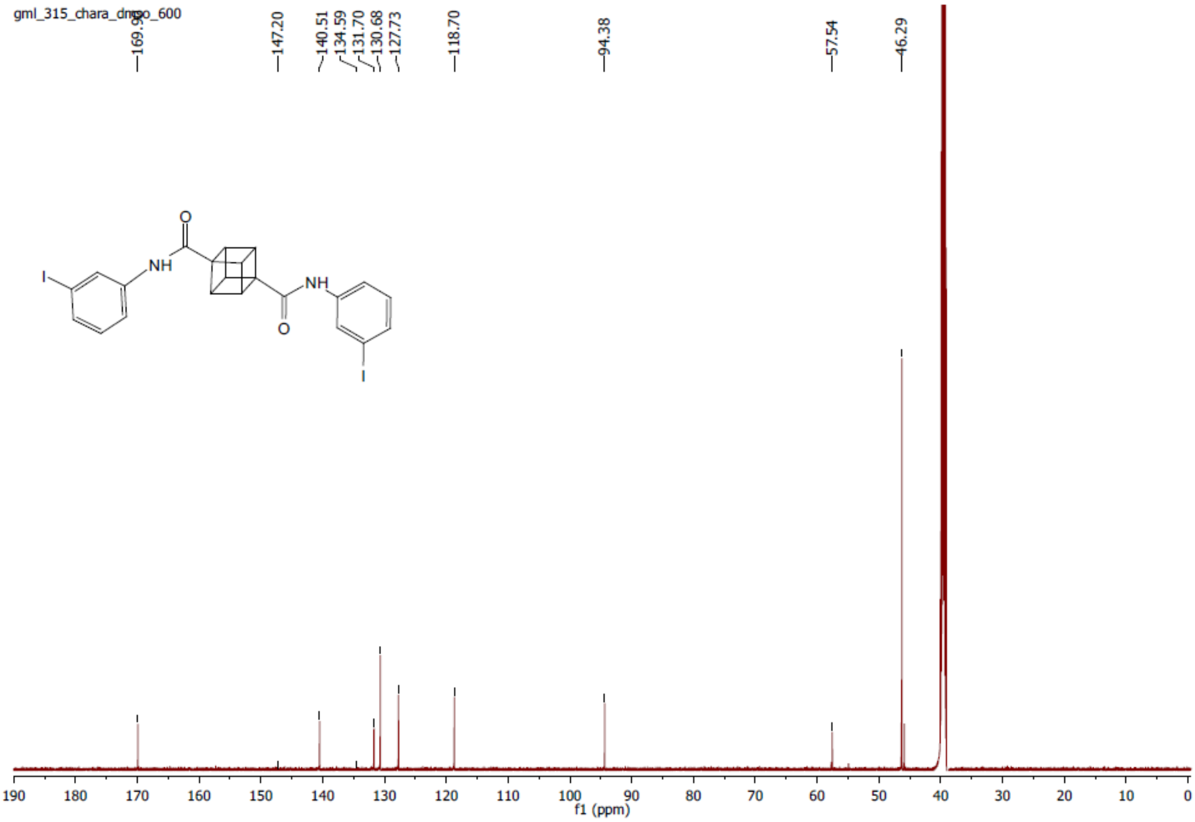

Fig. S44 ^{13}C NMR spectrum of cubane **16** in DMSO-d$_6$.

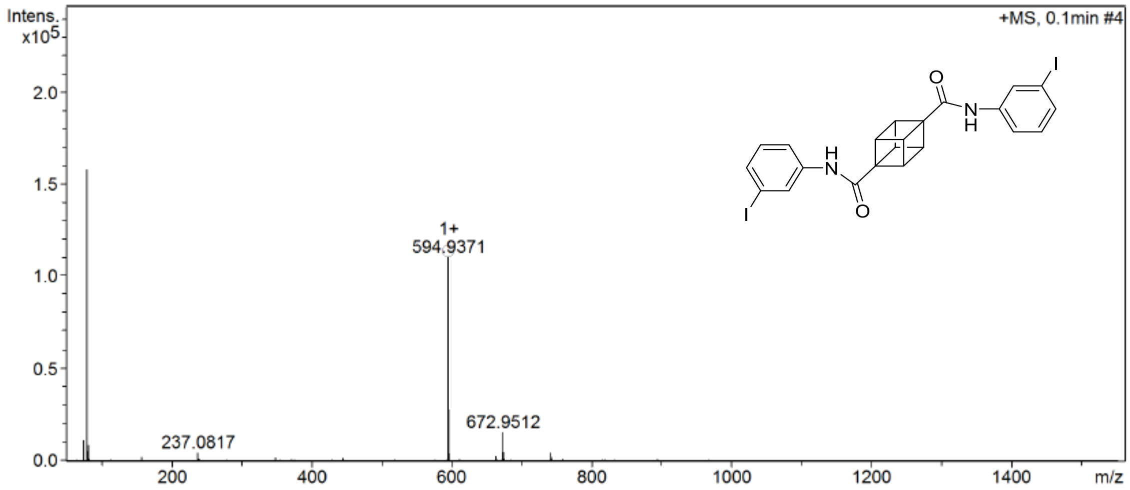

Meas. m/z	#	Ion Formula	m/z	err [mDa]	err [ppm]	rdb	N-Rule	e⁻ Conf	mSigma
594.937107	1	C22H17I2N2O2	594.937400	-0.3	-0.5	14.5	ok	even	2.7

Fig. S45 HRMS (ESI) of cubane **16**.

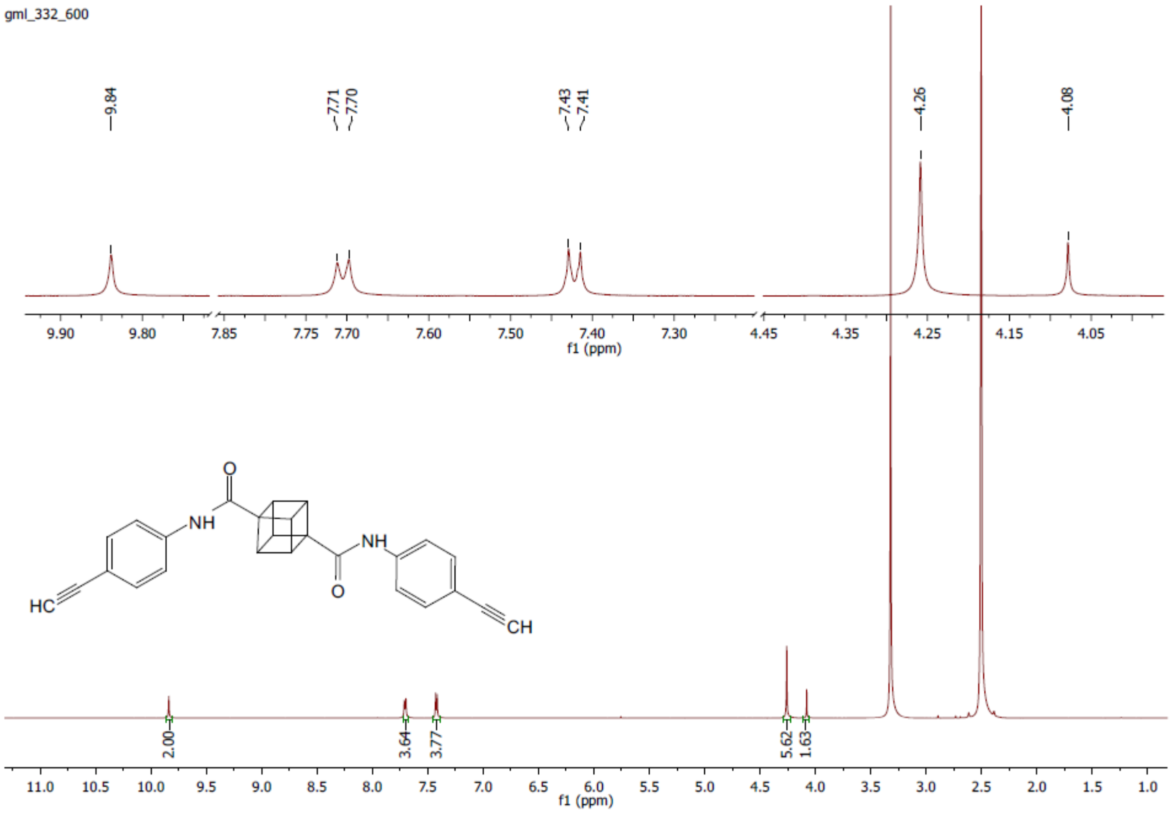

Fig. S46 ^{1}H NMR spectrum of cubane **17** in DMSO-d_6.

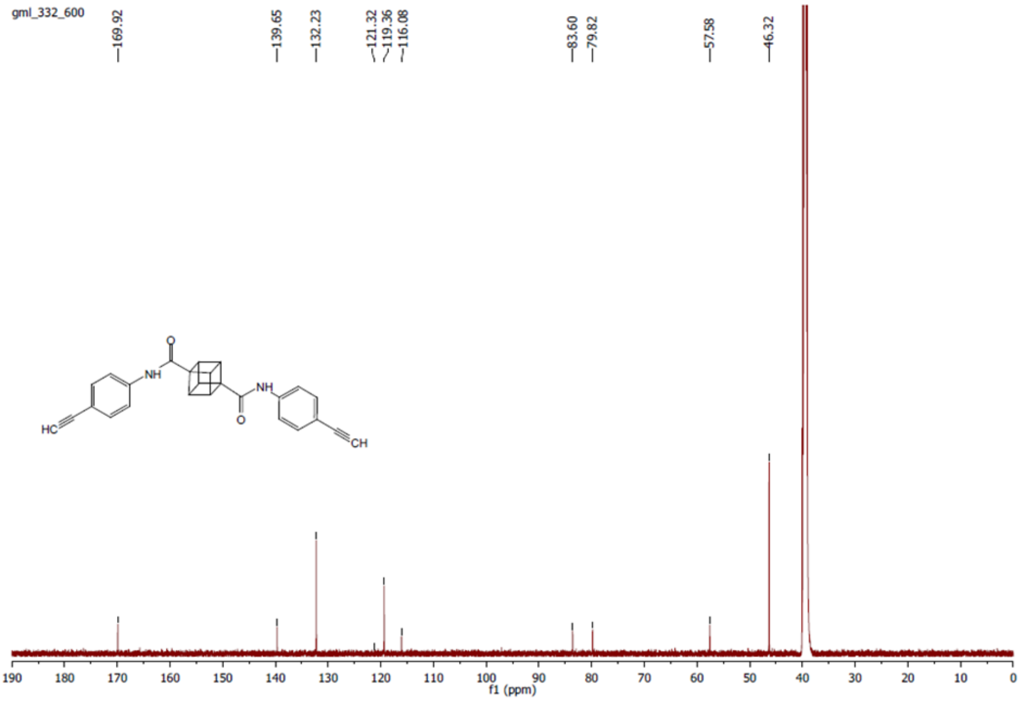

Fig. S47 ^{13}C NMR spectrum of cubane **17** in DMSO-d$_6$.

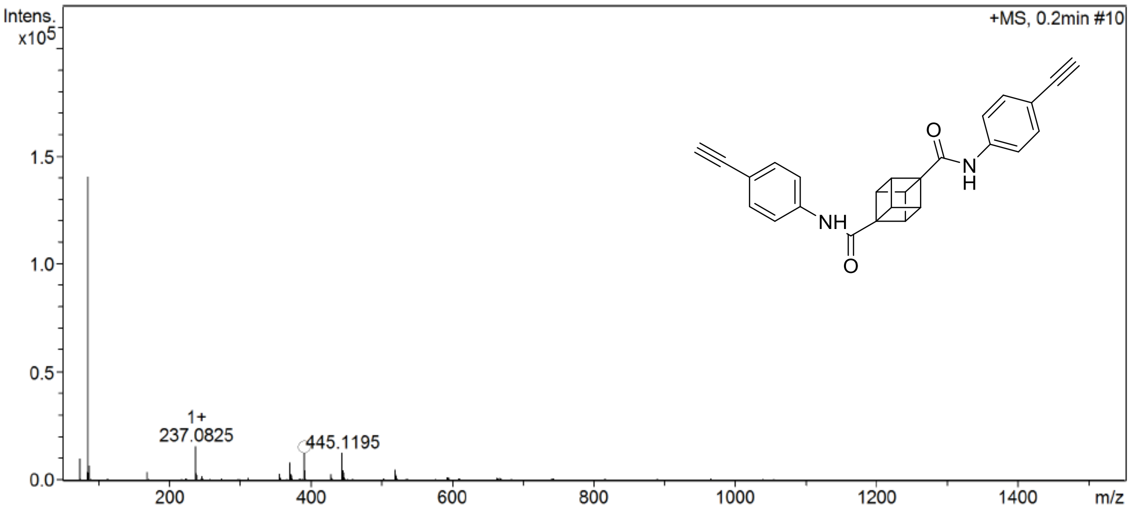

Meas. m/z	#	Ion Formula	m/z	err [mDa]	err [ppm]	rdb	N-Rule	e$^-$ Conf	mSigma
391.143171	1	C26H19N2O2	391.144104	-0.9	-2.4	18.5	ok	even	40.9

Fig. S48 HRMS (ESI) of cubane **17**.

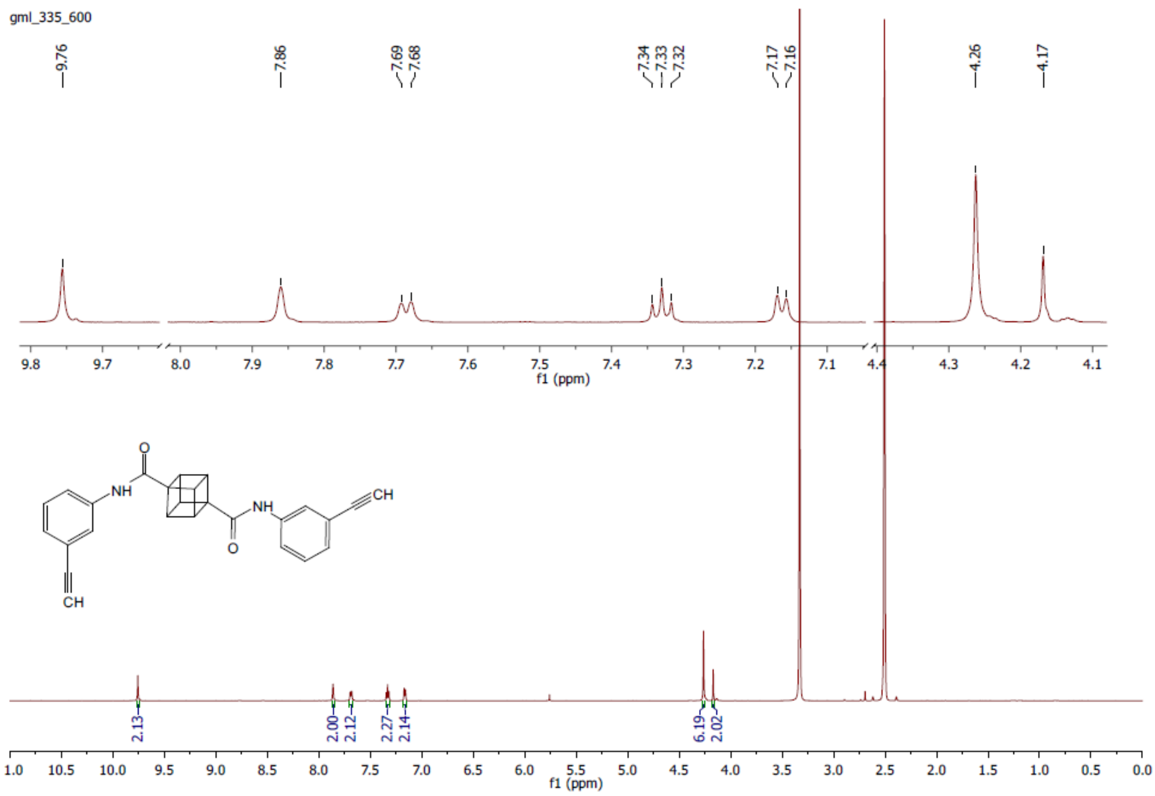

Fig. S49 ^{1}H NMR spectrum of cubane **18** in DMSO-d_6.

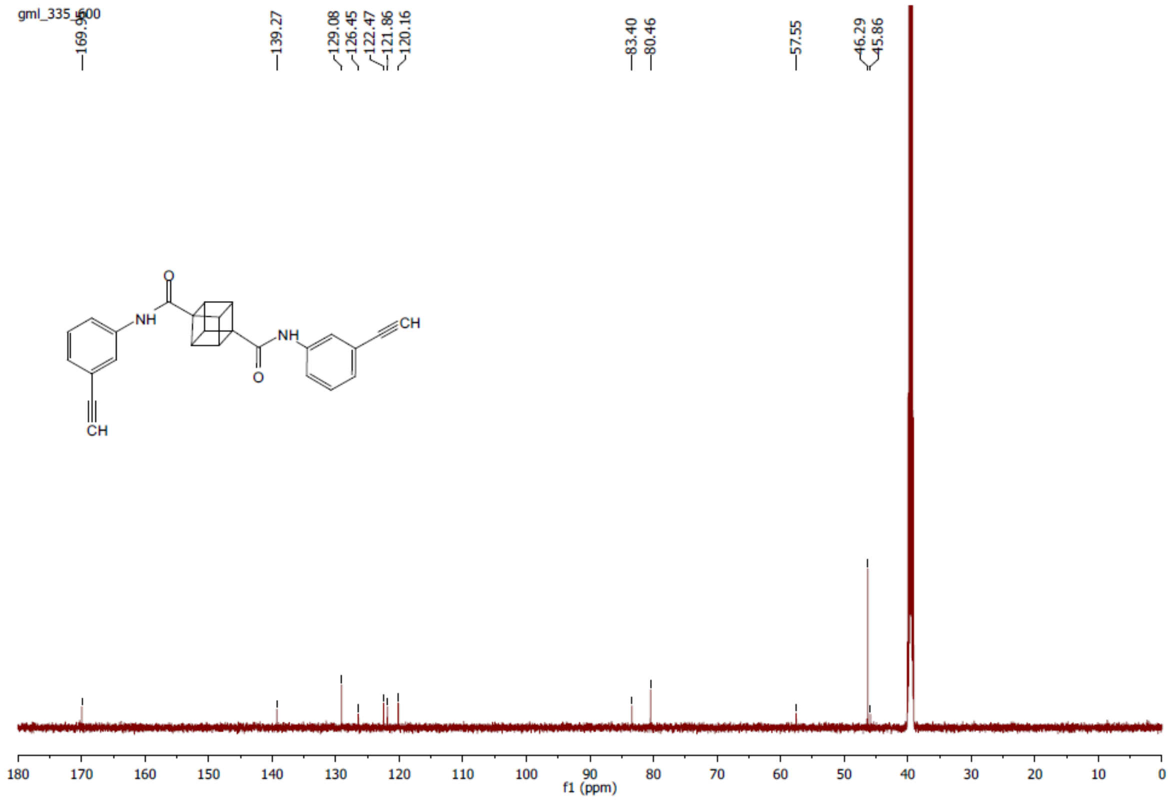

Fig. S50 ^{13}C NMR spectrum of cubane **18** in DMSO-d_6.

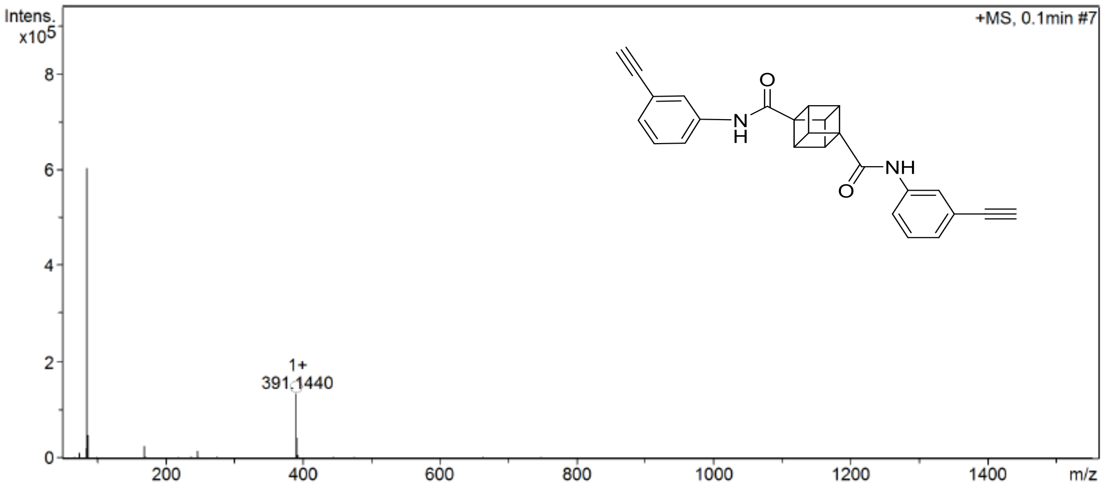

Meas. m/z	#	Ion Formula	m/z	err [mDa]	err [ppm]	rdb	N-Rule	e⁻ Conf	mSigma
391.143981	1	C26H19N2O2	391.144104	-0.1	-0.3	18.5	ok	even	11.1

Fig. S51 HRMS (ESI) of cubane **18**.

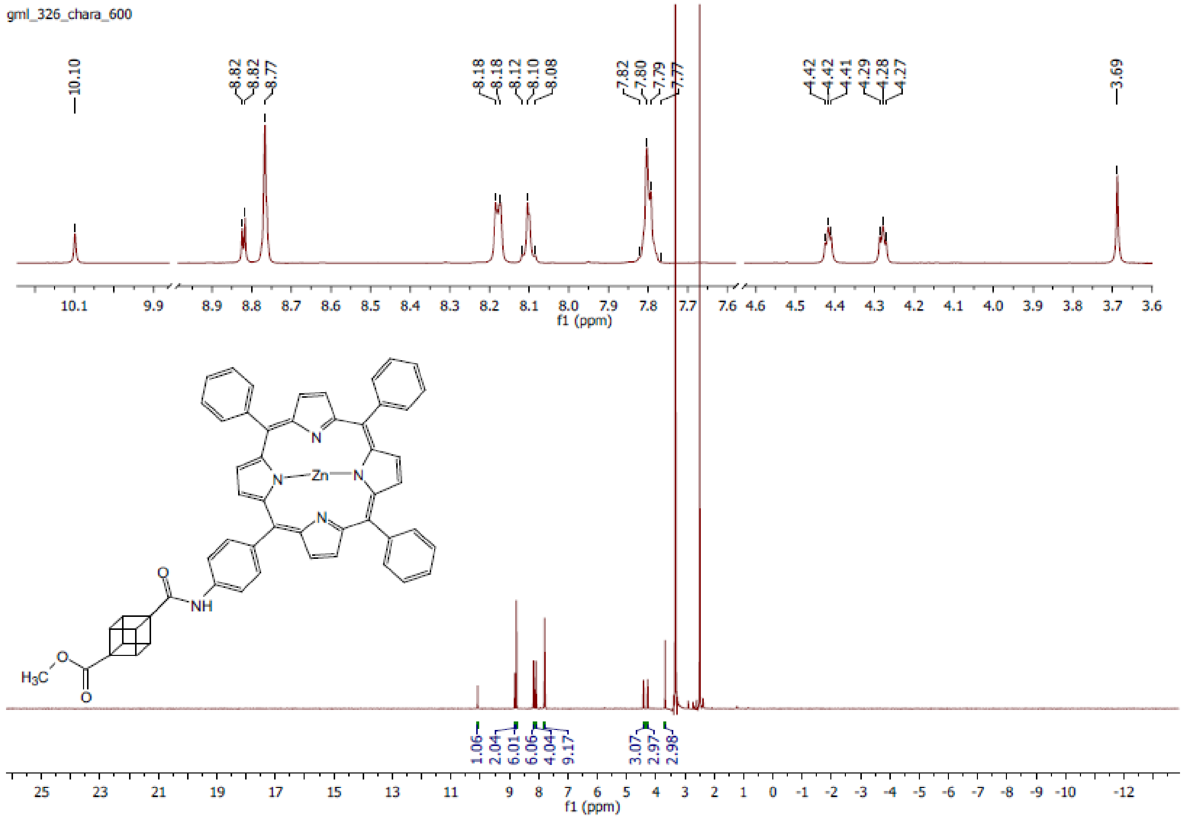

Fig. S52 ^{1}H NMR spectrum of cubane porphyrin monomer **19** in DMSO-d_6.

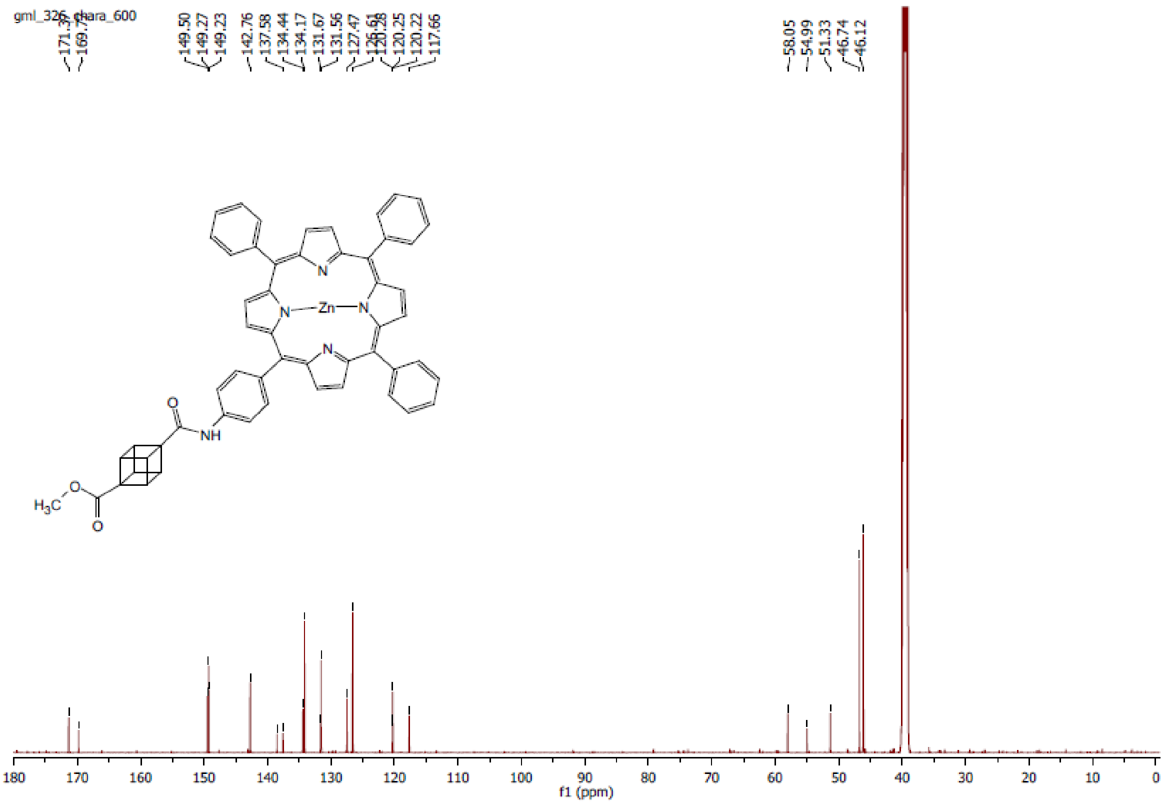

Fig. S53 ^{13}C NMR spectrum of cubane porphyrin monomer **19** in DMSO-d$_6$.

Elemental Composition Report **Page 1**

Single Mass Analysis
Tolerance = 10.0 PPM / DBE: min = -1.5, max = 400.0
Element prediction: Off
Number of isotope peaks used for i-FIT = 5

Monoisotopic Mass, Odd and Even Electron Ions
61 formula(e) evaluated with 1 results within limits (up to 10 closest results for each mass)
Elements Used:
C: 0-55 H: 0-37 N: 0-5 O: 0-3 Zn: 0-2
Gemma Locke (MSe), GML-326
Q-TOF20190123MF007 84 (4.218) AM (Cen,8, 80.00, Ht,10000.0,1570.68,0.70); Sm (SG, 2x3.00); Sb (15,10.00); Cm (45:87) TOF MS LD+ 1.98e+003

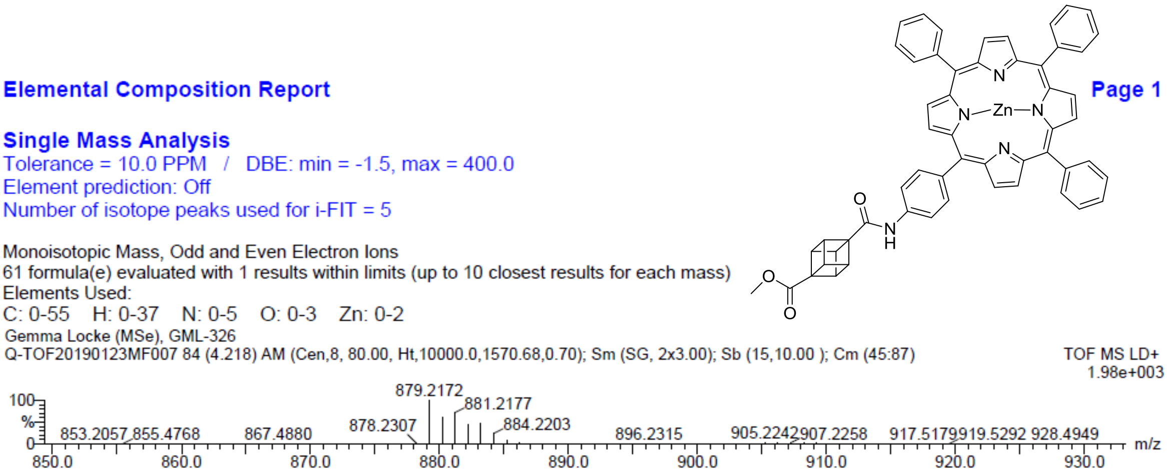

Minimum:				-1.5			
Maximum:		5.0	10.0	400.0			
Mass	Calc. Mass	mDa	PPM	DBE	i-FIT	i-FIT (Norm)	Formula
879.2172	879.2188	-1.6	-1.8	40.0	65.5	0.0	C55 H37 N5 O3 Zn

Fig. S54 MALDI-TOF-MS of cubane **19**.

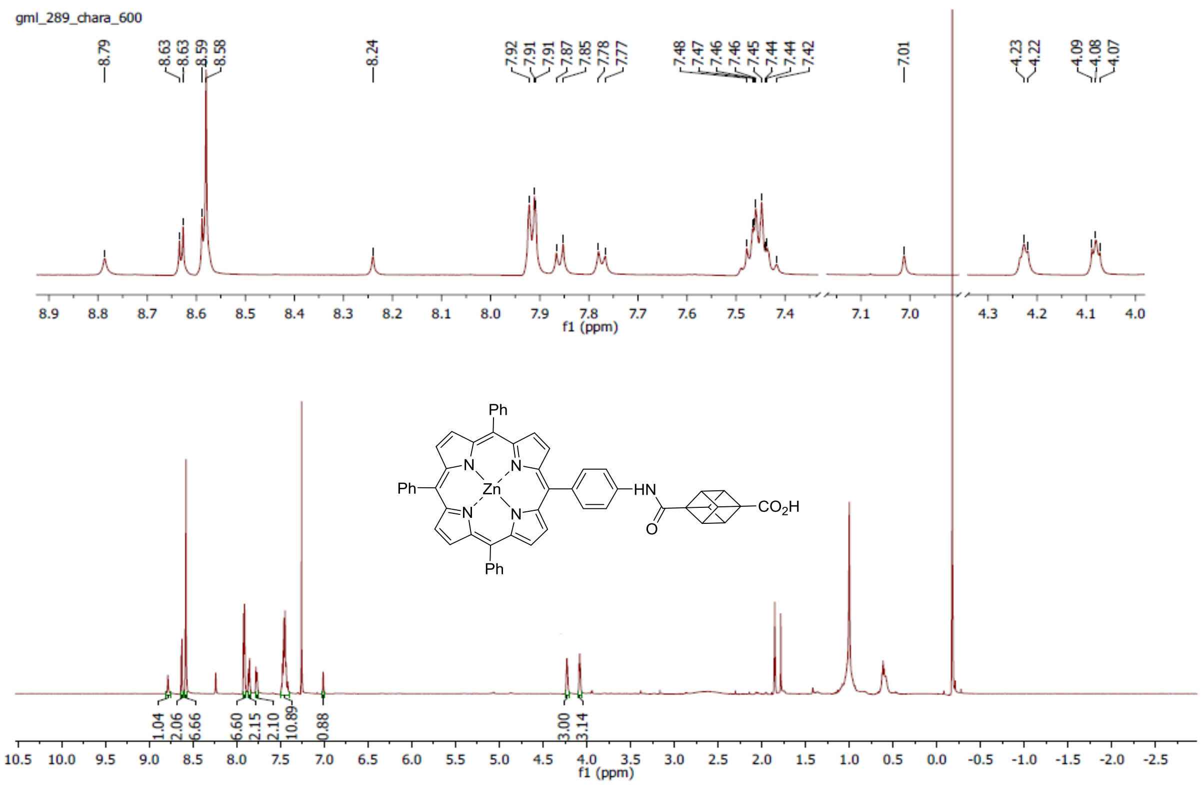

Fig. S55 ^{1}H NMR spectrum of cubane porphyrin monomer **20** in $CDCl_3$:$(CD_3)_2CO$: Pyridine-d_5 (5:2:0.01).

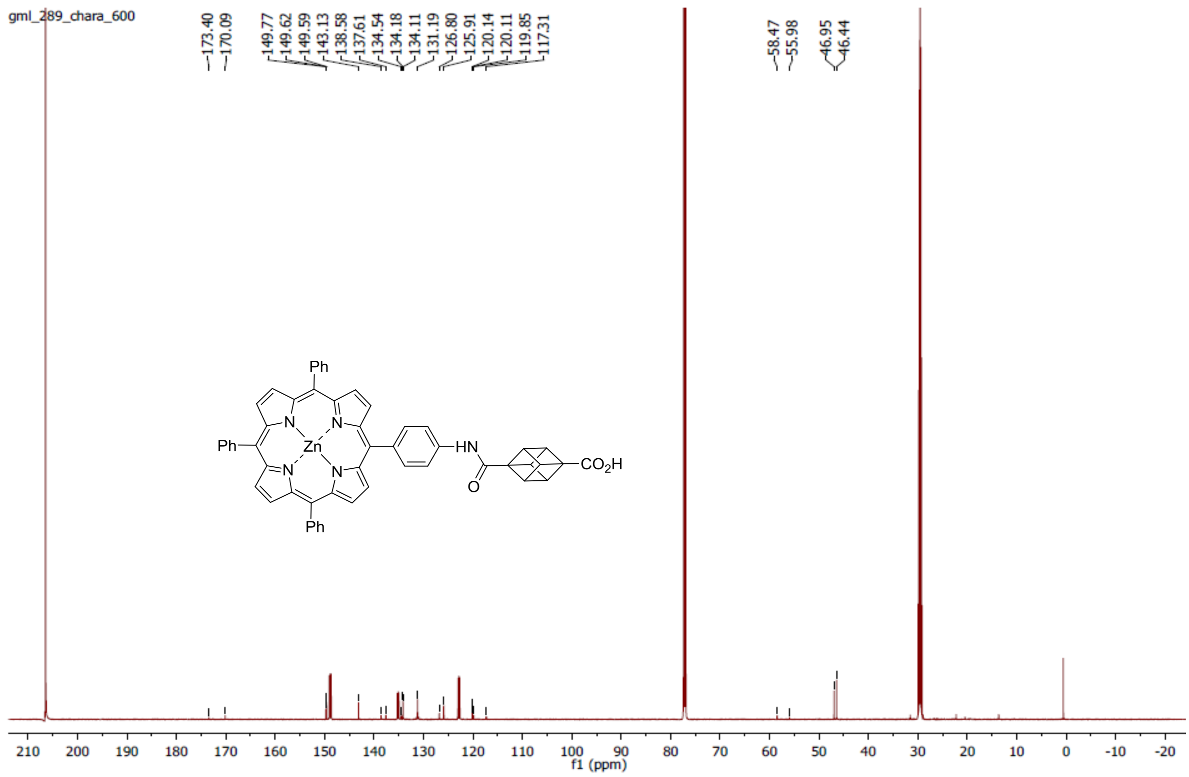

Fig. S56 ^{13}C NMR spectrum of cubane porphyrin monomer **20** in $CDCl_3$:$(CD_3)_2CO$: Pyridine-d_5 (5:2:0.01).

Elemental Composition Report

Page 1

Single Mass Analysis

Tolerance = 500.0 PPM / DBE: min = -1.5, max = 400.0
Element prediction: Off
Number of isotope peaks used for i-FIT = 5

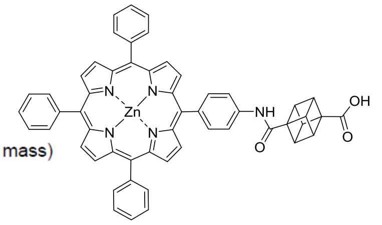

Monoisotopic Mass, Odd and Even Electron Ions
28 formula(e) evaluated with 1 results within limits (up to 10 best isotopic matches for each mass)
Elements Used:
C: 0-54 H: 0-35 N: 0-5 O: 0-3 Zn: 0-1

Gemma Locke (MSE), GML-289
Q-TOF20180906MF003 42 (0.778) AM (Cen,6, 80.00, Ht,10000.0,1570.68,0.70); Sm (SG, 2x3.00); Sb (15,10.00); Cm (7:90-32:56)

TOF MS LD+
5.16e+002

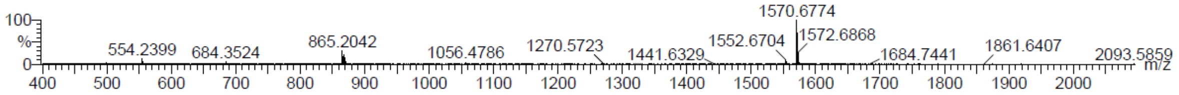

Minimum:				-1.5				
Maximum:		5.0	500.0	400.0				
Mass	Calc. Mass	mDa	PPM	DBE	i-FIT	i-FIT (Norm)	Formula	
865.2042	865.2031	1.1	1.3	40.0	16.6	0.0	C54 H35 N5 O3 Zn	

Fig. S57 MALDI-TOF-MS of cubane porphyrin monomer **20**.

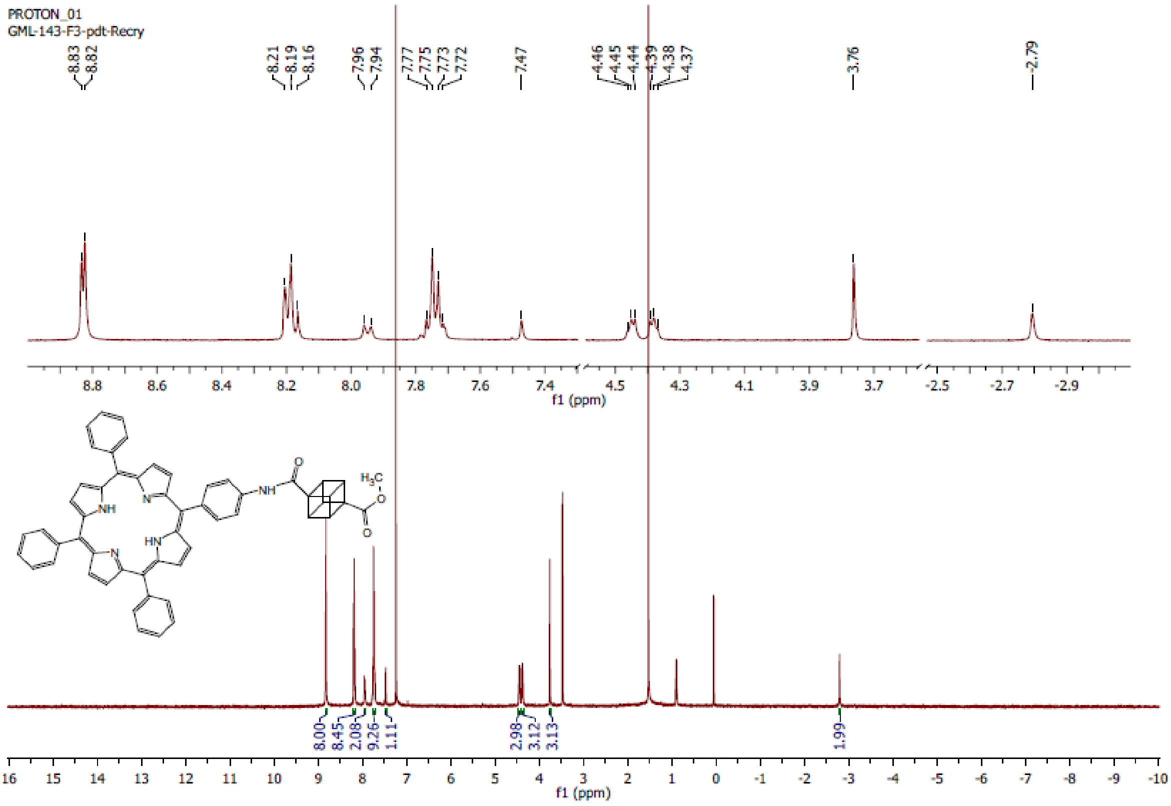

Fig. S58 ^{1}H NMR spectrum of cubane porphyrin monomer **21** in $CDCl_3$.

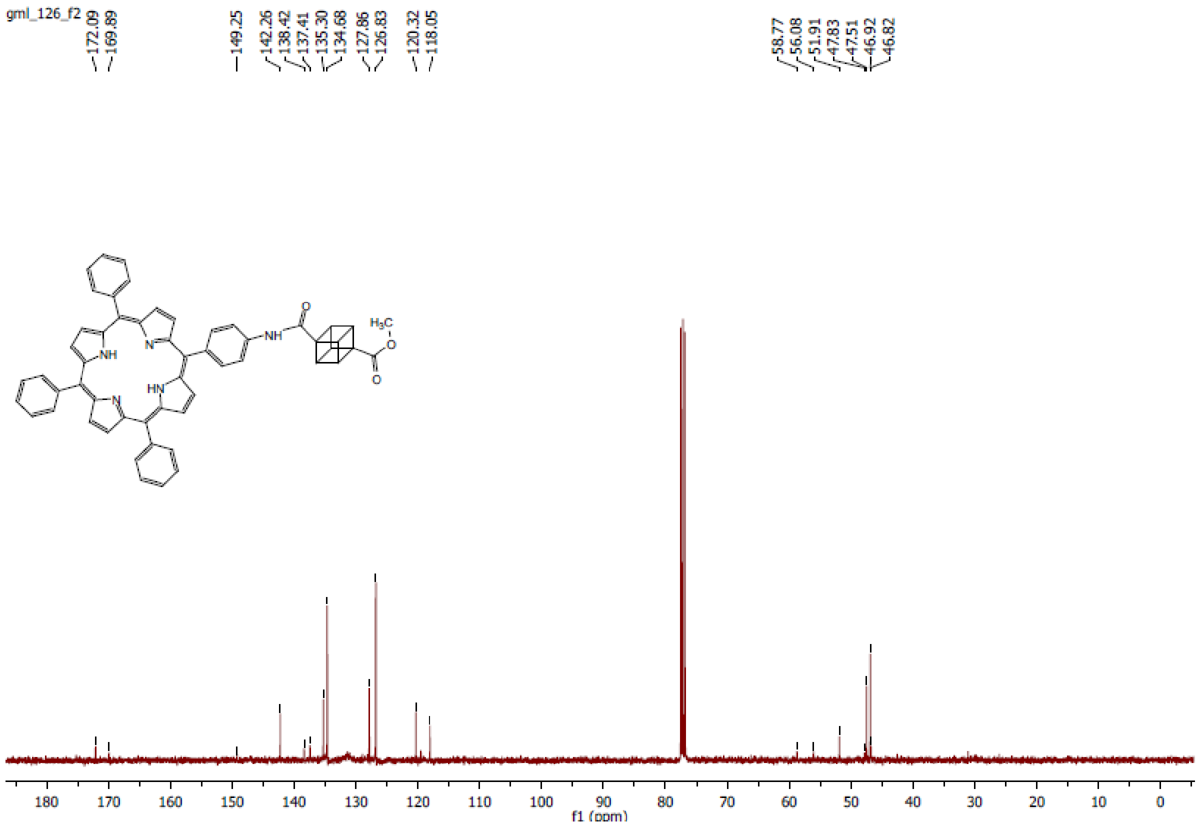

Fig. S59 ^{13}C NMR spectrum of cubane porphyrin monomer **21** in $CDCl_3$.

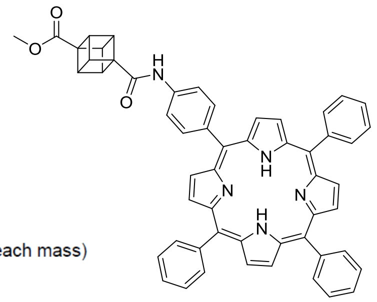

Elemental Composition Report

Page 1

Single Mass Analysis

Tolerance = 10.0 PPM / DBE: min = -1.5, max = 500.0
Element prediction: Off
Number of isotope peaks used for i-FIT = 3

Monoisotopic Mass, Odd and Even Electron Ions
9 formula(e) evaluated with 1 results within limits (up to 10 closest results for each mass)
Elements Used:
C: 0-55 H: 0-40 N: 0-6 O: 0-3

Gemma Locke (MSe), GML-135-F3
Q-TOF20170127MF004 71 (1.515) AM (Cen,4, 80.00, Ht,10000.0,1570.68,0.70); Sm (SG, 1x3.00); Sb (15,10.00); Cm (16:103-(66:78+95:100)) TOF MS LD+
5.65e+002

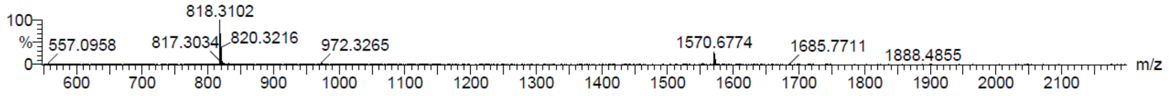

Minimum:				-1.5			
Maximum:		5.0	10.0	500.0			
Mass	Calc. Mass	mDa	PPM	DBE	i-FIT	i-FIT (Norm)	Formula
818.3102	818.3131	-2.9	-3.5	38.5	47.6	0.0	C55 H40 N5 O3

Fig. S60 MALDI-TOF-MS of cubane porphyrin monomer **21**.

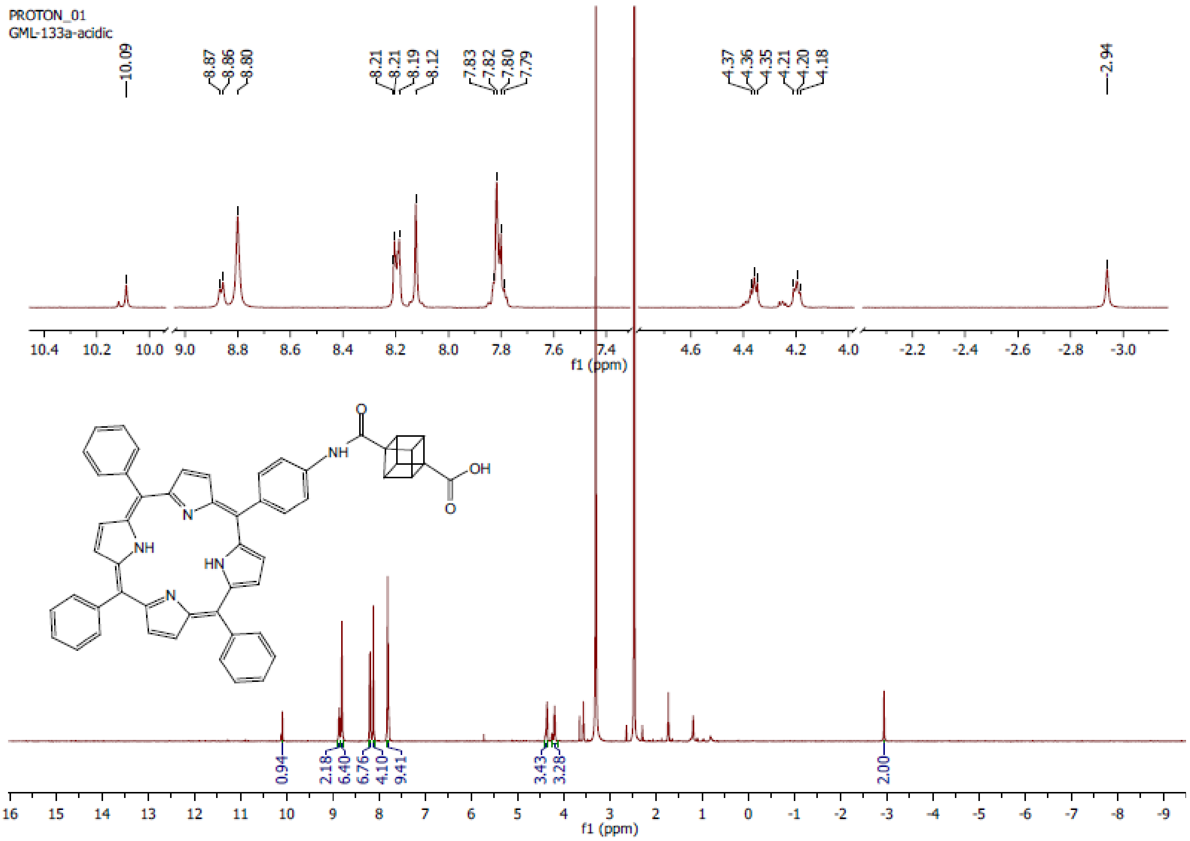

Fig. S61 ^{1}H NMR spectrum of cubane porphyrin monomer **22** in DMSO-d_6.

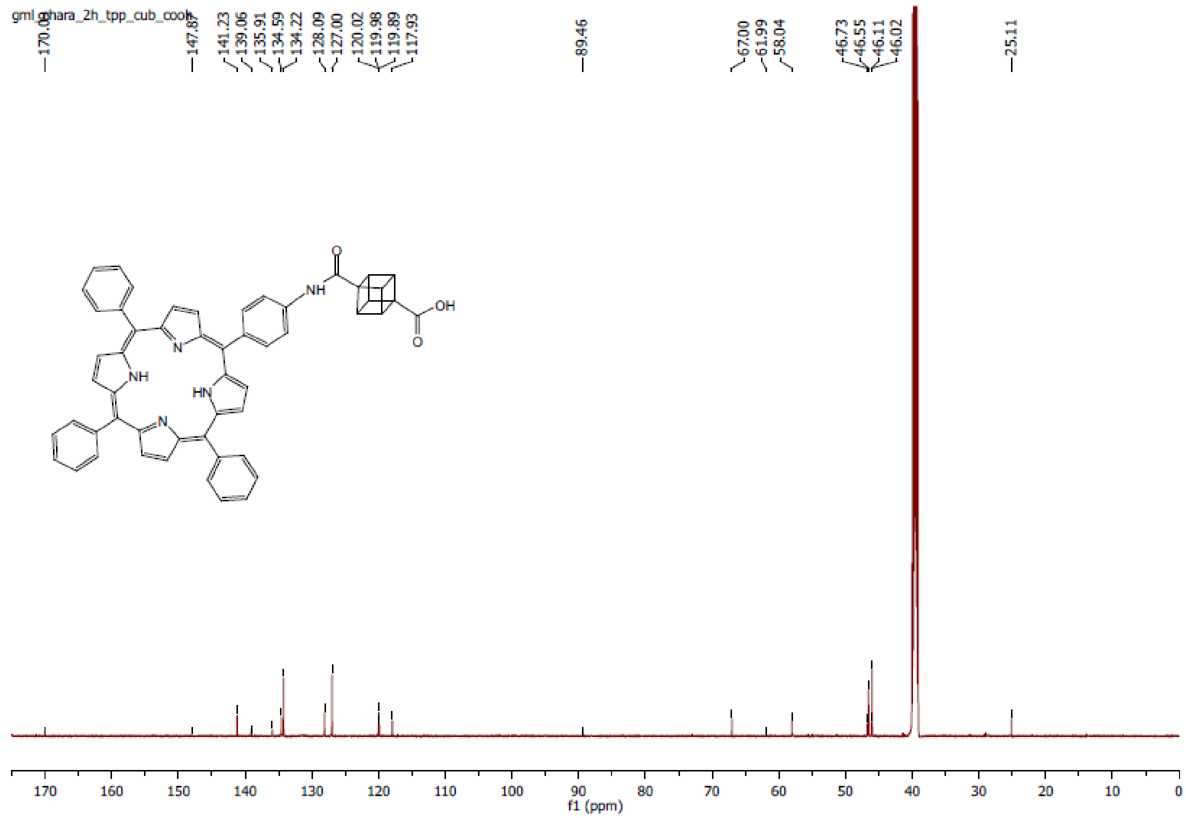

Fig. S62 ^{13}C NMR spectrum of cubane porphyrin monomer **22** in DMSO-d_6.

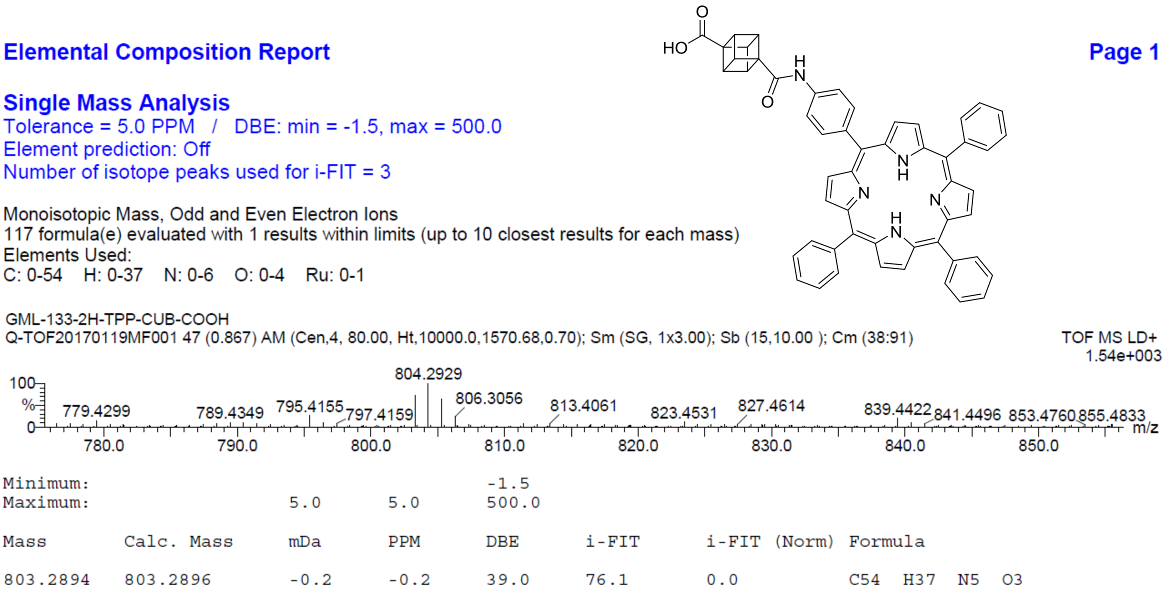

Fig. S63 MALDI-TOF-MS of cubane porphyrin monomer **22**.

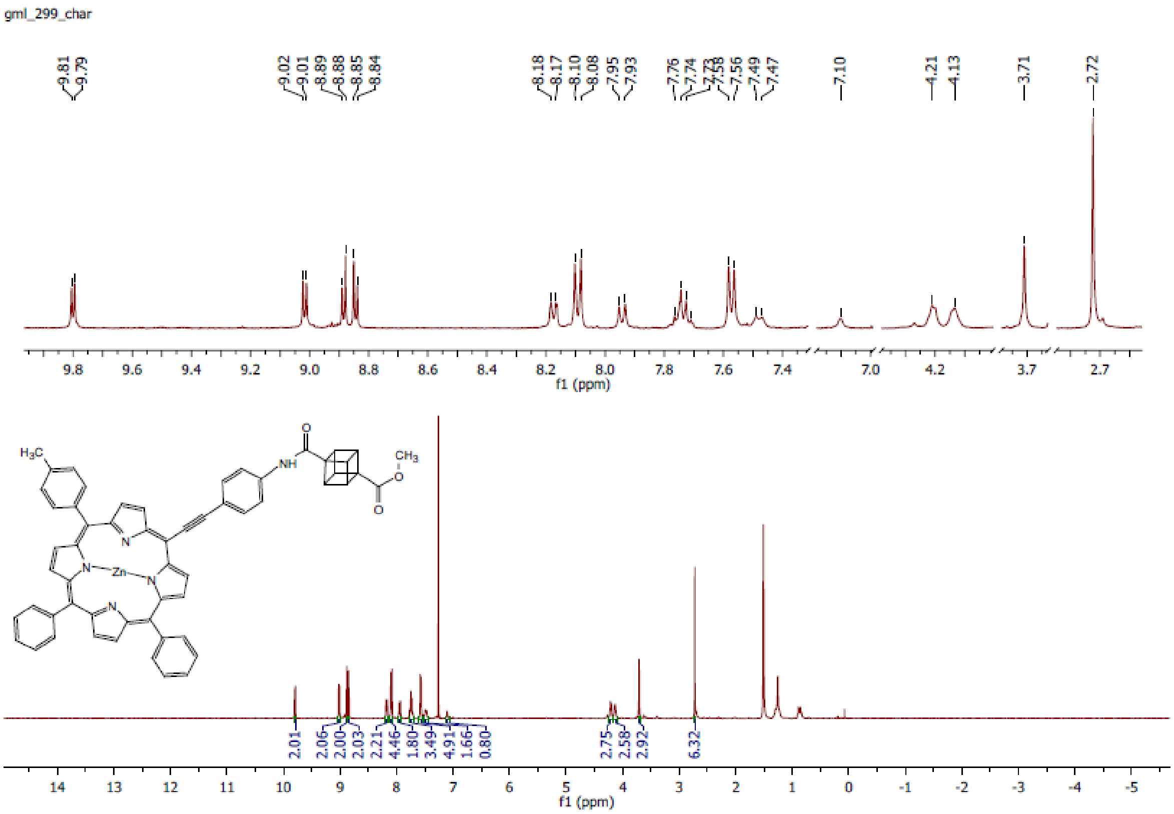

Fig. S64 ^{1}H NMR spectrum of cubane porphyrin monomer **23** in $CDCl_3$.

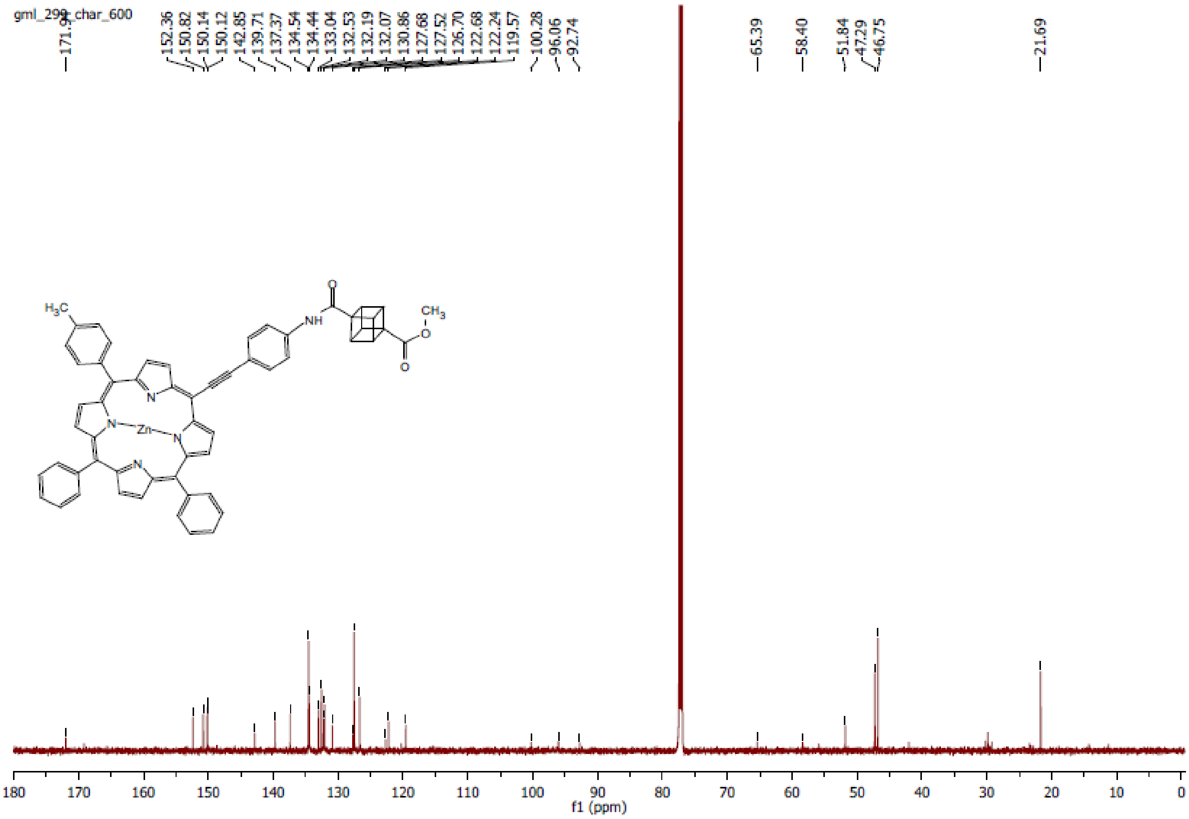

Fig. S65 ^{13}C NMR of cubane porphyrin monomer **23** in $CDCl_3$.

Elemental Composition Report

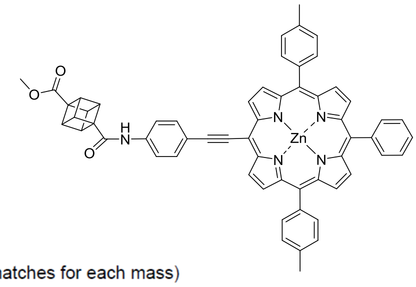

Page 1

Single Mass Analysis

Tolerance = 20.0 PPM / DBE: min = -1.5, max = 400.0
Element prediction: Off
Number of isotope peaks used for i-FIT = 5

Monoisotopic Mass, Odd and Even Electron Ions
58 formula(e) evaluated with 1 results within limits (up to 10 best isotopic matches for each mass)
Elements Used:
C: 0-59 H: 0-41 N: 0-5 O: 0-3 Zn: 0-2
Gemma Locke (MSe), GML-299
Q-TOF20180920MF029 53 (1.165) AM (Cen,8, 80.00, Ht,10000.0,1570.68,0.70); Sm (SG, 2x3.00); Sb (15,10.00); Cm (21:78-53) TOF MS LD+ 7.97e+002

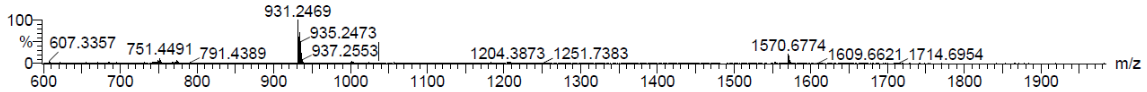

Minimum:				-1.5			
Maximum:		5.0	20.0	400.0			
Mass	Calc. Mass	mDa	PPM	DBE	i-FIT	i-FIT (Norm)	Formula
931.2469	931.2501	-3.2	-3.4	42.0	58.9	0.0	C59 H41 N5 O3 Zn

Fig. S66 MALDI-TOF-MS of cubane porphyrin monomer **23**.

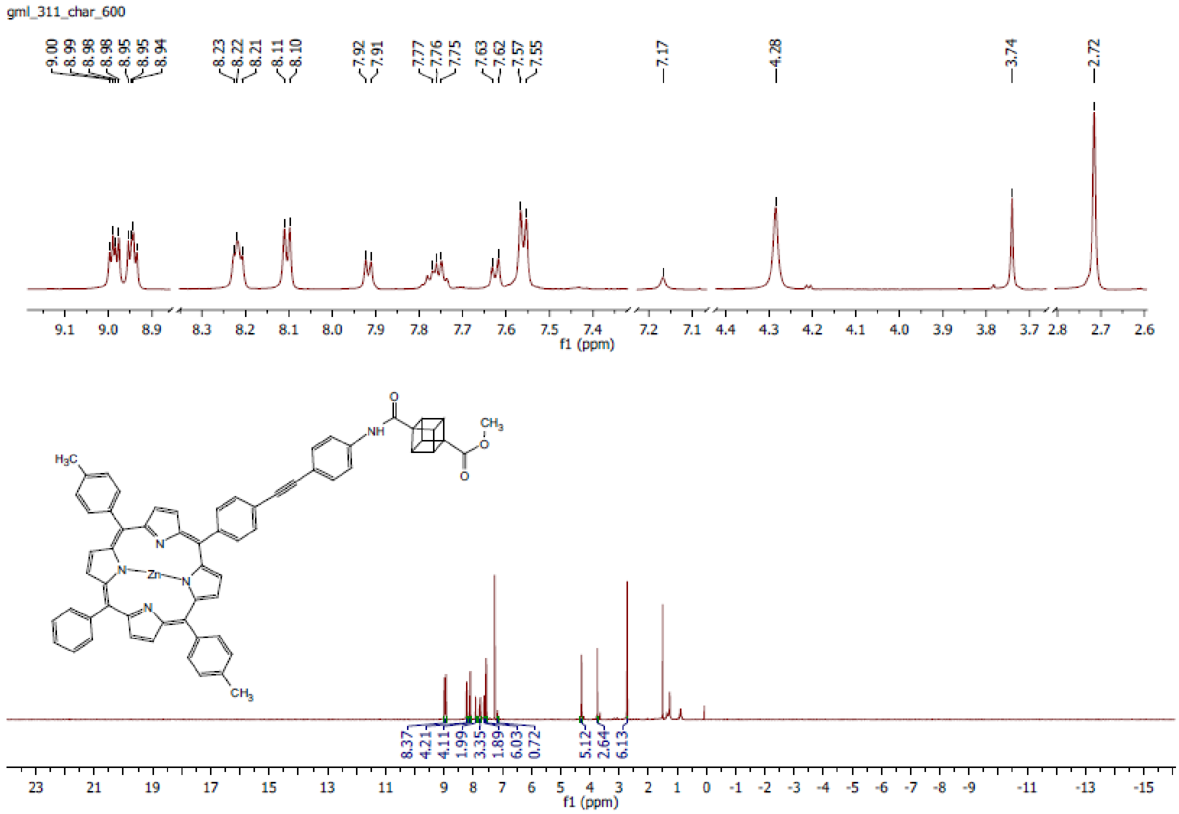

Fig. S67 ^{1}H NMR spectrum of cubane porphyrin monomer **24** in $CDCl_3$.

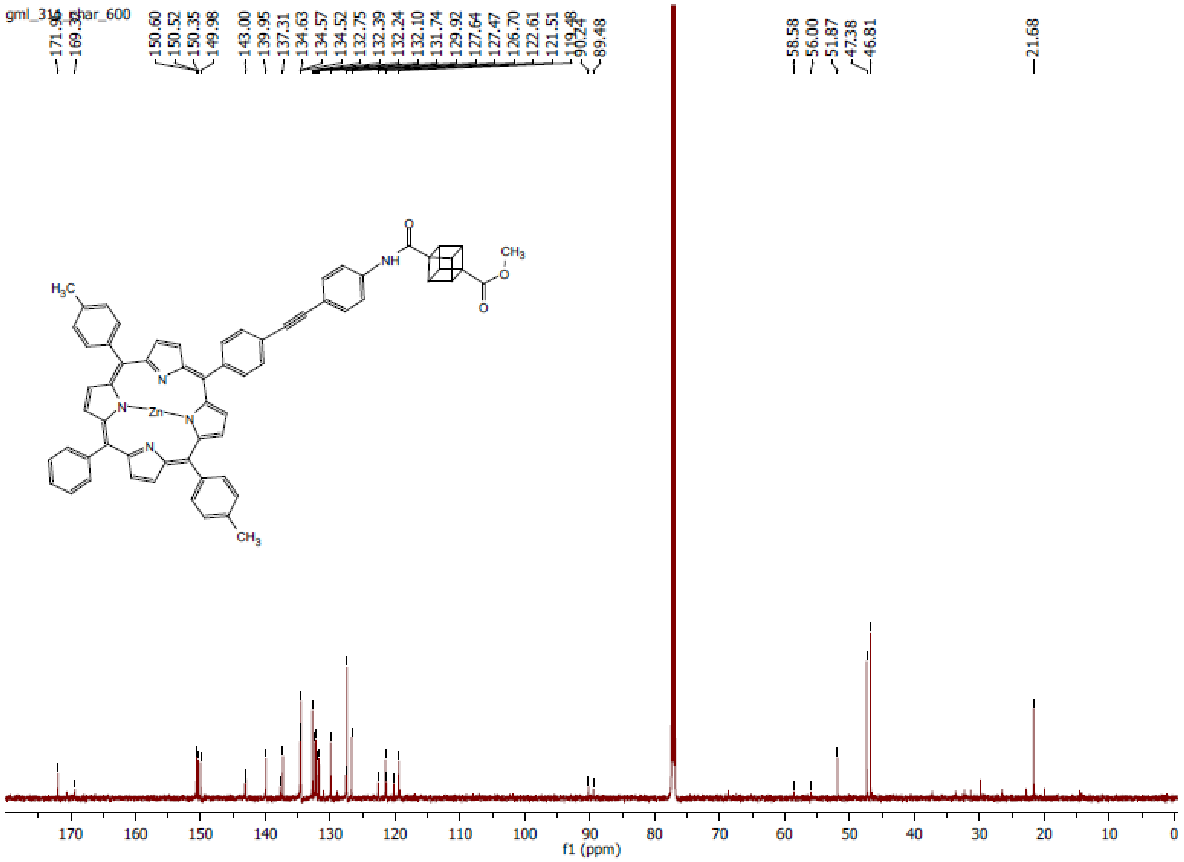

Fig. S68 ^{13}C NMR spectrum of cubane porphyrin monomer **24** in $CDCl_3$.

Elemental Composition Report

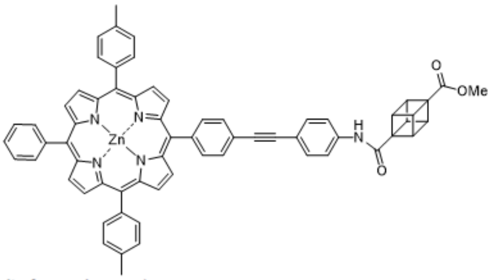

Page 1

Single Mass Analysis
Tolerance = 100.0 PPM / DBE: min = -1.5, max = 400.0
Element prediction: Off
Number of isotope peaks used for i-FIT = 5

Monoisotopic Mass, Odd and Even Electron Ions
22 formula(e) evaluated with 1 results within limits (up to 10 closest results for each mass)
Elements Used:
C: 0-65 H: 0-45 N: 0-5 O: 0-3 Zn: 0-1

Gemma Locke (MSe), GML-311-P2
Q-TOF20181119MF20 52 (1.220) AM (Cen,8, 80.00, Ht,10000.0,1570.68,0.70); Sm (SG, 2x3.00); Sb (15,10.00); Cm (9:66-52)
TOF MS LD+
1.70e+003

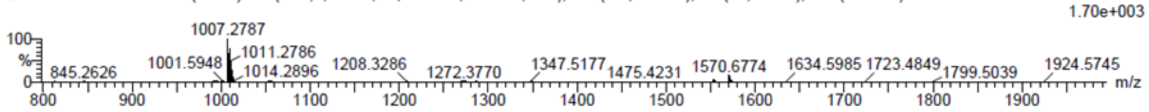

Minimum:				-1.5			
Maximum:		5.0	100.0	400.0			
Mass	Calc. Mass	mDa	PPM	DBE	i-FIT	i-FIT (Norm)	Formula
1007.2787	1007.2814	-2.7	-2.7	46.0	62.4	0.0	C65 H45 N5 O3 Zn

Fig. S69 MALDI-TOF-MS of cubane porphyrin monomer **24**.

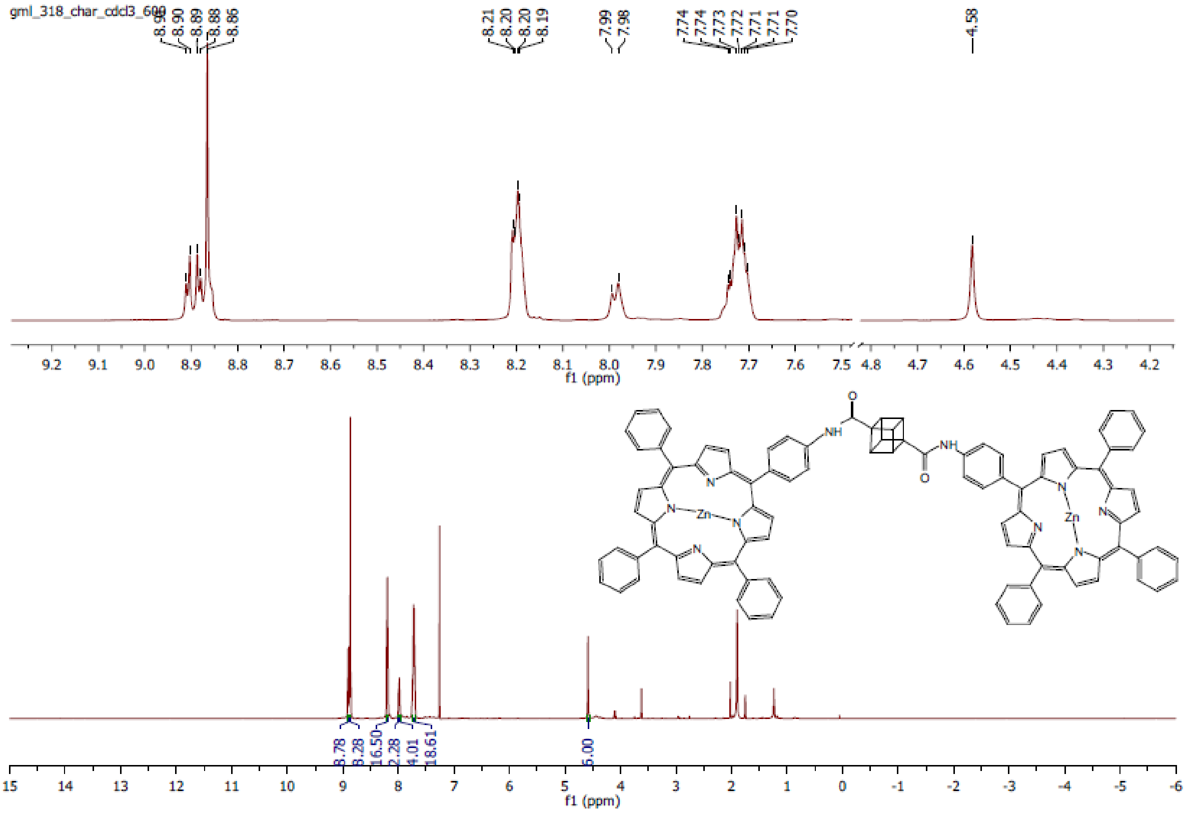

Fig. S70 ^{1}H NMR spectrum of cubane porphyrin dimer **25** in $CDCl_3$/THF-d_8.

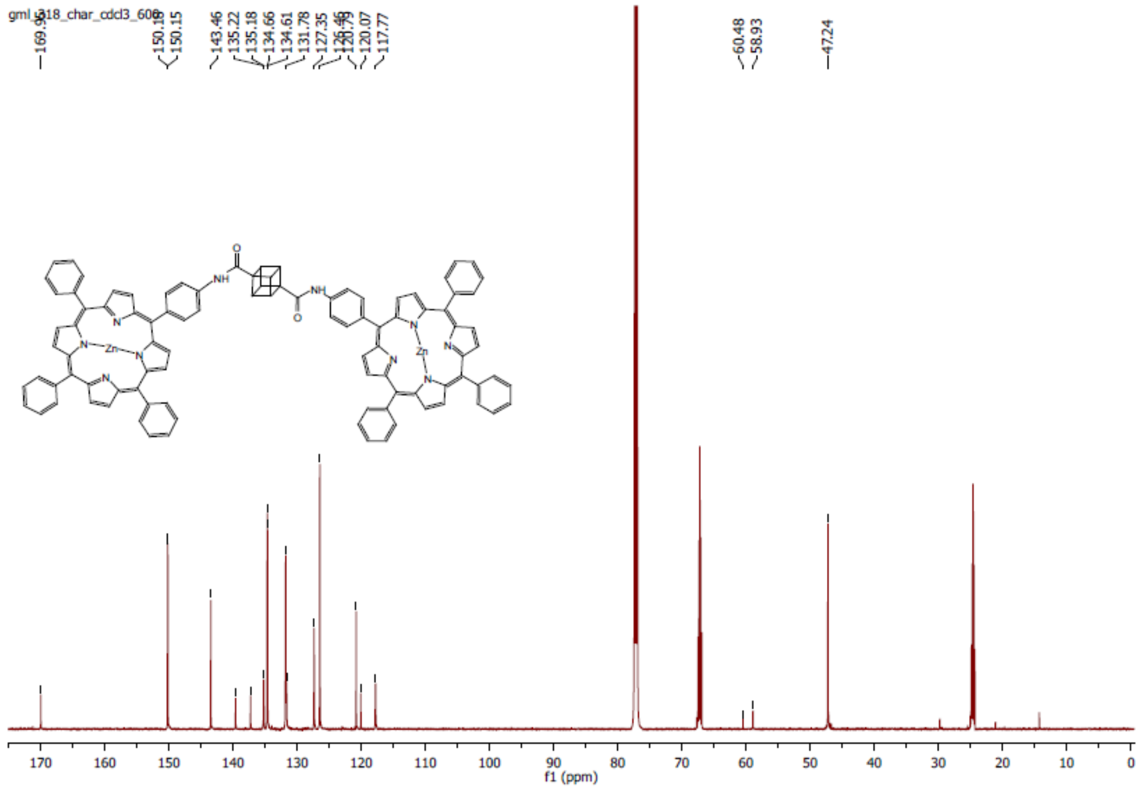

Fig. S71 ^{13}C NMR spectrum of cubane porphyrin dimer **25** in $CDCl_3$/THF-d_8.

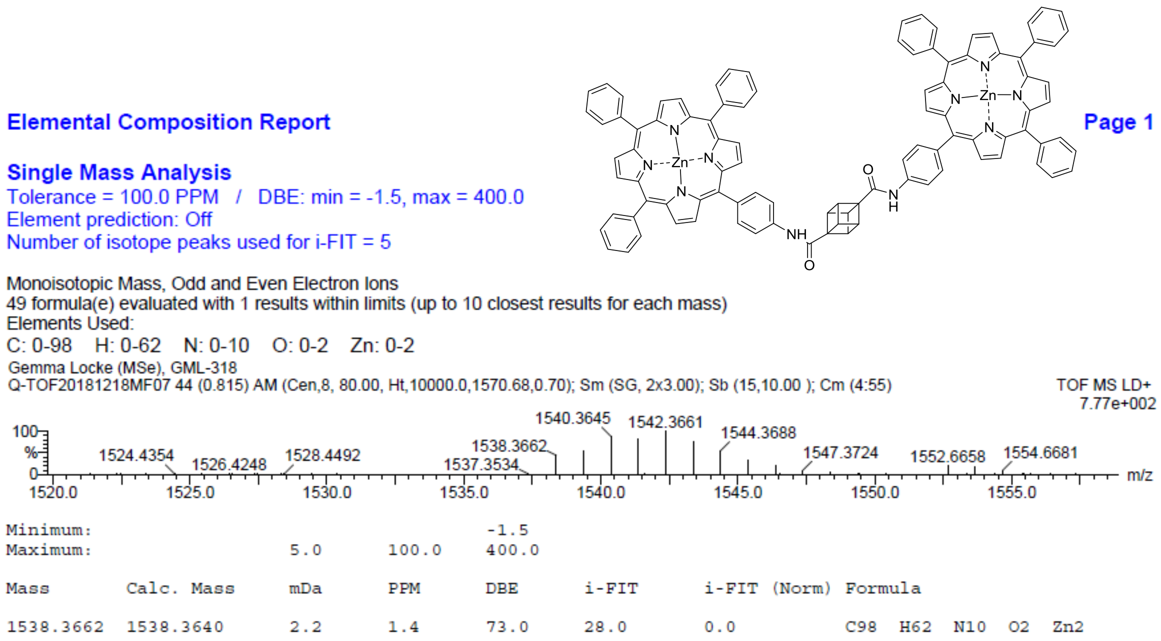

Fig. S72 MALDI-TOF-MS of cubane porphyrin dimer **25**.

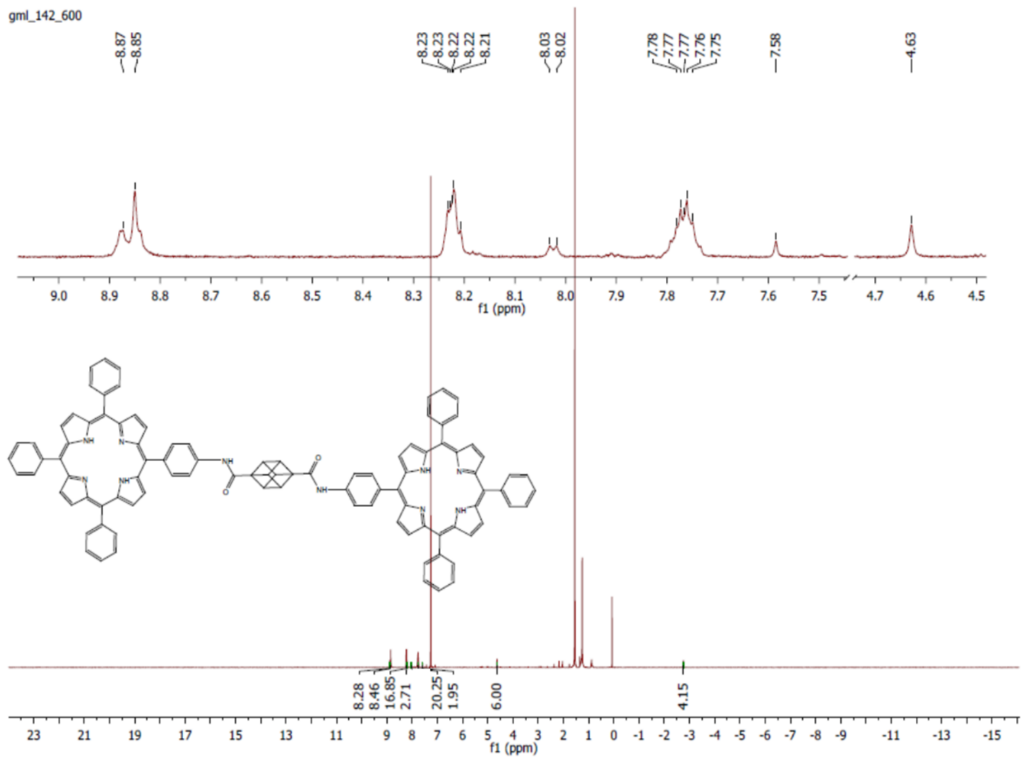

Fig S73 ^{1}H NMR of cubane porphyrin dimer **26** in $CDCl_3$.

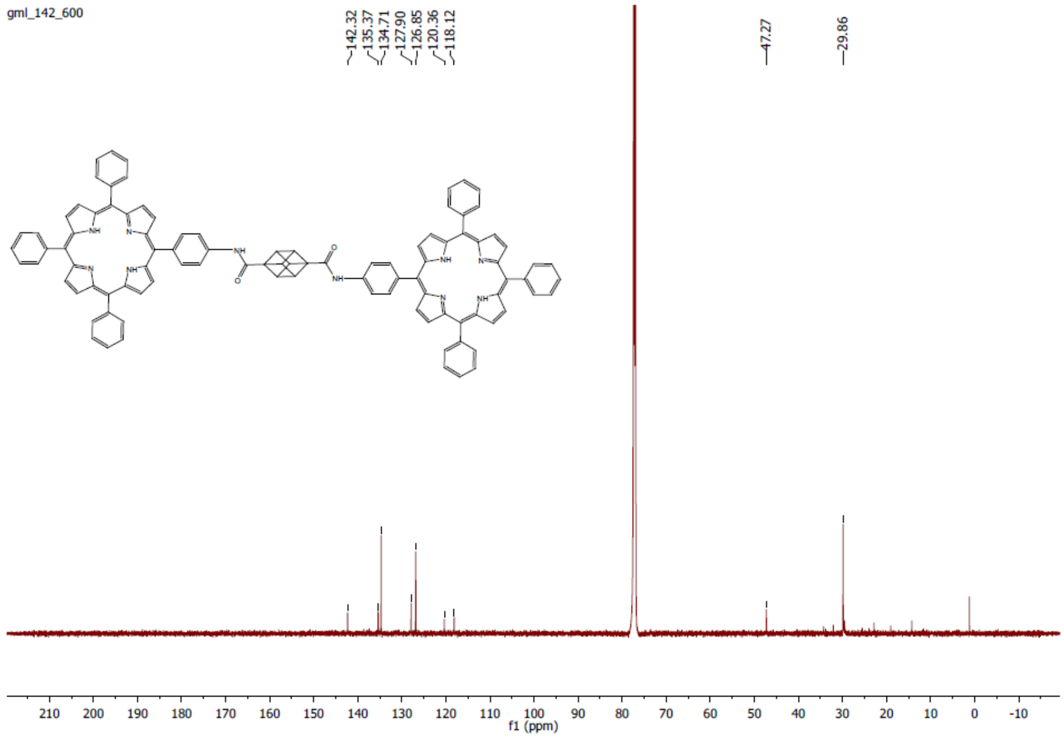

Fig. S74 ^{13}C NMR of cubane porphyrin dimer **26** in $CDCl_3$.

Elemental Composition Report

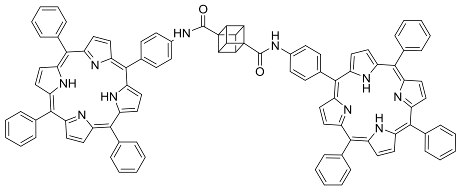

Page 1

Single Mass Analysis

Tolerance = 50.0 PPM / DBE: min = -1.5, max = 500.0
Element prediction: Off
Number of isotope peaks used for i-FIT = 3

Monoisotopic Mass, Odd and Even Electron Ions
90 formula(e) evaluated with 1 results within limits (up to 10 closest results for each mass)
Elements Used:
C: 0-98 H: 0-67 N: 0-10 O: 0-2 Pd: 0-1

Gemma Locke (MSe), GML-137-BIS-2H TPP-CUB DHB
Q-TOF20170130MF003 43 (0.793) AM (Cen,4, 80.00, Ht,10000.0,1570.68,0.70); Sm (SG, 1x3.00); Sb (15,10.00); Cm (8:92-(25:27+54:59)) TOF MS LD+ 9.31e+002

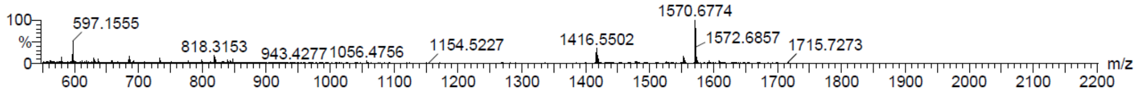

Minimum:				-1.5			
Maximum:		5.0	50.0	500.0			
Mass	Calc. Mass	mDa	PPM	DBE	i-FIT	i-FIT (Norm)	Formula
1415.5514	1415.5448	6.6	4.7	70.5	50.6	0.0	C98 H67 N10 O2

Fig. S75 MALDI-TOF-MS of cubane porphyrin dimer **26**.

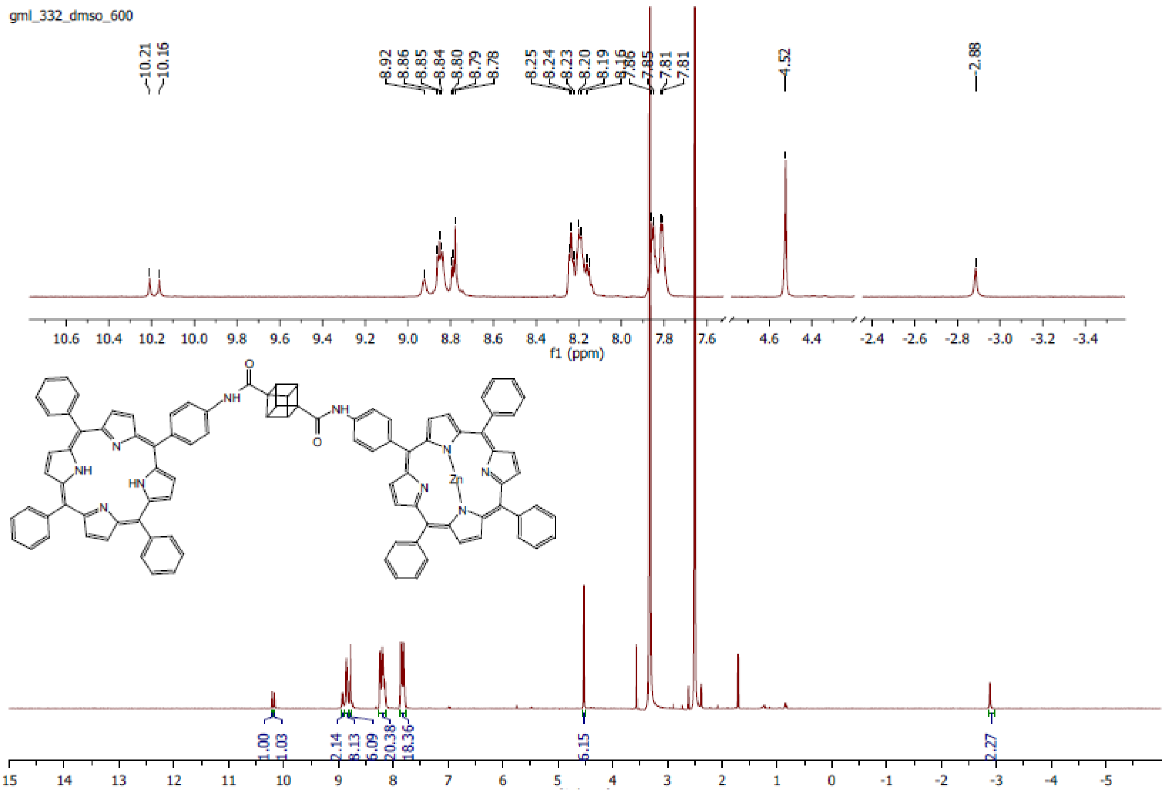

Fig. S76 ^{1}H NMR spectrum of cubane porphyrin dimer **27** in DMSO-d_6.

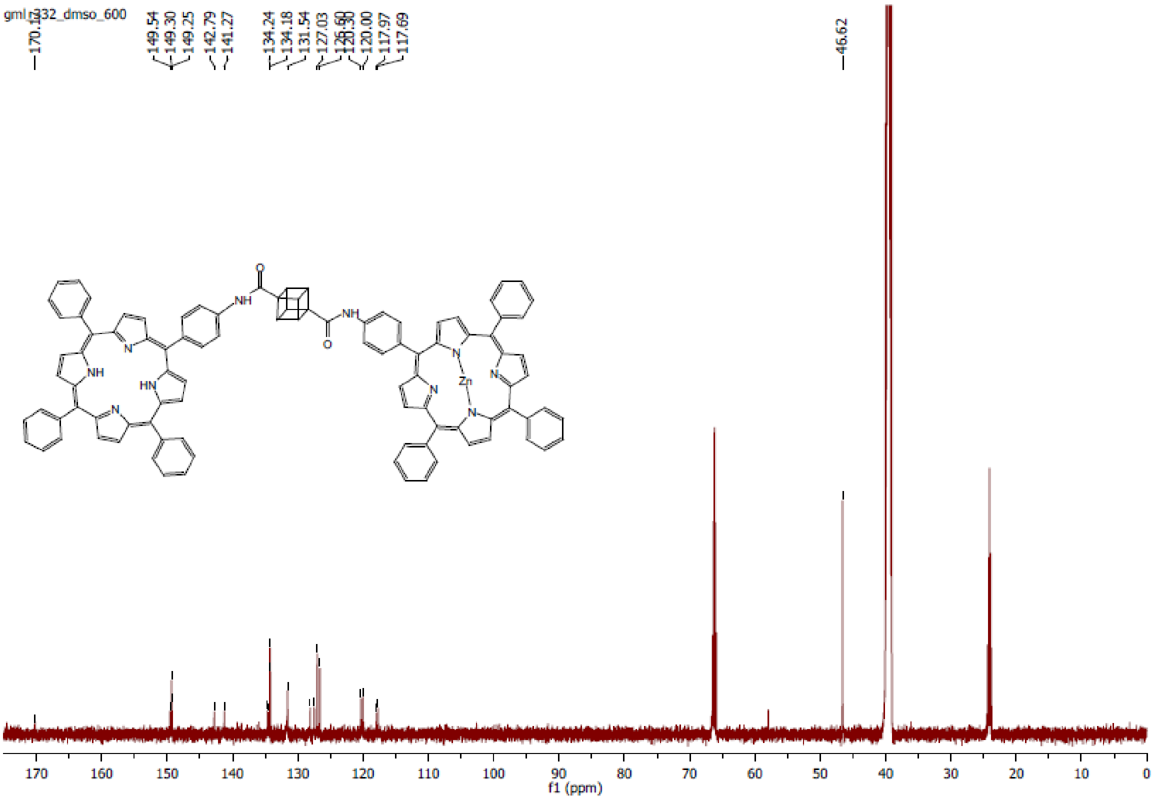

Fig. S77 ^{13}C NMR spectrum of cubane porphyrin dimer **27** in DMSO-d_6.

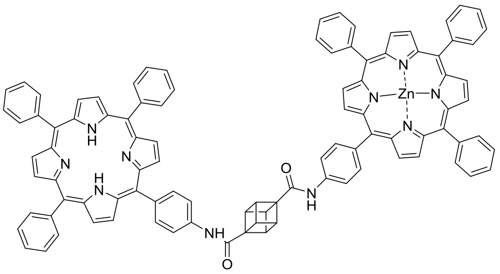

Elemental Composition Report Page 1

Single Mass Analysis
Tolerance = 100.0 PPM / DBE: min = -1.5, max = 400.0
Element prediction: Off
Number of isotope peaks used for i-FIT = 5

Monoisotopic Mass, Odd and Even Electron Ions
20 formula(e) evaluated with 1 results within limits (up to 10 closest results for each mass)
Elements Used:
C: 0-98 H: 0-64 N: 0-10 O: 0-2 Zn: 0-1

Gemma Locke (MSe), GML-332
Q-TOF20190218MF001 40 (0.741) AM (Cen,8, 80.00, Ht,10000.0,1570.68,0.70); Sm (SG, 2x3.00); Sb (15,10.00); Cm (3:63)
TOF MS LD+
1.79e+003

Minimum:				-1.5			
Maximum:		5.0	100.0	400.0			
Mass	Calc. Mass	mDa	PPM	DBE	i-FIT	i-FIT (Norm)	Formula
1476.4495	1476.4505	-1.0	-0.7	72.0	64.7	0.0	C98 H64 N10 O2 Zn

Fig. S78 MALDI-TOF-MS of cubane porphyrin dimer **27**.

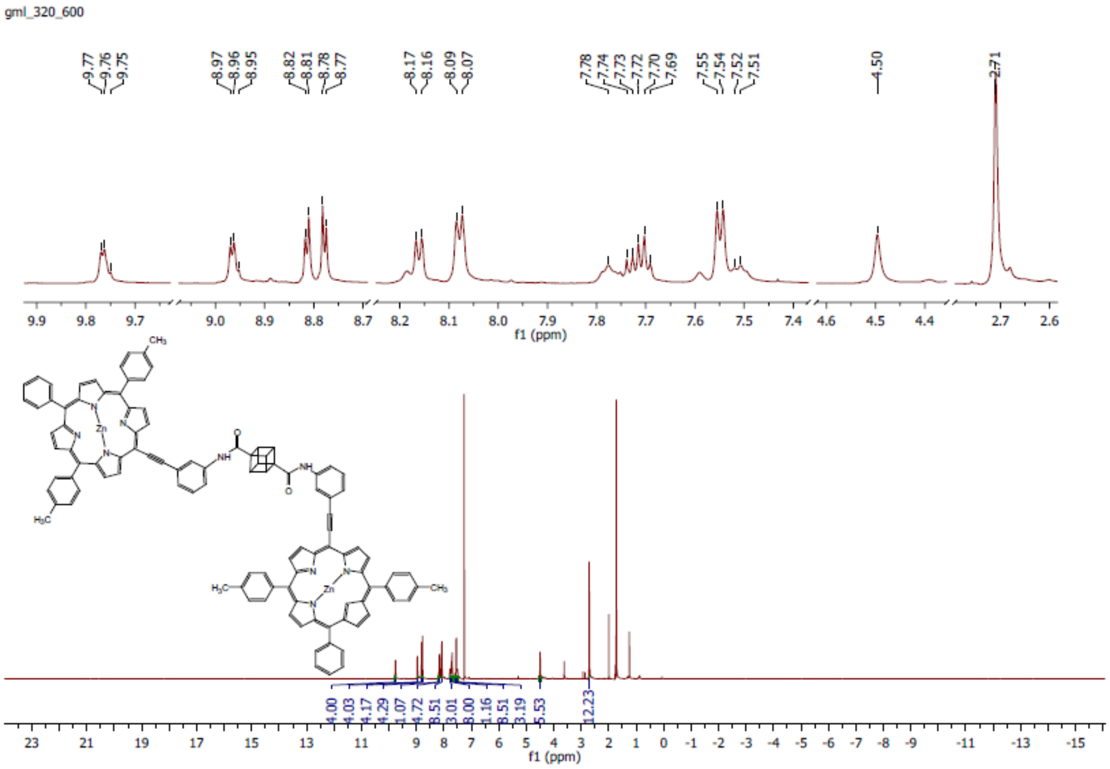

Fig. S79 ^{1}H NMR spectrum of cubane porphyrin dimer **28** in $CDCl_3$/THF-d_8.

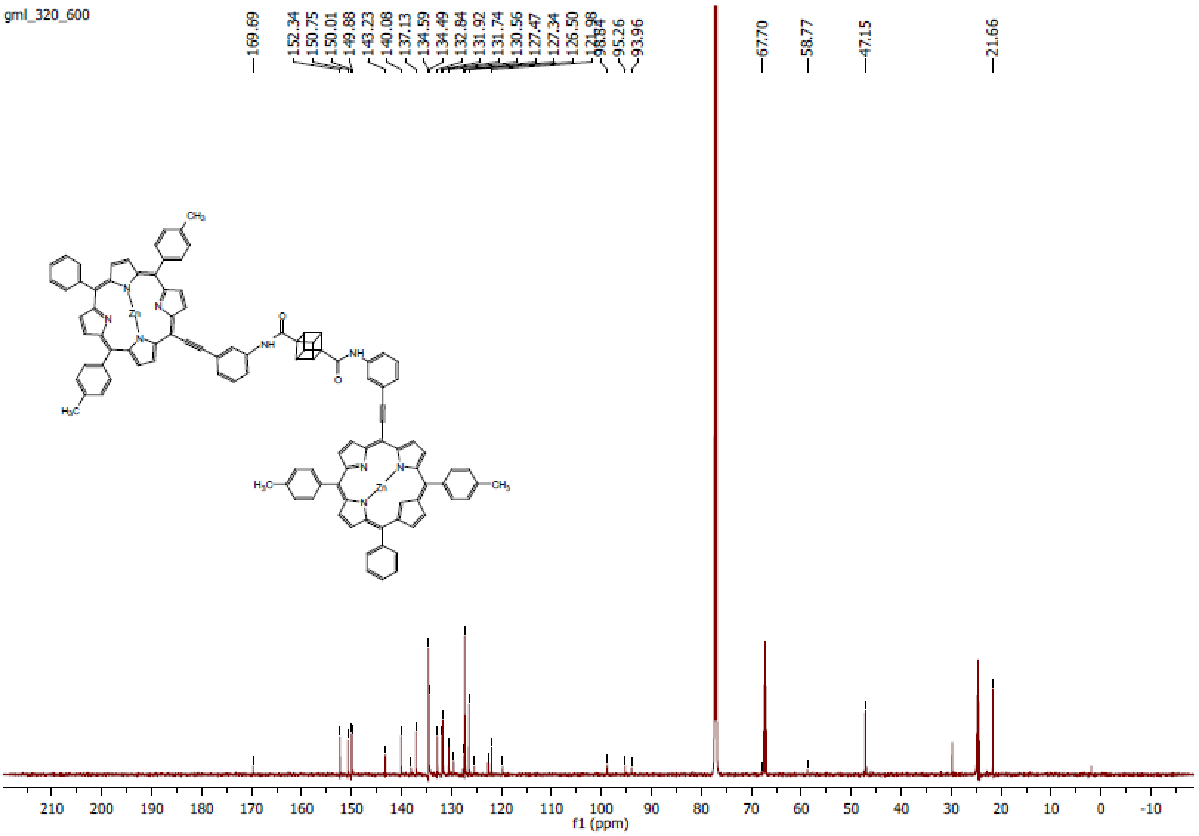

Fig. S80 ^{13}C NMR spectrum of cubane porphyrin dimer **28** in $CDCl_3$/THF-d_8.

Elemental Composition Report **Page 1**

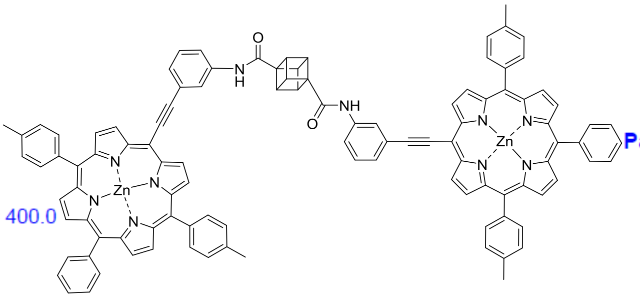

Single Mass Analysis
Tolerance = 100.0 PPM / DBE: min = -1.5, max = 400.0
Element prediction: Off
Number of isotope peaks used for i-FIT = 5

Monoisotopic Mass, Odd and Even Electron Ions
46 formula(e) evaluated with 1 results within limits (up to 10 closest results for each mass)
Elements Used:
C: 0-106 H: 0-70 N: 0-10 O: 0-2 Zn: 0-2
Gemma Locke (MSe), GML-320
Q-TOF2019016MF001 58 (1.074) AM (Cen,8, 80.00, Ht,10000.0,1570.68,0.70); Sm (SG, 2x3.00); Sb (15,10.00); Cm (8:83) TOF MS LD+ 3.67e+003

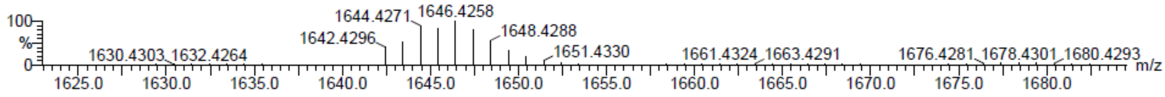

Minimum:				-1.5			
Maximum:		5.0	100.0	400.0			
Mass	Calc. Mass	mDa	PPM	DBE	i-FIT	i-FIT (Norm)	Formula
1642.4296	1642.4266	3.0	1.8	77.0	89.0	0.0	C106 H70 N10 O2 Zn2

Fig. S81 MALDI-TOF-MS of cubane porphyrin dimer **28**.

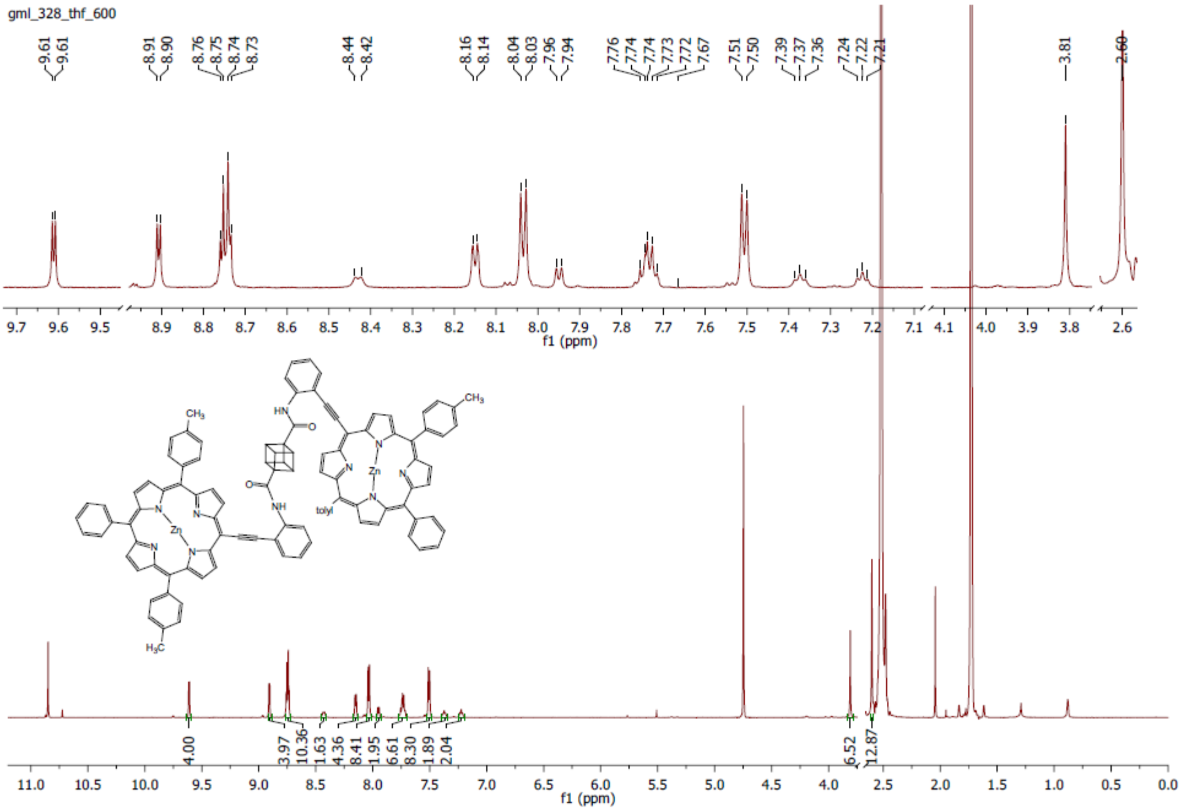

Fig. S82 ^{1}H NMR spectrum of cubane porphyrin dimer **29** in THF-d_8.

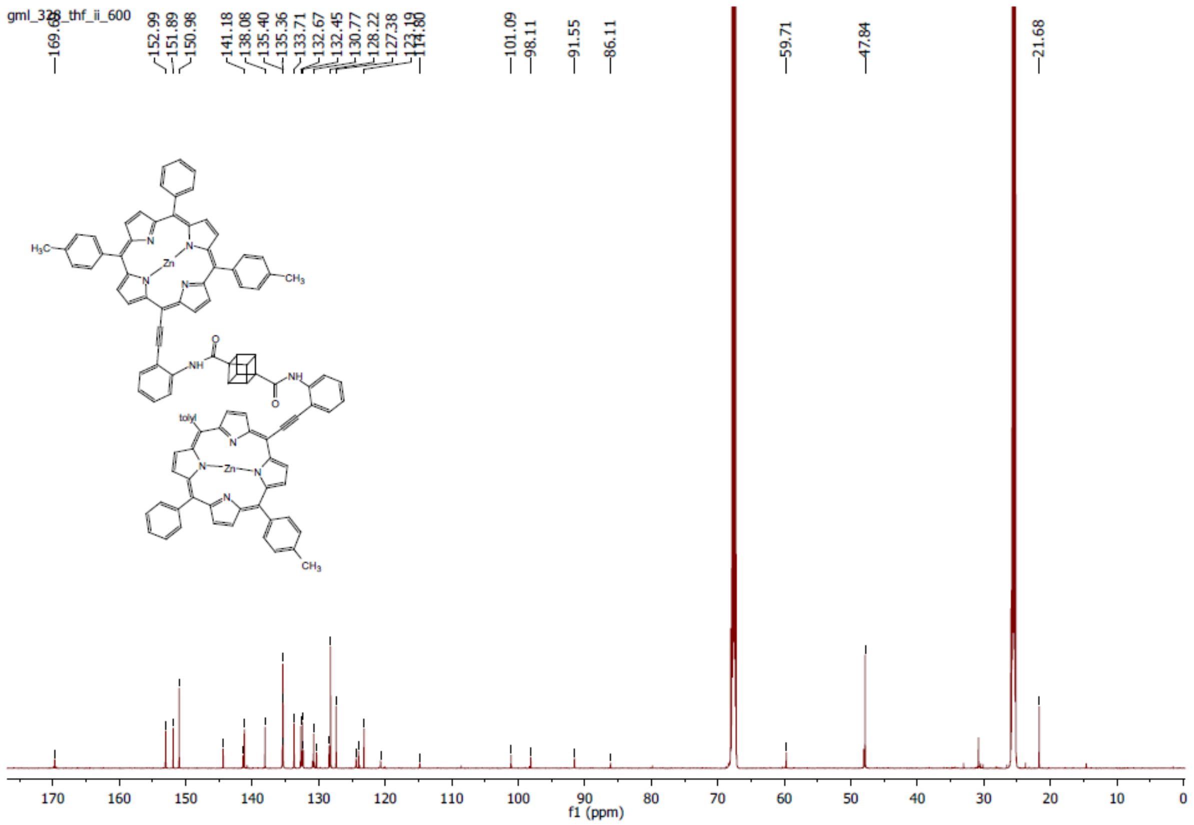

Fig. S83 ^{13}C NMR spectrum of cubane porphyrin dimer **29** in THF-d$_8$.

Elemental Composition Report

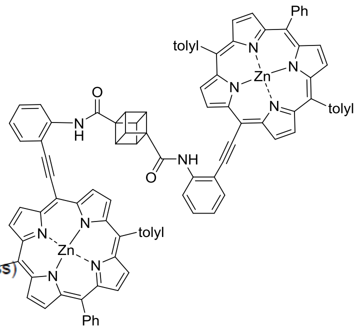

Page 1

Single Mass Analysis

Tolerance = 100.0 PPM / DBE: min = -1.5, max = 400.0

Element prediction: Off

Number of isotope peaks used for i-FIT = 5

Monoisotopic Mass, Odd and Even Electron Ions

46 formula(e) evaluated with 1 results within limits (up to 10 closest results for each mass)

Elements Used:

C: 0-106 H: 0-70 N: 0-10 O: 0-2 Zn: 0-2

Gemma Locke (MSe), GML-328

Q-TOF20190123MF009 37 (0.685) AM (Cen,8, 80.00, Ht,10000.0,1570.68,0.70); Sm (SG, 2x3.00); Sb (15,10.00); Cm (10:53) TOF MS LD+ 1.68e+003

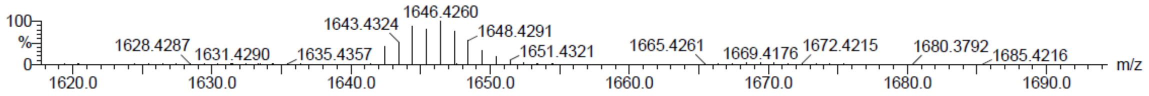

Minimum:				-1.5			
Maximum:		5.0	100.0	400.0			
Mass	Calc. Mass	mDa	PPM	DBE	i-FIT	i-FIT (Norm)	Formula
1642.4304	1642.4266	3.8	2.3	77.0	90.7	0.0	C106 H70 N10 O2 Zn2

Fig. S84 MALDI-TOF-MS of cubane porphyrin dimer **29**.

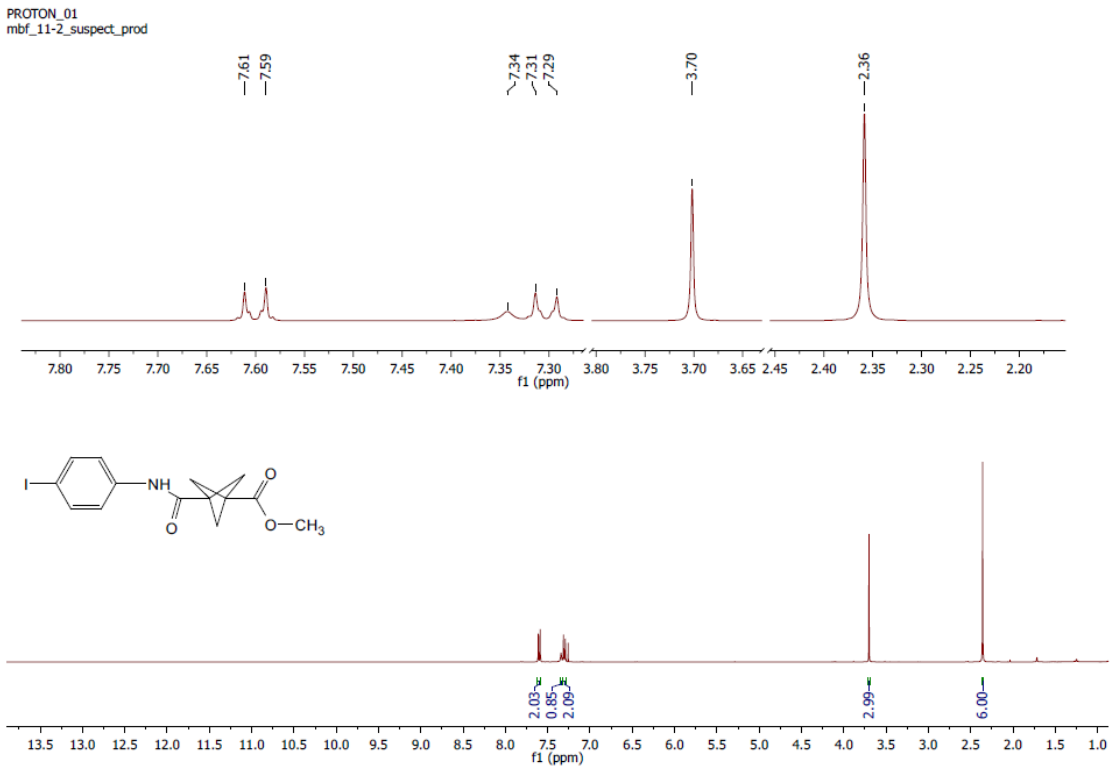

Fig. S85 ^{1}H NMR spectrum of BCP **32** in $CDCl_3$.

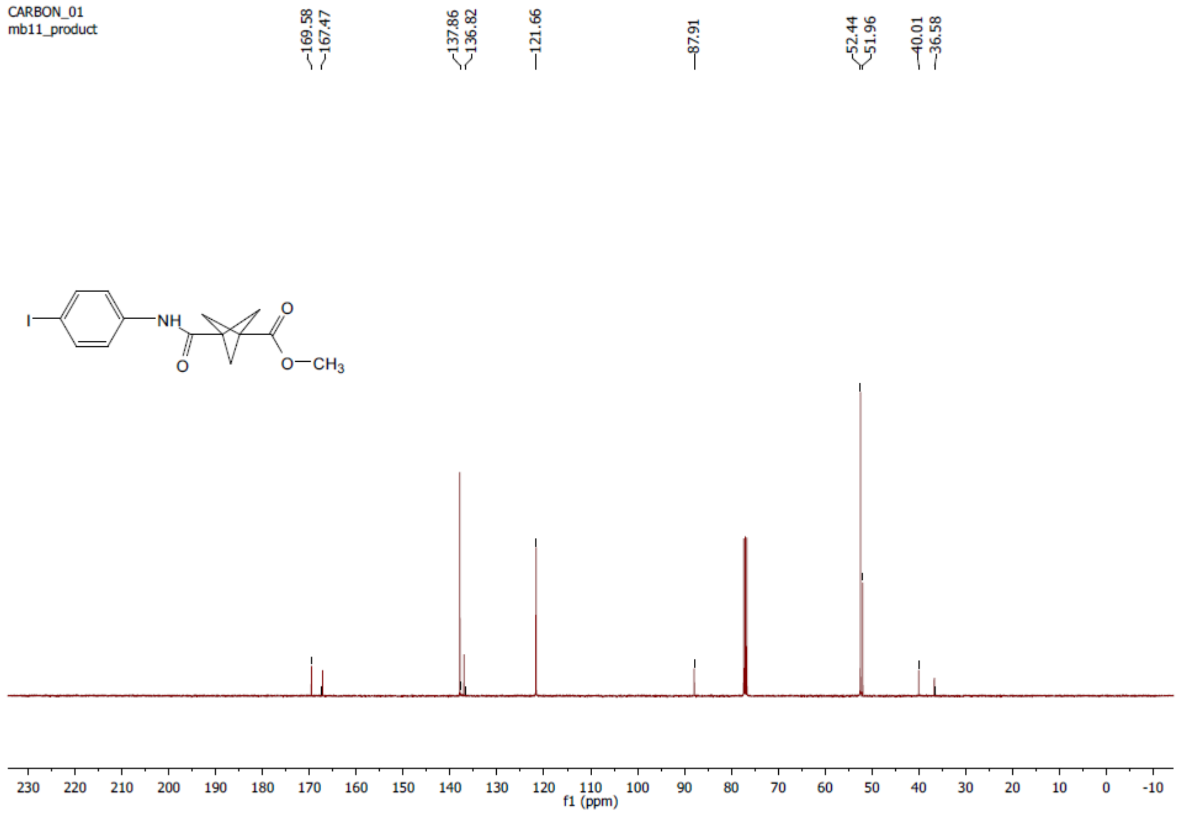

Fig. S86 ^{13}C NMR spectrum of BCP **32** in $CDCl_3$.

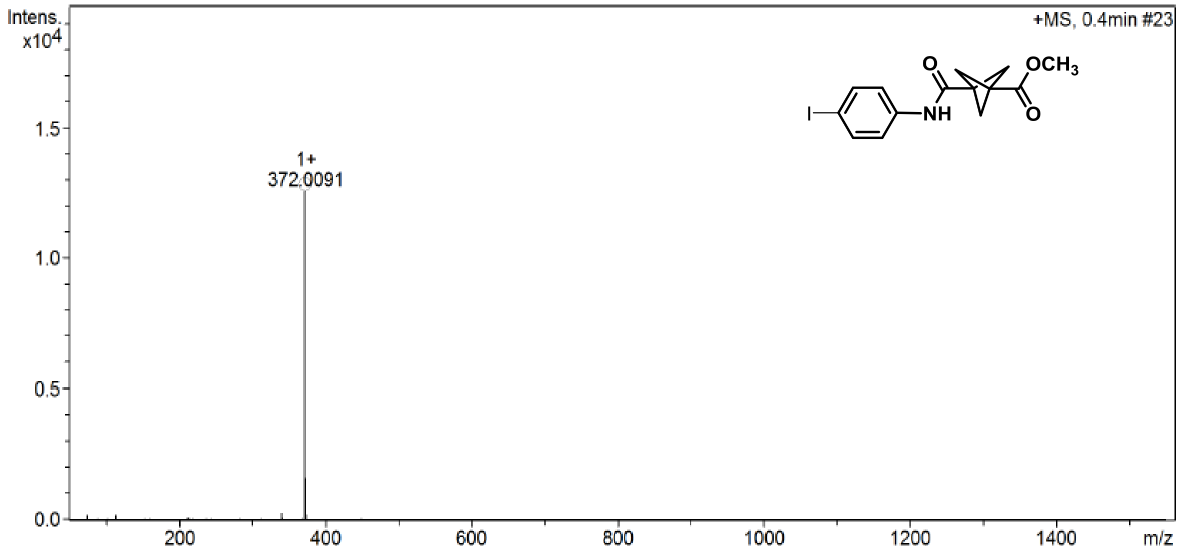

Meas. m/z	#	Ion Formula	m/z	err [mDa]	err [ppm]	rdb	N-Rule	e⁻ Conf	mSigma
372.009106	1	C14H15INO3	372.009118	0.0	0.0	7.5	ok	even	17.5

Fig. S87 HRMS (APCI) of BCP **32**.

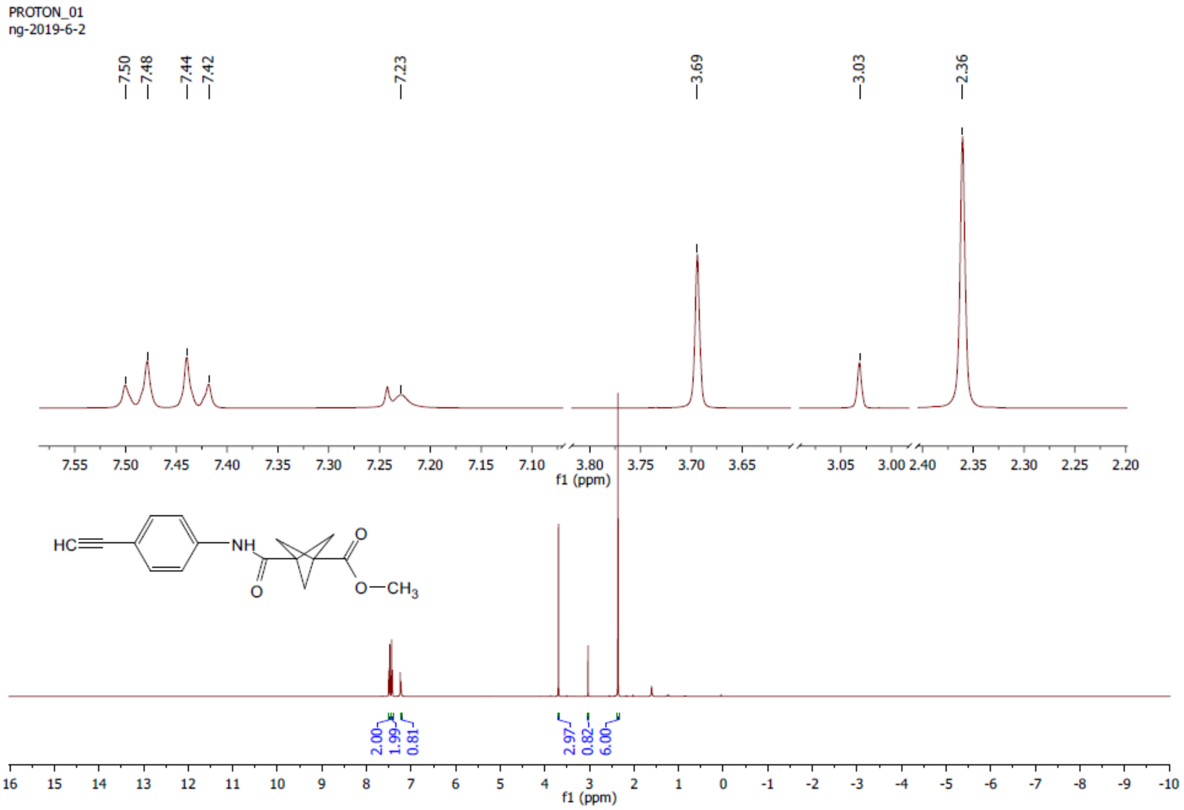

Fig. S88 ^{1}H NMR spectrum of BCP **33** in $CDCl_3$.

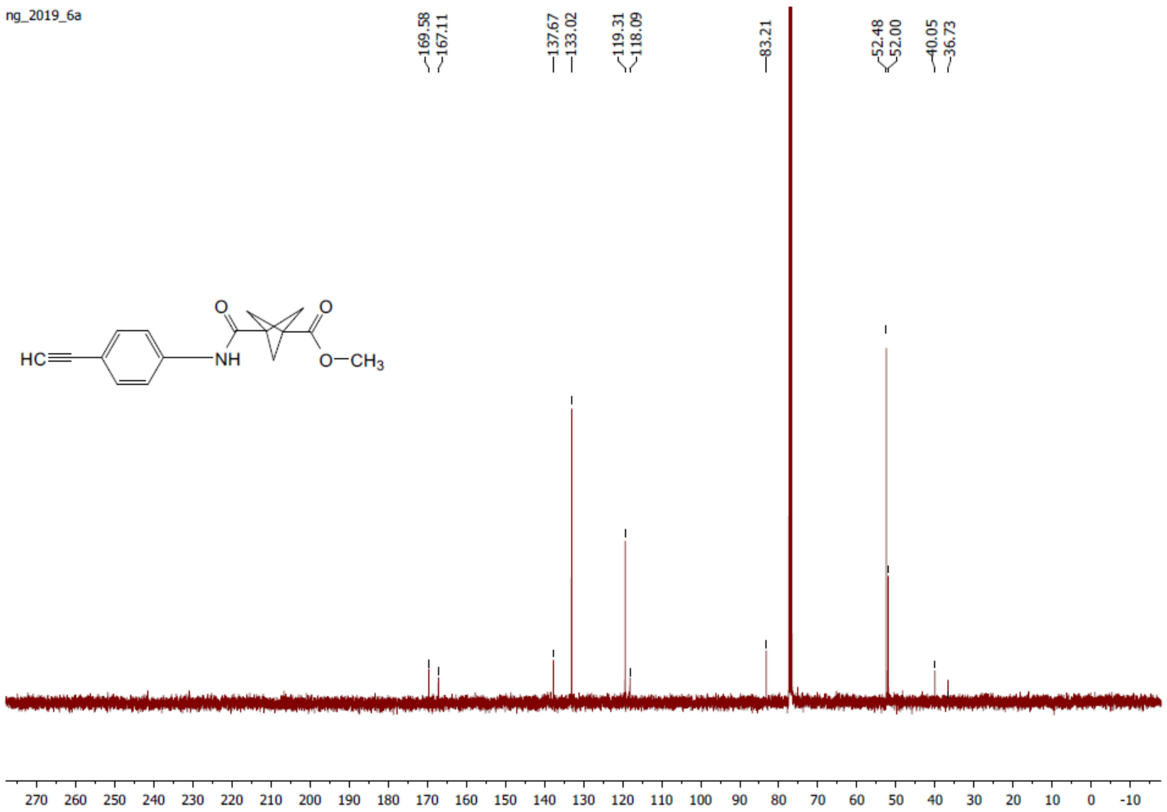

Fig. S89 ^{13}C NMR spectrum of BCP **33** in $CDCl_3$.

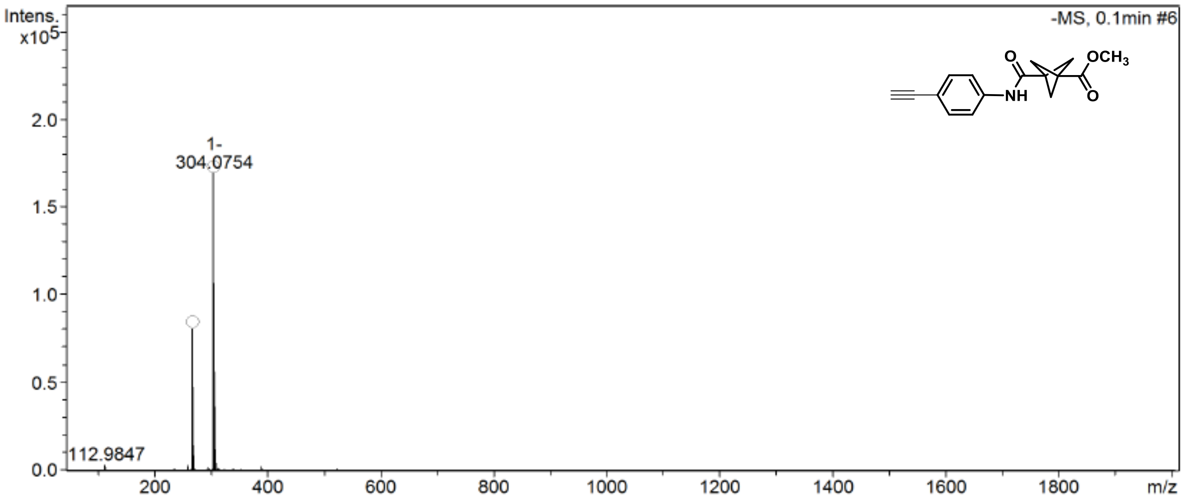

Meas. m/z	#	Ion Formula	m/z	err [mDa]	err [ppm]	rdb	N-Rule	e⁻ Conf	mSigma
268.098463	1	C16H14NO3	268.097917	0.5	2.0	10.5	ok	even	2.4
304.075412	1	C16H15ClNO3	304.074595	-0.8	-2.7	9.5	ok	even	3.5

Fig. S90 HRMS (APCI) of BCP **33**.

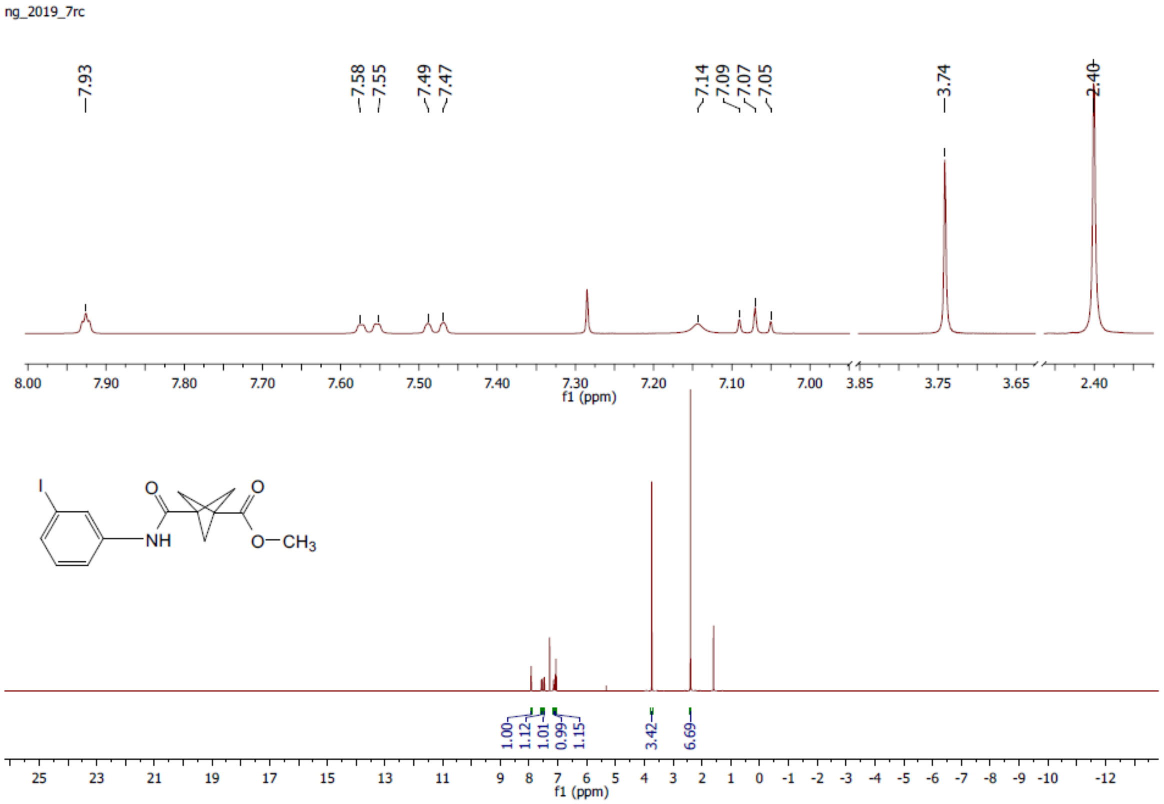

Fig. S91 ^{1}H NMR spectrum of BCP **34** in $CDCl_3$.

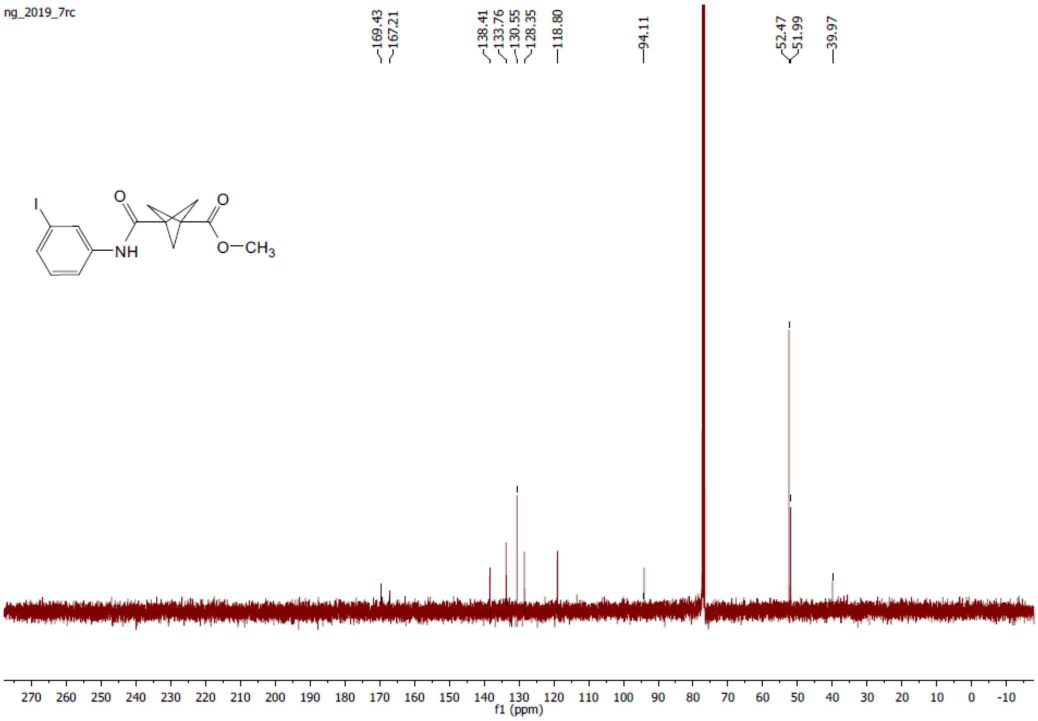

Fig. S92 ^{13}C NMR spectrum of BCP **34** in $CDCl_3$.

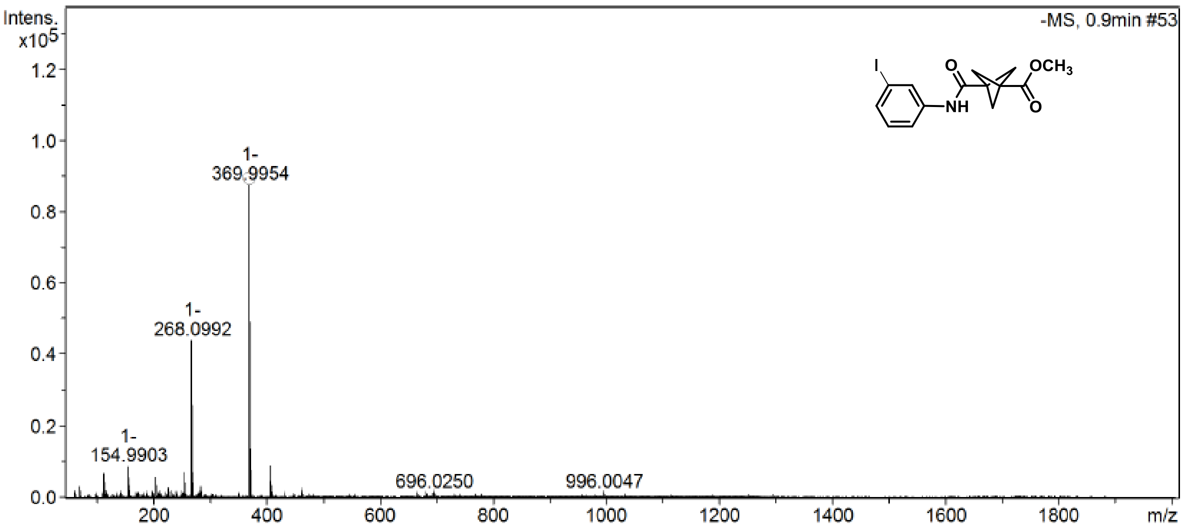

Meas. m/z	#	Ion Formula	m/z	err [mDa]	err [ppm]	rdb	N-Rule	e⁻ Conf	mSigma
369.995415	1	C14H13INO3	369.994565	-0.9	-2.3	8.5	ok	even	2.2

Fig. S93 HRMS (APCI) of BCP **34**.

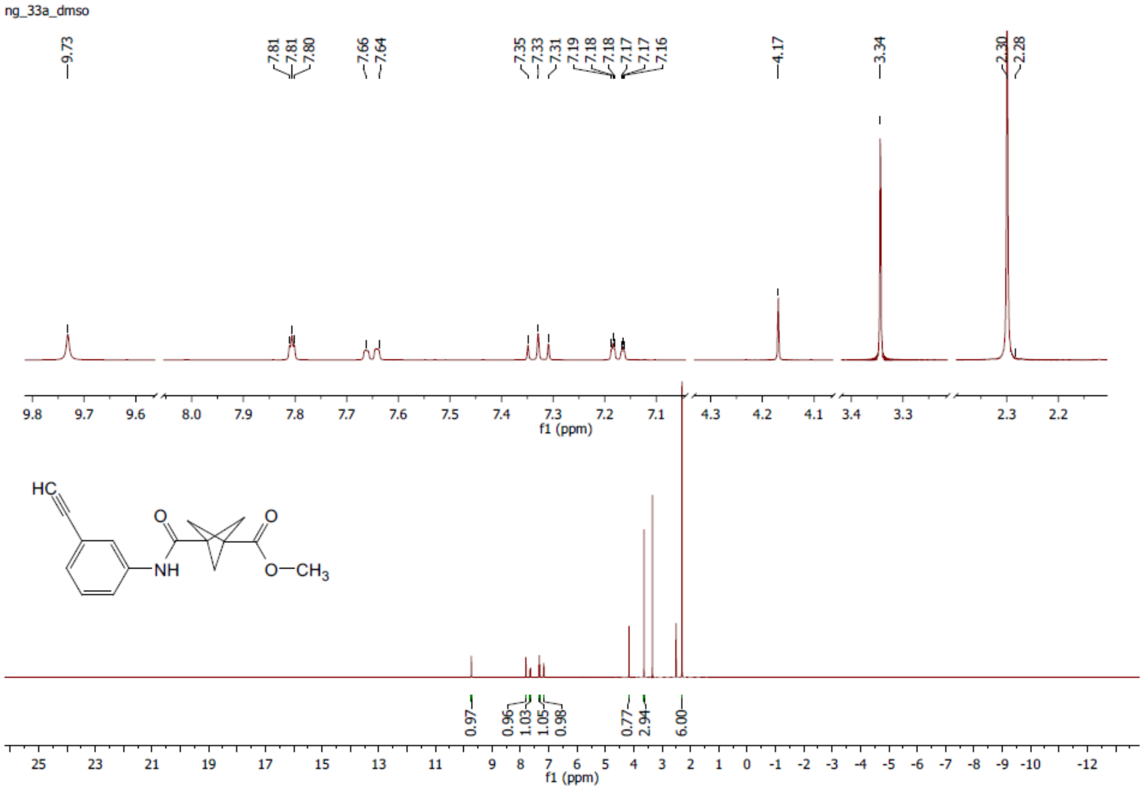

Fig. S94 ^{1}H NMR spectrum of BCP **35** in DMSO-d$_6$.

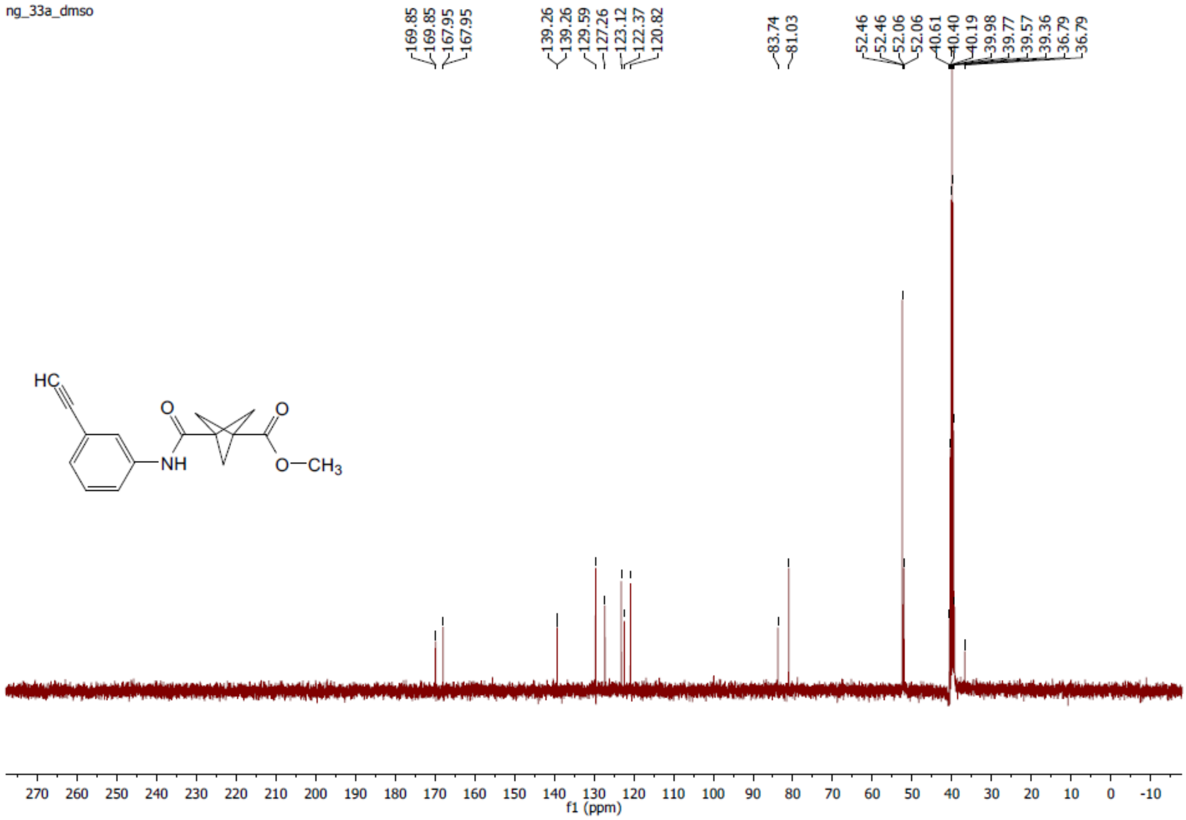

Fig. S95 ^{13}C NMR spectrum of BCP **35** in DMSO-d_6.

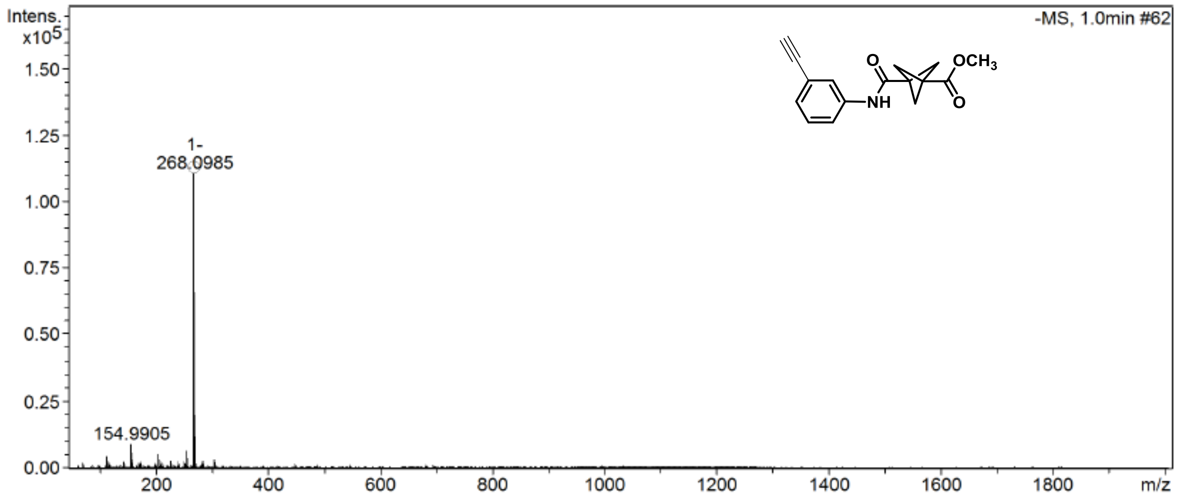

Meas. m/z	#	Ion Formula	m/z	err [mDa]	err [ppm]	rdb	N-Rule	e⁻ Conf	mSigma
268.098494	1	C16H14NO3	268.097917	0.6	2.2	10.5	ok	even	1.5

Fig. S96 HRMS (APCI) of BCP **35**.

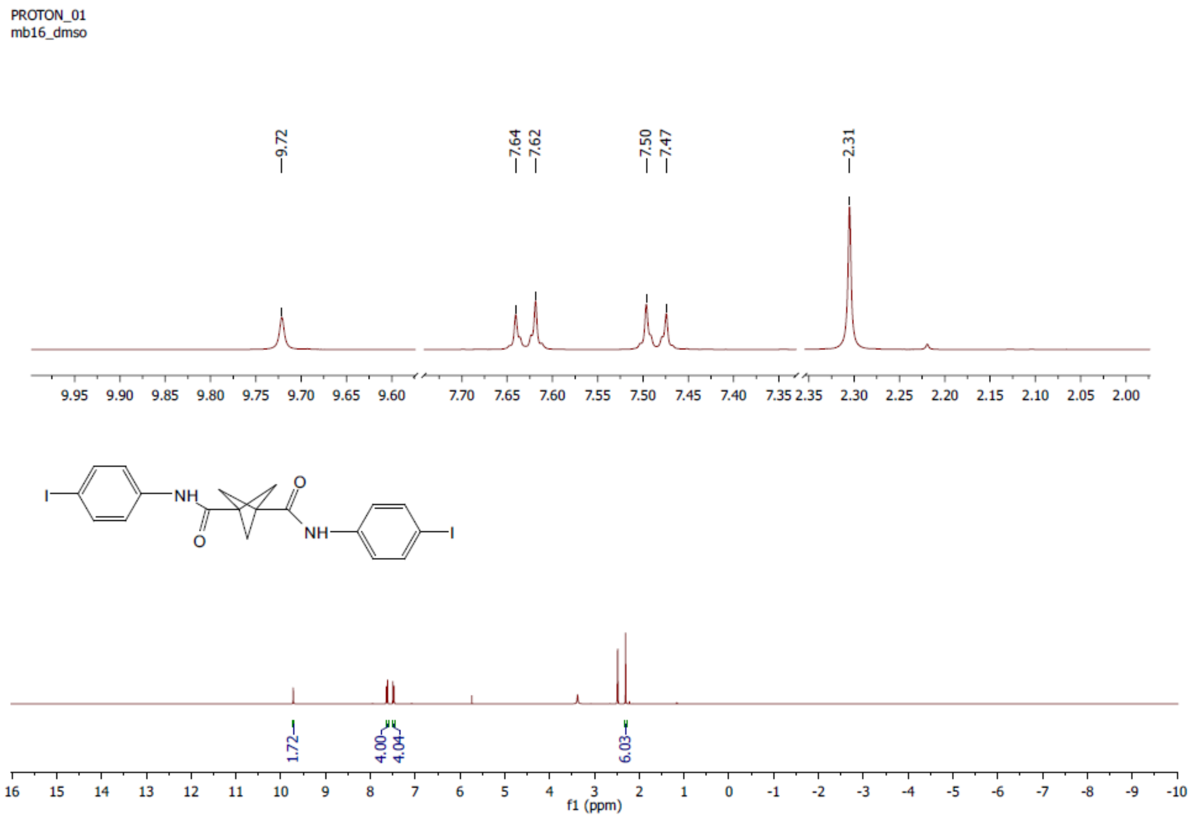

Fig. S97 ^{1}H NMR spectrum of BCP **36** in DMSO-d_6.

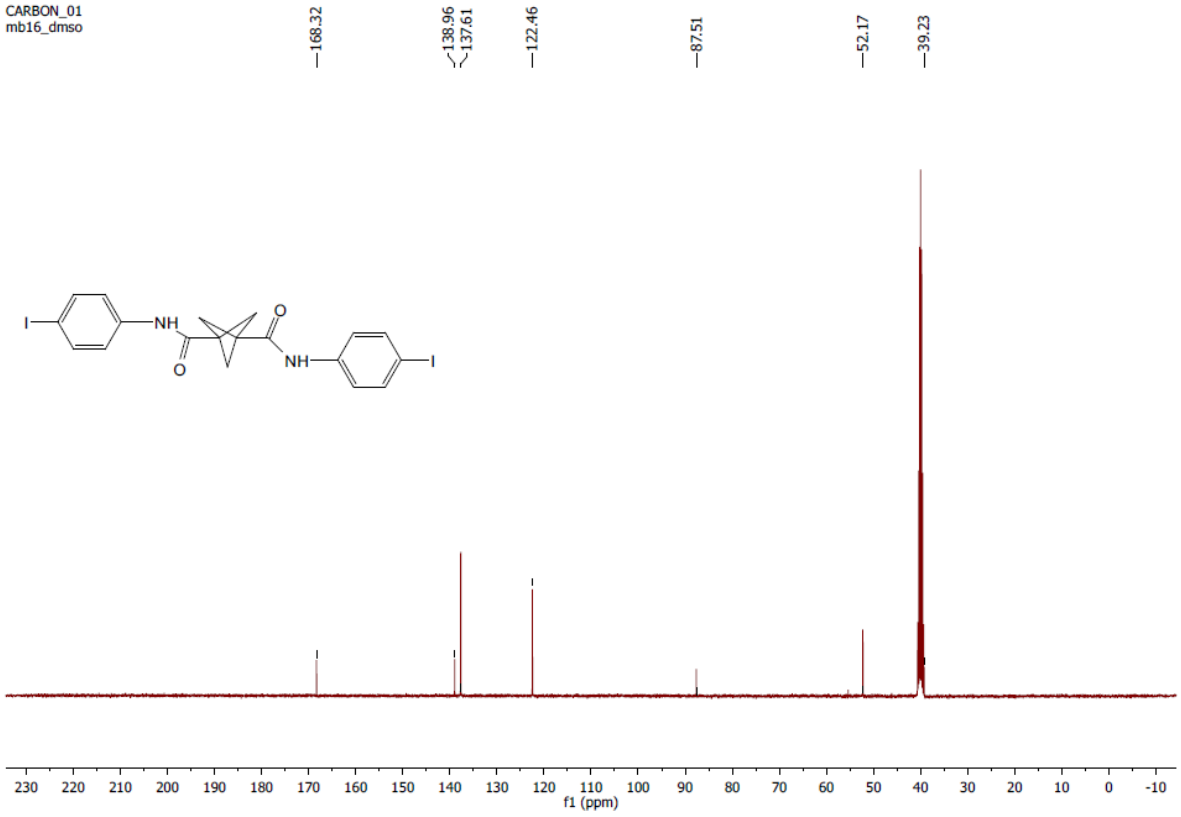

Fig. S98 ^{13}C NMR spectrum of BCP **36** in DMSO-d_6.

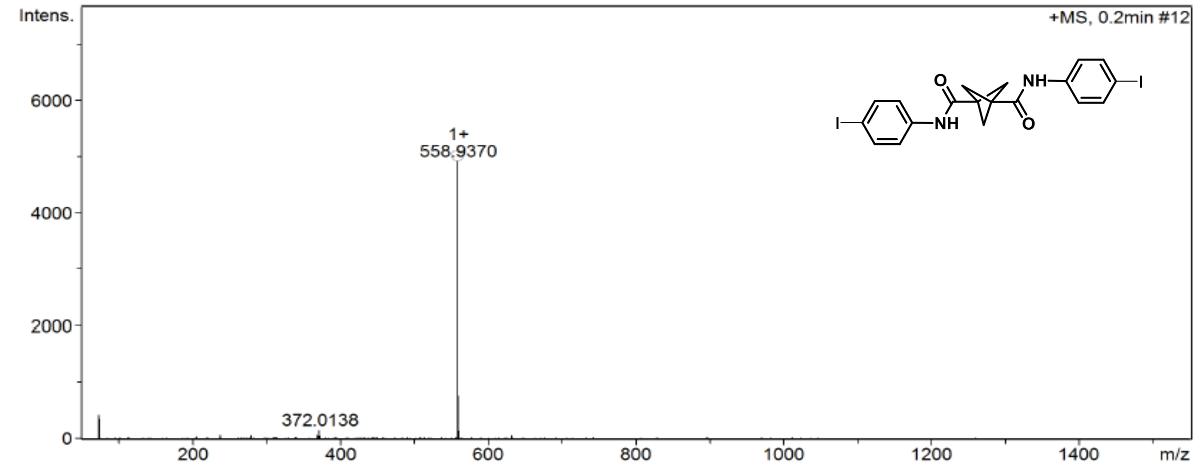

Meas. m/z	#	Ion Formula	m/z	err [mDa]	err [ppm]	rdb	N-Rule	e⁻ Conf	mSigma
558.936967	1	C19H17I2N2O2	558.937400	0.4	0.8	11.5	ok	even	34.8

Fig. S99 HRMS (APCI) of BCP **36**.

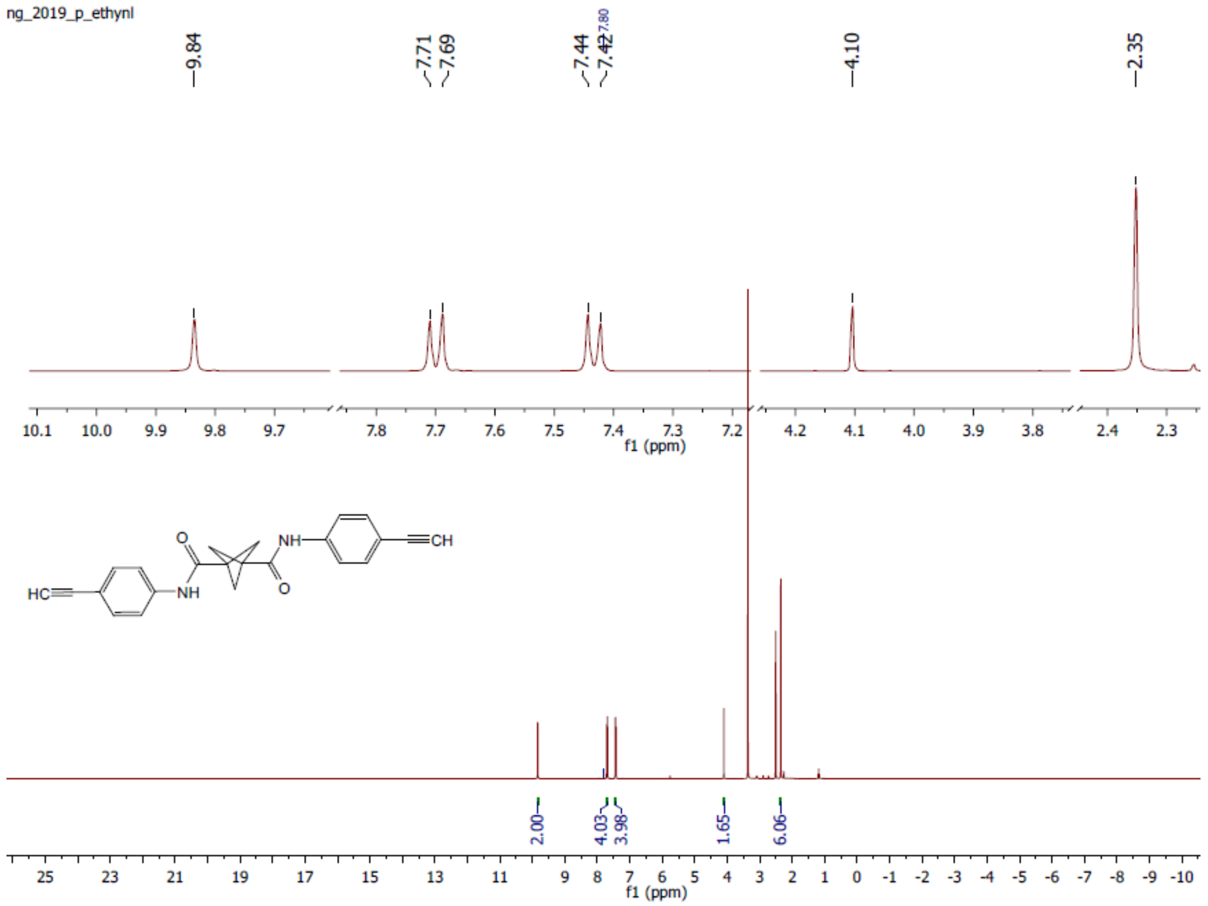

Fig. S100 ^{1}H NMR spectrum of BCP **37** in DMSO-d_6.

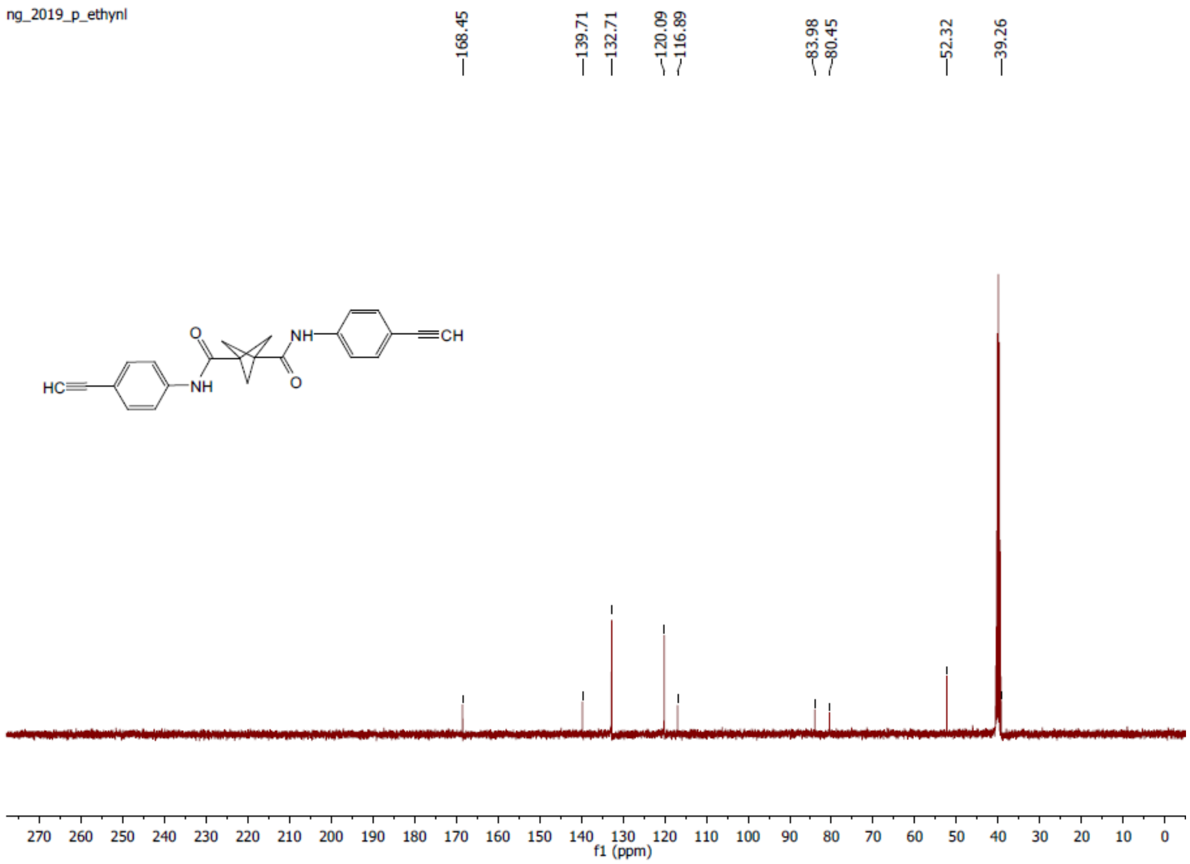

Fig. S101 ^{13}C NMR spectrum of BCP **37** in DMSO-d_6.

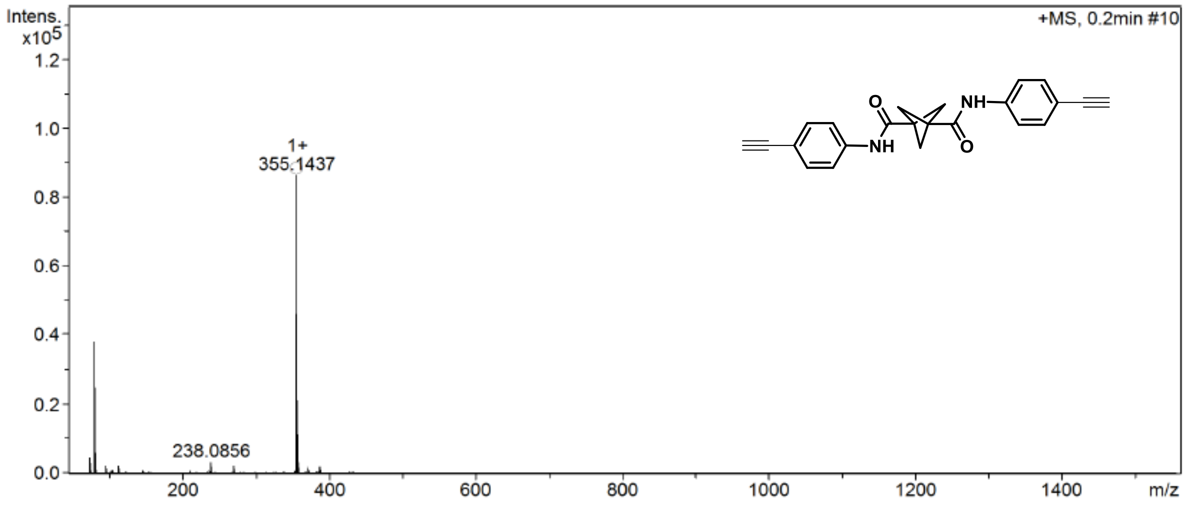

Meas. m/z	#	Ion Formula	m/z	err [mDa]	err [ppm]	rdb	N-Rule	e⁻ Conf	mSigma
355.143670	1	C23H19N2O2	355.144104	-0.4	-1.2	15.5	ok	even	5.4

Fig. S102 HRMS (APCI) of BCP **37**.

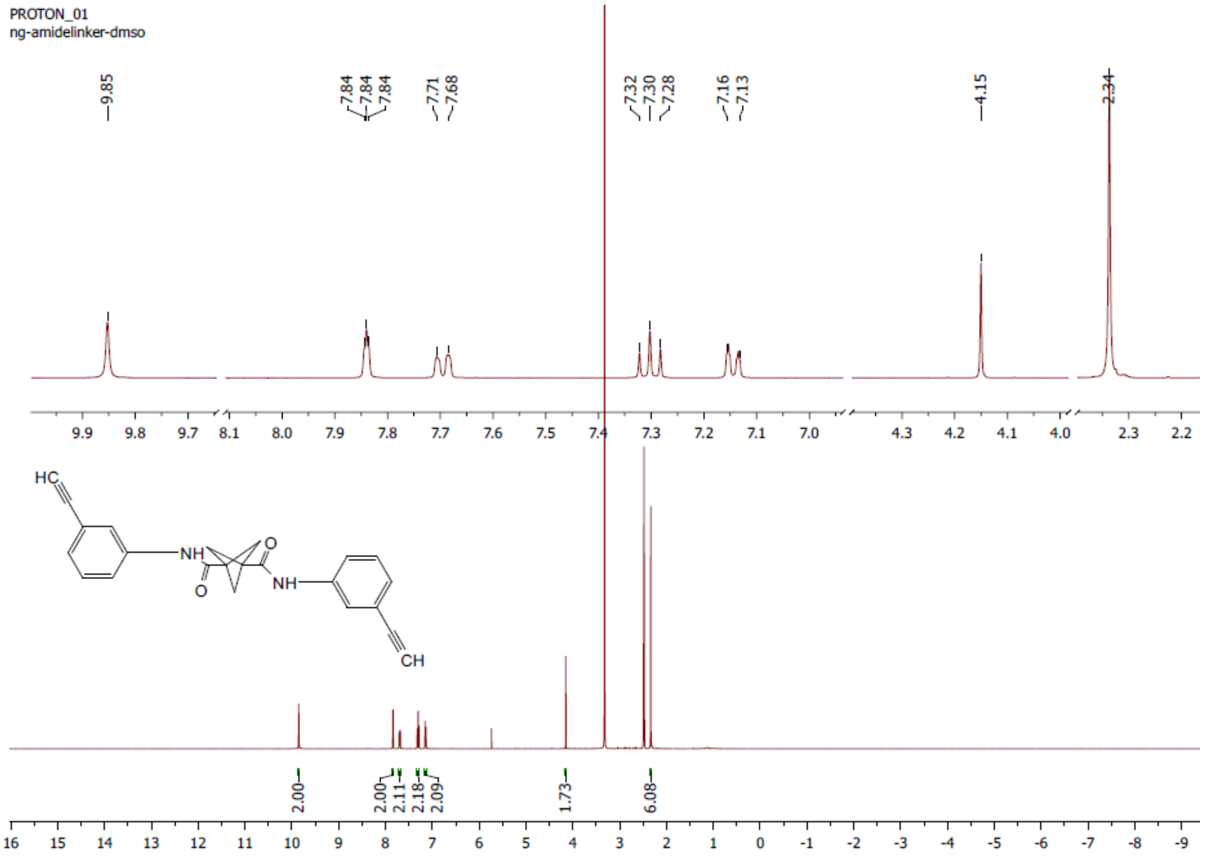

Fig. S103 ^{1}H NMR spectrum of BCP **38** in DMSO-d_6.

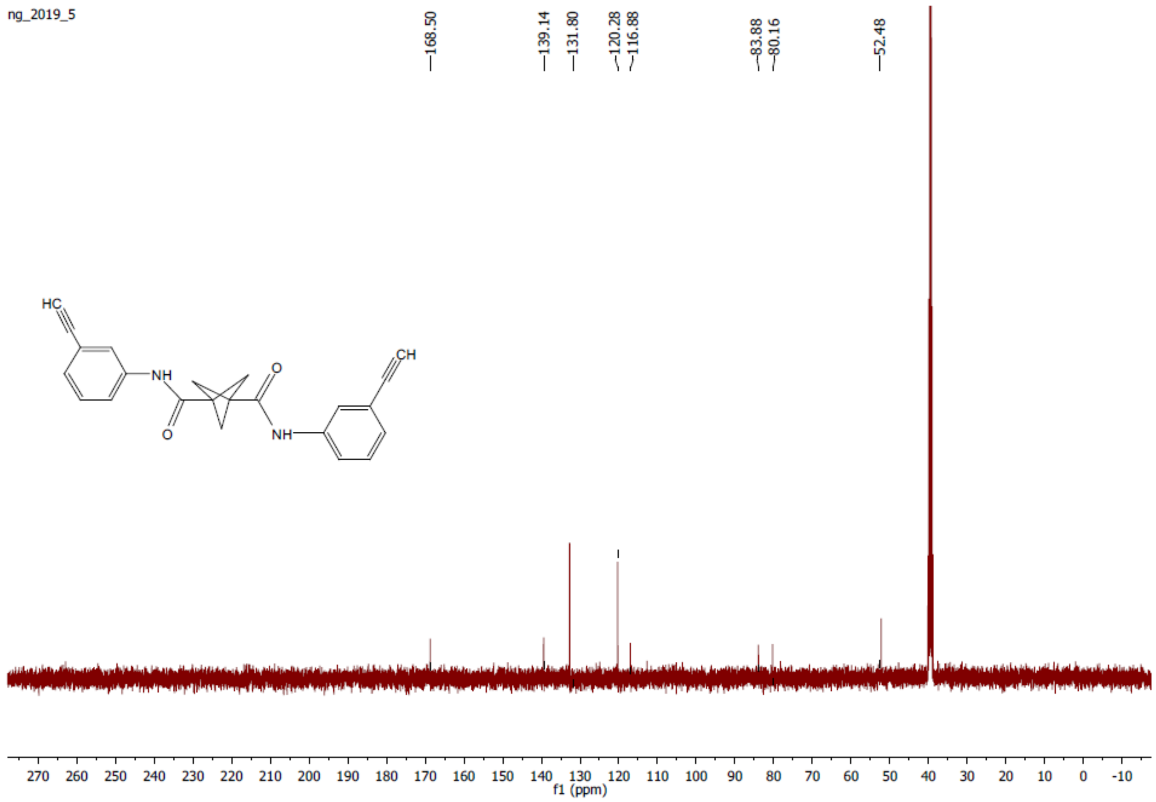

Fig. S104 ^{13}C NMR spectrum of BCP **38** in DMSO-d_6

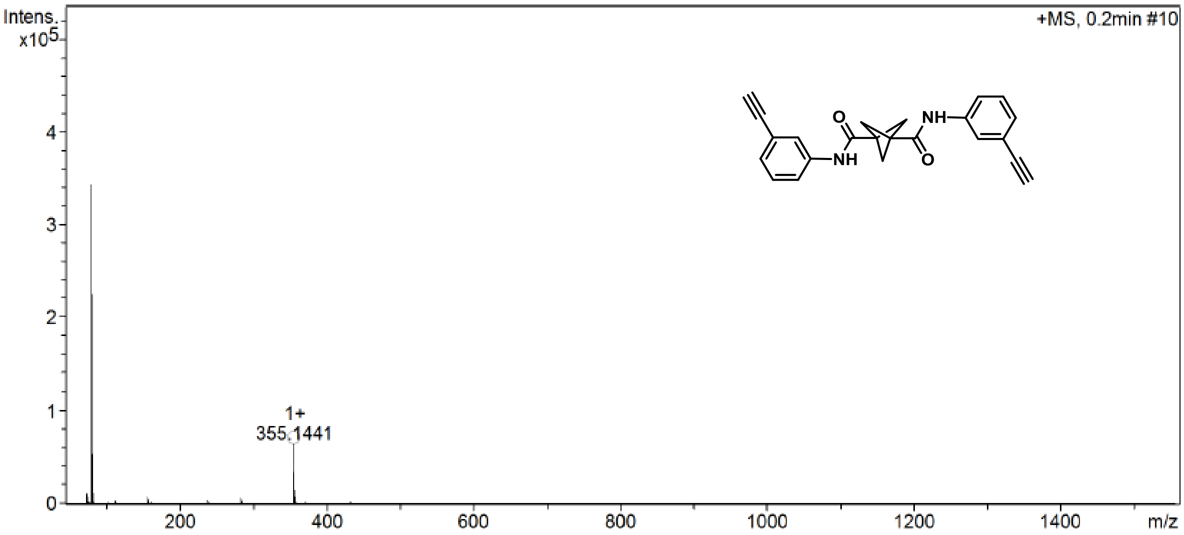

Meas. m/z	#	Ion Formula	m/z	err [mDa]	err [ppm]	rdb	N-Rule	e⁻ Conf	mSigma
355.144130	1	C23H19N2O2	355.144104	0.0	0.1	15.5	ok	even	8.0

Fig. S105 HRMS (APCI) of BCP **38**.

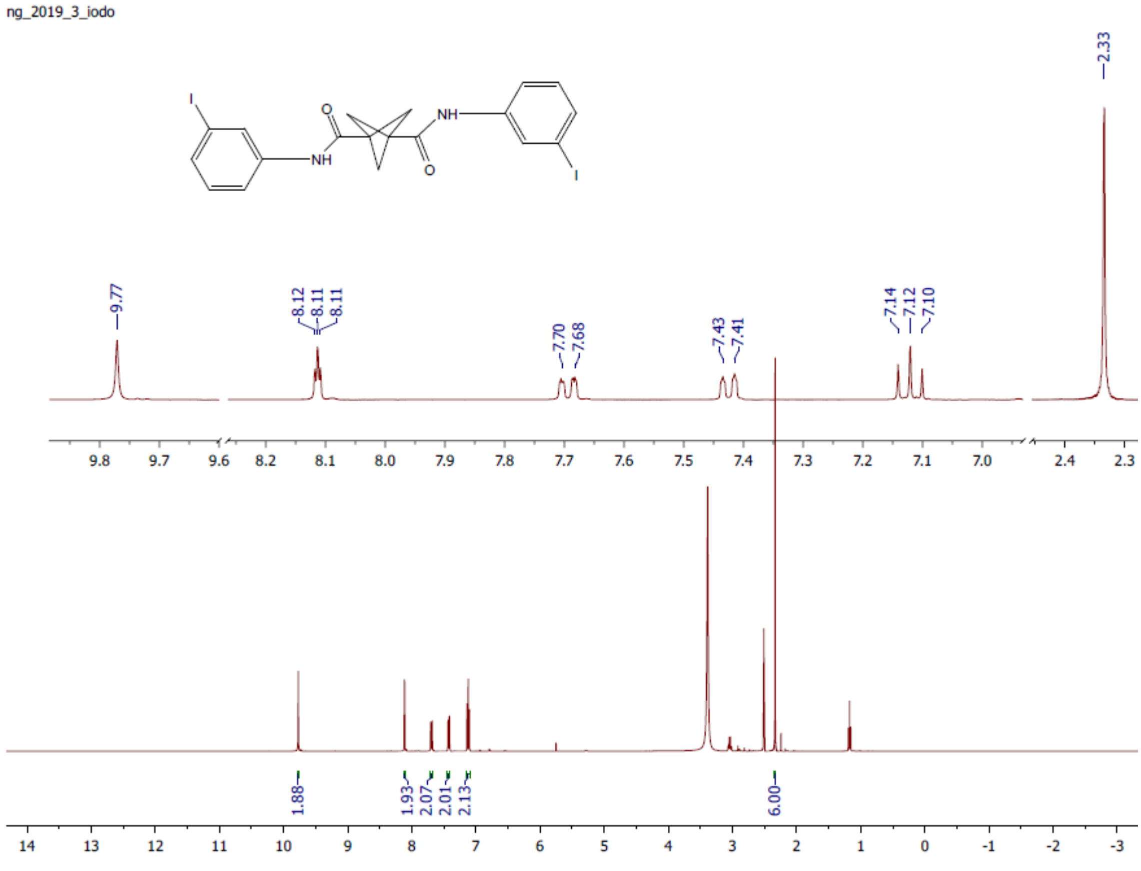

Fi g. S106 ^{1}H NMR spectrum of BCP **39** in DMSO-d_6.

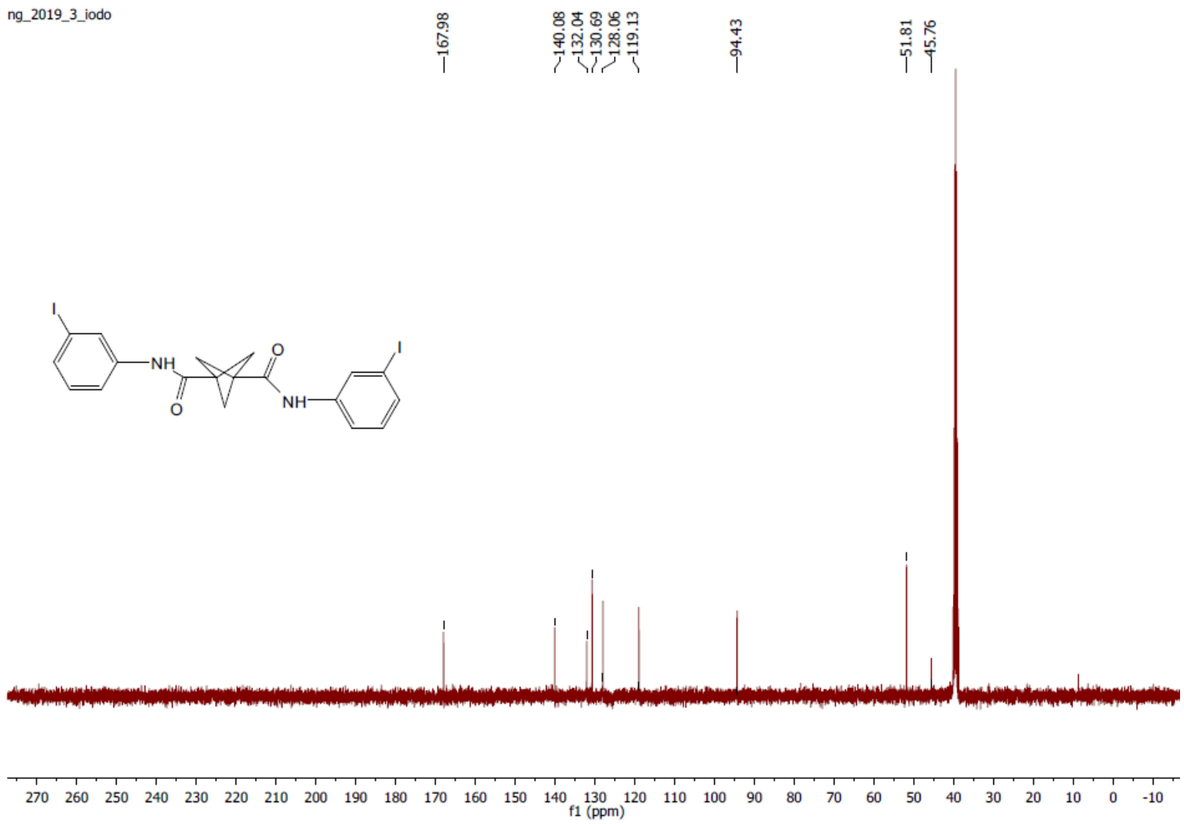

Fig. S107 ^{13}C NMR spectrum of BCP **39** in DMSO-d_6.

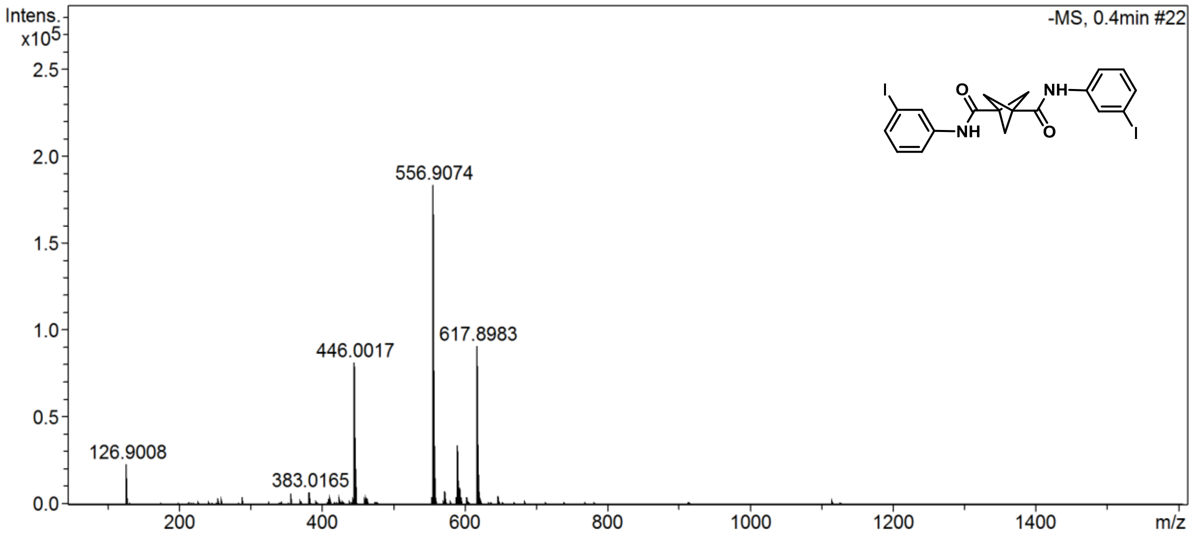

Fig. S108 HRMS (APCI) of BCP **39**.

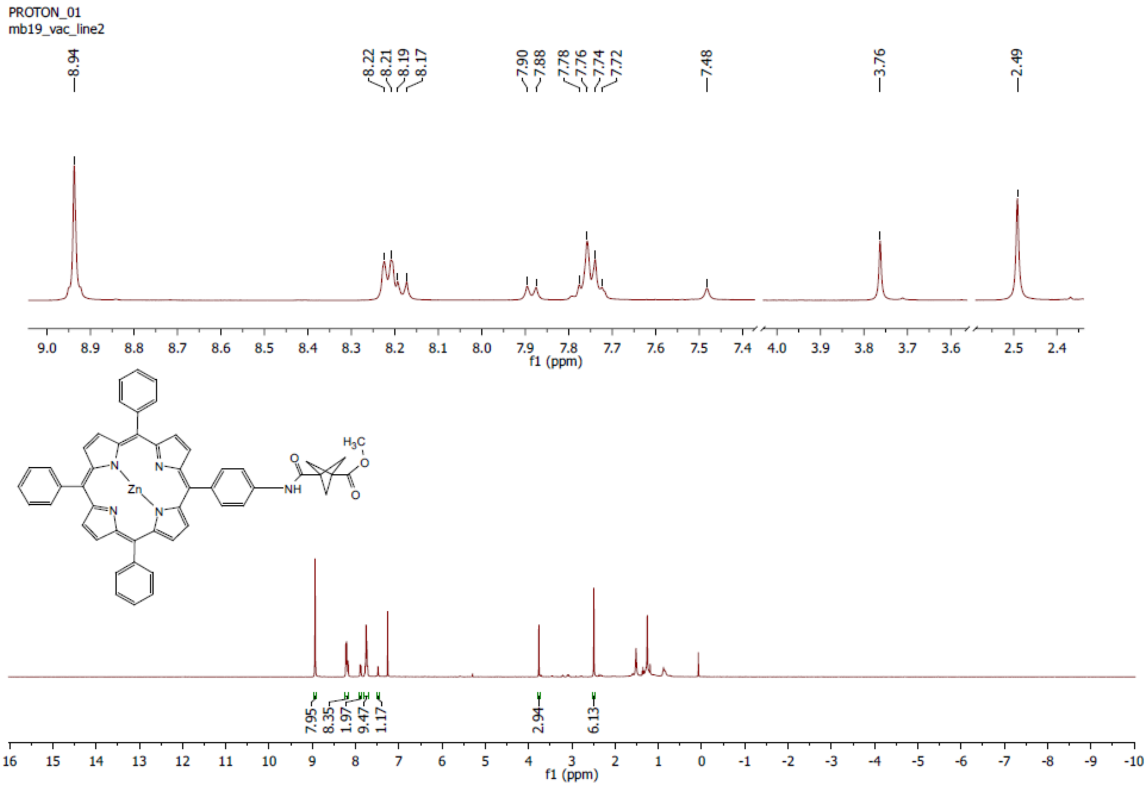

Fig. S109 ^{1}H NMR spectrum of BCP porphyrin monomer **44** in $CDCl_3$.

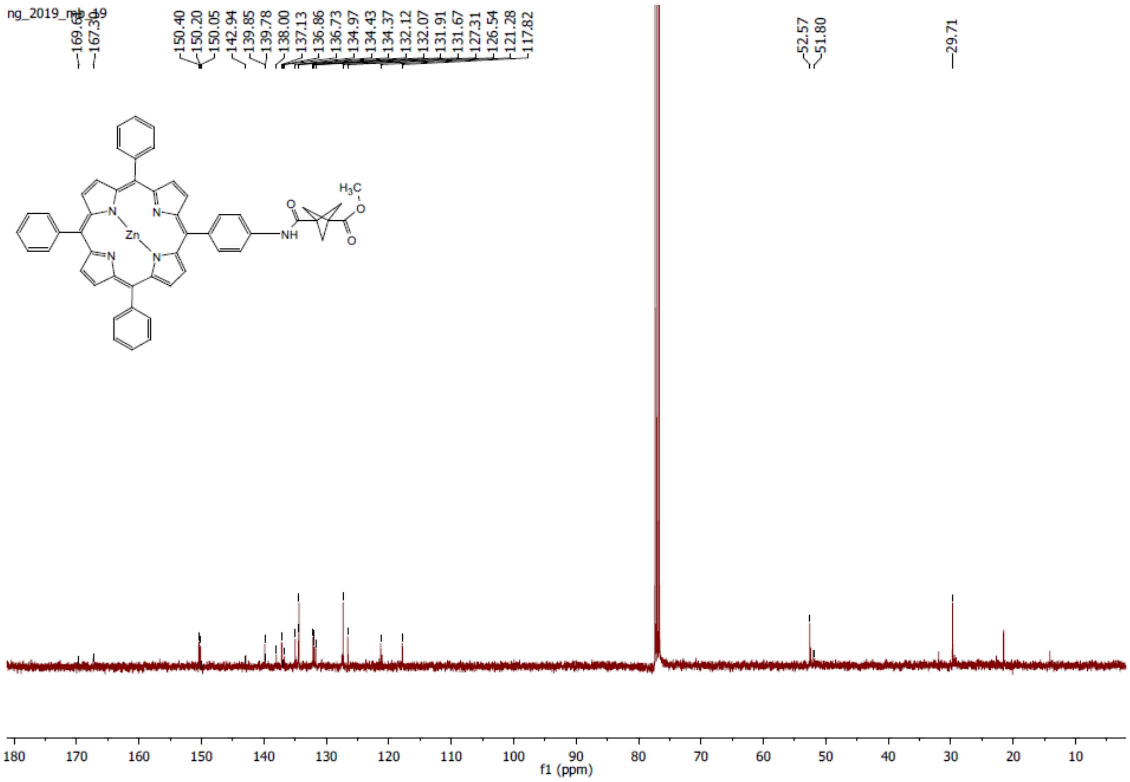

Fig. S110 ^{13}C NMR spectrum of BCP porphyrin monomer **44** in $CDCl_3$.

Single Mass Analysis

Tolerance = 10.0 PPM / DBE: min = -1.5, max = 200.0
Element prediction: Off
Number of isotope peaks used for i-FIT = 5

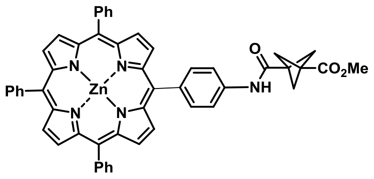

Monoisotopic Mass, Odd and Even Electron Ions
23 formula(e) evaluated with 1 results within limits (up to 10 best isotopic matches for each mass)
Elements Used:
C: 0-56 H: 0-41 N: 0-5 O: 0-3 Zn: 1-1
Michael Beh (MSe), MB19
Q-TOF20180606MF008 52 (1.146) AM (Cen,6, 80.00, Ht,10000.0,1570.68,0.70); Sm (SG, 2x3.00); Sb (15,10.00); Cm (12:81-51:69) TOF MS LD+ 6.49e+002

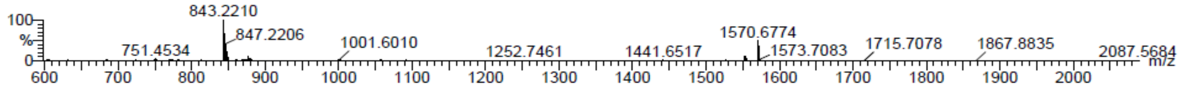

Minimum:				-1.5			
Maximum:		5.0	10.0	200.0			
Mass	Calc. Mass	mDa	PPM	DBE	i-FIT	i-FIT (Norm)	Formula
843.2210	843.2188	2.2	2.6	37.0	54.6	0.0	C52 H37 N5 O3 Zn

Fig. S111 MALDI-TOF-MS of BCP porphyrin monomer **44**.

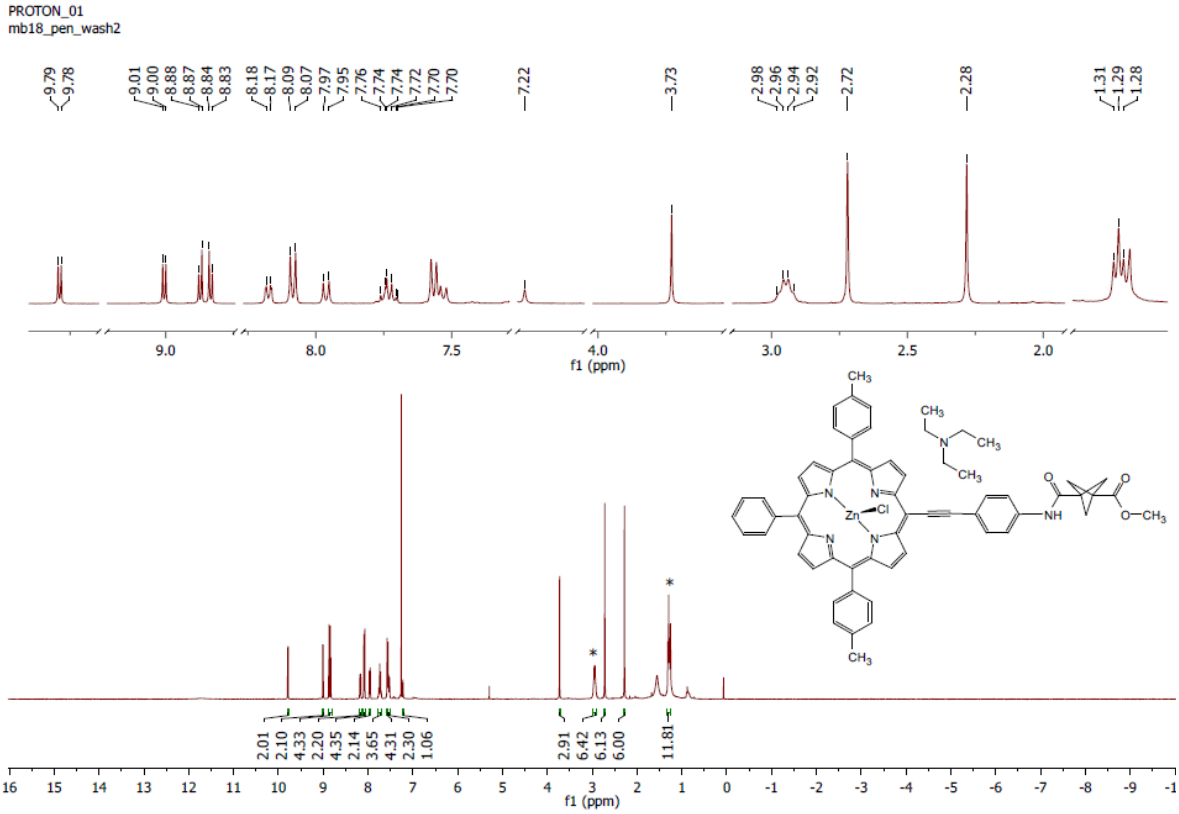

Fig. S112 ^{1}H NMR spectrum of BCP porphyrin monomer **45** in $CDCl_3$. (Asterisks show peaks corresponding to Et_3NI).

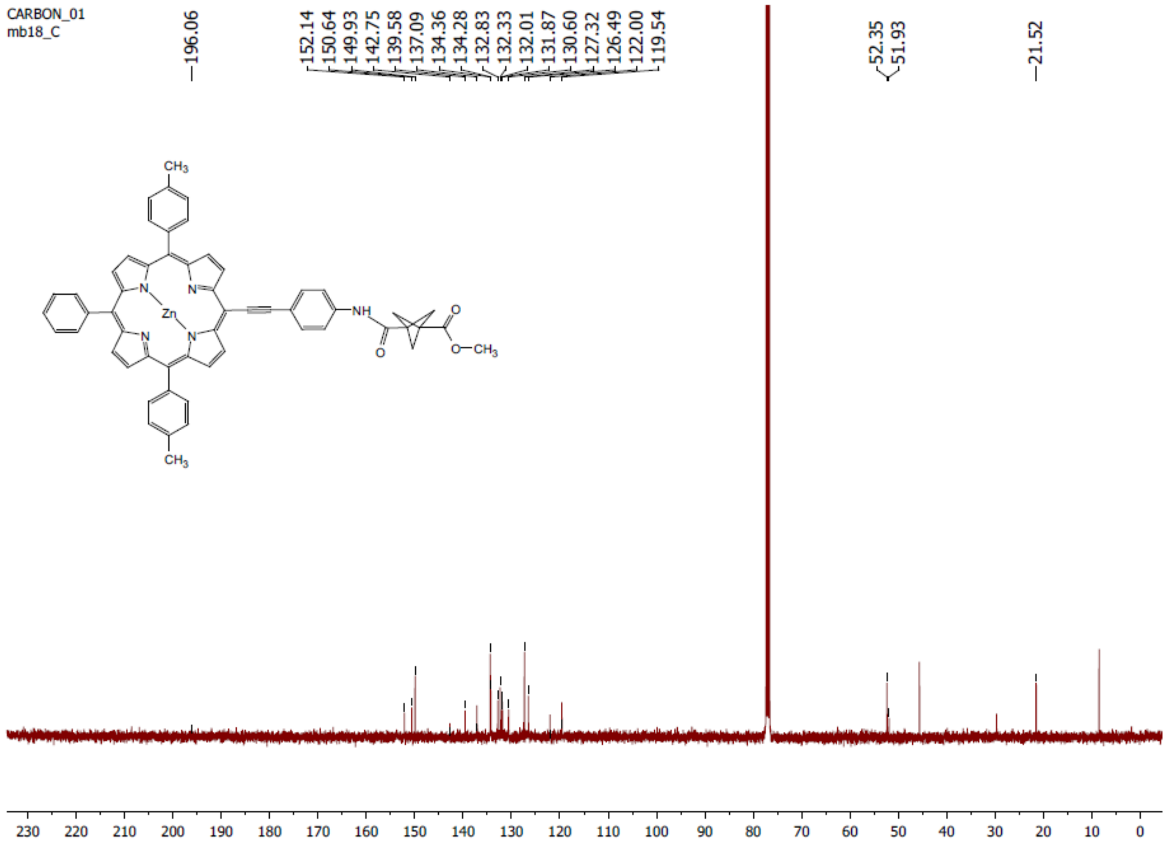

Fig. S113 ^{13}C NMR spectrum of BCP porphyrin monomer **45** in $CDCl_3$.

Single Mass Analysis
Tolerance = 10.0 PPM / DBE: min = -1.5, max = 200.0
Element prediction: Off
Number of isotope peaks used for i-FIT = 5

Monoisotopic Mass, Odd and Even Electron Ions
7 formula(e) evaluated with 1 results within limits (up to 10 best isotopic matches for each mass)
Elements Used:
C: 0-56 H: 0-41 N: 0-5 O: 0-3 Zn: 1-1

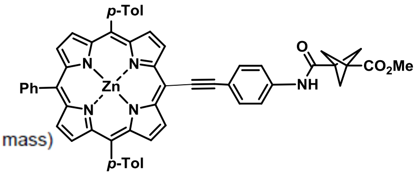

Michael Beh (MSe), MB18
Q-TOF20180606MF007 26 (0.482) AM (Cen,6, 80.00, Ht,10000.0,1570.68,0.70); Sm (SG, 2x3.00); Sb (15,10.00); Cm (7:79)
TOF MS LD+
9.87e+002

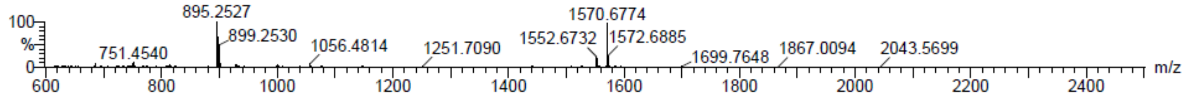

Minimum:				-1.5			
Maximum:		5.0	10.0	200.0			
Mass	Calc. Mass	mDa	PPM	DBE	i-FIT	i-FIT (Norm)	Formula
895.2527	895.2501	2.6	2.9	39.0	69.6	0.0	C56 H41 N5 O3 Zn

Fig. S114 MALDI-TOF-MS of BCP porphyrin monomer **45**.

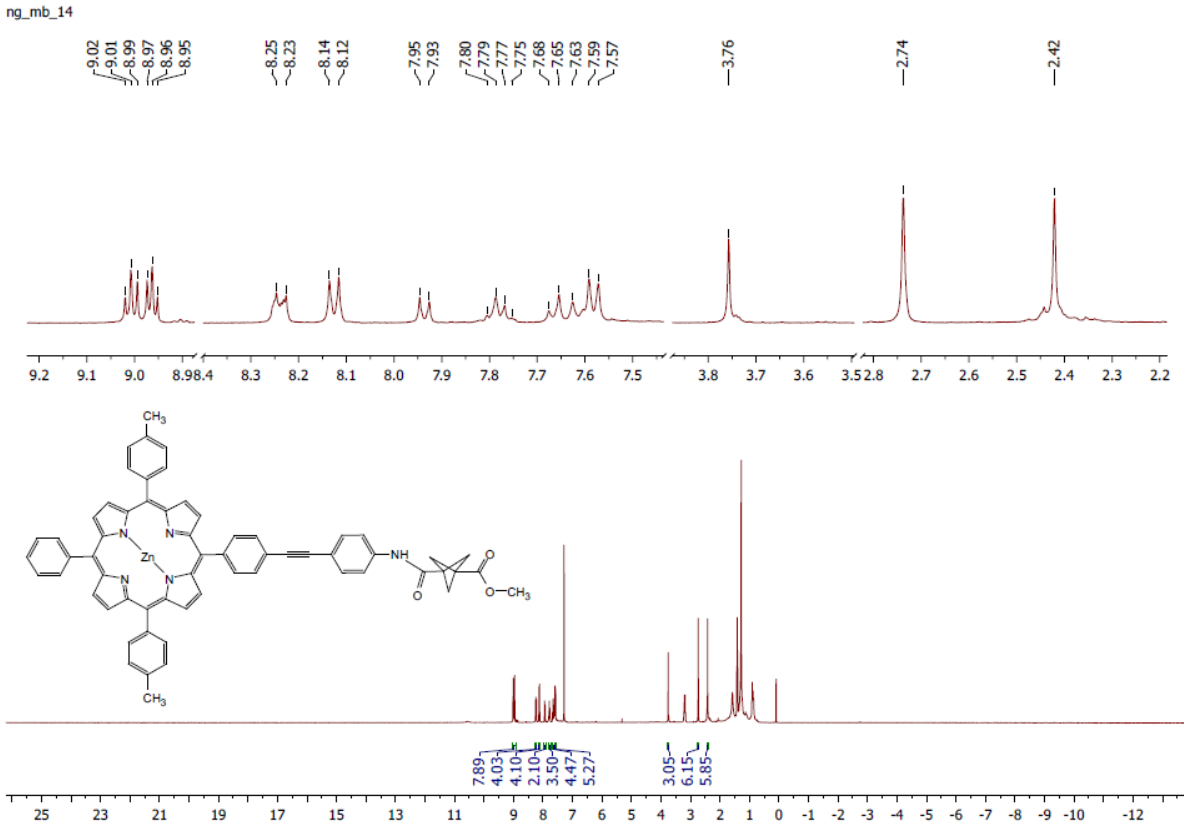

Fig. S115 ^{1}H NMR spectrum of BCP porphyrin monomer **46** in $CDCl_3$.

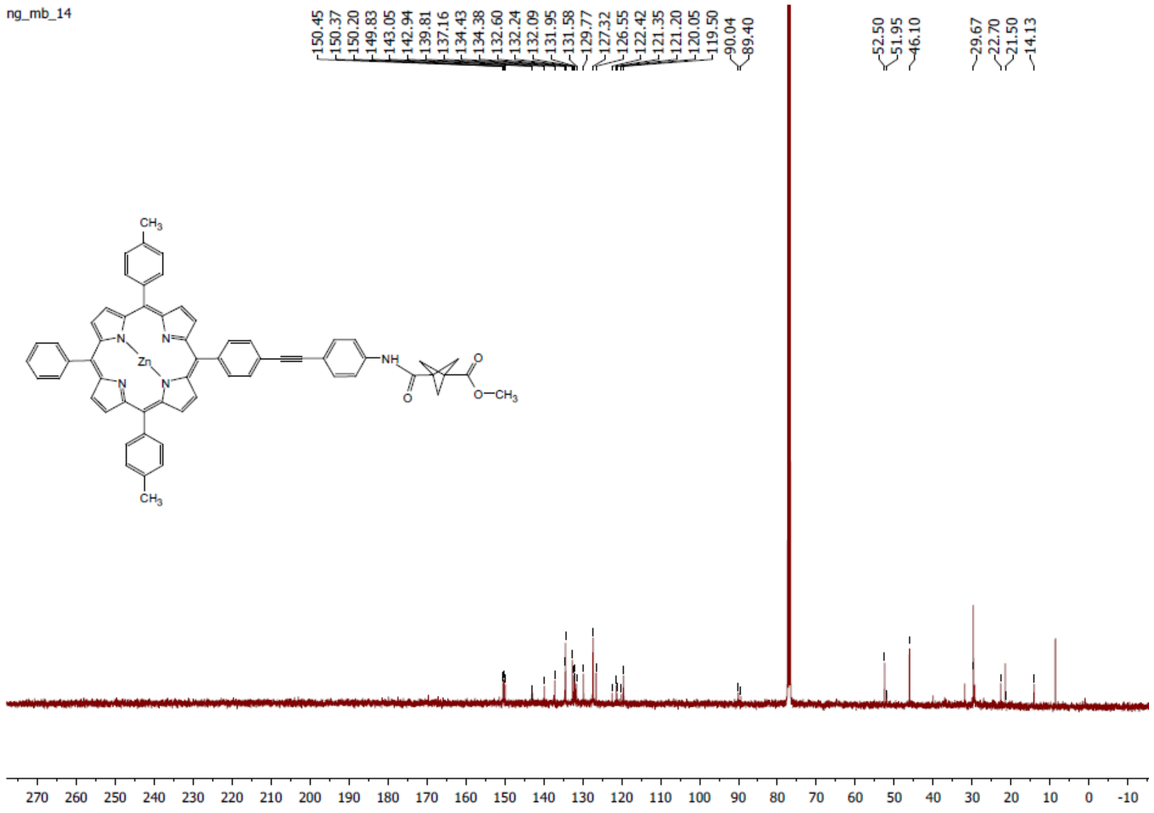

Fig. S116 ^{13}C NMR spectrum of BCP porphyrin monomer **46** in $CDCl_3$.

Single Mass Analysis
Tolerance = 10.0 PPM / DBE: min = -1.5, max = 200.0
Element prediction: Off
Number of isotope peaks used for i-FIT = 5

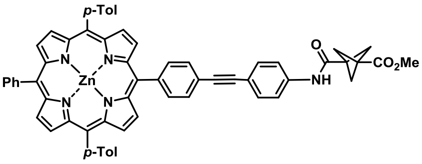

Monoisotopic Mass, Odd and Even Electron Ions
4 formula(e) evaluated with 1 results within limits (up to 10 best isotopic matches for each mass)
Elements Used:
C: 0-62 H: 0-45 N: 0-5 O: 0-3 Zn: 1-1

Michael Beh (MSe), MB14
Q-TOF20180606MF006 48 (1.084) AM (Cen,6, 80.00, Ht,10000.0,1570.68,0.70); Sm (SG, 2x3.00); Sb (15,10.00); Cm (6:81-47:59) TOF MS LD+ 8.00e+002

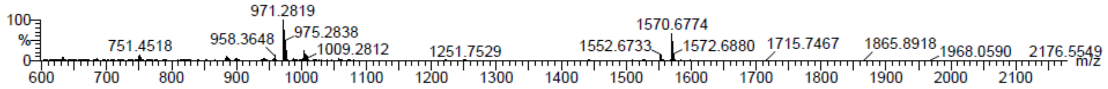

Minimum:				-1.5			
Maximum:		5.0	10.0	200.0			
Mass	Calc. Mass	mDa	PPM	DBE	i-FIT	i-FIT (Norm)	Formula
971.2819	971.2814	0.5	0.5	43.0	74.7	0.0	C62 H45 N5 O3 Zn

Fig. S117 MALDI-TOF-MS of BCP porphyrin monomer **46**.

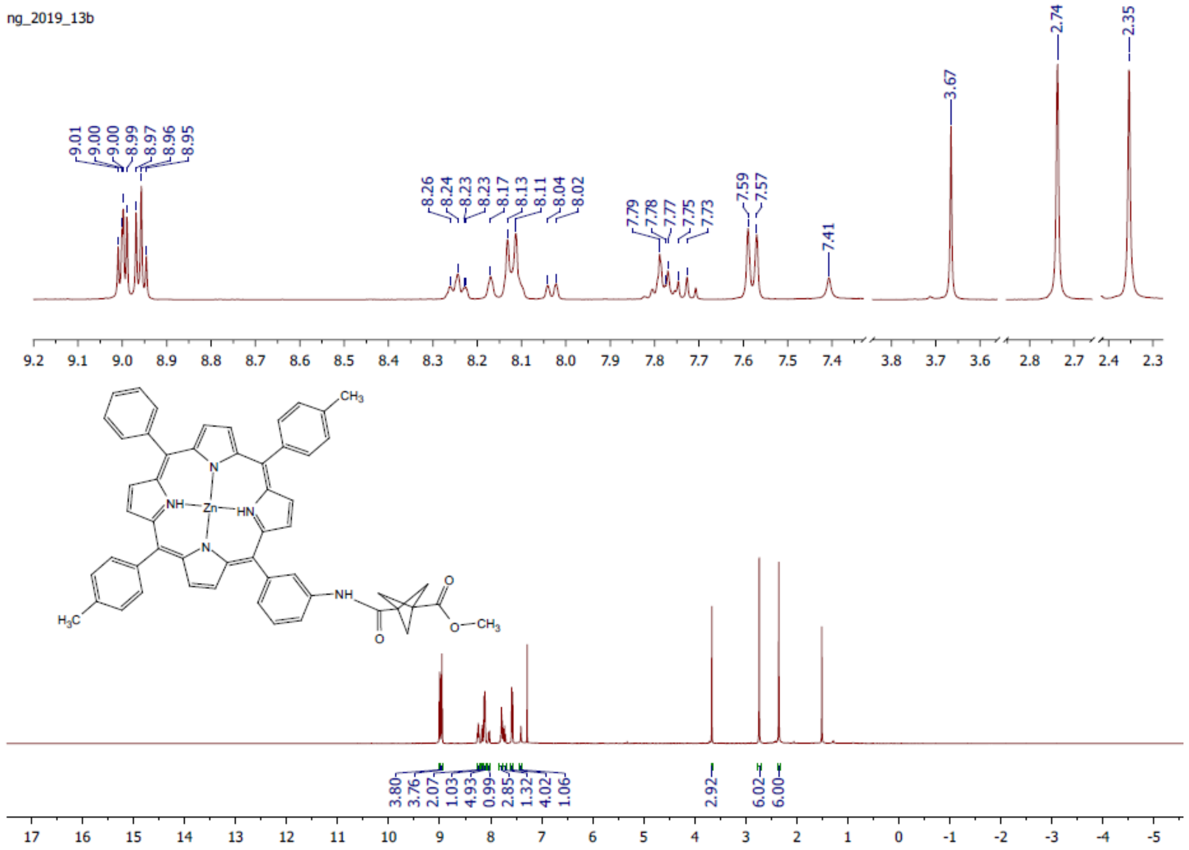

Fig. S118 ^{1}H NMR spectrum of BCP porphyrin monomer **47** in $CDCl_3$.

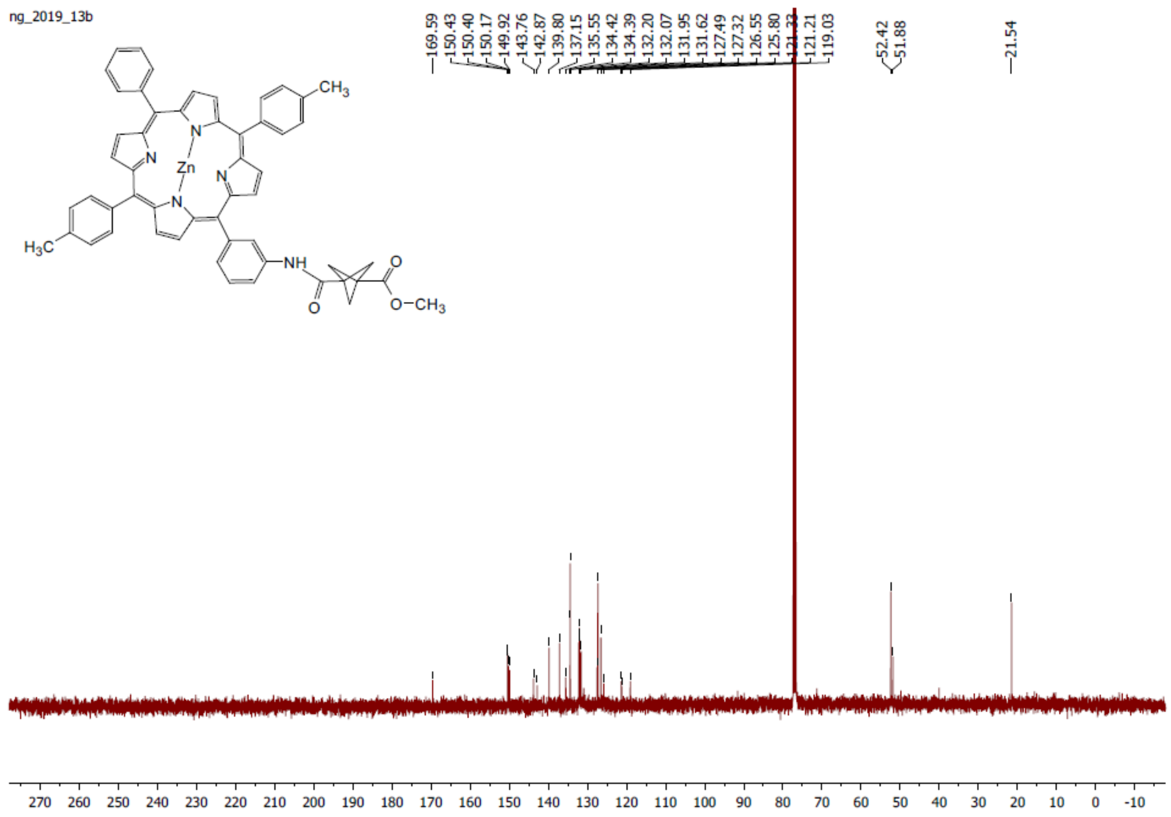

Fig. S119 ^{13}C NMR spectrum of BCP porphyrin monomer **47** in $CDCl_3$.

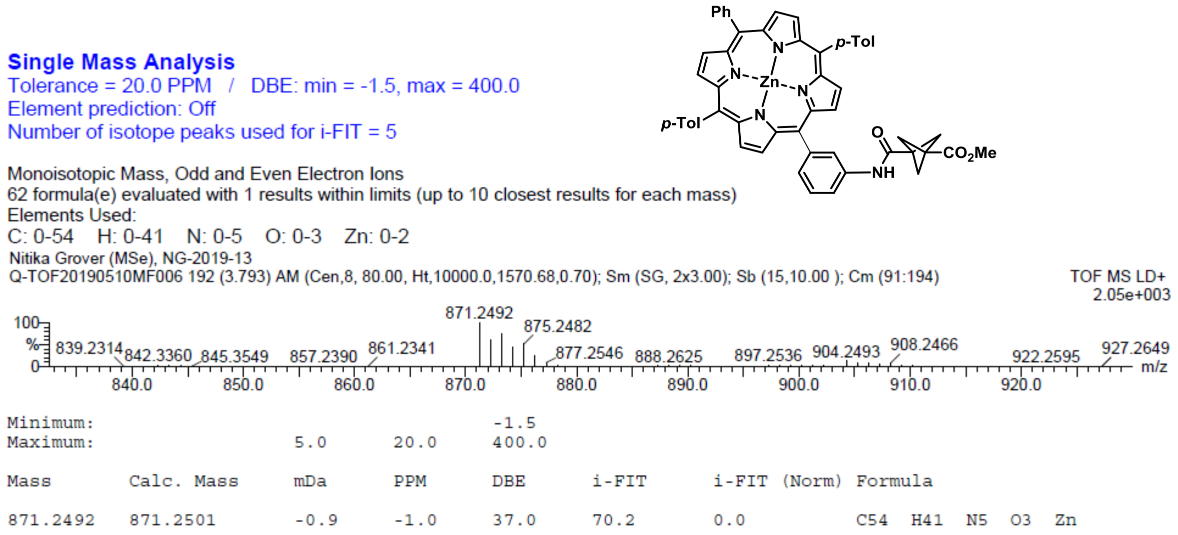

Fig. S120 MALDI-TOF-MS of BCP porphyrin monomer **47**.

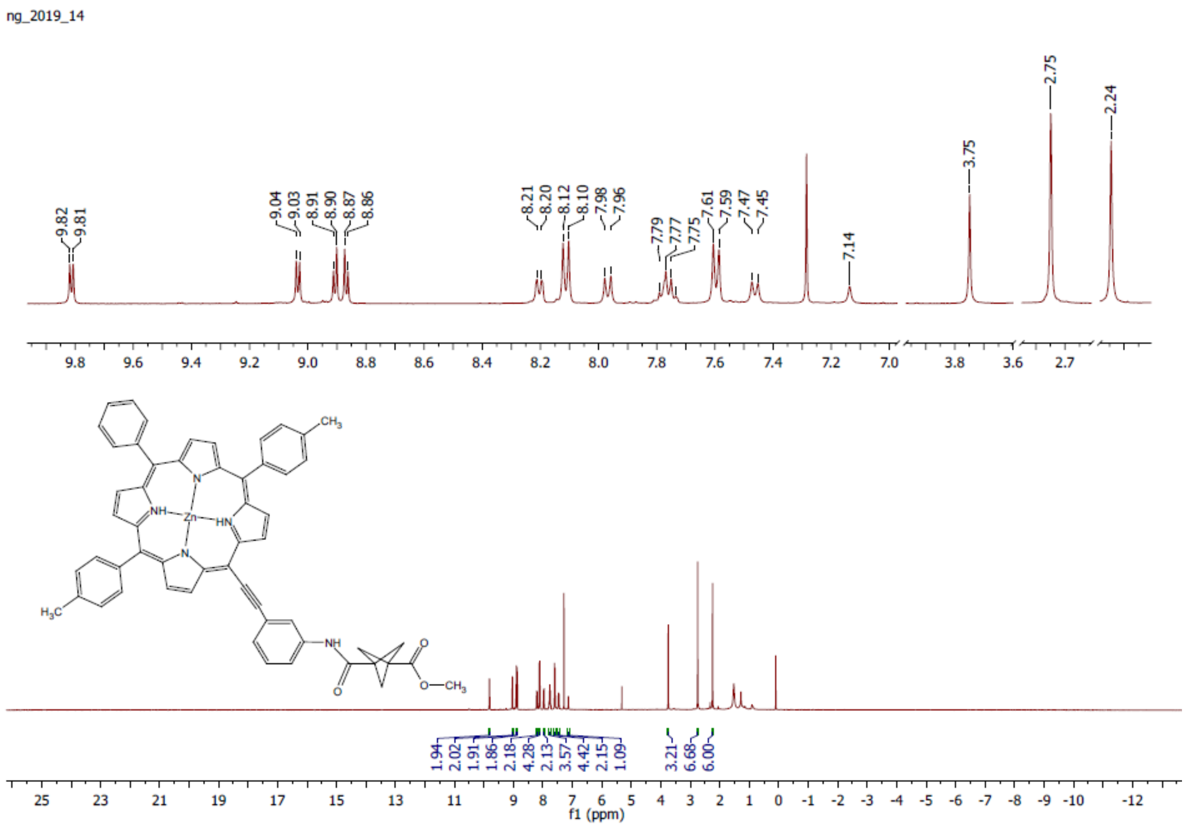

Fig. S121 ^{1}H NMR spectrum of BCP porphyrin monomer **48** in $CDCl_3$.

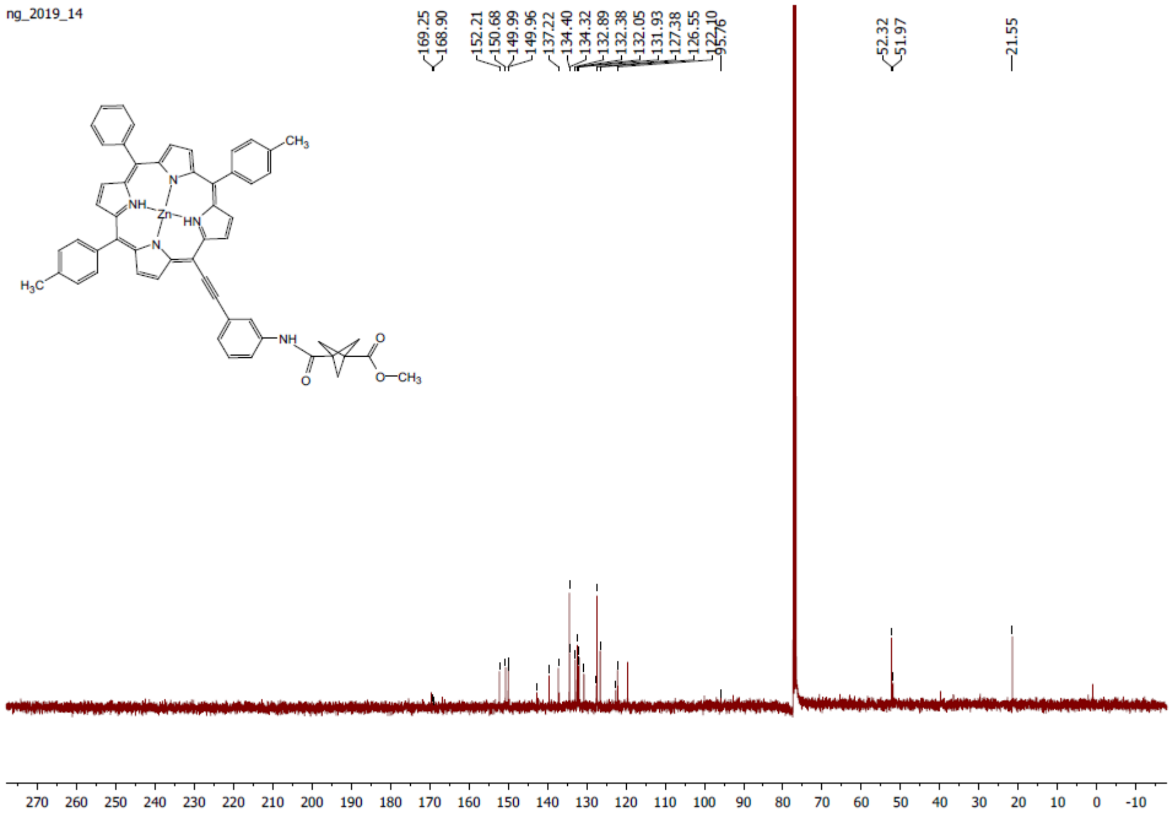

Fig. S122 ^{13}C NMR spectrum of BCP porphyrin monomer **48** in $CDCl_3$.

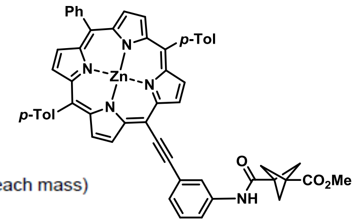

Single Mass Analysis
Tolerance = 20.0 PPM / DBE: min = -1.5, max = 400.0
Element prediction: Off
Number of isotope peaks used for i-FIT = 5

Monoisotopic Mass, Odd and Even Electron Ions
28 formula(e) evaluated with 1 results within limits (up to 10 closest results for each mass)
Elements Used:
C: 0-56 H: 0-41 N: 0-5 O: 0-3 Zn: 0-1
Nitika Grover (MSe), NG-2019-14
Q-TOF20190510MF004 35 (0.648) AM (Cen,8, 80.00, Ht,10000.0,1570.68,0.70); Sm (SG, 2x3.00); Sb (15,10.00); Cm (16:66) TOF MS LD+ 3.30e+003

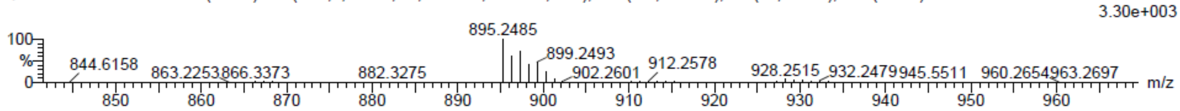

Minimum:				-1.5			
Maximum:		5.0	20.0	400.0			
Mass	Calc. Mass	mDa	PPM	DBE	i-FIT	i-FIT (Norm)	Formula
895.2485	895.2501	-1.6	-1.8	39.0	85.2	0.0	C56 H41 N5 O3 Zn

Fig. S123 MALDI-TOF-MS of BCP porphyrin monomer **48**.

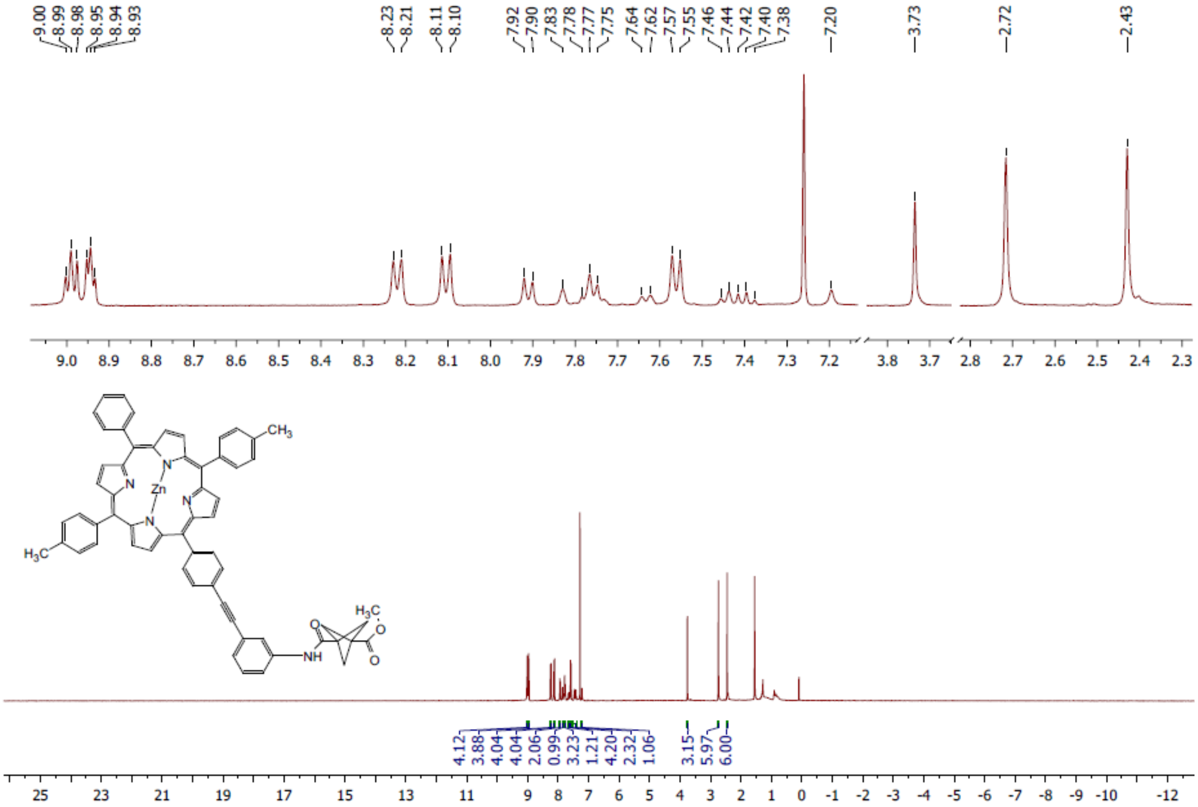

Fig. S124 ^{1}H NMR spectrum of BCP porphyrin monomer **49** in $CDCl_3$.

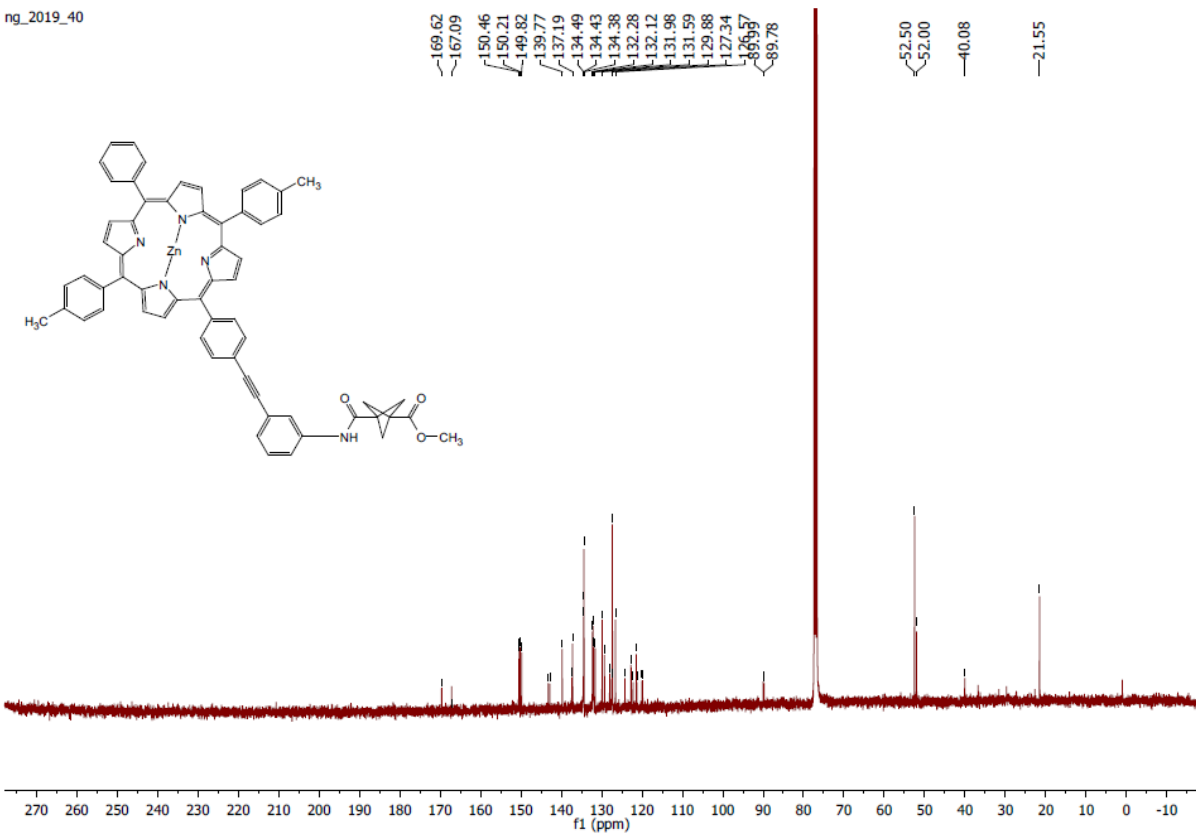

Fig. S125 ^{13}C NMR spectrum of BCP porphyrin monomer **49** in $CDCl_3$.

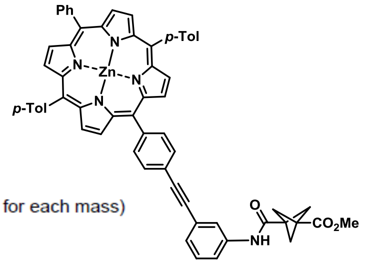

Single Mass Analysis
Tolerance = 20.0 PPM / DBE: min = -1.5, max = 400.0
Element prediction: Off
Number of isotope peaks used for i-FIT = 5

Monoisotopic Mass, Odd and Even Electron Ions
22 formula(e) evaluated with 1 results within limits (up to 10 closest results for each mass)
Elements Used:
C: 0-62 H: 0-45 N: 0-5 O: 0-3 Zn: 0-1
Nitika Grover (Mse), NG-2019-40
Q-TOF20190607MF008 35 (0.648) AM (Cen,8, 80.00, Ht,10000.0,1570.68,0.70); Sm (SG, 2x3.00); Sb (15,10.00); Cm (24:53)

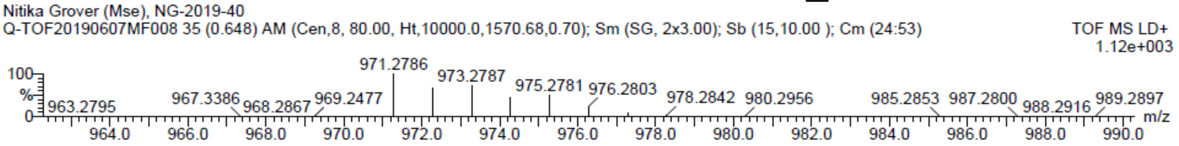

Minimum:				-1.5			
Maximum:		5.0	20.0	400.0			
Mass	Calc. Mass	mDa	PPM	DBE	i-FIT	i-FIT (Norm)	Formula
971.2786	971.2814	-2.8	-2.9	43.0	63.5	0.0	C62 H45 N5 O3 Zn

Fig. S126 MALDI-TOF-MS of BCP porphyrin monomer **49**.

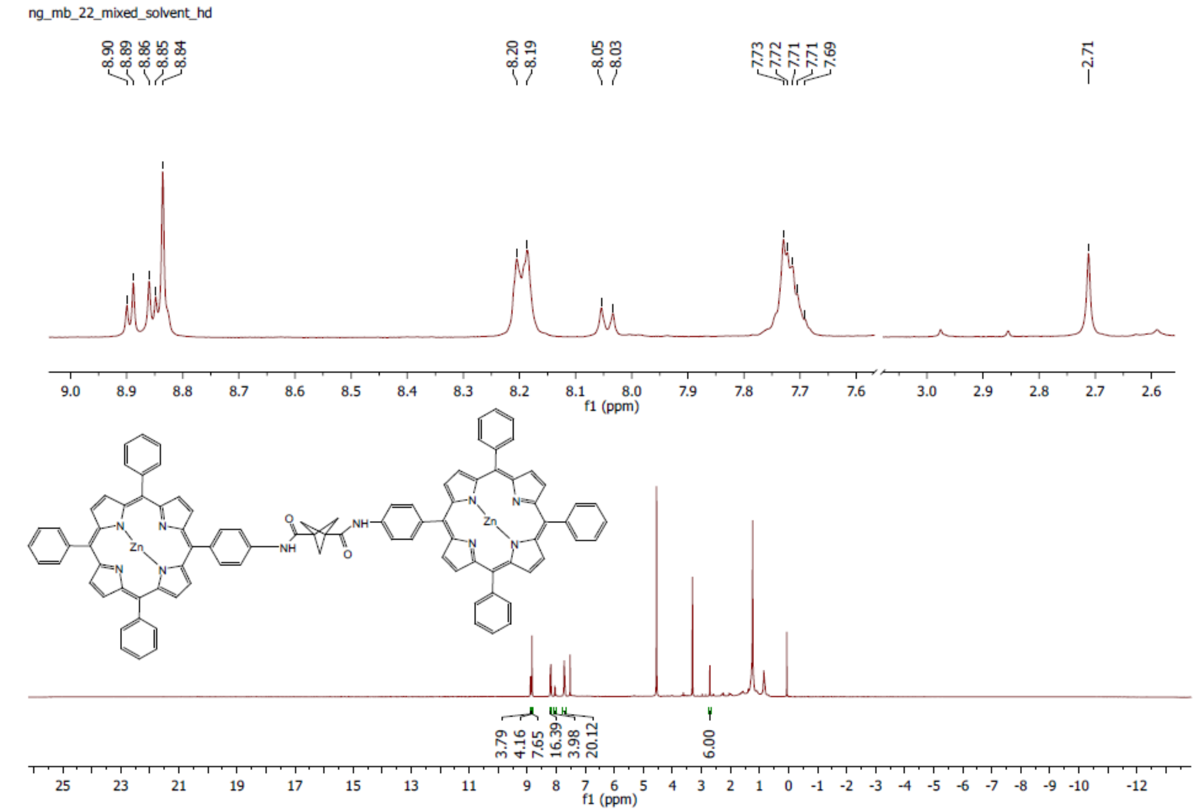

Fig. S127 ^{1}H NMR spectrum of BCP porphyrin dimer **50** in $CDCl_3/CD_3OD$.

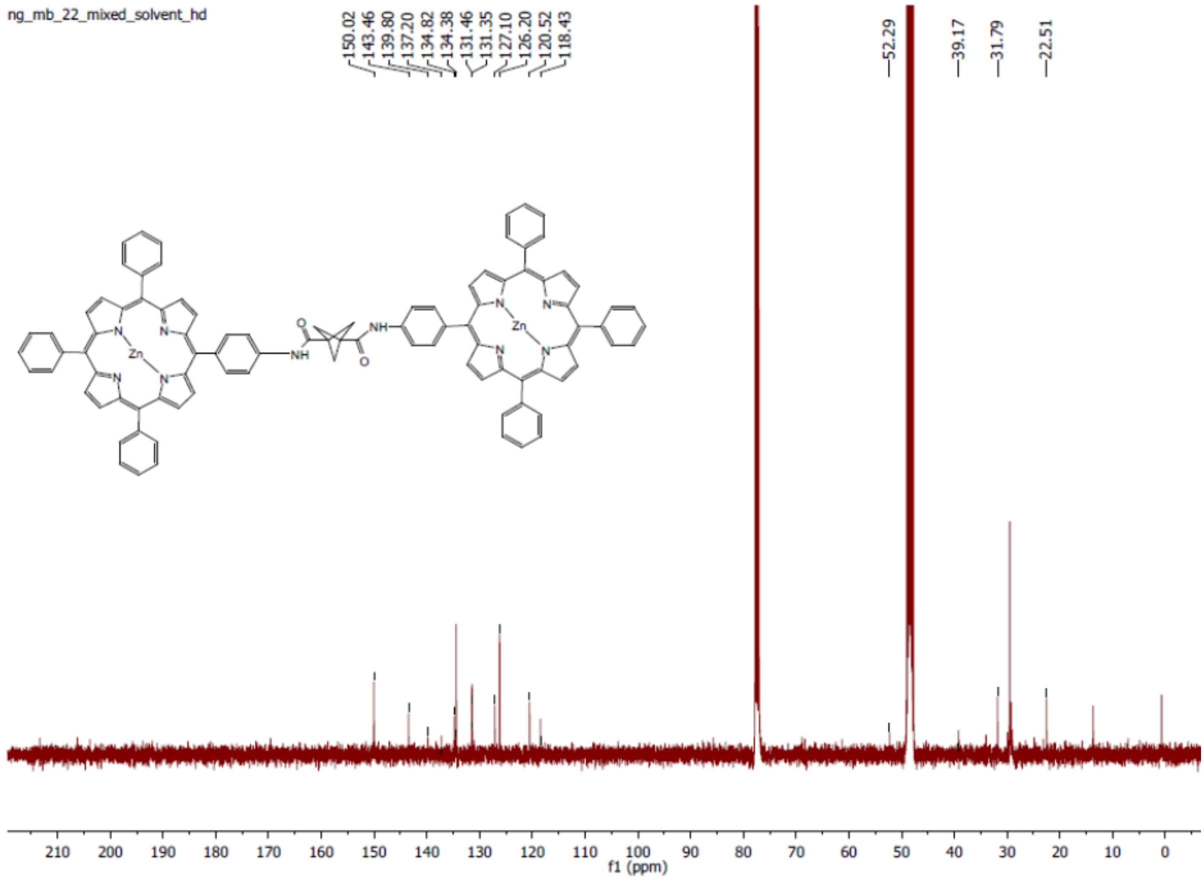

Fig. S128 ^{13}C NMR spectrum of BCP porphyrin dimer **50** in $CDCl_3/CD_3OD$.

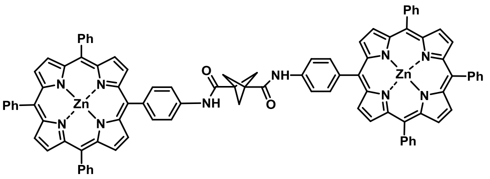

Single Mass Analysis
Tolerance = 10.0 PPM / DBE: min = -1.5, max = 200.0
Element prediction: Off
Number of isotope peaks used for i-FIT = 5

Monoisotopic Mass, Odd and Even Electron Ions
53 formula(e) evaluated with 1 results within limits (up to 10 best isotopic matches for each mass)
Elements Used:
C: 0-95 H: 0-62 N: 0-10 O: 0-2 Zn: 0-2

Michael Beh (MSe), MB21-2 mid
Q-TOF20180608MF003 49 (0.908) AM (Cen,6, 80.00, Ht,10000.0,1570.68,0.70); Sm (SG, 2x3.00); Sb (15,10.00); Cm (9:145-(83:97+115)) TOF MS LD+ 2.41e+003

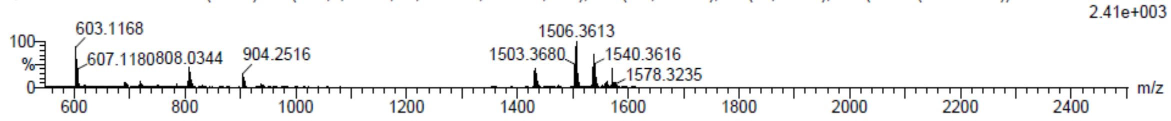

Minimum:				-1.5			
Maximum:		5.0	10.0	200.0			
Mass	Calc. Mass	mDa	PPM	DBE	i-FIT	i-FIT (Norm)	Formula
1502.3638	1502.3640	-0.2	-0.1	70.0	111.0	0.0	C95 H62 N10 O2 Zn2

Fig. S129 MALDI-TOF-MS of BCP porphyrin dimer **50**.

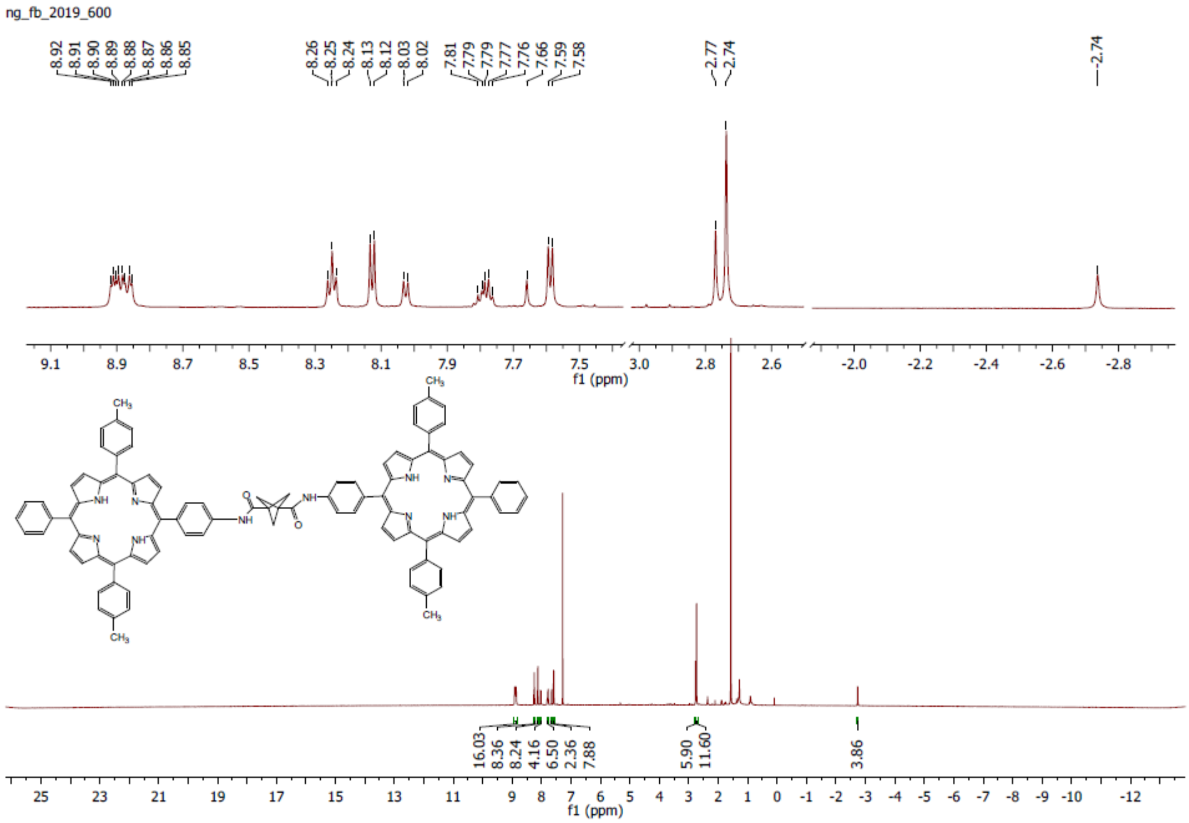

Fig. S130 ^{1}H NMR spectrum of BCP porphyrin dimer **51** in $CDCl_3$.

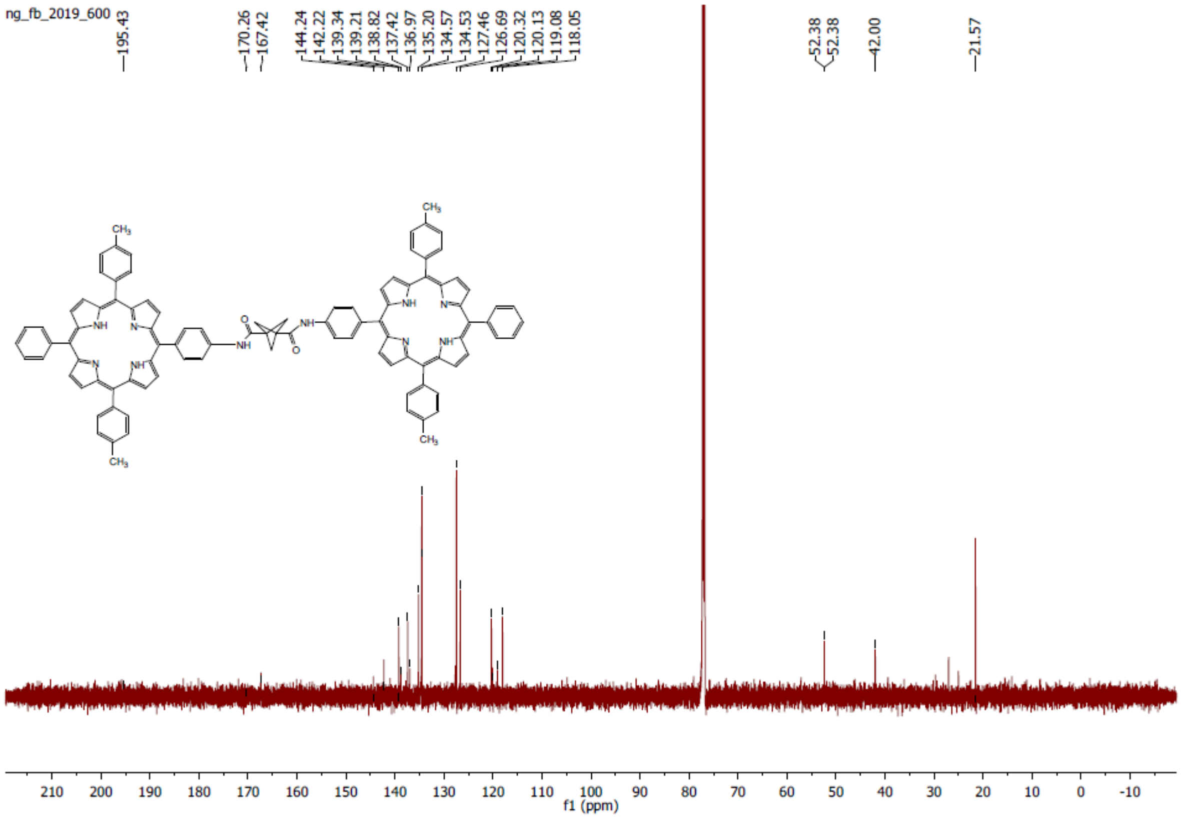

Fig. S131 ^{1}H NMR spectrum of BCP porphyrin dimer **51** in $CDCl_3$.

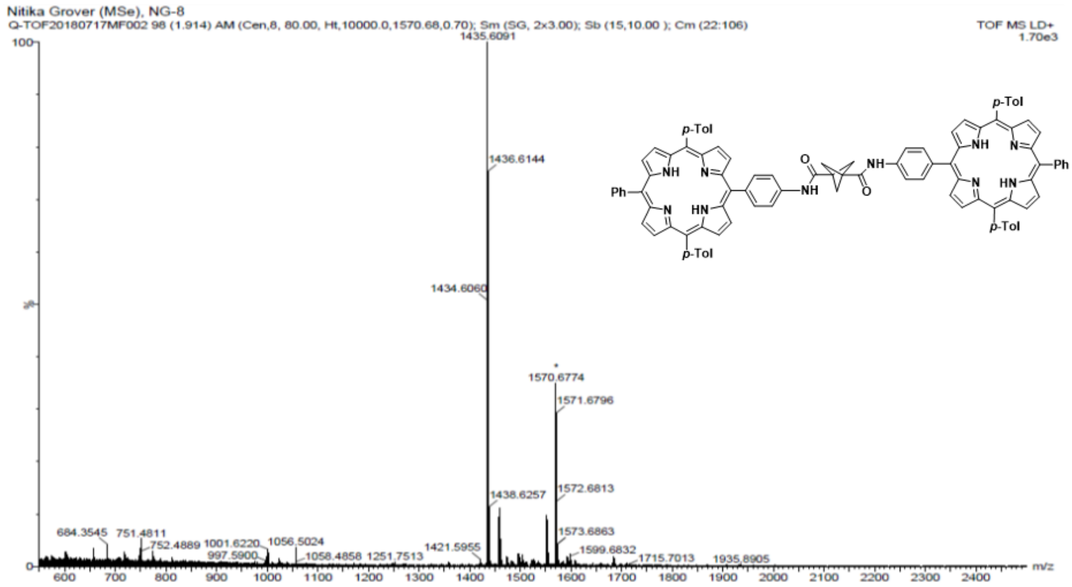

Fig. S132 MALDI-TOF-MS of BCP porphyrin dimer **51**.

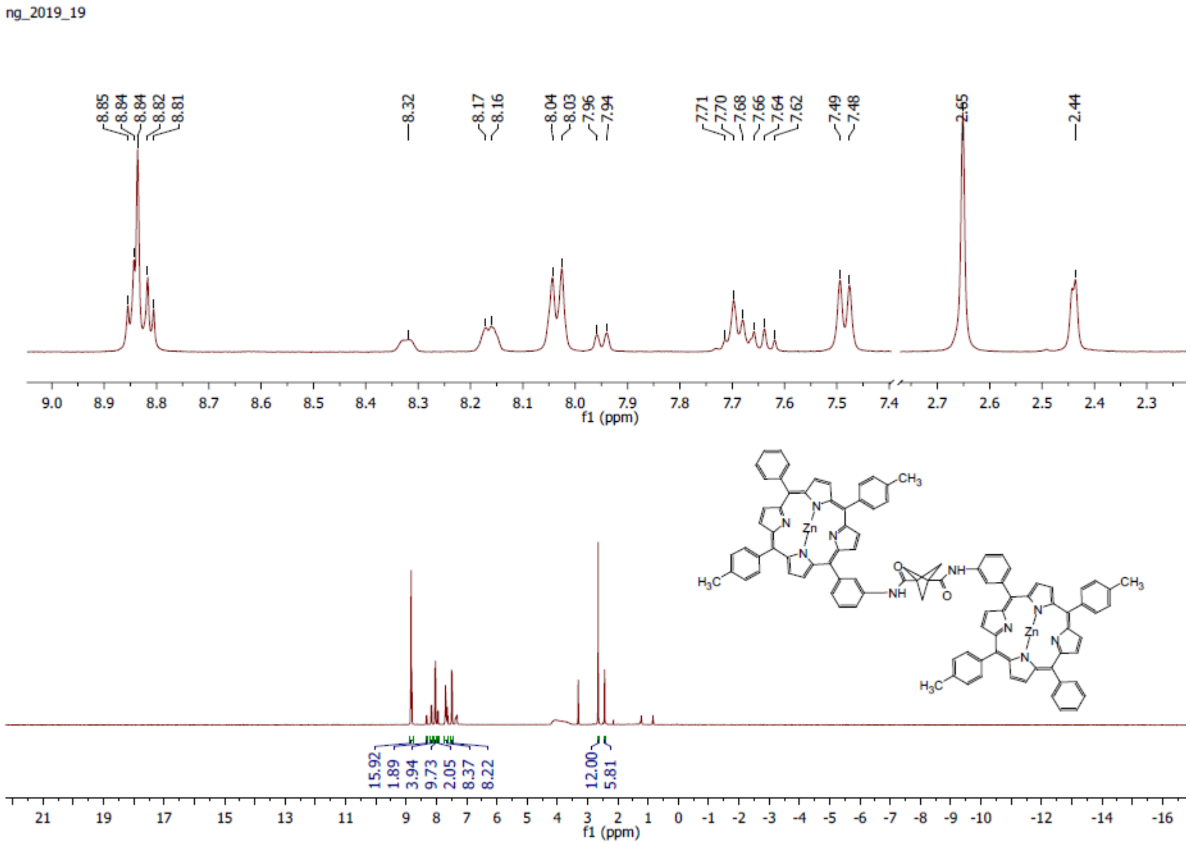

Fig. S133 ^{1}H NMR spectrum of BCP porphyrin dimer **52** in $CDCl_3/CD_3OD$.

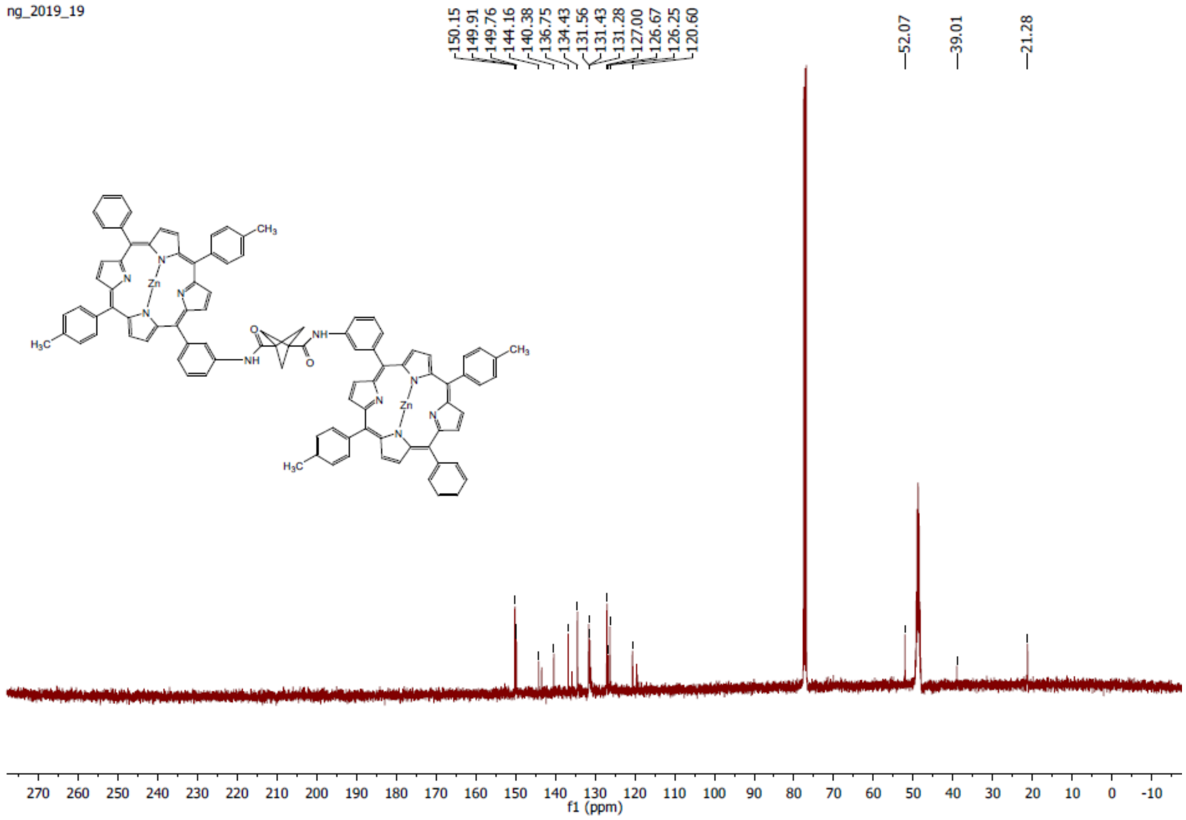

Fig. S134 ^{13}C NMR spectrum of BCP porphyrin dimer **52** in $CDCl_3/CD_3OD$.

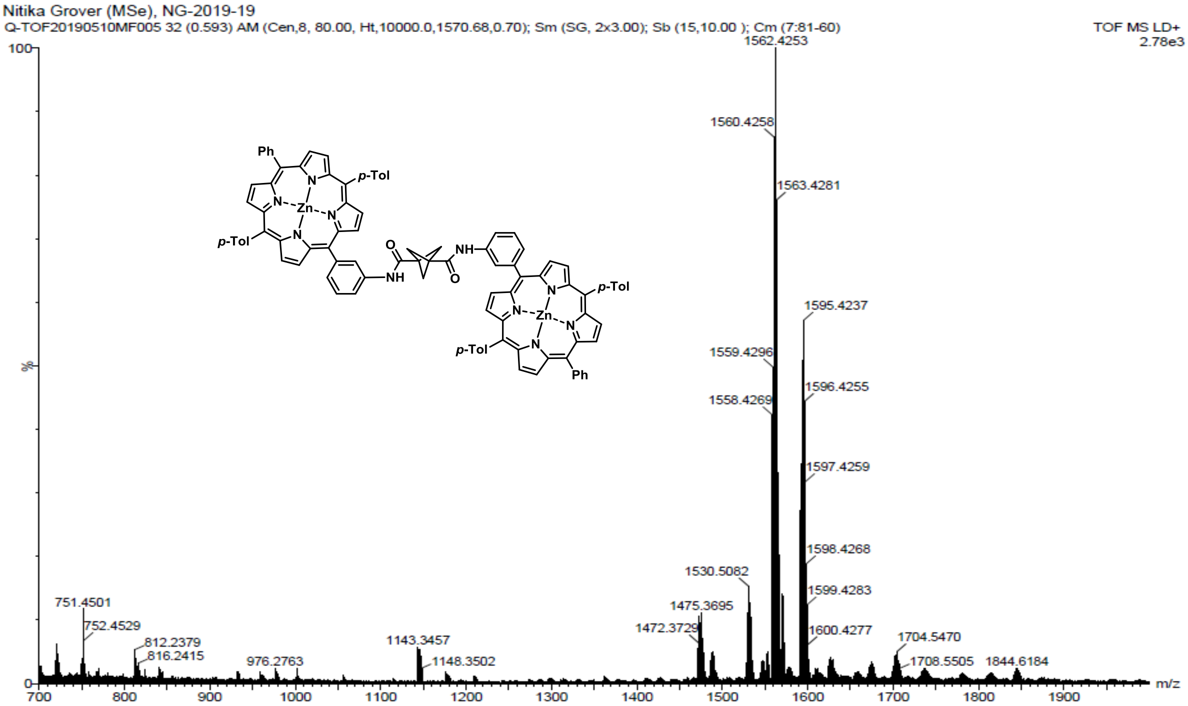

Fig. S135 MALDI-TOF-MS of BCP porphyrin dimer **52**.

3. Single Crystal X-ray Structures

Experimental details

The crystals of compound (**12, 13**, **33**, **35**, **38**, **45**, **46** and dimethyl bicyclo[1.1.1]pentane-1,3-dicarboxylate) were grown following the protocol developed by Hope by dissolving the compounds in either CH_2Cl_2 or $CDCl_3$ and layering with a second solvent (MeOH or hexane) for liquid diffusion over time.[12] The crystals of BCP diester and **23** were grown by slow evaporation in CH_2Cl_2 and DMSO. Single crystal X-ray diffraction data for all compounds were collected on a Bruker APEX 2 DUO CCD diffractometer by using graphite-monochromated MoK_α (λ = 0.71073 Å) radiation (**12, 33**, **35**, **38**, **28** and dimethyl bicyclo[1.1.1]pentane-1,3-dicarboxylate) and Incoatec IμS CuK_α (λ = 1.54178 Å) (**13** and **45**) radiation. Crystals were mounted on a MiTeGen MicroMount and collected at 100(2) K by using an Oxford Cryosystems Cobra low-temperature device. Data were collected by using omega and phi scans and were corrected for Lorentz and polarization effects by using the APEX software suite.[13] Using Olex2, the structure was solved with the XT structure solution program, using the intrinsic phasing solution method and refined against |F2| with XL using least squares minimization.[14] Hydrogen atoms were generally placed in geometrically calculated positions and refined using a riding model. Details of data refinements can be found in Table S1 and S2. All images were prepared by using Mercury3.7[15]and Olex2.[13a]

Refinement details of porphyrin 12

The distance of the N-H bond was fixed using HIMP. The tolyl group at C29 was modelled over two position using the restrain SIMU in a 58:42% occupancy. The solvent in the void was squeezed out using OLEX2 maps as no reliable solution could be obtained.

Refinement details of compound 13

The cubane moiety was fixed using restraint SIMU.

Refinement details of compound 33

The distance of the N-H bond was fixed using the restraint DFIX.

Refinement details of compound 35

The distance of the N-H bond was fixed using the restraint DFIX.

Refinement details of compound 38

The distance of the N-H bond was fixed using the restraint DFIX.

Refinement details of compound 45

The distance of the N-H bond was fixed using the restraint DFIX. The solvent in the void was squeezed out using OLEX2 maps as no reliable solution could be obtained.

Refinement details of compound 46

The methyl ester at C71 was modelled over two position using restrains (SADI, SIMU, DFIX, and ISOR) in a 55:45% occupancy. The phenyl group at C151 was treated with the restraint SIMU. The distance of the N-H bond was fixed using the restraint DFIX. The solvent in the void was squeezed out using OLEX2 maps as no reliable solution could be obtained.

Refinement details of BCP diester

The structure was modelled by importing a rigid molecule using FRAG/FEND and refined at 25% occupancy due to the four-fold disorder present in the structure. All non-hydrogen atoms had the SIMU restraint applied to them.

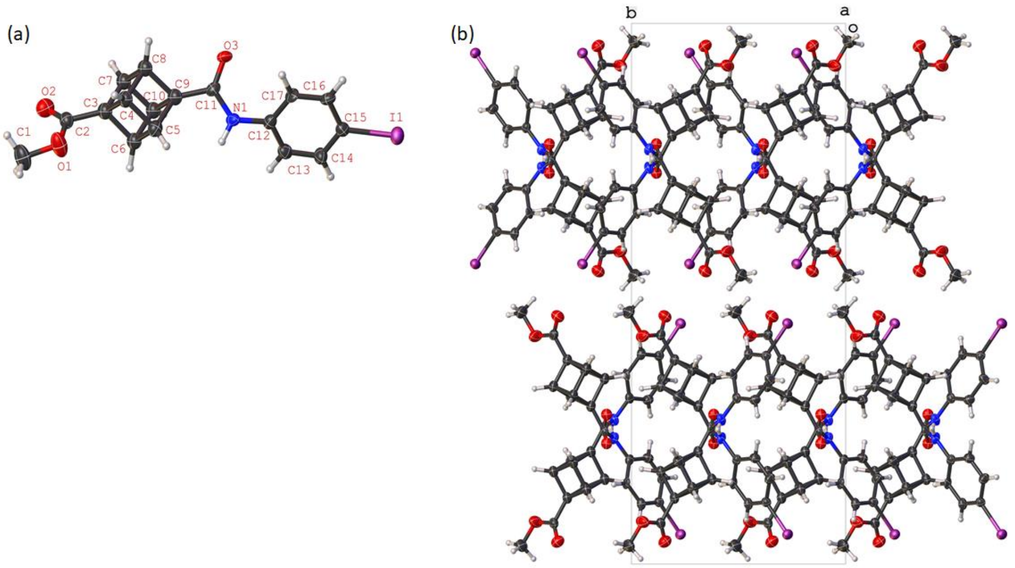

Fig. **S136** (a) Molecular structure of cubane **13** with all non-hydrogen atoms labelled (thermal displacement 50%). (b) Moiety packing shown with labels omitted.

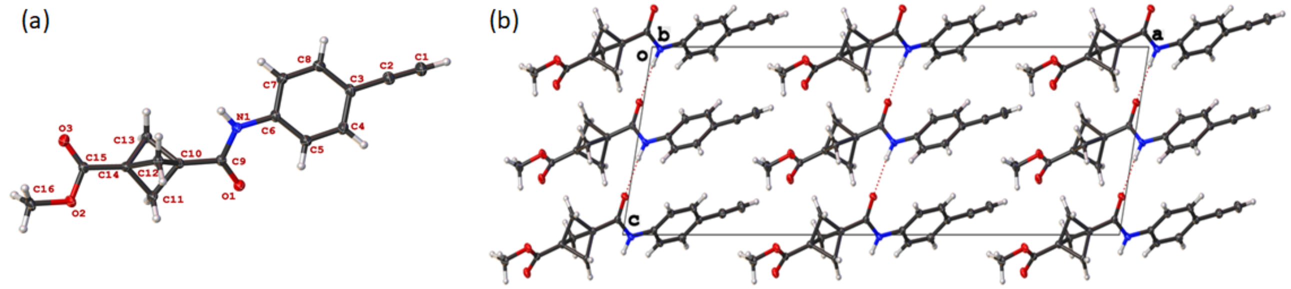

Fig. **S137** (a) Molecular structure of BCP **33** with all non-hydrogen atoms labelled (thermal displacement 50%). (b) Moiety packing shown with labels omitted.

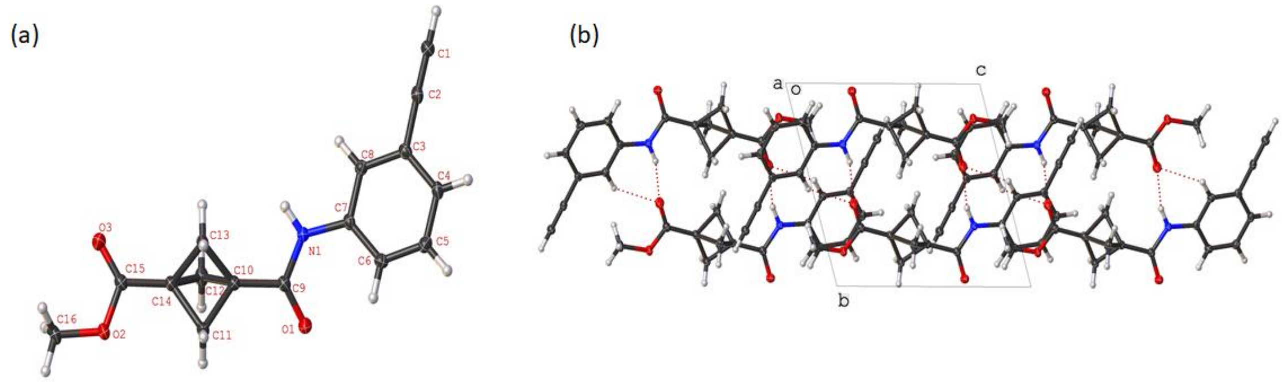

Fig. S138 (a) Molecular structure of BCP **35** with all non-hydrogen atoms labelled (thermal displacement 50%). (b) Moiety packing shown with labels omitted.

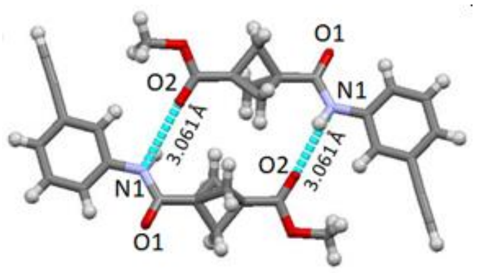

Fig. S139 Molecular arrangement of BCP **35** in the crystal showing the non-covalent interactions between N1···O2.

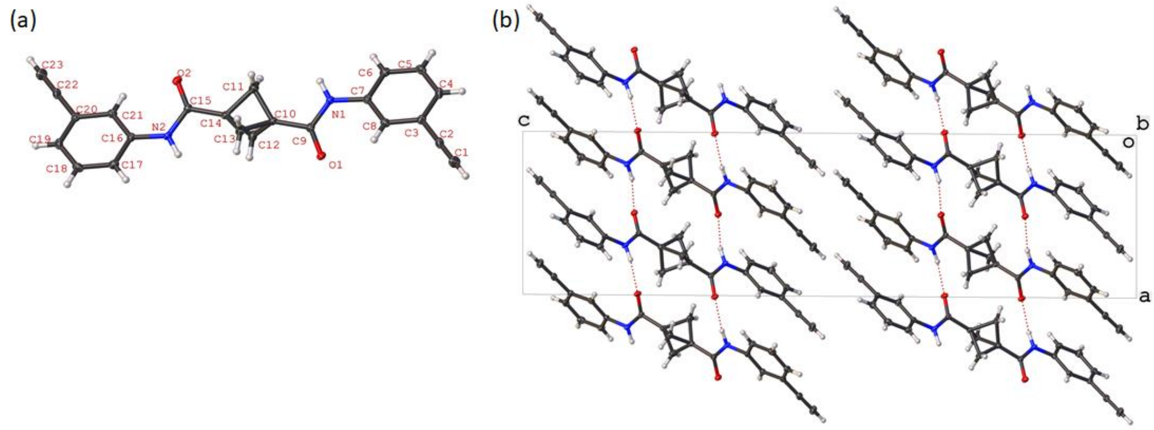

Fig. S140 (a) Molecular structure of BCP dimer **38** with all non-hydrogen atoms labelled (thermal displacement 50%). (b) Moiety packing shown with labels omitted.

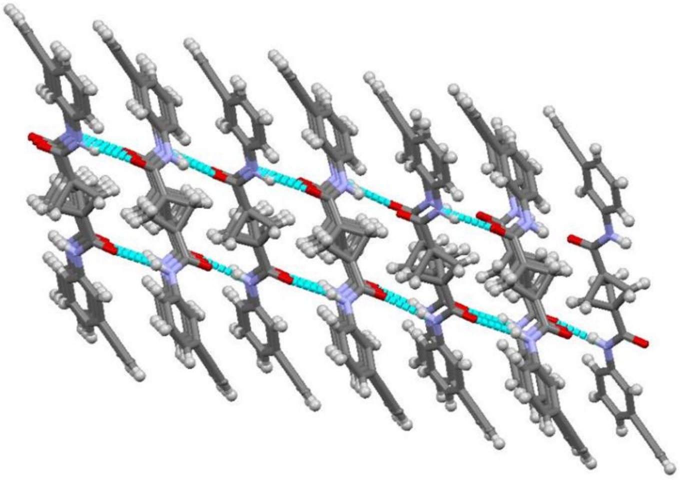

Fig. S141 Molecular arrangement of **38** in the crystal showing the non-covalent interactions between N1···O2.

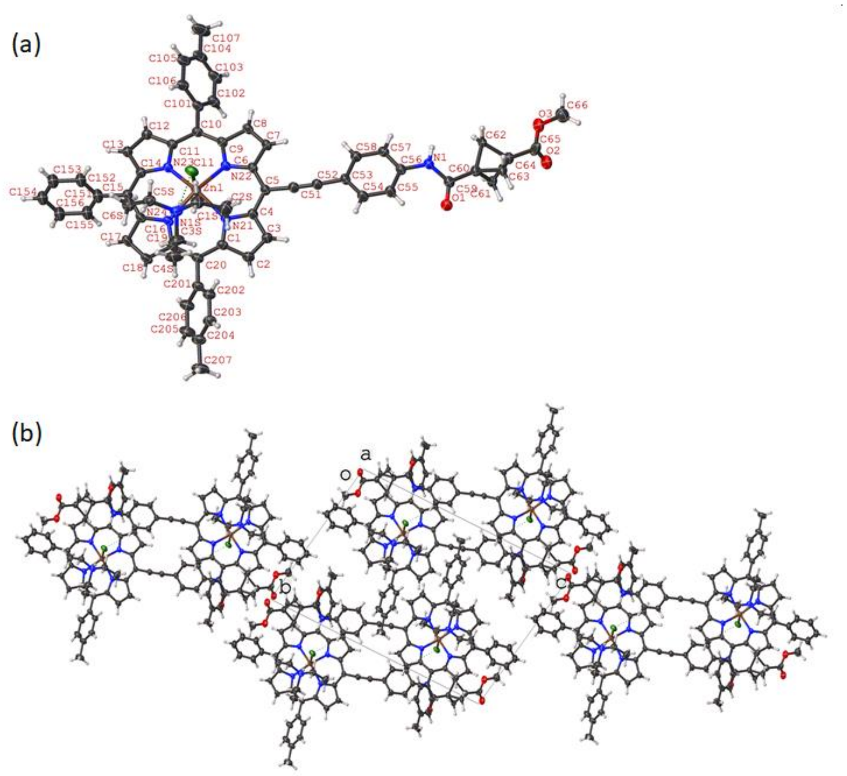

Fig. S142 (a) Molecular structure of BCP porphyrin monomer **45** with all non-hydrogen atoms labelled (thermal displacement 50%). (b) Moiety packing shown with labels omitted.

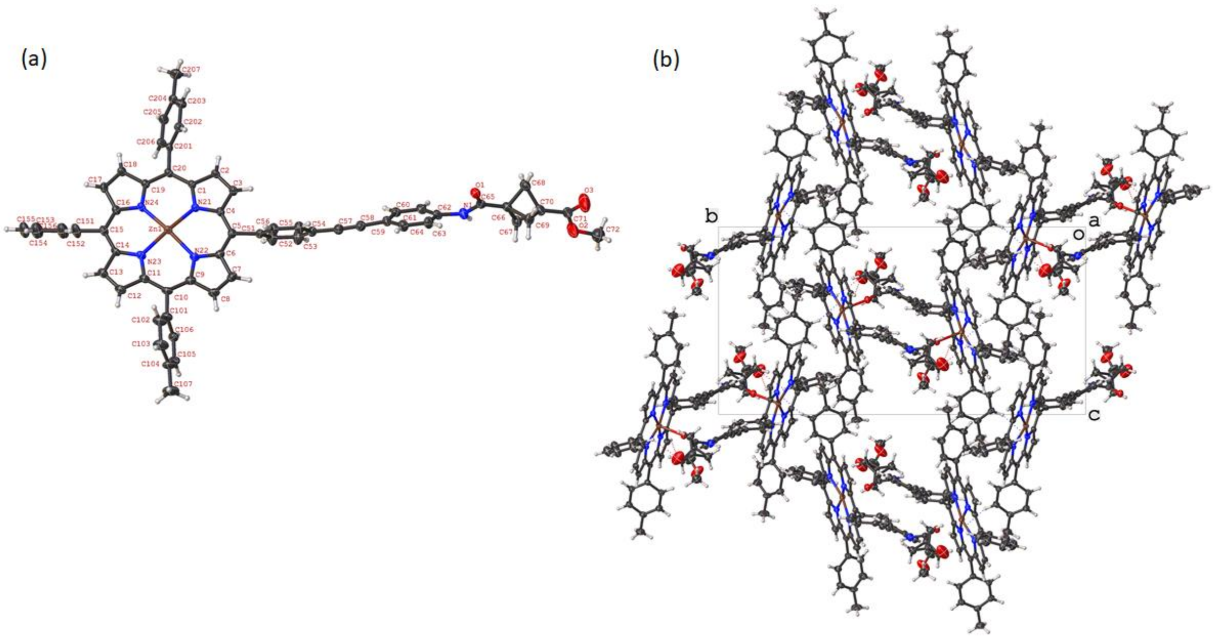

Fig. S143 (a) Molecular structure of **46** with all non-hydrogen atoms labelled (thermal displacement 50%). (b) Moiety packing shown with labels omitted.

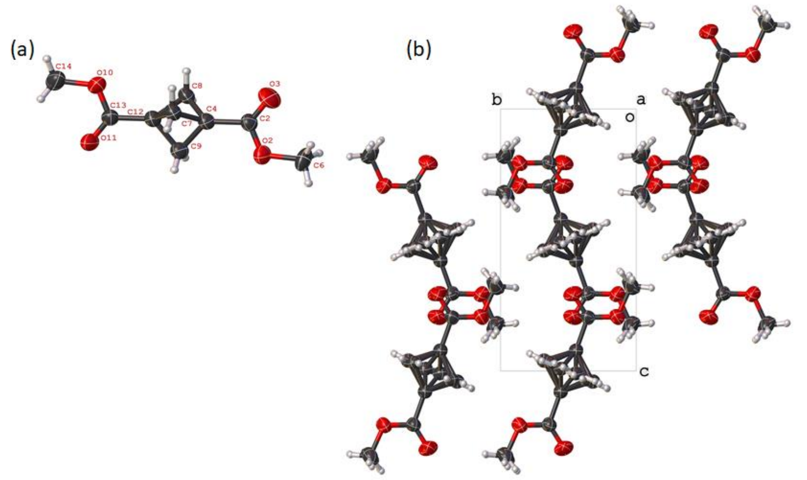

Fig. S144 (a) Molecular structure of BCP diester with all non-hydrogen atoms labelled (thermal displacement 50%). (b) Moiety packing shown with labels omitted.

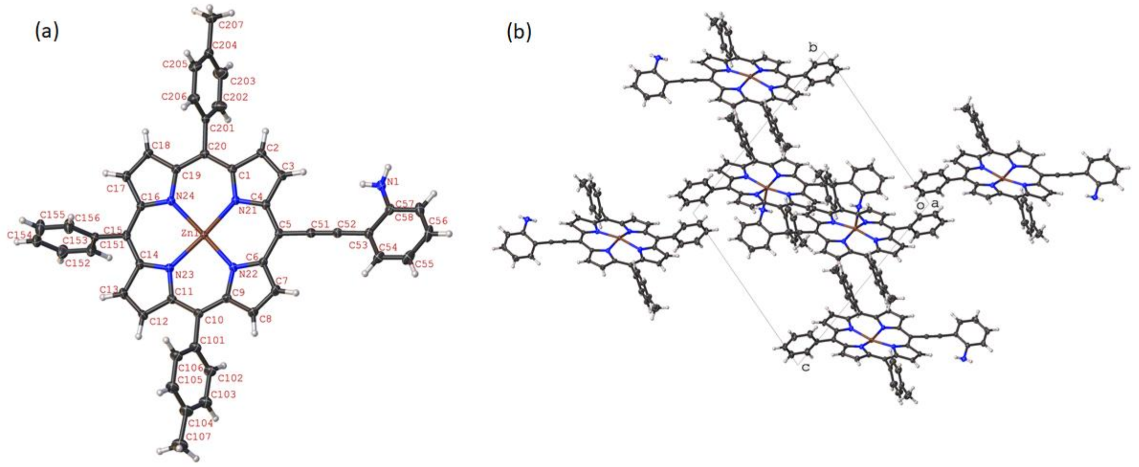

Fig. S145 (a) Molecular structure of *ortho*-amine porphyrin (**12**) with all non-hydrogen atoms labelled (thermal displacement 50%). (b) Moiety packing shown with labels omitted.

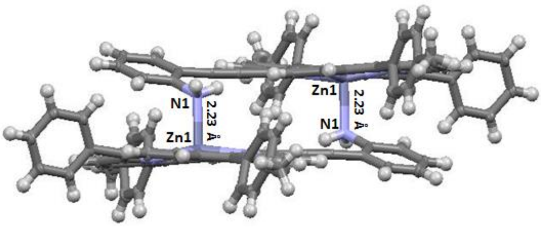

Fig. S146 Intermolecular head-to-tail interaction between the Zn metal of porphyrin unit (acceptor) and N donor atom of NH_2 moiety.

Table S1. Details of XRD data refinement.				
	**33**	**35**	**38**	**BCP-diester**
Empirical Formula	$C_{16}H_{15}NO_3$	$C_{16}H_{15}NO_3$	$C_{23}H_{18}N_2O_2$	$C_9H_{12}O_4$
Formula weight	269.29	269.29	354.39	184.19
Temperature/K	100(2)	100(2)	100(2)	298(2)
Crystal System	monoclinic	triclinic	monoclinic	orthorhombic
Space group	Cc	$P\overline{1}$	$P2_1/c$	Pmna
a/Å	27.3008(18)	8.2928(12)	9.0135(5)	7.088(7)
b/Å	5.7779(4)	9.0284(13	5.8708(3)	6.138(7)
c/Å	8.9838(6)	9.4196(13)	33.920(2)	11.040(11)
α/º	90	72.781(2)	90	90
β/º	100.351(2)	80.630(2)	90.372(3)	90
γ/º	90	81.819(2)	90	90
Volume/ $Å^3$	1394.05(16)	661.33(16)	1794.91(18)	480.3(8)
Z	4	2	4	2
ρ_{calc} g/cm^3	1.283	1.352	1.311	1.274
μ/mm^{-1}	0.089	0.094	0.085	0.100
F(000)	568.0	284.0	744.0	196.0
Crystal size/mm^3	0.26 × 0.08 × 0.08	0.23 × 0.15 × 0.05	0.17 × 0.09 × 0.05	0.4 × 0.3 × 0.2
Radiation	MoK_α	MoK_α	MoK_α	MoK_α
Wavelength/ Å	0.71073	0.71073	0.71073	0.71073
2θ/ º	3.032 to 56.722	4.564 to 55.228	4.52 to 51.968	6.638 to 51.736
Reflections collected	36314	15338	25482	3978
Independent reflections	3494	3062	3506	511
R_{int}	0.0423	0.0499	0.0665	0.0395
R_{sigma}	0.0215	0.0420	0.0471	0.0244
Restraints	3	1	2	90
Parameters	186	186	252	88
GooF	1.038	1.023	1.021	1.043
R_1 [I> 2σ (I)]	0.0290	0.0399	0.0506	0.0391
wR_2 [I> 2σ (I)]	0.0673	0.0854	0.1139	0.0869
R_1 [all data]	0.0367	0.0699	0.0925	0.0640
wR_2 [all data]	0.0715	0.0973	0.1306	0.0993
Largest peak/e Å^{-3}	0.22	0.25	0.29	0.11
Deepest hole/e Å^{-3}	-0.15	-0.24	-0.23	-0.15
Flack parameter	-0.1(3)	-	-	-

Table S2. Details of XRD data refinement.				
	**12**	**13**	**45**	**46**
Empirical Formula	$C_{96}H_{66}N_{10}Zn_2$	$C_{17}H_{14}INO_3$	$C_{62}H_{57}ClN_6O_3Zn$	$C_{62}H_{45}N_5O_3Zn$
Formula weight	1490.32	407.19	1034.95	973.40
Temperature/K	100(2)	100(2)	100(2)	100(2)
Crystal System	triclinic	Orthorhombic	triclinic	monoclinic
Space group	$P\overline{1}$	Pbca	$P\overline{1}$	$P2_1/c$
a/Å	14.5004(5)	9.4896(7)	8.6271(4)	13.5354(8)
b/Å	15.7893(6)	11.2295(8)	15.3786(6)	29.1359(18)
c/Å	18.4632(7)	28.340(2)	22.6212(9)	14.9364(10)
α/º	105.7570(10)	90	102.505(2)	90
β/º	106.3710(10)	90	91.359(2)	94.587(2)
γ/º	93.4260(10)	90	104.606(2)	90
Volume/ $Å^3$	3860.6(2)	3020.0(4)	2825.6(2)	5871.5(6)
Z	2	8	2	4
ρ_{calc} g/cm^3	1.282	1.791	1.216	1.101
μ/mm^{-1}	0.677		1.422	0.463
F(000)	1544.0	1600	1084.0	2024.0
Crystal size/mm^3	0.27 × 0.16 × 0.05	0.6 x 0.5 x 0.03	0.4 × 0.09 × 0.05	0.47 × 0.2 × 0.2
Radiation	MoK_α	CuKα	CuKα	MoK_α
Wavelength/ Å	0.71073	1.54178	1.54178	0.71073
2θ/ º	2.71 to 52.226	3.119 to 68.360	4.014 to 133.506	3.018 to 52.224
Reflections collected	234315	50812	53860	103497
Independent reflections	15291	2770	9918	11643
R_{int}	0.0752,	0.0855	0.0697,	0.0733
R_{sigma}	0.0354	0.0313	0.0466	0.0436
Restraints	228	54	201	84
Parameters	1049	200	733	685
GooF	1.033	1.260	1.025	1.037
R_1 [I> 2σ (I)]	0.0413	0.0819	0.0532	0.0574
wR_2 [I> 2σ (I)]	0.0938	0.2405	0.1436	0.1301
R_1 [all data]	0.0654	0.0854,	0.0584,	0.0807
wR_2 [all data]	0.1041	0.2422	0.1480	0.1405
Largest peak/e $Å^{-3}$	0.87	1.593	0.79	0.83
Deepest hole/e $Å^{-3}$	-0.57	1.649	-0.80	-0.66
Flack parameter	-	-	-	-

5. References

1. M. Bliese, J. Tsanaktsidis, *Aust. J. Chem*., **1997**, *50*, 189–192.
2. C. Beinat, S. D. Banister, J. Hoban, J. Tsanaktsidis, A. Metaxas, A. D. Windhorst, M. Kassiou, *Bioorg. Med. Chem. Lett*., **2014**, *24*, 828–830.
3. A. D. Adler, F. R. Longo, J. D. Finarelli, J. Goldmacher, J. Assour, L. Korsakoff, *J. Org. Chem*., **1967**, *32*, 476–476.
4. S. Mathew, M. R. Johnston, *Chem. Eur. J*., **2009**, *15*, 248–253.
5. C. H. Lee, J. S. Lindsey, *Tetrahedron*, **1994**, *50*, 11427–11440.
6. C. Brückner, J. J. Posakony, C. K. Johnson, R. W. Boyle, B. R. James, D. Dolphin, *J. Porphyrins Phthalocyanines*, **1998**, *2*, 455–465.
7. W. W. Kalisch, M. O. Senge, *Angew. Chem. Int. Ed*., **1998**, *37*, 1107–1109.
8. S. Plunkett, K. Dahms, M. O. Senge, *Eur. J. Org. Chem*., **2013**, *8*, 1566–1579.
9. A. G. Hyslop, M. A. Kellett, P. M. Lovine, M. J. Therien, *J. Am. Chem. Soc*., **1998**, *120*, 12676–12677.
10. A. Ryan, A. Gehrold, R. Perusitti, M. Pintea, M. Fazekas, O. B. Locos, F. Blaikie, M. O. Senge, *Eur. J. Org. Chem*., **2011**, 5817–5844.
11. G. Costantino, K. Maltoni, M. Marinozzi, E. Camaioni, L. Prezeau, J.-P. Pin, R. Pellicciari, *Bioorg. Med. Chem*., **2001**, *9*, 221–227.
12. H. Hope, *Prog. Inorg. Chem.*, **2007**, 1–19.
13. (a) *Saint*, Version 8.37a., Bruker AXS, Inc., Madison, WI, **2013**; (b) *SADABS*, version 2016/2., Bruker AXS, Inc, Madison, WI,, **2014**; (c) *APEX3*, Version **2016**.9-0., Bruker AXS, Inc., Madison, WI, **2016**.
14. (a) O. V. Dolomanov, L. J. Bourhis, R. J. Gildea, J. A. K. Howard, H. Puschmann, *J. Appl. Crystallogr.*, **2009**, *42*, 339–341; (b) G. Sheldrick, *Acta Cryst. Sect. A*, **2015**, *71*, 3–8.
15. C. F. Macrae, I. J. Bruno, J. A. Chisholm, P. R. Edgington, P. McCabe, E. Pidcock, L. Rodriguez-Monge, R. Taylor, J. van de Streek, P. A. Wood *J. Appl. Cryst*., **2008**, *41*, 466-470.
